# Integrative taxonomy of New World *Euplectrus* Westwood (Hymenoptera, Eulophidae), with focus on 55 new species from Area de Conservación Guanacaste, northwestern Costa Rica

**DOI:** 10.3897/zookeys.485.9124

**Published:** 2015-03-10

**Authors:** Christer Hansson, M. Alex Smith, Daniel H. Janzen, Winnie Hallwachs

**Affiliations:** 1 Scientific Associate, Natural History Museum, Cromwell Road, London SW7 5BD, United Kingdom; 2 Museum of Biology (Entomology), Lund University, Lund, Sweden; 3 Department of Integrative Biology, University of Guelph, Guelph, ON N1G 2W1 Canada; 4 Department of Biology, University of Pennsylvania, Philadelphia, PA 19104-6018 USA

**Keywords:** Chalcidoidea, Eulophinae, Euplectrini, DNA barcoding, Lepidoptera, gregarious caterpillar ectoparasitoids, tropical biodiversity, Nearctic, Neotropical

## Abstract

90 species of *Euplectrus* are treated: 55 newly described, all from Area de Conservación Guanacaste (ACG), and 35 previously described species, of which 20 occur in ACG. Three of the previously described species (*Euplectrus brasiliensis* Ashmead, *Euplectrus hircinus* (Say), *Euplectrus ronnai* (Brèthes)) have unknown status, owing to missing or severely damaged type material. The new species, all authored by C. Hansson, are: *Euplectrus alejandrovalerioi*, *Euplectrus alexsmithi*, *Euplectrus alvarowillei*, *Euplectrus andybennetti*, *Euplectrus andydeansi*, *Euplectrus annettewalkerae*, *Euplectrus billbrowni*, *Euplectrus bobwhartoni*, *Euplectrus carlosarmientoi*, *Euplectrus carlrettenmeyeri*, *Euplectrus charlesmicheneri*, *Euplectrus charlesporteri*, *Euplectrus chrisdarlingi*, *Euplectrus chrisgrinteri*, *Euplectrus corriemoreauae*, *Euplectrus daveroubiki*, *Euplectrus davesmithi*, *Euplectrus davidwahli*, *Euplectrus dianariasae*, *Euplectrus donquickei*, *Euplectrus eowilsoni*, *Euplectrus garygibsoni*, *Euplectrus gavinbroadi*, *Euplectrus gerarddelvarei*, *Euplectrus henrytownesi*, *Euplectrus howelldalyi*, *Euplectrus hugokonsi*, *Euplectrus iangauldi*, *Euplectrus jacklonginoi*, *Euplectrus jesusugaldei*, *Euplectrus jimwhitfieldi*, *Euplectrus jjrodriguezae*, *Euplectrus johnheratyi*, *Euplectrus johnlasallei*, *Euplectrus johnnoyesi*, *Euplectrus josefernandezi*, *Euplectrus lubomirmasneri*, *Euplectrus markshawi*, *Euplectrus mikegatesi*, *Euplectrus mikeschauffi*, *Euplectrus mikesharkeyi*, *Euplectrus ninazitaniae*, *Euplectrus pammitchellae*, *Euplectrus paulhansoni*, *Euplectrus paulheberti*, *Euplectrus paulhurdi*, *Euplectrus philwardi*, *Euplectrus robbinthorpi*, *Euplectrus ronaldzunigai*, *Euplectrus roysnellingi*, *Euplectrus scottshawi*, *Euplectrus sondrawardae*, *Euplectrus sydneycameronae*, *Euplectrus victoriapookae*, *Euplectrus wonyoungchoi*. The species are described or redescribed, and thoroughly and uniformly illustrated, and included in two identification keys, one for females and one for males. Lectotypes are designated for eight species: *Euplectrus catocalae* Howard (♂), *Euplectrus junctus* Gahan (♀), *Euplectrus leucotrophis* Howard (♂), *Euplectrus marginatus* Ashmead (♀), *Euplectrus pachyscaphus* Girault (♀), *Euplectrus platyhypenae* Howard (♂), *Euplectrus semimarginatus* Girault (♀), *Heteroscapus ronnai* Brèthes (♂). One synonym is established: *Euplectrus walteri* Schauff is a junior synonym of *Euplectrus testaceipes* (Cameron). Brief image notes and host records are provided on the natural history of the wasps as well as the details of their morphology. Hosts are known for 74 *Euplectrus* species.

## Introduction

Larvae of the genus *Euplectrus* develop as ectoparasitoids on various species of caterpillars that live exposed on their food plants (Schauff and Janzen 2001) (Figs 5–33). The species are usually gregarious, with five to several hundred larvae per host caterpillar (Schauff and Janzen 2001). In a few species only one egg is laid per host (Gerling and Limon 1976). Some species of *Euplectrus* females temporarily paralyze the host (Chattergee 1945) while other species do not (Clausen 1940). Prior to oviposition, the female injects a venom that inhibits further ecdyses of the host caterpillar. Regardless of the fate of the parasitoid larvae, this renders the host caterpillar incapable of molting (which would shed the parasitoid larvae), and it eventually dies (Coudron and Puttler 1988, Coudron et al. 1994) but not until after the wasp larvae are full-sized and ready to spin their cocoons. The eggs have a pedicel with an anchor at the terminus and are fastened to the host with the anchor under the cuticle, but above the hypodermis (Gerling and Limon 1976). They are usually placed on the dorsum of the caterpillar. The parasitoid larvae remain at their oviposition site throughout their development (Gerling and Limon 1976), sucking haemolymph out of the caterpillar. When fully grown, the larvae usually migrate to the underside of the dead host caterpillar to spin a loose cocoon prior to pupation (Gerling and Limon 1976). In some species the larvae spin a communal ruff of cocoons around the dying host, in which they pupate (Schauff and Janzen 2001). The ability of *Euplectrus* to spin a cocoon is unique among the Eulophidae. The silken material in the cocoon is made by modified malpighian tubules and is secreted through the anal opening (Thomsen 1927). The parasitoid larva undergoes 3–5 molts and the time from egg-laying to adult is two weeks or less (Gerling and Limon 1976).

*Euplectrus* is a cosmopolitan genus with 141 valid species names prior to this study (Noyes 2014), and forms the bulk of the tribe Euplectrini, which currently includes six additional genera in the Americas (Wijesekara and Schauff 1995). Members of *Euplectrus* are easy to recognize and differentiate from other Eulophinae through a combination of three easy-to-see characters: hind tibial spurs very long and strong (the longest spur is at least half as long as hind tarsus and serves to help anchor the wasp to the back of the caterpillar while ovipositing), scutellum without lateral grooves/pit-rows, and propodeum with a single strong median carina. *Euplectrus* is a morphologically conservative genus, frequently displaying small interspecific morphological differences. These slight differences among species have been largely overlooked, which, coupled with non-existent or insufficient tools for identification, has resulted in numerous erroneous host and distribution records in the literature. Due to the difficulties associated with the identification of *Euplectrus* species, only biological information about the type series is included for previously described species.

American species of *Euplectrus* have never been comprehensively treated, neither in North, nor in Central or South America. Prior to this publication, 35 species were known from the entire American continent. Of these 20 were recorded from Costa Rica, and mostly from Area de Conservación Guanacaste (ACG), the geographic focus here. Girault (1916) included all eight species known from North America in a key, and Schauff and Janzen (2001) treated the known Costa Rican species and included them in a key. These are the only keys to *Euplectrus* species available for the Americas. *Euplectrus* species have also been included in catalogues covering different parts of the Americas, in the Nearctic by Peck (1951, 1963) and Burks (1979), and in the Neotropics by De Santis (1967, 1979, 1980b) and De Santis and Fidalgo (1994). Due to misidentifications of both wasps and hosts, many distribution records and host records in these catalogues are erroneous, and we do not discuss them.

All patronyms assigned here are offered in deep appreciation from Janzen and Hallwachs for the Hymenoptera portion of the “taxasphere” (Janzen 1993) having collectively offered Hymenoptera knowledge and identification for the ongoing biodiversity inventory and conservation of ACG for more than five decades.

Interim species-level epithets of hosts and *Euplectrus* species are not italicized, so as to distinguish them from published scientific names.

## Methods

The specimens described in this study are entirely reared from ACG in Costa Rica (see http://janzen.sas.upenn.edu for details for any particular specimen, to be found by its voucher code). Host caterpillars, ecological information, and morphological data, along with DNA barcodes (standardised region of the cytochrome c oxidase I (COI) gene) when available, are used for species determination and are available in the project website: http://janzen.sas.upenn.edu/caterpillars/database.lasso. See Janzen et al. 2009 for a description of the entire ACG inventory process, which captures any and all parastoids in wild-caught caterpillars. The newly eclosed wasps were killed by freezing at the field site where reared, and kept in alcohol until processed for morphological analysis. The latter process included dehydration through an alcohol series followed by air-drying and glue-mounting on paper cards as described by Noyes (1982).

The species descriptions made here were based on morphology, however, when available we also analyzed DNA sequence data from the standard DNA barcode region for animals (the 5’ region of the cytochrome c oxidase I (COI) gene, Hebert et al. 2003). DNA barcode sequences for all available ACG inventory *Euplectrus* specimens were obtained using DNA extracts prepared from single legs using a glass fibre protocol (Ivanova et al. 2006). Following total genomic DNA extraction, DNA was re-suspended in 30 μl of dH_2_O, and a 658-bp region near the 5’ terminus of the COI gene was amplified using standard primers (LepF1–LepR1) following established protocols (Smith et al. 2006, 2007, 2008). If the initial 658 bp amplification was unsuccessful, smaller, overlapping sequences were generated using internal primers. If each of these smaller amplifications was successful a composite sequence was generated. However in cases where only one read amplified, this shorter sequence was used. All information for the sequences associated with each individual specimen (including GenBank accessions) can be retrieved from the Barcode of Life Data System (BOLD; www.boldsystems.org) (Ratnasingham and Hebert 2007) using the following public DOI: 10.5883/DS-ASEUPL.

The NJ trees presented (Fig. 35 & Suppl. material 1) are phenetic drawings of similarity (or distance) of barcodes, but they are not phylogenetic hypotheses. While, in some cases, the branching pattern may in fact resemble a proper phylogenetic hypothesis derived using multiple information sources and more evolutionary tree-building algorithms (e.g. Maximum Parsimony, Maximum Likelihood or Bayesian) it is not on it’s own an evolutionary branching diagram. They are presented here as visual representations of the COI distances, and as such are useful in illustrating the divergence of species from each other (i.e. a visual confirmation that the species hypotheses erected have different DNA barcodes), but the NJ trees should not be used on their own to state sister species relationships. This requires more information and better evolutionary algorithms.

Observations of the adults were made through a stereomicroscope, Nikon^©^ SMZ 1000 with a halogen ring light. The colour photos of adults were taken with a DS-Fi1 camera mounted on the stereomicroscope and the light source for the photos was a dome light made from a description by Kerr et al. (2008). Each picture was made from several photos taken at different levels of focus, and merged using Helicon Focus^©^. Micrographs are from uncoated specimens analyzed in low vacuum, with a JEOL^©^ JSM 5600 LV scanning microscope. Caterpillar, cocoon and wasp larvae images (Figs 5–33) were taken of living specimens in the field, originally with Kodachrome II film and a Nikon camera, and with a Canon digital camera after about 1995.

### Acronyms

BMNH the Natural History Museum, London, United Kingdom (Natalie Dale-Skey)

CNC Canadian National Collection of Insects and Arachnids, Ottawa, Canada (John T. Huber)

INBio Instituto Nacional de Biodiversidad, Santo Domingo de Heredia, Costa Rica (Ronald Zuñiga)

MACN Museo Argentino de Ciencias Naturales “Bernardino Rivadavia”, Buenos Aires, Argentina (Juan José Martínez)

MIUCR Museo de Insectos, Universidad de Costa Rica, San José, Costa Rica (Paul Hanson)

MZLU Museum of Biology (Entomology), Lund, Sweden

UCR University of California, Riverside, USA (Douglas A. Yanega)

USNM United States National Museum of Natural History, Washington, D.C., USA (Michael E. Gates)

### Abbreviations (Figs 1–4)

DO = largest diameter of one posterior ocellus; HE = height of eye in frontal view; HH = height of head in frontal view; LC = length of scape; LG = length of the gaster; LP = length of petiole; LT = length of hind tarsus; **LT1** = length of first tarsomere on hind leg, LT2 = length of second tarsomere on hind leg, etc.; MM = length of the mesosoma, measured along the median mesosoma, from the pronotal collar carina to posterior margin of the propodeum; MS = malar space; OOL = the distance between eye and posterior ocellus; PM = length of the postmarginal vein; POL = the distance between posterior ocelli; POO = the distance between posterior ocelli and occipital margin; ST = length of the stigmal vein; TS1 = length of longest tibial spur; TS2 = length of shortest tibial spur; WE = width of eye; **WF** = width of frons, in frontal view the largest distance between eyes; WH = width of the head, measured at widest part; WM = width of mouth opening; WP = width of petiole, measured across widest part; WS = width of scape, measured across widest part; WT = width of the thorax, measured across the widest part which is usually just in front of the attachment point of the fore wing, the “shoulders”; nm = not measurable.

**Lower face** as defined by Gibson (1997), i.e. the part below an imaginary line from eye to eye touching ventral edge of toruli. Additional terminology is explained and illustrated on http://www.neotropicaleulophidae.com/.

ACG = Area de Conservación Guanacaste, 120.000 ha of conserved dry forest, cloud forest and rain forest, and intergrades, in northwestern Costa Rica, from the Pacific slopes to Caribbean lowlands (0–2000 m elevation (http://www.acguanacaste.ac.cr and Janzen et al. 2009).

The male has pores on the scape (Figs 426, 427), presumably for purposes of communication with females. The part of the scape to which these pores are confined is referred to as the “**sensory area**” in the descriptive text.

The descriptions are based on the holotype, and if the other sex is present, on one of the paratypes.

## Results

### Biology

Population biology of ACG *Euplectrus* will be discussed in the context of total parasitization (e.g. Smith et al. 2006, 2007, 2008, 2012, Fernandez-Triana et al. 2014) of the ACG caterpillar fauna in later publications. In general terms, in those cases where we have achieved large samples of a particular species of caterpillar, the percent parasitized by a species of *Euplectrus* ranges from well less than 1% to as high as 10%. Equally striking is that all species of *Euplectrus* reared to date are either (usually) host-specific to a particular species of caterpillar or (occasionally) to a particular life-form of caterpillar within a genus or family (Table 1, Fig. 34), and those usually feeding on a narrow range of food plants. It is noteworthy that no ACG *Euplectrus* has been reared from a butterfly caterpillar (Hesperiidae plus all higher butterflies), Arctiinae (now in Erebidae, the Arctiidae of old), Saturniidae, or hairy caterpillars (irrespective of the family), with one exception (*Euplectrus josefernandezi* parasitizing *Euglyphis jessiehillae*, which does not spin a silk cocoon, but rather, pupates naked below the cadaver of its host that is appressed to the leaf (Figs 32–33).

**Table 1.** Hosts with their *Euplectrus* parasitoids.

Host	*Euplectrus* species
**Arctiidae**	
Arctiidae indet.?doubtful record	*Euplectrus leucotrophis*
**Erebidae**	
*Alabama argillacea*	*Euplectrus comstockii*
*Antapistis* Poole10	*Euplectrus ninazitaniae*
*Antiblemma amarga*	*Euplectrus mikegatesi*
*Antiblemma ceras*	*Euplectrus carlrettenmeyeri*
*Antiblemma* Poole03	*Euplectrus ronaldzunigai*
*Antiblemma* Poole21DHJ02	*Euplectrus dianariasae*
*Antiblemma* Poole22	*Euplectrus gavinbroadi*
*Anticarsia gemmatalis*	*Euplectrus puttleri*
*Catocala* sp.	*Euplectrus catocalae*
*Ceromacra* Poole02	*Euplectrus davesmithi*
*Eulepidotis caeruleilinea*	*Euplectrus chrisgrinteri*
*Gonodonta holosericea*	*Euplectrus johnnoyesi*
*Gonodonta sicheas*	*Euplectrus johnnoyesi*
*Gonodonta sinaldus*	*Euplectrus johnnoyesi*
*Hypena* Poole36	*Euplectrus davidwahli*
*Letis mycerina*	*Euplectrus chrisdarlingi*, *Euplectrus roysnellingi*
*Oraesia serpens*	*Euplectrus johnnoyesi*
*Oxidercia thaumantis*	*Euplectrus garygibsoni*
*Plusiodonta clavifera*	*Euplectrus sydneycameronae*
*Rejectaria splendida*	*Euplectrus paulhansoni*
*Rejectaria* Janzen06	*Euplectrus paulhansoni*
*Sanys irrosea*	*Euplectrus sondrawardae*
*Trauaxa lua*	*Euplectrus scottshawi*
*Tyrissa* Poole01	*Euplectrus annettewalkerae*
**Euteliidae**	
*Paectes lunodes*	*Euplectrus josei*
*Paectes nana*	*Euplectrus josei*
**Geometridae**	
*Cyclophora* Janzen14	*Euplectrus victoriapookae*
*Iridopsis herse*	*Euplectrus alejandrovalerioi*
*Isochromodes* sheilaDHJ05	*Euplectrus hansoni*
*Oxydia apidania*	*Euplectrus carlosarmientoi*
*Oxydia* sociataDHJ02	*Euplectrus orias*
*Prochoerodes marciana*	*Euplectrus charlesmicheneri*
*Sicya medangula*	*Euplectrus charlesmicheneri*
*Sphacelodes vulneraria*	*Euplectrus anae*
*Thysanopyga cermala*	*Euplectrus henrytownesi*
**Lasiocampidae**	
*Euglyphis jessiehillae*	*Euplectrus josefernandezi*
**Noctuidae**	
*Argyrosticta* aurifundensDHJ02	*Euplectrus eowilsoni*
*Argyrosticta bellinita*	*Euplectrus eowilsoni*
*Argyrosticta* scionePoole01	*Euplectrus eowilsoni*
*Argyrosticta vauaurea*	*Euplectrus eowilsoni*
*Bagisara pacifica*	*Euplectrus pammitchellae*
*Bagisara tristicta*	*Euplectrus bobwhartoni*
*Baniana signiplena*	*Euplectrus philwardi*
*Callopistria floridensis*	*Euplectrus alvarowillei*
*Cecharismena zoum*	*Euplectrus lubomirmasneri*
*Concana hoshea*	*Euplectrus mariae*
*Concana* Poole01	*Euplectrus mariae*
*Concana* Poole02	*Euplectrus mariae*
*Cropia cedica*	*Euplectrus ivonae*
*Cropia connecta*	*Euplectrus ivonae*
*Cropia hadenoides*	*Euplectrus ivonae*
*Cropia rivulosa*	*Euplectrus johnheratyi*
*Ctenoplusia oxygramma*	*Euplectrus jimwhitfieldi*
*Diastema tigris*	*Euplectrus charlesporteri*
*Dyops* chromatophilaDHJ01	*Euplectrus billbrowni*
*Elaphria* Poole08	*Euplectrus wonyoungchoi*
*Euscirrhopterus poeyi*	*Euplectrus ivonae*
*Eustrotia* Poole04	*Euplectrus andydeansi*
*Eustrotia* Poole14	*Euplectrus alexsmithi*
*Hemicephalis* alesaDHJ01	*Euplectrus donquickei*, *Euplectrus howelldalyi*
*Lophomyra* Poole02	*Euplectrus eowilsoni*
*Mursa* maricaDHJ01	*Euplectrus jacklonginoi*
noctuid 11-SRNP-33495	*Euplectrus corriemoreauae*
Noctuidae indet.	*Euplectrus frontalis*
*Ozarba geta*	*Euplectrus jjrodriguezae*
*Perigea* berindaDHJ02	*Euplectrus mikesharkeyi*
*Perigea micrippia*	*Euplectrus mikesharkeyi*
*Plathypena scabra*	*Euplectrus platyhypenae*
*Pseudoplusia includens*	*Euplectrus lubomirmasneri*
*Spodoptera frugiperda*	*Euplectrus platyhypenae*
*Tarachidia bicolorata*	*Euplectrus jesusugaldei*
**Nolidae**	
*Motya* Poole02	*Euplectrus ireneae*
**Notodontidae**	
*Cargida pyrrha*	*Euplectrus markshawi*
*Chliara croesus*	*Euplectrus magdae*
*Colax* apulusDHJ01	*Euplectrus magdae*
*Dasylophia guarana*	*Euplectrus hugokonsi*, *Euplectrus magdae*
*Dasylophia* maxtlaDHJ06	*Euplectrus magdae*
*Elymiotis attenuata*	*Euplectrus andybennetti*
*Hapigiodes sigifredomarini*	*Euplectrus magdae*
*Hemiceras clarki*	*Euplectrus xiomarae*
*Hemiceras corema*	*Euplectrus xiomarae*
*Hemiceras* Janzen13	*Euplectrus xiomarae*
*Hemiceras nigrescens*	*Euplectrus daveroubiki*, *Euplectrus xiomarae*
*Hemiceras sabis*	*Euplectrus daveroubiki*, *Euplectrus xiomarae*
*Hemiceras vecina*	*Euplectrus daveroubiki*, *Euplectrus xiomarae*
*Hemiceras zula*	*Euplectrus xiomarae*
*Pentobesa* pinnaDHJ02	*Euplectrus magdae*
*Rosema attenuata*	*Euplectrus xiomarae*
*Rosema* thestiaDHJ02	*Euplectrus xiomarae*
*Sericochroa* Janzen01	*Euplectrus johnlasallei*
*Tagela cayuga*	*Euplectrus paulhurdi*
**Sphingidae**	
*Aellopos clavipes*	*Euplectrus paulheberti*
*Callionima denticulata*	*Euplectrus scottshawi*
*Cautethia spuria*	*Euplectrus floryae*, *Euplectrus ronniei*
*Enyo ocypete*	*Euplectrus mikeschauffi*, *Euplectrus floryae*
*Erinnyis alope*	*Euplectrus gerarddelvarei*
*Isoparce cupressi*	*Euplectrus junctus*
*Manduca dilucida*	*Euplectrus testaceipes*
*Manduca florestan*	*Euplectrus testaceipes*
*Manduca lanuginosa*	*Euplectrus testaceipes*
*Manduca rustica*	*Euplectrus testaceipes*
*Manduca sexta*	*Euplectrus testaceipes*
*Perigonia* ilusDHJ01	*Euplectrus floryae*
*Perigonia lusca*	*Euplectrus iangauldi*, *Euplectrus floryae*
**Tortricidae**	
*Olethreutes* Brown20	*Euplectrus robbinthorpi*

There is no evidence of pupal dormancy to pass inimical seasons, there are species of *Euplectrus* in ACG dry forest, rain forest and cloud forest, and the caterpillar species attacked range from very small noctuoids and notodontoids to very large sphingids. However, to date all species of caterpillars attacked are external feeders on living leaves. No hyperparasitoids of *Euplectrus* have been encountered, though owing to the method of rearing wild-caught caterpillars in captivity (in closed plastic bags), *Euplectrus* prepupae and pupae in their cocoons are not exposed to potential pupal hyperparasitoids. *Euplectrus* are only very rarely caught by ACG Malaise traps, despite the wasps themselves being common in the habitat where trapping has occurred.

All species of feeding (growing) *Euplectrus* larvae are green to greenish yellow, irrespective of the species of caterpillar attacked. The wasp larvae are very visible (Figs 7–26) and not easily scraped off the caterpillar. Parasitized caterpillars are active and continue to feed in captivity, but as observed by others, never molt once the eggs have been deposited into the caterpillar’s cuticle.

### DNA barcodes

Members of the genus *Euplectrus* from ACG are characterised by a homopolymer read in the 5’ region of the barcode standard (11 base pair polyT). Such a homopolymer region reduces Sanger-based sequencing success due to slippage of the Taq polymerase resulting in sequences of varying lengths after this point (Kieleczawa 2006). This homopolymer region likely accounts for the lower than expected sequencing success we have observed within this genus (70%, 170 sequences/242 specimens) compared with other experiences DNA barcoding ACG parasitoid Hymenoptera. We do not consider this as low success in DNA barcoding per se, since any gene region that possessed such a homopolymer region would likely have reduced sequencing success. In addition to this poly-T region, ACG *Euplectrus* are characterised by a 6 bp deletion in the 3’ end of the barcode region of COI. While the mitochondrial protein-coding COI does not usually have deletions, such an in-frame anomaly is not uncommon in some families of Hymenoptera (Quicke et al. 2012).

In two cases, species were described based on morphology and biology as there were no DNA barcode differences that could be used to differentiate amongst these two species pairs (*Euplectrus roysnellingi* from *Euplectrus daveroubiki* and *Euplectrus davesmithi* from *Euplectrus victoriapookae* (Figure 35, Suppl. material 1)). Three out of the four cases currently have a very small number of sequences and or specimens and so an increased sample size will be needed to resolve whether these cases represent incidences where viable, species-specific morphological discontinuities were not associated with differences within the standard animal DNA barcode region.

In most cases, the DNA barcodes match perfectly with what is expected from the combination of close examination of morphology/color, and biological data where the host species has been accurately identified. It was possible to DNA barcode nearly all reared species because the holotype had siblings from the same rearing, one being used as the holotype and another DNA barcoded. However, for some species the barcode analysis failed and these are hence not included in the NJ-tree, but they are nevertheless included the paper because their biology and morphology are specific. We expect that the molecular data will support these conclusions once we have fresh specimens. A current total NJ tree is available for all specimens of the DNA barcoded *Euplectrus* (Suppl. material 1). We use such an NJ tree as a device for flagging potential different species, an attention-grabber that is then followed up by close morphological and host analysis. In a few cases we were not prepared to describe what appear to be additional species, without having larger or better samples, and therefore have left them in the total specimen NJ tree as a way of indicating their existence in nature, but we have not included them in the single-specimen tree. An NJ tree of one representative barcode per species (Fig. 35) is offered to illustrate the interspecific differences for *Euplectrus* barcodes.

## Taxonomy

The addition of the 55 new species does not alter the morphological definition of *Euplectrus* (see below), and as the American species do not show a significant group variation in morphological characters a subdivision of the genus into species-groups or subgenera is not justified at this time, if ever.

### Euplectrus

Taxon classificationAnimaliaHymenopteraEulophidae

Westwood

Euplectrus Westwood, 1832. Type species *Euplectrus maculiventris* Westwood, 1832, by monotypy.
Diplectron Dahlbom, 1857. Type species *Pteromalus bicolor* Swederus, 1795, by subsequent designation. Synonymized by Dalla Torre 1898.
Heteroscapus Brèthes, 1918. Type species *Heteroscapus ronnai* Brèthes, 1918, by monotypy. Synonymized by De Santis 1980b.
Pachyscapha Howard, 1897. Type species *Pachyscapha insularis* Howard, 1897, by monotypy. Synonymized by Ferrière 1941.
Rekabia Cameron, 1904. Type species *Rekabia testaceipes* Cameron, 1904, by monotypy. Synonymized by Kerrich 1974.

#### Diagnosis.

Hind tibial spurs elongate and strong (Figs 3, 424, 425), longest spur usually at least half as long as length of hind tarsus; scutellum without lateral grooves or lines of foveae (e.g. Figs 117, 120); propodeum anteromedially with a raised “cup” - triangular to semicircular, and with a complete median carina behind cup (e.g. Figs 733–738).

#### Description.

**Figures 1–4.**
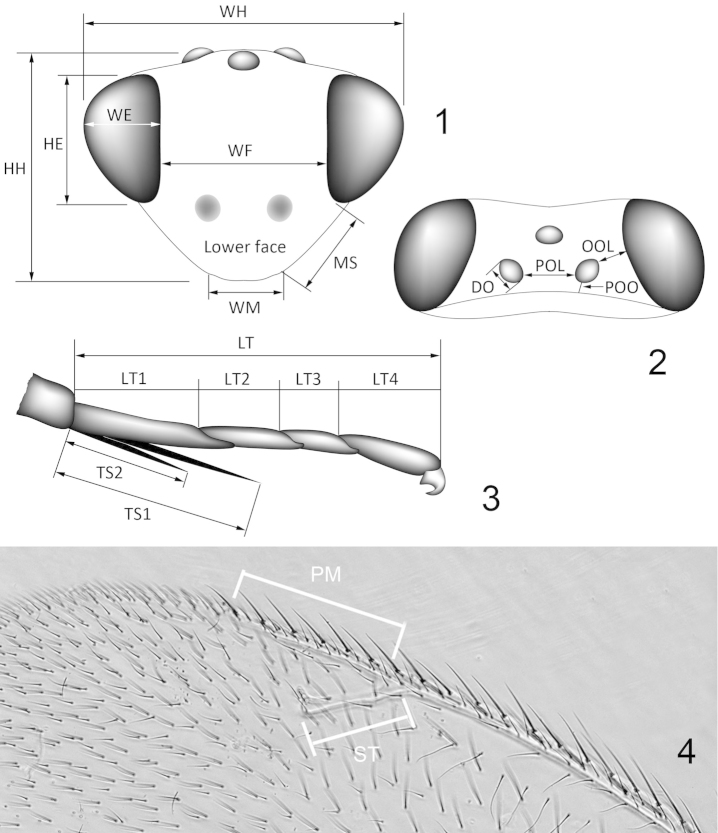
*Euplectrus* terminology, schematic illustrations: **1** head in frontal view **2** vertex **3** apex of hind leg with tibial spurs and tarsus **4** part of left fore wing. For explanation of abbreviations see text under “Abbreviations”.

**Figures 5–8.**
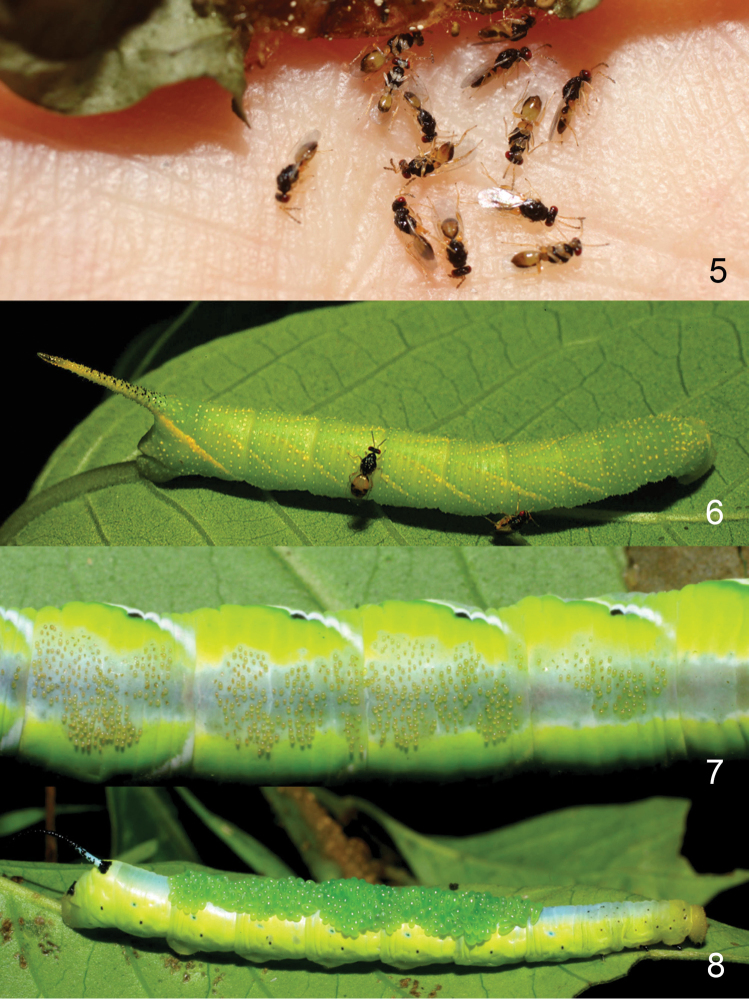
*Euplectrus* spp.: **5** freshly killed (by freezing) adult *Euplectrus paulheberti* on an author’s hand (DHJ), newly eclosed from cocoons below the cadaver of *Aellopos clavipes* (Sphingidae) (13-SRNP-10107) **6** two female *Euplectrus testaceipes* exploring a penultimate instar *Manduca barnesi* (Sphingidae) (94-SRNP-4497) for oviposition in the field; they were extremely tenacious in staying mounted on the caterpillar through hours of strong buffeting in a plastic bag on the author’s belt (DHJ) **7** early instar *Euplectrus testaceipes* where oviposited on the back of a last instar *Manduca albiplaga* (Sphingidae) (08-SRNP-16395) **8** middle instar *Euplectrus testaceipes* growing on the back of a penultimate instar *Manduca albiplaga* (Sphingidae) (08-SRNP-16396).

**Figures 9–11.**
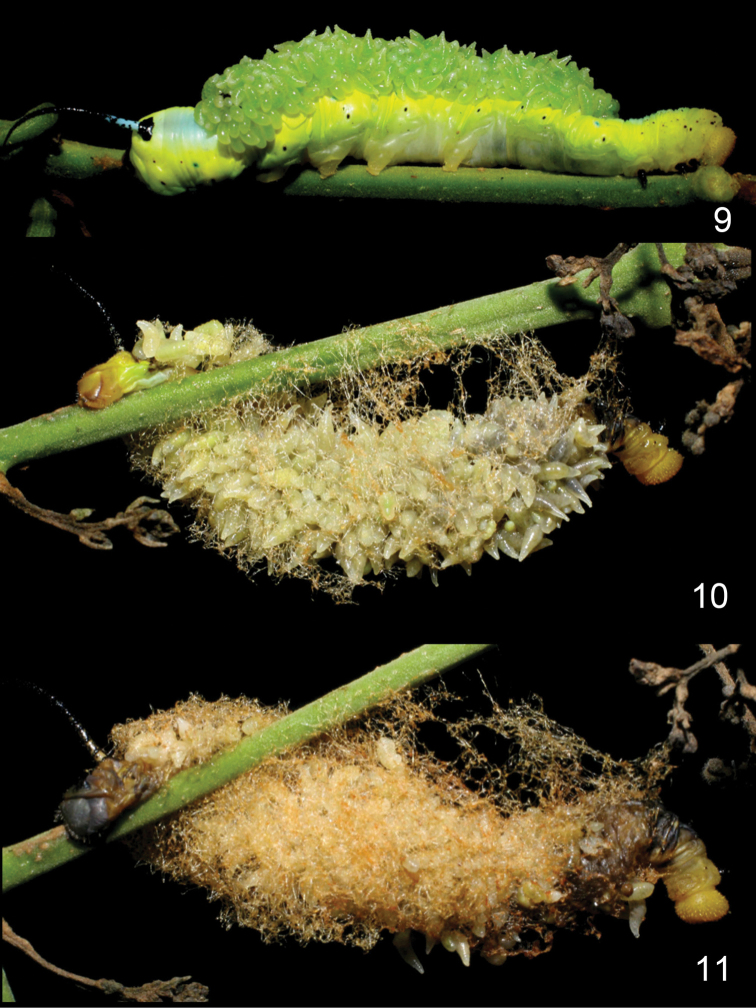
*Euplectrus testaceipes*: **9** last instar growing on the back of the caterpillar (08-SRNP-16396) in Fig. 8 **10** beginning cocoon spinning on the back of the caterpillar (08-SRNP-16396) in Fig. 8 **11** nearly completed ruff of gregarious cocoons on the back of caterpillar (08-SRNP-16396) in Fig. 8.

**Figures 12–15.**
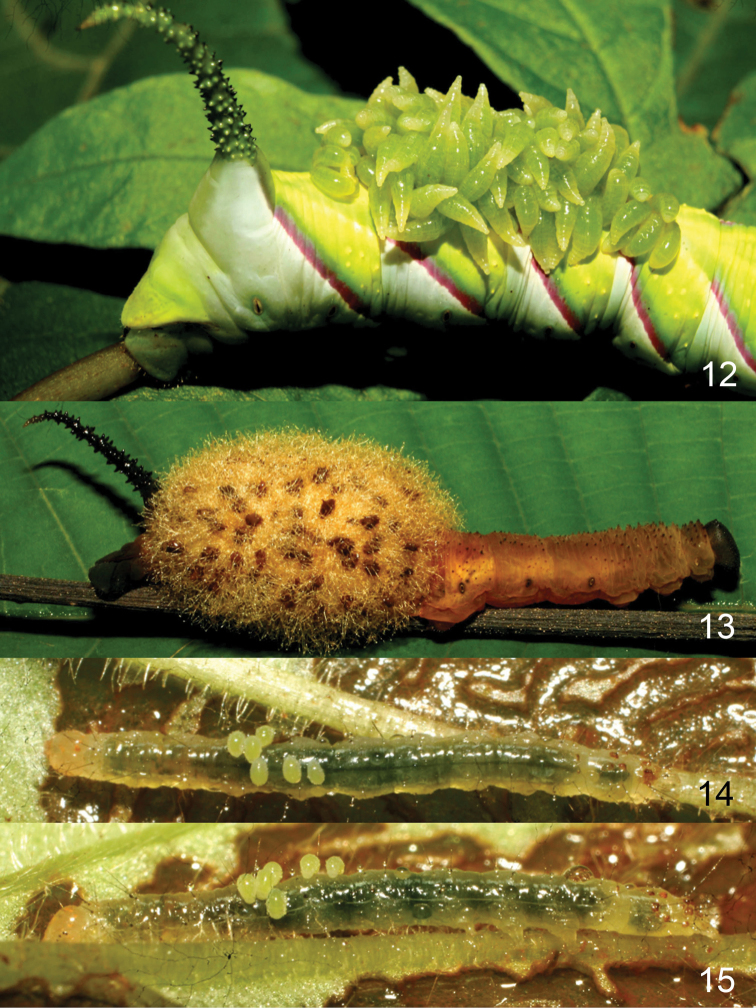
*Euplectrus* spp.: **12** close view of last instar *Euplectrus testaceipes* feeding on the back of penultimate instar *Manduca rustica* (Sphingidae) (13-SRNP-71715) **13** cocoon ruff of *Euplectrus testaceipes* on penultimate instar *Manduca florestan* (Sphingidae) (10-SRNP-72733) **14** dorsal view of mid-instar *Euplectrus davidwahli* feeding on last instar *Hypena* Poole36 (Erebidae) (11-SRNP-30665) **15** lateral view of mid-instar *Euplectrus davidwahli* feeding on last instar *Hypena* Poole36 (Erebidae) (11-SRNP-30665).

**Figures 16–18.**
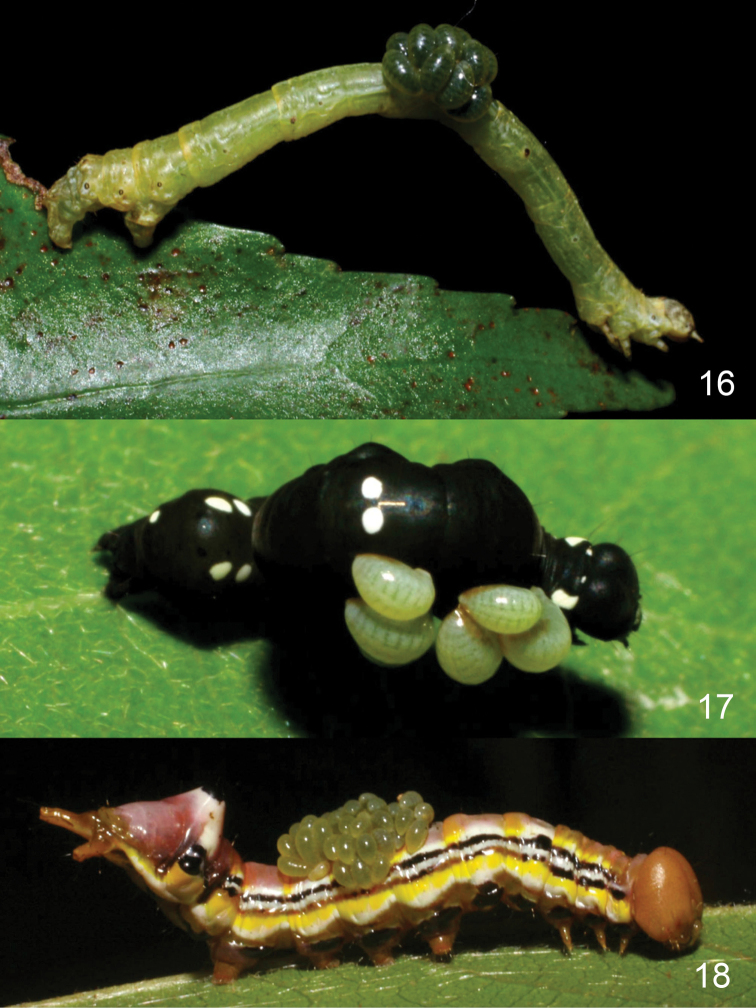
*Euplectrus* spp.: **16** last instar *Euplectrus charlesmicheneri* feeding on last instar *Sicya medangula* (Geometridae) (08-SRNP-35772) **17** last instar *Euplectrus johnnoyesi* feeding on pen-penultimate instar *Gonodonta sinaldus* (Erebidae) (07-SRNP-33589) **18** last instar *Euplectrus magdae* feeding on penultimate instar *Pentobesa* pinnaDHJ02 (Notodontidae) (05-SRNP-19353).

**Figures 19–22.**
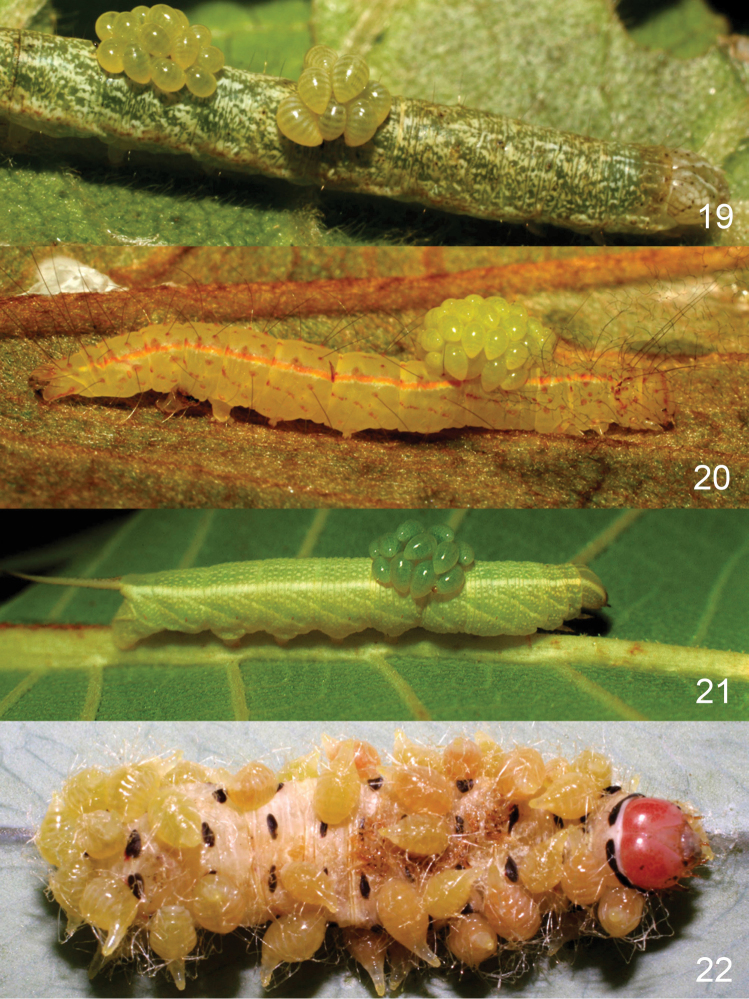
*Euplectrus* spp.: **19** two different sizes of *Euplectrus mikesharkeyi* feeding on penultimate instar *Perigea micrippia* (Noctuidae) (10-SRNP-36122); this may represent two different oviposition events or differential growth capacity that depends on the location on the caterpillar and/or whether competing parasitoids are present **20** last instar *Euplectrus mariae* feeding on last instar *Concana hoshea* (Noctuidae) (09-SRNP-72128) **21** last instar *Euplectrus floryae* feeding on penultimate instar *Enyo ocypete* (Sphingidae) (97-SRNP-4188) **22** prepupal *Euplectrus eowilsoni* beginning to spin their cocoon mass on last instar *Argyrosticta vauaurea* (Noctuidae) (07-SRNP-65277).

**Figures 23–26.**
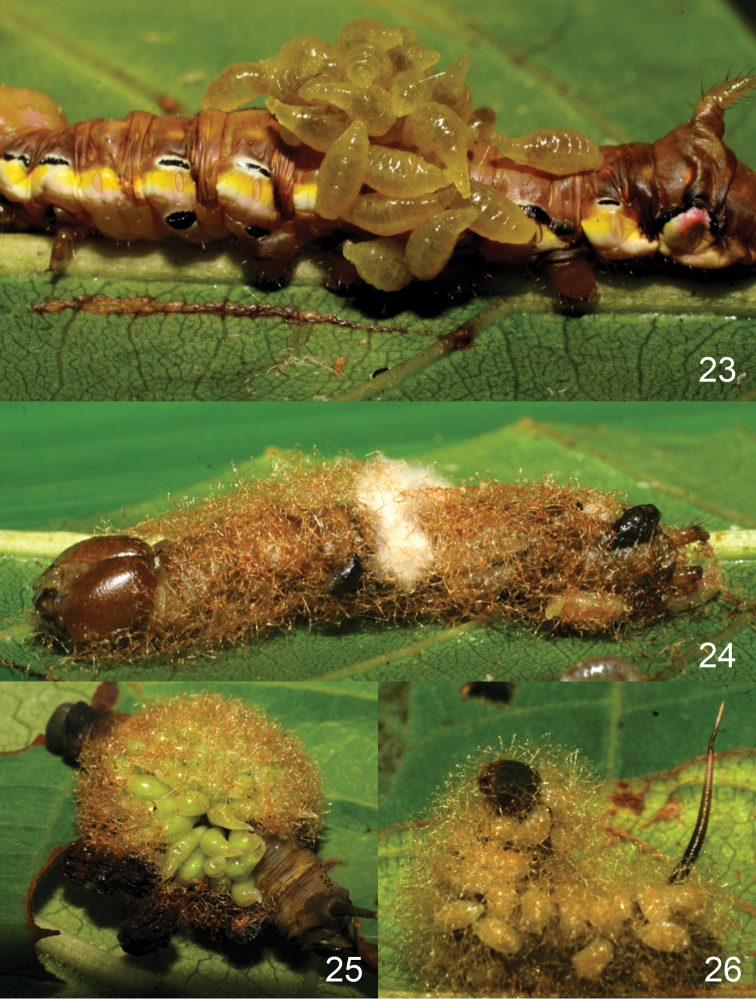
*Euplectrus* spp.: **23** prepupal *Euplectrus hugokonsi* beginning to search for their cocooning site under the cadaver of last instar *Dasylophia guarana* (Notodontidae) (07-SRNP-58948) **24** newly spun cocoons of *Euplectrus hugokonsi* enveloping the cadaver of the *Dasylophia guarana* in Fig. 23 **25** prepupal *Euplectrus iangauldi* spinning their communal ruff of cocoons around the cadaver of a penultimate instar *Perigonia lusca* (Sphingidae) (06-SRNP-22585) **26** prepupal *Euplectrus mikeschauffi* within their communal ruff of cocoons around the cadaver of a penultimate instar *Enyo ocypete* (Sphingidae) (11-SRNP-31737).

**Figure 34.**
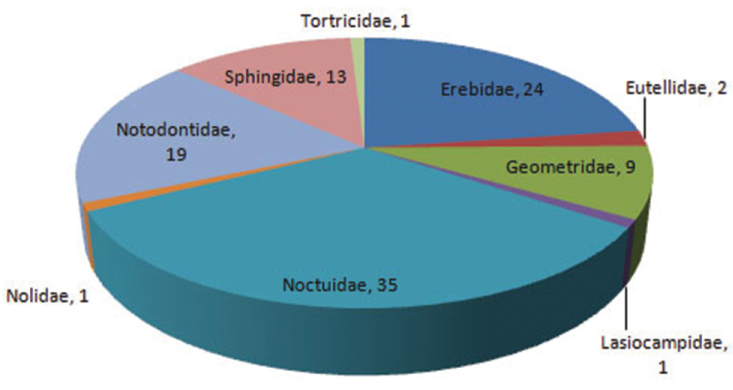
Proportion of Lepidoptera families, each with number of species, parasitized by 74 species of *Euplectrus* with known host records from the Americas (data source mainly from the ACG inventory).

Antenna in both sexes with six flagellomeres, including a 2-segmented clava (e.g. Figs 58, 59, 63); male scape usually wider than female scape and with sensory pores that are usually confined to ventral margin, but occasionally scattered over the inner or outer lateral surface; in the latter case the scape is ± strongly swollen (e.g. Figs 59, 726). Mandibles and palpi usually white, occasionally dark brown. Head dark and shiny, lower face usually ± pale, pale area reaching from eye to eye (e.g. Figs 139, 140), to confined to a pale spot below toruli (e.g. Figs 37, 38); in some species the lower face is completely dark (e.g. Figs 54, 55). Lower frons smooth, upper frons with very weak reticulation (e.g. Figs 47, 50); 1–4 rows of setae close to eyes. Occipital margin rounded (e.g. Fig. 48) to carinate (e.g. Fig. 51).

Mesoscutum with raised and distinct reticulation (e.g. Figs 49, 52); midlobe with a complete median carina and with three pairs of setae, two pairs close to notauli and one pair medially; each sidelobe with three strong setae close to posterior margin and with 5–18 scattered setae in front of strong setae; notauli distinct throughout. Scutellum 0.8–1.1× as long as wide, usually convex but occasionally almost flat, with two pairs of setae close to lateral margins (e.g. Figs 49, 52). Scutoscutellar suture wide (e.g. Figs 49, 52). Axillae with very weak reticulation (e.g. Figs 49, 52). Dorsellum 0.2× as long (medially) as wide, and 0.3× as long as length of median propodeum, ± flat, smooth and shiny, with (e.g. Figs 733–738) or without (e.g. Figs 741, 743) a groove or foveae along anterior margin, posterior margin with two oblong-rounded indentations laterally and medially with 2–4 (usually two) spines. Propodeum anteromedially with a triangular to semicircular cup that continues backwards as a narrow median carina (e.g. Figs 733–738) that splits into two carinae just before posterior margin of propodeum, carinae reach down to supracoxal flanges; propodeal callus with 5–15 setae. Hind tibial spurs long and strong, longest spur usually at least half as long as length of hind tarsus (Figs 3, 424, 425). Wings transparent, veins yellowish-white to yellowish-brown and setae dark brown to black; submarginal vein usually with five setae, occasionally four or six; speculum present and usually closed, very occasionally open below and towards base of wing; costal cell with 1–2 rows of setae on ventral surface, and fore margin with 0–8 setae close to marginal vein; with 13–36 admarginal setae, in 1–3 rows.

Petiole black with strong sculpture, 0.5–1.5× as long as wide, frequently longer in male. Female gaster ± ovate to rounded (e.g. Figs 56, 62), male gaster rounded ± triangular (e.g. Figs 125, 193). However, it should be noted that the shape and length of the gaster are affected by how the specimen has been killed and subsequently dried.

#### Hosts and biology.

Species develop as gregarious ectoparasitoids on caterpillars of several Lepidoptera families: “Arctiidae” (unconfirmed, and now a subfamily – Arctiinae – in Erebidae), Erebidae, Euteliidae, Geometridae, Lasiocampidae (one record), Noctuidae, Nolidae, Notodontidae, Sphingidae, Tortricidae (one doubtful record) – all treated in this paper. See also chapter on “Biology” above.

#### Distribution.

Cosmopolitan (Noyes 2014).

#### Key to females.

**Table:** 

1	Frons with lower face dark (black to very dark reddish-brown) and not delimited from surrounding parts of frons (e.g. Figs 480, 503)	**2**
–	Frons with at least parts of lower face distinctly paler than surrounding parts of frons (e.g. Figs 105, 241)	**19**
2(1)	Basal 3–4 flagellomeres distinctly paler than flagellomeres 5–6 (Fig. 505); mandibles dark brown	***Euplectrus zamorai* Schauff**
–	At most with basal flagellomeres 1–2 more or less pale; mandibles dark brown to white	**3**
3(2)	Hind coxa black to brown (dark brown to pale brown) (e.g. Figs 479, 720)	**4**
–	Hind coxa yellow to dark yellowish-brown (e.g. Figs 104, 138)	**8**
4(3)	Mandibles dark brown to dark yellowish-brown	**5**
–	Mandibles predominantly to completely yellowish-white to yellowish-brown, occasionally with base brown	**7**
5(4)	Hind coxa pale brown (Fig. 720)	***Euplectrus valverdei* Schauff**
–	Hind coxa black to dark brown (Figs 349, 479)	**6**
6(5)	Mandibles and hind coxa very dark brown, almost black (Fig. 349); female flagellomeres shorter, e.g. flagellomere 1 1.8× as long as wide (Fig. 352)	***Euplectrus wonyoungchoi* Hansson, sp. n.**
–	Mandibles dark yellowish-brown to brown and hind coxa dark brown (Fig. 479); female flagellomeres longer, e.g. flagellomere 1 2.5× as long as wide (Fig. 484)	***Euplectrus paulhansoni* Hansson, sp. n.**
7(4)	Mid coxa brown (Fig. 53)	***Euplectrus alvarowillei* Hansson, sp. n.**
–	Mid coxa white (Fig. 539)	***Euplectrus scottshawi* Hansson, sp. n.**
8(3)	Gaster yellowish-brown with dark brown lateral margins (Figs 639, 695)	**9**
–	Gaster completely dark brown in at least posterior ½ (e.g. Figs 328, 431)	**10**
9(8)	Posterior ocelli large, OOL/DO = 1.5, and situated away from occipital margin, POL/OOL/POO = 6.1/3.6/1.0 (Fig. 493); scutellum with very weak and superficial reticulation (Fig. 494)	***Euplectrus platyhypenae* Howard**
–	Posterior ocelli small, OOL/DO = 2.3, and situated very close to occipital margin, POL/OOL/POO = 21.0/16.0/1.0 (Fig. 704); scutellum with stronger and more distinct reticulation (Fig. 705)	***Euplectrus edithae* Schauff**
10(8)	Scutellum smooth or with very weak traces of reticulation (Fig. 338); hind leg with 4^th^ tarsomere darker than hind tibia (Fig. 325); postmarginal vein 1.2× as long as stigmal vein	***Euplectrus jacklonginoi* Hansson, sp. n.**
–	Scutellum predominantly reticulate (e.g. Figs 100, 437), occasionally very weak (*Euplectrus furnius*); hind leg with 4^th^ tarsomere with same colour as hind tibia; postmarginal vein 1.3–2.0× as long as stigmal vein	**11**
11(10)	Gaster with anterior ½ yellowish-brown with dark brown lateral margins (Fig. 431)	**12**
–	Gaster dark brown with a pale median spot or stripe anteromedially (e.g. Figs 90, 413)	**13**
12(11)	Flagellomeres longer, e.g. flagellomere 4 1.9× as long as wide (Fig. 433)	***Euplectrus lubomirmasneri* Hansson, sp. n.**
–	Flagellomeres shorter, e.g. flagellomere 4 1.2× as long as wide	***Euplectrus furnius* Walker**
13(11)	Gaster with pale spot I-shaped, i.e. with about same width throughout (Figs 90, 413, 729)	**14**
–	Gaster with pale spot ±T-shaped, i.e. with posterior part expanded (e.g. Figs 260, 283)	**16**
14(13)	Scutellum with very weak reticulation, shiny (Fig. 100); gaster with pale I-shaped spot narrow, 1.0× as wide as width of petiole (Fig. 90)	***Euplectrus andydeansi* Hansson, sp. n.**
–	Scutellum with strong reticulation (Fig. 423); gaster with pale I-shaped spot wider, 1.6× as wide as width of petiole (Figs 413, 729)	**15**
15(14)	Petiole 1.0× as long as wide; propodeum with weak and superficial reticulation (Fig. 768)	***Euplectrus josefernandezi* Hansson, sp. n.**
–	Petiole 0.7× as long as wide; propodeum with strong reticulation	***Euplectrus marginatus* Ashmead**
16(13)	Mandibles yellowish-white or yellowish-brown	**17**
–	Mandibles brown	**18**
17(16)	Petiole 0.5× as long as wide	***Euplectrus eowilsoni* Hansson, sp. n.**
–	Petiole 0.8–1.0× as long as wide	***Euplectrus sondrawardae* Hansson, sp. n.**
18(16)	Gaster with basal part of pale spot sharped to a point (Fig. 283)	***Euplectrus ninazitaniae* Hansson, sp. n.**
–	Gaster with basal part of pale spot not pointed (Fig. 368)	***Euplectrus carlowae* Schauff**
19(1)	Hind coxa dark brown (e.g. Figs 417, 662) to dark yellowish brown (Fig. 70)	**20**
–	Hind coxa white to yellowish-brown (e.g. Figs 162, 308)	**25**
20(19)	Petiole 1.5× as long as wide; posterior part of scutellum overhanging and hiding anterior part of dorsellum (see figs 68 & 69 in Schauff and Janzen 2001)	***Euplectrus rojasi* Schauff**
–	Petiole 0.6–1.0× as long as wide; entire dorsellum visible (e.g. Figs 761, 802)	**21**
21(20)	Head dark brown, lower face medially pale brown (Fig. 418); scutellum smooth laterally, posteriorly and medially, remaining parts of scutellum with very weak and superficial reticulation	***Euplectrus orias* Schauff**
–	Head black, lower face medially yellowish-brown (Fig. 265) or dark reddish-brown (Fig. 326); scutellum predominantly reticulate, or with very weak reticulation all over	**22**
22(21)	Hind femur predominantly dark brown (Fig. 662); scutellum medially with isodiametric meshes (Fig. 798)	***Euplectrus xiomarae* Schauff**
–	Hind femur yellowish-brown (e.g. Figs 70, 264); scutellum medially with elongate meshes (e.g. Figs 736, 770)	**23**
23(22)	OOL/DO = 1.2; hind coxa dark yellowish-brown (Fig. 70)	***Euplectrus andybennetti* Hansson, sp. n.**
–	OOL/DO = 1.7–1.9; hind coxa dark yellowish-brown to dark brown (Figs 264, 325)	**24**
24(23)	Scutellum with very weak and superficial reticulation, partly smooth (Fig. 338); hind coxa dark yellowish-brown (Fig. 325)	***Euplectrus jacklonginoi* Hansson, sp. n.**
–	Scutellum with strong reticulation (Fig. 273); hind coxa pale brown to dark brown (Fig. 264)	***Euplectrus markshawi* Hansson, sp. n.**
25(19)	Hind femur with apical ½ pale brown (Fig. 308)	***Euplectrus iangauldi* Hansson, sp. n.**
–	Hind femur with apical ½ white to yellowish-brown (e.g. Figs 162, 572)	**26**
26(25)	Eyes and ocelli large (Figs 408, 614), WE/WF = 0.6–0.9, OOL/DO = 0.5–0.6	**27**
–	Eyes smaller (e.g. Figs 169, 285), WE/WF = 0.3–0.5, OOL/DO = 0.8–1.8	**28**
27(26)	Eyes very large (Fig. 407), WE/WF = 0.9; vertex between posterior ocelli with one pair of setae (Fig. 408)	***Euplectrus ireneae* Schauff**
–	Eyes smaller (Fig. 613), WE/WF = 0.6; vertex behind posterior ocelli with two pairs of setae (Fig. 614)	***Euplectrus josei* Schauff**
28(26)	Pale area on lower face dark reddish-brown, small and confined to level of inner margins of toruli (e.g. Figs 163, 275)	**29**
–	Pale area on lower face more distinct and larger (e.g. Figs 78, 584)	**33**
29(28)	Hind tarsus short, tarsomere 1 shorter than either tarsomere 2 or 3 (Fig. 424)	***Euplectrus garygibsoni* Hansson, sp. n.**
–	Hind tarsus long and slender (Fig. 425), tarsomere 1 longer or with same length as either tarsomere 2 or 3	**30**
30(29)	Dorsellum with a wide groove along anterior margin, 0.4× as long as length of median dorsellum (Figs 757, 785)	**31**
–	Dorsellum without a groove along anterior margin (Figs 755, 779)	**32**
31(30)	Setae on vertex dark brown (Fig. 573)	***Euplectrus victoriapookae* Hansson, sp. n.**
–	Setae on vertex predominantly yellowish-white (Fig. 275)	***Euplectrus henrytownesi* Hansson, sp. n.**
32(30)	Hind leg with tarsomere 1 2.4× as long as tarsomere 3; petiole 1.1× as long as wide	***Euplectrus philwardi* Hansson, sp. n.**
–	Hind leg with tarsomere 1 1.6× as long as tarsomere 3; petiole 1.3× as long as wide	***Euplectrus gavinbroadi* Hansson, sp. n. / *Euplectrus dianariasae* Hansson, sp. n.**
33(28)	Hind leg with 4^th^ tarsomere darker than hind tibia (Fig. 325)	***Euplectrus jacklonginoi* Hansson, sp. n.**
–	Hind leg with 4^th^ tarsomere with same colour as hind tibia	**34**
34(33)	Gaster with posterior ½ predominantly pale, pale to dark yellowish-brown, distinctly paler than lateral margins in anterior ½ of gaster (e.g. Figs 79, 209)	**35**
–	Gaster with posterior ½ predominantly dark brown to black, with same colour as lateral margins in anterior ½ of gaster (e.g. Figs 141, 498), sometimes with apex of gaster paler	**45**
35(34)	Entire lower face pale, pale area reaching from eye to eye (Fig. 584)	***Euplectrus floryae* Schauff**
–	Lower face with a narrow black stripe, at least 0.5× the diameter of one torulus, between lower part of eye and pale area (e.g. Figs 78, 207)	**36**
36(35)	Gaster with posterior ½ pale, except sometimes dark lateral margins (e.g. Figs 79, 209)	**37**
–	Gaster with posterior ½ with a dark median spot (Fig. 45) or a dark transverse band (Fig. 158, 345)	**41**
37(36)	Pale area on lower face more extensive, reaching almost to eye (Fig. 78); scutellum with strong reticulation (Fig. 86)	***Euplectrus charlesporteri* Hansson, sp. n.**
–	Pale area on lower face smaller, reaching at most slightly outside of level of outer lateral margins of toruli (e.g. Figs 207, 231); scutellum with weak reticulation, shiny (e.g. Figs 219, 239)	**38**
38(37)	Pale area on lower face small and dark, laterally reaching to level of midpoint of toruli (Fig. 207)	***Euplectrus corriemoreauae* Hansson, sp. n.**
–	Pale area on lower face larger and more pale, laterally reaching at least to level of outer lateral margins of toruli (Figs 61, 231)	**39**
39(38)	Petiole 0.7× as long as wide; hind leg with tarsomere 1 2.4× as long as tarsomere 3	***Euplectrus jesusugaldei* Hansson, sp. n.**
–	Petiole 0.5× as long as wide; hind leg with tarsomere 1 1.5–1.8× as long as tarsomere 3	**40**
40(39)	Pale area on lower face laterally reaching half-way between lateral margin of toruli and eye margin (Fig. 689); scutellum distinctly reticulate, only with posterior margin smooth	***Euplectrus junctus* Gahan**
–	Pale area on lower face laterally reaching just outside of lateral margin of toruli (Fig. 61); scutellum smooth medially and with very weak engraved reticulation laterally (Fig. 69)	***Euplectrus bobwhartoni* Hansson, sp. n.**
41(36)	Posterior part of gaster with a dark band reaching from side to side (Figs 158, 345)	**42**
–	Posterior part of gaster with a dark median spot (e.g. Figs 45, 718)	**43**
42(41)	Head with pale area on lower face reaching half-way between outer margin of toruli and eye margin (Fig. 343)	***Euplectrus jimwhitfieldi* Hansson, sp. n.**
–	Head with pale area on lower face confined to part below toruli, does not reach outside level of lateral margins of toruli (Fig. 156)	***Euplectrus carlrettenmeyeri* Hansson, sp. n.**
43(41)	Head with pale area on lower face reaching almost to eye margin, leaving just a narrow dark band close to eye margin (Fig. 717)	***Euplectrus frontalis* Howard**
–	Head with pale area on lower face confined to part below toruli, does not reach outside level of lateral margins of toruli (Fig. 44)	**44**
44(43)	Gaster in anterior ½ with dark lateral margins each as wide as median pale area (Fig. 45)	***Euplectrus alexsmithi* Hansson, sp. n.**
–	Gaster in anterior ½ with dark lateral margins much narrower than median pale area	***Euplectrus pachyscaphus* Girault**
45(34)	Entire lower face pale, pale area reaching from eye to eye (e.g. Figs 394, 496)	**46**
–	Pale area on lower face smaller, with at least a dark stripe between pale area and eye (e.g. Figs 241, 471)	**49**
46(45)	Pale area on lower face reaching to hypostomal carina, i.e. with gena below level of eye pale (Fig. 582)	***Euplectrus paulheberti* Hansson, sp. n.**
–	Pale area on lower face does not reach hypostomal carina, i.e. with part of gena close to mouth cavity dark (Fig. 581)	**47**
47(46)	Lower face with median part (area between and below toruli) reddish-brown, lateral parts yellowish-brown (Fig. 677)	***Euplectrus chapadae* Ashmead**
–	Lower face with entire median part yellowish-brown (Figs 139, 394)	**48**
48(47)	Hind leg with 1^st^ tarsomere longer, 1.8× as long as 2^nd^ and 2.9× as long as 3^rd^ tarsomere	***Euplectrus carlosarmientoi* Hansson, sp. n.**
–	Hind leg with 1^st^ tarsomere shorter, 1.4× as long as 2^nd^ and 2.0× as long as 3^rd^ tarsomere	***Euplectrus johnnoyesi* Hansson, sp. n. / *Euplectrus sydneycameronae* Hansson, sp. n. (inseparable)**
49(45)	Scutellum with median carinae (Fig. 469), *or* with a small hump posteromedially (Figs 253, 612), meshes of reticulation more or less elongate (Figs 253, 469, 612)	**50**
–	Scutellum without median carinae and evenly convex to almost flat, meshes of reticulation isodiametric (e.g. Figs 304, 307) or elongate (few species)	**52**
50(49)	Scutellum with median carinae (Fig. 469), but without a hump	***Euplectrus mikesharkeyi* Hansson, sp. n.**
–	Scutellum with a small hump posteromedially (Figs 253, 612), but without median carinae	**51**
51(50)	Eyes larger (Fig. 610), HE/MS = 2.6; POL/OOL = 2.2	***Euplectrus ivonae* Schauff**
–	Eyes smaller (Fig. 251), HE/MS = 1.8; POL/OOL = 1.6	***Euplectrus donquickei* Hansson, sp. n.**
52(49)	Pale area on lower face with median part darker than lateral parts (e.g. Figs 292, 710), pale area *usually* large, almost reaching eyes	**53**
–	Pale area on lower face not darkened medially (e.g. Figs 224, 442)	**62**
53(52)	Gaster with posterior ½ partly pale (Figs 300, 672)	**54**
–	Gaster with posterior ½ completely dark brown (e.g. Figs 294, 712)	**55**
54(53)	Vertex lateral to ocellar triangle black; propodeum with lateral panels smooth (Fig. 775); petiole 1.1× as long as wide	***Euplectrus pammitchellae* Hansson, sp. n.**
–	Vertex lateral to ocellar triangle dark reddish-brown; propodeum with lateral panels with weak reticulation; petiole 0.8× as long as wide	***Euplectrus catocalae* Howard**
55(53)	Distance between posterior ocelli and eyes large (Fig. 707), OOL/DO = 1.7	***Euplectrus puttleri* Gordh**
–	Distance between posterior ocelli and eyes smaller, OOL/DO = 1.1–1.3	**56**
56(55)	POL/POO = 5.0–6.3, OOL/POO = 2.2–3.3	**57**
–	POL/POO = 7.2–9.8, OOL/POO = 4.0–5.8	**59**
57(56)	Gaster with dark lateral margins in anterior ½ complete and wide, about as wide as width of petiole (Fig. 294)	***Euplectrus hugokonsi* Hansson, sp. n.**
–	Gaster with dark lateral margins in anterior ½, missing in posterior part, and narrow, about 0.2× as wide as width of petiole (Figs 96, 653)	**58**
58(57)	Petiole 0.7× as long as wide; hind leg with tarsus 1.6× as long as longest tibial spur	***Euplectrus ronniei* Schauff**
–	Petiole 0.9× as long as wide; hind leg with tarsus 1.8× as long as longest tibial spur	***Euplectrus chrisgrinteri* Hansson, sp. n.**
59(56)	Lower face with pale area larger, reaching almost to eye margin, leaving just a narrow dark stripe close to eye (Fig. 197)	***Euplectrus gerarddelvarei* Hansson, sp. n.**
–	Lower face with pale area smaller, reaching at most half-way between outer lateral margins of toruli and eyes (Figs 533, 624, 360)	**60**
60(59)	Lower face with pale area confined to surface below toruli (Fig. 533)	***Euplectrus robbinthorpi* Hansson, sp. n.**
–	Lower face with pale area reaching outside of level of lateral margins of toruli (Figs 360, 624)	**61**
61(60)	Hind leg with tarsomeres 1+2 3.5× as long as tarsomere 3; lower face with pale area paler (Fig. 624)	***Euplectrus mariae* Schauff**
–	Hind leg with tarsomeres 1+2 4.2× as long as tarsomere 3; lower face with pale area darker (Fig. 360)	***Euplectrus johnheratyi* Hansson, sp. n.**
62(52)	Petiole 1.1–1.3× as long as wide	**63**
–	Petiole 0.5–1.0× as long as wide	**69**
63(62)	Dorsellum with a wide groove anteriorly, 0.3× as long as length of median dorsellum (Fig. 750)	***Euplectrus davidwahli* Hansson, sp. n.**
–	Dorsellum without a groove (Fig. 781), *or* anteriorly with a narrow groove that is at most 0.2× the length of median dorsellum (Fig. 790)	**64**
64(63)	Hind leg with longest tibial spur 4.7× as long as tarsomere 3	***Euplectrus hansoni* Schauff**
–	Hind leg with longest tibial spur at most 4.2× as long as tarsomere 3	**65**
65(64)	Hind leg with longest tibial spur 3.1× as long as tarsomere 3	***Euplectrus mikegatesi* Hansson, sp. n.**
–	Hind leg with longest tibial spur at 3.5–4.2× as long as tarsomere 3	**66**
66(65)	Dorsellum with a narrow groove along anterior margin, groove 0.2× as long as length of median dorsellum (Fig. 738)	***Euplectrus annettewalkerae* Hansson, sp. n.**
–	Dorsellum without a groove along anterior margin (Figs 743, 755, 781)	**67**
67(66)	Hind leg with 4^th^ tarsomere 1.4× as long as tarsomere 2	***Euplectrus ronaldzunigai* Hansson, sp. n.**
–	Hind leg with 4^th^ tarsomere 0.9–1.0× as long as tarsomere 2	**68**
68(67)	Hind leg with tarsomere 1 2.6× as long as tarsomere 3; POL/OOL = 1.4	***Euplectrus charlesmicheneri* Hansson, sp. n.**
–	Hind leg with tarsomere 1 1.6× as long as tarsomere 3; POL/OOL = 1.9	***Euplectrus gavinbroadi* Hansson, sp. n. / *Euplectrus dianariasae* Hansson, sp. n.**
69(62)	Dorsellum with two large foveae anteriorly (Fig. 794)	***Euplectrus magdae* Schauff**
–	Dorsellum with foveae smaller, *or* with a groove anteriorly, *or* smooth and shiny without a groove or foveae anteriorly	**70**
70(69)	Dorsellum without a groove along anterior margin (Fig. 782), or with groove very narrow, medially 0.1–0.2× as long as length of dorsellum (Fig. 748)	**71**
–	Dorsellum with a groove or foveae along anterior margin, groove medially at least 0.3× as long as length of dorsellum (e.g. Figs 749, 754)	**74**
71(70)	Hind leg with tarsomere 4 4.6× as long as tarsomere 3	***Euplectrus daveroubiki* Hansson, sp. n.**
–	Hind leg with tarsomere 4 1.6–2.4× as long as tarsomere 3	**72**
72(71)	Scutellum with elongate and narrow meshes, mesh-rows converging towards the middle	***Euplectrus mellipes* Provancher**
–	Scutellum with isodiametric to transverse meshes, *if* meshes are elongate then with mesh-rows parallel	**73**
73(72)	Scutellum with medioposterior part with transverse meshes (Fig. 341); hind tarsus with tarsomere 2 1.9× as long as tarsomere 3	***Euplectrus roysnellingi* Hansson, sp. n.**
–	Scutellum with medioposterior part with ±isodiametric meshes (Fig. 202); hind tarsus with tarsomere 2 1.3× as long as tarsomere 3	***Euplectrus chrisdarlingi* Hansson, sp. n.**
74(70)	Hind leg with tarsomere 1 the shortest tarsomere	***Euplectrus garygibsoni* Hansson, sp. n.**
–	Hind leg with tarsomere 3 the shortest tarsomere	**75**
75(74)	Hind leg with tarsomere 4 2.0–2.5× as long as tarsomere 3	**76**
–	Hind leg with tarsomere 4 1.2–1.8× as long as tarsomere 3	**77**
76(75)	Hind leg with longest tibial spur 6.3× as long as tarsomere 3	***Euplectrus davesmithi* Hansson, sp. n.**
–	Hind leg with longest tibial spur 3.5–4.3× as long as tarsomere 3	***Euplectrus mikeschauffi* Hansson, sp. n.**
77(75)	Head with part below level of lower margin of eyes longer and more pointed (Fig. 656)	***Euplectrus testaceipes* (Cameron)**
–	Head with part below level of lower margin of eyes shorter and more blunt (e.g. Figs 234, 546)	**78**
78(77)	Hind leg with tarsomeres 1 and 4 about equally long, LT1/LT4 = 0.9–1.0	**79**
–	Hind leg with tarsomere 1 always distinctly longer than 4, LT1/LT4 = 1.3–2.1	**80**
79(78)	POL/OOL = 1.5; HE/MS = 2.2	***Euplectrus robbinthorpi* Hansson, sp. n.**
–	POL/OOL = 1.9; HE/MS = 1.8	***Euplectrus jjrodriguezae* Hansson, sp. n.**
80(78)	Hind leg with tarsomeres 2 and 4 with same length	**81**
–	Hind leg with tarsomeres 2 and 4 different in length, LT1/LT4 = 0.8 or 1.2–1.3	**84**
81(80)	POL/OOL = 2.3; OOL/DO = 1.0	***Euplectrus anae* Schauff**
–	POL/OOL = 1.8–2.0; OOL/DO = 1.2–1.3	**82**
82(81)	Petiole 1.0–1.1× as long as wide; hind leg with longest tibial spur 3.7× as long as tarsomere 3	***Euplectrus davidwahli* Hansson, sp. n.**
–	Petiole 0.8× as long as wide; hind leg with longest tibial spur 4.2–4.9× as long as tarsomere 3	**83**
83(82)	Pale area on lower face reddish-brown and smaller, does not reach outside level of lateral margins of toruli (Fig. 122)	***Euplectrus billbrowni* Hansson, sp. n.**
–	Pale area on lower face yellowish-brown and larger, reaching outside level of lateral margins of toruli (Fig. 513)	***Euplectrus paulhurdi* Hansson, sp. n.**
84(80)	Hind leg with tarsomere 4 longer than tarsomere 2, LT4/LT2 = 1.3	***Euplectrus comstockii* Howard**
–	Hind leg with tarsomere 4 shorter than tarsomere 2, LT4/LT2 = 0.8–0.9	**85**
85(84)	Petiole as long as wide; eyes smaller (Fig. 387), WE/WF = 0.3; head less wide (Fig. 387), WH/HH = 1.3	***Euplectrus johnlasallei* Hansson, sp. n.**
–	Petiole 0.8× as long as wide; eyes larger (Fig. 47), WE/WF = 0.4; head wider (Fig. 47), WH/HH = 1.4	***Euplectrus alejandrovalerioi* Hansson, sp. n.**

#### Key to males.

**Table:** 

1	Scape strongly swollen, 1.4–1.6× as long as wide (e.g. Figs 59, 263)	**2**
–	Scape at most moderately swollen, at least 2.2× as long as wide	**6**
2(1)	Scape dark brown to black (Fig. 59)	**3**
–	Scape yellow to pale brown (e.g. Figs 263, 726)	**4**
3(2)	Lower face with median part reddish-brown; hind leg with coxa and femur yellowish-brown; petiole 0.8× as long as wide	***Euplectrus insularis* (Howard)**
–	Lower face with median part black (Fig. 55); hind leg with coxa with posterior surface brown and femora infuscate; petiole 1.0× as long as wide	***Euplectrus alvarowillei* Hansson, sp. n.**
4(2)	Scape with inner lateral surface pale brown (Fig. 263)	***Euplectrus eowilsoni* Hansson, sp. n.**
–	Scape with inner lateral surface yellow to yellowish-brown (Figs 580, 726)	**5**
5(4)	Flagellomeres 1–4 yellowish-white and 5–6 pale brown (Fig. 726); mid tibia yellowish-brown	***Euplectrus valverdei* Schauff**
–	Entire flagellum yellow (Fig. 580); mid tibia with apex black	***Euplectrus ronnai* (Bréthes)**
6(1)	Lower face dark (e.g. Figs 430, 481)	**7**
–	Lower face with at least median part paler than surrounding parts of frons (e.g. Figs 472, 72)	**12**
7(6)	Hind coxa dark brown (Figs 479, 539)	**8**
–	Hind coxa yellow to yellowish-brown (e.g. Figs 87, 428)	**9**
8(7)	Hind femur yellowish-brown (Fig. 479)	***Euplectrus paulhansoni* Hansson, sp. n.**
–	Hind femur with basal ⅓ yellowish-brown and apical ⅔ dark brown (Fig. 539)	***Euplectrus scottshawi* Hansson, sp. n.**
9(7)	Mandibles dark brown	***Euplectrus lubomirmasneri* Hansson, sp. n.**
–	Mandibles white to yellowish-white	**10**
10(9)	Petiole 1.0× as long as wide	***Euplectrus andydeansi* Hansson, sp. n.**
–	Petiole 0.6–0.8× as long as wide	**11**
11(10)	Scape 4.1× as long as wide, widest in apical part (Fig. 698); pale area on gaster smaller, with posterior part of pale area 0.3× the width of gaster at that level (Fig. 696)	***Euplectrus edithae* Schauff**
–	Scape 3.1× as long as wide, widest in median part (Fig. 643); pale area on gaster larger, with posterior part of pale area 0.5× the width of gaster at that level (Fig. 640)	***Euplectrus platyhypenae* Howard**
12(6)	Hind coxa dark brown (Fig. 662) to pale brown (Fig. 70)	**13**
–	Hind coxa white to yellowish-brown (e.g. Figs 308, 470)	**14**
13(12)	Hind femur predominantly dark brown (Fig. 662)	***Euplectrus xiomarae* Schauff**
–	Hind femur yellowish-brown (Fig. 70)	***Euplectrus andybennetti* Hansson, sp. n.**
14(12)	Dorsellum anteriorly with two large foveae (Figs 760, 773, 794)	**15**
–	Dorsellum along anterior margin with a groove (e.g. Fig. 766), *or* a row of foveae (Fig. 772), *or* without a groove or foveae (e.g. Fig. 741)	**17**
15(14)	Scape with sensory area brown (Fig. 476)	***Euplectrus mikesharkeyi* Hansson, sp. n.**
–	Scape with sensory area as pale as remaining scape	**16**
16(15)	Scape with base narrow (Fig. 314); flagellomere 1 distinctly narrower at base than at apex (Fig. 314)	***Euplectrus iangauldi* Hansson, sp. n.**
–	Scape with base wide (Fig. 622); flagellomere 1 with same width at base and at apex (Fig. 622)	***Euplectrus magdae* Schauff**
17(14)	Scape with sensory pores scattered all over outer lateral surface (Fig. 382)	***Euplectrus johnlasallei* Hansson, sp. n.**
–	Scape with sensory pores confined to ventral part, or apicolateral ¾	**18**
18(17)	Lower face with pale area reaching distinctly outside of level of outer lateral margins of toruli (e.g. Figs 140, 395)	**19**
–	Lower face with pale area reaching at most to level of outer lateral margins of toruli (e.g. Figs 157, 174)	**36**
19(18)	Lower face completely pale, pale area reaching from eye to eye (e.g. Figs 140, 395)	**20**
–	Lower face with a dark area close to eyes, this area is at least as wide as ½ the diameter of one torulus (e.g. Figs 671, 711)	**23**
20(19)	Dorsellum without a groove along anterior margin (Fig. 741), hind leg with tarsomere 1 2.9× as long as tarsomere 3	***Euplectrus carlosarmientoi* Hansson, sp. n.**
–	Dorsellum with a groove along anterior margin that is 0.1–0.3× as long as length of median dorsellum, if very narrow (0.1× as long as length of median dorsellum) then hind leg with tarsomere 1 only 2.0× as long as tarsomere 3	**21**
21(20)	Pale area on lower face does not reach hypostomal carina, i.e. with parts close to mouth cavity dark (Fig. 581)	***Euplectrus johnnoyesi* Hansson, sp. n. / *Euplectrus sydneycameronae* Hansson, sp. n. (inseparable)**
–	Pale area on lower face reaches hypostomal carina, i.e. with parts close to mouth cavity pale (Fig. 582)	**22**
22(21)	Lower face with pale area close to eyes reaching distinctly above upper level of toruli (Fig. 585)	***Euplectrus floryae* Schauff**
–	Lower face with pale area close to eyes reaching to upper level of toruli (Fig. 497)	***Euplectrus paulheberti* Hansson, sp. n.**
23(19)	Petiole 0.5-0.9× as long as wide	**24**
–	Petiole 1.0–1.2× as long as wide	**33**
24(23)	Scape with sensory area as pale as remaining scape	**25**
–	Scape with sensory area darker than remaining scape (e.g. Figs 602, 715)	**28**
25(24)	Scape 4.8× as long as wide and widest medially (Fig. 675)	***Euplectrus catocalae* Howard**
–	Scape 3.2–3.6× as long as wide, slightly expanded apically, or widest medially but then only 3.2× as long as wide	**26**
26(25)	Hind leg with tarsus 5.0× as long as length of tarsomere 3 and with tarsomere 4 1.3× as long as tarsomere 3; posterior margin of petiole strongly curved forwards (Fig. 702)	***Euplectrus leucotrophis* Howard**
–	Hind leg with tarsus 5.7–7.1× as long as length of tarsomere 3 and with tarsomere 4 1.6–1.8× as long as tarsomere 3; posterior margin of petiole straight	**27**
27(26)	Petiole 0,5× as long as wide; OOL/DO = 0.8	***Euplectrus frontalis* Howard**
–	Petiole 0.7× as long as wide; OOL/DO = 1.5	***Euplectrus comstockii* Howard**
28(24)	Dorsellum with anterior groove medially 0.5–0.7× as long as length of dorsellum (e.g. Figs 792, 800)	**29**
–	Dorsellum with anterior groove covering 0.2–0.4× as long as length of dorsellum (Figs 752, 795)	**32**
29(28)	Scape narrower, LC/WS = 4.4, and with sensory area dark brown (Fig. 715)	***Euplectrus puttleri* Gordh**
–	Scape wider, LC/WS = 2.8–3.3, and with sensory area pale brown (e.g. Figs 287, 602)	**30**
30(29)	Flagellomeres more slender (Fig. 602), e.g. flagellomere 3 2.7× as long as wide	***Euplectrus ivonae* Schauff**
–	Flagellomeres more stout (Figs 287, 565), flagellomere 3 1.5–1.8× as long as wide	**31**
31(30)	Scape 2.8× as long as wide (Fig. 565)	***Euplectrus anae* Schauff**
–	Scape 3.3× as long as wide (Fig. 297)	***Euplectrus hugokonsi* Hansson, sp. n.**
32(28)	Scape with sensory area pale brown (Fig. 246)	***Euplectrus donquickei* Hansson, sp. n.**
–	Scape with sensory area dark brown (Fig. 629) (head of male *Euplectrus ronniei* not known – missing in single known specimen, but *Euplectrus ronniei* also belongs in this couplet)	***Euplectrus mariae* Schauff**
33(23)	Head with part below level of eyes and toruli narrow and strongly pointed (Fig. 646)	***Euplectrus testaceipes* (Cameron)**
–	Head with part below level of eyes and toruli wider and more blunt (e.g. Figs 361, 514)	**34**
34(33)	Scutellum with more or less isodiametric meshes (Fig. 528)	***Euplectrus paulhurdi* Hansson, sp. n.**
–	Scutellum with distinctly elongate meshes (Figs 355, 372)	**35**
35(34)	Scape widest in median part (Fig. 365); dorsellum behind anterior groove with a median carina (Fig. 765); hind leg with longest tibial spur 4.2× as long as tarsomere 3	***Euplectrus johnheratyi* Hansson, sp. n.**
–	Scape widest above the middle (Fig. 348); dorsellum behind anterior groove without median carina (Fig. 763); hind leg with longest tibial spur 3.7× as long as tarsomere 3	***Euplectrus jimwhitfieldi* Hansson, sp. n.**
36(18)	Petiole 1.1–1.3× as long as wide	**37**
–	Petiole 0.6–1.0× as long as wide	**42**
37(36)	Dorsellum without a groove along anterior margin (Figs 743, 779)	**38**
–	Dorsellum with a narrow to wide groove along anterior margin (e.g. Figs 738, 742)	**39**
38(37)	Scape wider, 2.3× as long as wide (Fig. 178)	***Euplectrus charlesmicheneri* Hansson, sp. n.**
–	Scape narrower, 2.9× as long as wide (Fig. 525)	***Euplectrus philwardi* Hansson, sp. n.**
39(37)	OOL/DO = 0.9	**40**
–	OOL/DO = 1.2–1.5	**41**
40(39)	Scape wider, 2.5× as long as wide (Fig. 161)	***Euplectrus carlrettenmeyeri* Hansson, sp. n.**
–	Scape narrower, 3.1× as long as wide (Fig. 447)	***Euplectrus mikegatesi* Hansson, sp. n.**
41(39)	Frons with median part of lower face yellowish-brown in upper ½, dark brown in lower ½ (Fig. 106); scutellum with larger meshes (Fig. 117)	***Euplectrus annettewalkerae* Hansson, sp. n.**
–	Frons with entire median part of lower face yellowish-brown (Fig. 225); scutellum with smaller meshes (Fig. 236)	***Euplectrus davidwahli* Hansson, sp. n.**
42(36)	Postmarginal vein 2.0–2.1× as long as stigmal vein	**43**
–	Postmarginal vein 1.2–1.8× as long as stigmal vein	**46**
43(42)	Hind leg with tarsomere 1 1.1× as long as tarsomere 3	***Euplectrus sondrawardae* Hansson, sp. n.**
–	Hind leg with tarsomere 1 2.0–2.7× as long as tarsomere 3	**44**
44(43)	Hind leg with tarsomere 1 2.7× as long as tarsomere 3	***Euplectrus alejandrovalerioi* Hansson, sp. n.**
–	Hind leg with tarsomere 1 2.0× as long as tarsomere 3	**45**
45(44)	Petiole 0.8× as long as wide; hind leg with tarsomere 4 2.0× as long as tarsomere 3, and 1.0× as long as tarsomere 1	***Euplectrus mikeschauffi* Hansson, sp. n.**
–	Petiole 1.0× as long as wide; hind leg with tarsomere 4 1.4× as long as tarsomere 3, and 0.7× as long as tarsomere 1	***Euplectrus davidwahli* Hansson, sp. n.**
46(42)	Scape with sensory area dark brown (Fig. 331)	***Euplectrus jacklonginoi* Hansson, sp. n.**
–	Scape with sensory area white to pale brown	**47**
47(46)	Eyes small (Figs 123, 412), WF/WE = 2.8–2.9	**48**
–	Eyes larger, WF/WE = 1.5–2.6	**49**
48(47)	Petiole 0.8× as long as wide; scape 2.8× as long as wide (Fig. 127)	***Euplectrus billbrowni* Hansson, sp. n.**
–	Petiole 1.0× as long as wide; scape 3.5× as long as wide (Fig. 416)	***Euplectrus josefernandezi* Hansson, sp. n.**
49(47)	Eyes very large (Fig. 605), WF/WE = 1.5, OOL/DO = 0.6	***Euplectrus josei* Schauff**
–	Eyes smaller, WF/WE = 2.1–2.6, OOL/DO = 1.1–1.5	**50**
50(49)	Setae on vertex dark brown (Fig. 574)	***Euplectrus victoriapookae* Hansson, sp. n.**
–	Setae on vertex transparent to whitish (e.g. Figs 208, 276)	**51**
51(50)	Scape widest below the middle and with sensory area pale brown (Fig. 212)	***Euplectrus corriemoreauae* Hansson, sp. n.**
–	Scape widest in median part or above the middle and with sensory area white to yellowish-white(Figs 195, 280)	**52**
52(51)	Scape widest above the middle (Fig. 280); OOL/DO = 1.5	***Euplectrus henrytownesi* Hansson, sp. n.**
–	Scape widest in the middle (Figs 195, 538); OOL/DO = 1.2–1.3	**53**
53(52)	Scutellum with very weak reticulation, partly smooth (Fig. 202); dorsellum with groove along anterior margin medially 0.1× as long as length of dorsellum (Fig. 745)	***Euplectrus chrisdarlingi* Hansson, sp. n.**
–	Scutellum with weak but distinct reticulation (Fig. 548); dorsellum with groove along anterior margin medially 0.3× as long as length of dorsellum (Fig. 780)	***Euplectrus robbinthorpi* Hansson, sp. n.**

### Species treatments – new species, all from ACG

#### Euplectrus alejandrovalerioi

Taxon classificationAnimaliaHymenopteraEulophidae

Hansson sp. n.

http://zoobank.org/22DAD0B0-803E-4B34-8E69-07FED8FC8F20

Figures 36–42 , 47–49 , 733

##### Material.

Holotype a female labeled “COSTA RICA: Guanacaste, ACG, Sector Mundo Nuevo, Punta Plancha, 31.vii.2013, M. Pereira, ex *Iridopsis herse* eating *Cochlospermum vitifolium*, sibling of wasp DHJPAR0053946, 13-SRNP-56118” (BMNH). Paratypes: 10♀ 1♂ with same label data as holotype (BMNH, CNC, INBio, MZLU, USNM).

##### Diagnosis.

Lower face medially dark yellowish-brown reaching to level of middle of toruli (Figs 37, 38); scutellum smooth (Fig. 49); fore and mid legs with coxae yellowish-white, femora tibiae and tarsi yellowish-brown, hind leg with coxa dark yellowish-brown, femur, tibia and tarsus yellowish-brown (Fig. 36); petiole 0.8× as long as wide; female and male gaster with anterior ½ yellowish-brown with narrow dark brown lateral margins, posterior ½ dark brown (Figs 39, 40); male scape slightly enlarged (Fig. 42), widest medially, 3.2× as long as wide.

##### Description.

*Female*. Length of body 1.7 mm. Antenna with scape yellowish-white, pedicel yellowish-brown, flagellum dark brown (Fig. 41). Mandibles and palpi yellowish-white. Head black and shiny, lower face medially dark yellowish-brown reaching to level of middle of toruli (Fig. 37). Frons close to eyes with two rows of setae (Fig. 47). Vertex smooth and shiny (Fig. 48). Occipital margin rounded (Fig. 48).

Mesosoma black and shiny (Fig. 36). Scutellum 1.0× as long as wide; smooth and shiny (Fig. 49). Dorsellum along anterior margin with a groove that is divided by longitudinal carina medially (Fig. 733), groove medially 0.3× as long as length of dorsellum. Propodeum smooth and shiny (Fig. 733); anteromedially with a short and wide semicircular cup that is strongly raised in posterior part; propodeal callus with nine setae. Legs (Fig. 36): fore and mid legs with coxae yellowish-white, femora tibiae and tarsi yellowish-brown; hind leg with coxa dark yellowish-brown, femur, tibia and tarsus yellowish-brown. Fore wing: costal cell with one row of setae on ventral surface, and margin with four setae close to marginal vein; with 14 admarginal setae.

Gaster with anterior ½ yellowish-brown with narrow dark brown lateral margins, posterior ½ dark brown (Fig. 39).

Ratios. HE/MS/WM = 2.0/1.0/1.1; POL/OOL/POO = 8.6/4.8/1.0; OOL/DO = 1.4; WE/WF/WH/HH = 1.0/2.6/4.8/3.3; WH/WT = 1.2; PM/ST = 1.6; TS1/TS2/LT/LT1/LT2/LT3/LT4 = 4.2/2.4/7.2/3.0/1.6/1.0/1.4; LP/WP = 0.8; MM/LG = 1.4.

*Male*. Length of body 1.6 mm. Scape slightly enlarged, widest medially (Fig. 42), with sensory pores limited to apical ⅔ of ventral margin, sensory area yellowish-white. Similar to female except shorter gaster.

Ratios. LC/WS = 3.2; MM/LG = 1.7.

##### Hosts and biology.

Feeding on last instar larva of *Iridopsis herse* (Geometridae) feeding on *Cochlospermum vitifolium* (Bixaceae), parasitoid cocoons under larval mummy of host.

##### Distribution.

Costa Rica (Guanacaste Province).

##### Etymology.

This species is named after Alejandro A. Valerio, in recognition of his contribution to the understanding of ACG Hymenoptera taxonomy.

#### Euplectrus alexsmithi

Taxon classificationAnimaliaHymenopteraEulophidae

Hansson sp. n.

http://zoobank.org/9F8DCA96-E920-4E14-9842-47EF11542438

Figures 43–46 , 50–52 , 734

##### Material.

Holotype a female labeled “COSTA RICA: Guanacaste, ACG, Sector Pitilla, Pasmompa, 12.i.2006, P. Rios, ex *Eustrotia* Poole14 eating *Brachiaria arrecta*, sibling of wasp DHJPAR0028872, 06-SRNP-30437” (BMNH). PARATYPES: 8♀ with same label data as holotype (BMNH, CNC, INBio, MZLU, USNM).

##### Diagnosis.

Antenna long and slender (Fig. 46), e.g. flagellum 3.0× as long as height of an eye; lower face black with median part dark reddish-brown, pale area reaching to outer lateral margin of toruli (Fig. 44); legs yellowish-brown with fore and mid coxae yellowish-white (Fig. 43); petiole 1.0× as long as wide; gaster yellowish-brown with dark brown lateral margins and apex, and with a dark brown spot medially on tergite 4 (Fig. 45).

##### Description.

*Female*. Length of body 2.6 mm. Antenna long and slender, with scape yellowish-white in basal ⅔, yellowish-brown in apical ⅓, pedicel yellowish-brown, flagellomeres 1–3 yellowish-brown ventrally and dark brown dorsally, 4–6 dark brown (Fig. 46). Mandibles and palpi yellowish-white. Head black and shiny, lower face medially dark reddish-brown, pale area reaching to outer lateral margin of toruli (Fig. 44). Frons close to eyes with one row of setae with upper part of row removed away from eye (Fig. 50). Vertex smooth (Fig. 51). Occipital margin with a carina behind ocellar triangle (Fig. 51).

Mesosoma black and shiny (Fig. 43). Each sidelobe of mesoscutum with 11 setae. Scutellum 0.9× as long as wide; with very weak engraved reticulation (Fig. 52). Dorsellum along anterior margin with a groove that is divided by longitudinal carinae (Fig. 734), groove medially 0.3× as long as length of dorsellum. Propodeum with very weak reticulation (Fig. 734); anteromedially with a short and wide triangular cup; propodeal callus with 10 setae. Legs yellowish-brown with fore and mid coxae yellowish-white (Fig. 43). Fore wing: costal cell with two rows of setae on ventral surface, and margin with six setae close to marginal vein; with ten admarginal setae in one row.

Gaster yellowish-brown with sides and apex dark brown, and with a dark brown spot medially on tergite 4 (Fig. 45).

Ratios. HE/MS/WM = 1.8/1.0/1.2; POL/OOL/POO = 4.7/2.8/1.0; OOL/DO = 1.5; WE/WF/WH/HH = 1.0/2.7/4.7/3.5; WH/WT = 1.0; PM/ST = 1.5; TS1/TS2/LT/LT1/LT2/LT3/LT4 = 4.2/2.6/7.4/2.5/1.5/1.0/2.3; LP/WP = 1.0; MM/LG = 1.0.

*Male*. Unknown.

##### Hosts and biology.

Feeding on last instar larva of *Eustrotia* Poole14 (Noctuidae) feeding on *Brachiaria arrecta* (Poaceae), parasitoid cocoons stuck to dead larva and substrate.

##### Distribution.

Costa Rica (Guanacaste Province).

##### Etymology.

This species is named after M. Alex Smith, in recognition of his contribution to the understanding of ACG Hymenoptera taxonomy.

#### Euplectrus alvarowillei

Taxon classificationAnimaliaHymenopteraEulophidae

Hansson sp. n.

http://zoobank.org/60F76AF8-7CFF-44A0-877F-2A546D2FBB91

Figures 53–59 , 64–66 , 735

##### Material.

Holotype a female labeled “COSTA RICA: Guanacaste, ACG, Sector Potrerillos, Rio Azufrado, 18.v.2006, L. Swiacki, ex *Callopistria floridensis* eating Ceratopteris Espinoza 4311, sibling of wasp DHJPAR0028881, 06-SRNP-13561” (BMNH). Paratypes: 13♀ 2♂: COSTA RICA (ACG): **Guanacaste:** 5♀ 1♂ with same label data as holotype (BMNH, INBio); Sector Potrerillos: **Rio Azufrado:** ex *Callopistria floridensis* eating *Ceratopteris Espinoza* 4311 18.v.2006, L. Swiacki, sibling of wasp DHJPAR00288954311, 06-SRNP-13538, (6♀, in BMNH, CNC, MZLU, USNM); Sector Pitilla: **Bullas**, 12.ii.2010, D. Martinez, ex *Callopistria floridensis* eating *Niphidium oblanceolatum*, sibling of wasp DHJPAR00392, 10-SRNP-70752 (2♀ 1♂, in INBio).

##### Diagnosis.

Lower face black (Figs 54, 55); legs yellowish-brown with mid and hind coxae and hind femur brown (Fig. 53); petiole 1.0× as long as wide; gaster with anterior ½ yellowish-brown (female, Fig. 56) or white (male, Fig. 57), with lateral margins black, posterior ½ black; male antenna with scape dark brown to black, strongly swollen (Fig. 59), 1.6× as long as wide, with numerous sensory pores scattered all over the inner lateral surface. Very similar to *Euplectrus insularis* but with lower face black (reddish-brown in *Euplectrus insularis*), male petiole as long as wide (0.8× as long as wide in *Euplectrus insularis*), and hind legs with coxae and femora brown (yellowish-brown in *Euplectrus insularis*).

##### Description.

*Female*. Length of body 2.2 mm. Antenna with scape and pedicel yellowish-brown, flagellomeres 1–2 yellowish-brown with dorsal surface dark brown, flagellomeres 3–6 dark brown (Fig. 58). Mandibles and palpi yellowish-brown. Head including lower face black and shiny (Fig. 54). Frons close to eyes with four irregular rows of setae (Fig. 64). Vertex smooth and shiny (Fig. 65). Occipital margin with a carina behind ocellar triangle (Fig. 65).

Mesosoma black and shiny (Fig. 53). Scutellum 0.8× as long as wide; with very weak reticulation (Fig. 66). Dorsellum along anterior margin with a deep groove that is divided by longitudinal carinae (Fig. 735), groove medially 0.4× as long as length of dorsellum. Propodeum smooth and shiny (Fig. 735); anteromedially with a semicircular cup; propodeal callus with 11 setae. Legs (Fig. 53): fore leg yellowish-brown; mid leg with coxa brown, femur, tibia and tarsus yellowish-brown; hind leg with coxa and femur brown, tibia and tarsus yellowish-brown. Fore wing: costal cell with one complete row of setae on ventral surface, and margin with six setae close to marginal vein; with 14 admarginal setae in one row.

Gaster in anterior ½ yellowish-brown with lateral margins black, posterior ½ black (Fig. 56).

Ratios. HE/MS/WM = 1.7/1.0/1.0; POL/OOL/POO = 8.3/4.0/1.0; OOL/DO = 1.8; WE/WF/WH/HH = 1.0/2.8/4.9/3.7; WH/WT = 1.1; PM/ST = 1.3; TS1/TS2/LT/LT1/LT2/LT3/LT4 = 5.0/3.0/8.3/3.3/1.8/1.0/1.6; LP/WP = 1.0; MM/LG = 1.0.

*Male*. Length of body 1.6 mm. Scape dark brown to black, strongly swollen (Fig. 59); with entire inner lateral surface with sensory pores. Similar to female except antenna with pedicel and flagellomeres 1–2 yellowish-white, flagellomeres 3–6 dark brown to black, 3–4 with peduncles yellowish-white (Fig. 59), gaster with anterior pale area white (Fig. 57).

Ratios. LC/WS = 1.6; MM/LG = 1.0.

##### Hosts and biology.

Feeding on third instar larva of *Callopistria floridensis* (Noctuidae) feeding on *Ceratopteris Espinoza* 4311 (Parkeriaceae) and *Niphidium oblanceolatum* (Polypodiaceae), parasitoid cocoons stuck to dead larva and substrate.

##### Distribution.

Costa Rica (Guanacaste Province).

##### Etymology.

This species is named after Alvaro Wille, in recognition of his contribution to the understanding of ACG Hymenoptera taxonomy.

#### Euplectrus andybennetti

Taxon classificationAnimaliaHymenopteraEulophidae

Hansson sp. n.

http://zoobank.org/5C1B891A-00DB-42A2-B20B-FB94C6304A71

Figures 77–80 , 81–83 , 427 , 736

##### Material.

Holotype a female labeled “COSTA RICA: Guanacaste, ACG, Sector Santa Rosa, Bosque Humedo, 11.vii.1994, gusaneros, ex *Elymiotis attenuata* eating *Mascagnia sinemariensis*, no barcode, 94-SRNP-5588” (BMNH). PARATYPES: 48♀ 12♂: **Guanacaste:** 31♀ 2♂ with same label data as holotype (BMNH, CNC, INBio, MZLU, USNM); following from same locality and host as holotype but collected 9.viii.1992, no barcode, 92-SRNP-4426 (7♀, in INBio, MIUCR), 16.vii.1993, host feeding on Hiraea reclinata, no barcode, 93-SRNP-3731 (1♀ 2♂, in INBio), 11.vii.1994, no barcode, 94-SRNP-5595 and 94-SRNP-5588 (10♀ 8♂, in BMNH, CNC, MIUCR, USNM).

##### Diagnosis.

Lower face medially reddish-brown (female, Fig. 71) or yellowish-brown (male, Fig. 72), pale area reaching slightly outside level of lateral margin of toruli, parts betwen pale area and eyes black; vertex with parts lateral to ocellar triangle dark reddish-brown; fore and mid legs yellowish-white, hind leg with coxa dark yellowish-brown, femur yellowish-brown, tibia and tarsus yellowish-white (Fig. 70); petiole 0.7× as long as wide; gaster with anterior ½ yellowish-brown with dark brown lateral margins (female, Fig. 73) or white with dark brown lateral margins (male, Fig. 74), posterior ½ dark brown; male antenna with scape slightly expanded and widest medially, 3.3× as long as wide (Fig. 76), sensory area dark brown.

##### Description.

*Female*. Length of body 2.6 mm. Antenna with scape yellowish-white in basal ½ and yellowish-brown in apical ½, pedicel yellowish-brown, flagellomeres pale brown (Fig. 75). Mandibles yellowish-white, palpi white. Head black and shiny, vertex with parts lateral to ocellar triangle dark reddish-brown, lower face medially reddish-brown, pale area reaching slightly outside level of lateral margin of toruli, parts between pale area and eyes black (Fig. 71). Frons close to eyes with two rows of setae (Fig. 81). Vertex with very weak reticulation inside ocellar triangle, smooth outside triangle (Fig. 82). Occipital margin with a weak carina behind ocellar triangle (Fig. 82).

Mesosoma black and shiny (Fig. 70). Each sidelobe of mesoscutum with 14 setae. Scutellum 0.8× as long as wide; with weak engraved reticulation (Fig. 83). Dorsellum anteriorly with a wide groove that is divided by longitudinal carinae (Fig. 736), groove medially 0.5× as long as length of dorsellum. Propodeum with very weak reticulation (Fig. 736); anteromedially with a transverse semicircular cup; propodeal callus with five setae. Legs with fore and mid legs yellowish-white, hind leg with coxa dark yellowish-brown, femur yellowish-brown, tibia and tarsus yellowish-white (Fig. 70). Fore wing: costal cell with 1 row of setae on ventral surface, and margin with four setae close to marginal vein; with 13 admarginal setae, in one row.

Gaster with anterior ½ yellowish-brown with dark brown lateral margins, posterior ½ dark brown (Fig. 73).

Ratios. HE/MS/WM = 1.5/1.0/1.0; POL/OOL/POO = 10.0/5.0/1.0; OOL/DO = 1.2; WE/WF/WH/HH = 1.0/2.6/4.6/3.3; WH/WT = 1.0; PM/ST = 1.6; TS1/TS2/LT/LT1/LT2/LT3/LT4 = 4.8/2.8/7.6/2.8/1.8/1.0/1.8; LP/WP = 0.7; MM/LG = 1.0.

*Male*. Length of body 1.8 mm. Scape slightly expanded and widest medially, sensory pores confined to anteroventral ¾ (Fig. 427), sensory area dark brown (Fig. 76). Otherwise similar to female except lower face with pale part yellowish-brown (Fig. 72), gaster with anterior ½ white with dark brown lateral margins, and shorter (Fig. 74).

Ratios. LC/WS = 3.3; MM/LG = 1.2.

##### Hosts and biology.

Feeding on penultimate instar karva of *Elymiotis attenuata* (Notodontidae) feeding on *Hiraea reclinata* and *Mascagnia sinemariensis* (Malpighiaceae), parasitoid cocoons stuck to dead larva and substrate.

##### Distribution.

Costa Rica (Guanacaste Province).

##### Etymology.

This species is named after Andy Bennett, in recognition of his contribution to the understanding of ACG Hymenoptera taxonomy.

#### Euplectrus andydeansi

Taxon classificationAnimaliaHymenopteraEulophidae

Hansson sp. n.

http://zoobank.org/E46A857C-8864-4D53-B9D7-2F3A3276412C

Figures 87–93 , 98–100 , 737

##### Material.

Holotype a female labeled “COSTA RICA: Guanacaste, ACG, Sector Pitilla, Ingas, 28.xi.2011, F. Quesada, ex *Eustrotia* Poole04 feeding on *Scleria melaleuca*, sibling of wasp DHJPAR0046907, 11-SRNP-33575” (BMNH). PARATYPES: 4♀ 1♂ with same label data as holotype (BMNH, INBio).

##### Diagnosis.

Lower face completely black (Fig. 88, 89); legs yellowish-brown (Fig. 87); petiole 1.0× as long as wide; gaster in female dark brown with a narrow yellowish-brown longitudinal stripe in anteromedian ½ (Fig. 90), in male with anterior ½ white with dark brown lateral margins and posterior ½ dark brown (Fig. 91); male antenna with scape slightly expanded and widest in the middle and with apex narrow, 3.1× as long as wide, sensory area brown (Fig. 93).

##### Description.

*Female*. Length of body 2.0 mm. Antenna with scape and pedicel yellowish-brown, flagellomeres dark brown (Fig. 92). Mandibles and palpi white. Head including lower face black and shiny (Fig. 88). Frons close to eyes with two rows of setae (Fig. 98). Vertex smooth (Fig. 99). Occipital margin rounded (Fig. 99).

Mesosoma black and shiny (Fig. 87). Each sidelobe of mesoscutum with nine setae. Scutellum 1.0× as long as wide; with very weak engraved reticulation, posterior margin smooth (Fig. 100). Dorsellum anteriorly with a groove that is divided by longitudinal carinae (Fig. 737), groove medially 0.4× as long as length of dorsellum. Propodeum with very weak reticulation (Fig. 737); anteromedially with a triangular cup; propodeal callus with seven setae. Legs yellowish-brown (Fig. 87). Fore wing: costal cell with one row of setae on ventral surface, and margin with four setae close to marginal vein; with 13 admarginal setae, in one row.

Gaster dark brown with a narrow yellowish-brown longitudinal stripe in anteromedian ½ (Fig. 90).

Ratios. HE/MS/WM = 2.2/1.0/1.3; POL/OOL/POO = 3.3/2.2/1.0; OOL/DO = 1.5; WE/WF/WH/HH = 1.0/2.6/4.6/3.1; WH/WT = 1.1; PM/ST = 1.5; TS1/TS2/LT/LT1/LT2/LT3/LT4 = 4.0/2.8/7.3/2.6/1.6/1.0/2.0; LP/WP = 1.0; MM/LG = 1.1.

*Male*. Length of body 1.5 mm. Scape slightly expanded and widest in the middle and with apex narrow (Fig. 93), sensory pores confined to ventral margin and with sensory area brown. Otherwise similar to female except gaster with anterior ½ white with dark brown lateral margins (Fig. 91), and shorter.

Ratios. LC/WS = 3.1; MM/LG = 1.2.

##### Hosts and biology.

Feeding on third instar larva of *Eustrotia* Poole04 (Noctuidae) feeding on *Scleria melaleuca* (Cyperaceae), parasitoid cocoons stuck to dead larva and substrate.

##### Distribution.

Costa Rica (Guanacaste Province).

##### Etymology.

This species is named after Andy R. Deans, in recognition of his contribution to the understanding of ACG Hymenoptera taxonomy.

#### Euplectrus annettewalkerae

Taxon classificationAnimaliaHymenopteraEulophidae

Hansson sp. n.

http://zoobank.org/8EE48F40-3D32-48B4-9DB0-AE55C7BDEF5C

Figures 104–110 , 115–117 , 738

##### Material.

Holotype a female labeled “COSTA RICA: Guanacaste, ACG, Sector Pitilla, Medrano, 19.i.2012, R. Calero, ex *Tyrissa* Poole01 eating *Senegalia tenuifolia*, sibling of wasp DHJPAR0046917, 12-SRNP-70154” (BMNH). PARATYPE: 1♂ with same label data as holotype (BMNH).

##### Diagnosis.

Lower face with median part yellowish-brown in upper ½, dark brown in lower ½, pale area reaching to outer lateral margin of toruli (Figs 105, 106); legs yellowish-brown (Fig. 104); petiole 1.1× as long as wide; gaster dark brown, anterior ½ with a dusky pale spot medially (Figs 107, 108); male antenna with scape expanded and widest medially, 2.8× as long as wide (Fig. 110).

##### Description.

*Female*. Length of body 1.7 mm. Antenna with scape yellowish-white in basal ½ and yellowish-brown in apical ½, pedicel and flagellomere 1 yellowish-brown, 2–6 dark brown (Fig. 109). Mandibles and palpi white. Head black and shiny, lower face with median part yellowish-brown in upper ½, dark brown in lower ½, pale area reaching to outer lateral margin of toruli, parts between pale area and eyes black (Fig. 105). Frons close to eyes with two rows of setae (Fig. 115). Vertex smooth (Fig. 116). Occipital margin rounded (Fig. 116).

Mesosoma black and shiny (Fig. 104). Each sidelobe of mesoscutum with 16 setae. Scutellum 0.9× as long as wide; with weak engraved reticulation, posterior margin smooth (Fig. 117). Dorsellum anteriorly with a narrow groove that is divided by longitudinal carinae (Fig. 738), groove medially 0.2× as long as length of dorsellum. Propodeum smooth (Fig. 738); anteromedially with a semicircular cup; propodeal callus with six setae. Legs yellowish-brown (Fig. 104). Fore wing: costal cell on ventral surface with one row of setae, and margin with two setae close to marginal vein; with 12 admarginal setae, in one row.

Gaster dark brown, anterior ½ with a dusky pale spot medially (Fig. 107).

Ratios. HE/MS/WM = 1.8/1.0/1.0; POL/OOL/POO = 5.3/3.4/1.0; OOL/DO = 1.3; WE/WF/WH/HH = 1.0/2.3/4.2/3.0; WH/WT = 1.1; PM/ST = 1.5; TS1/TS2/LT/LT1/LT2/LT3/LT4 = 3.8/2.4/6.2/2.0/1.4/1.0/1.6; LP/WP = 1.1; MM/LG = 1.4.

*Male*. Length of body 1.4 mm. Scape expanded and widest medially (Fig. 110), sensory pores confined to ventral margin; sensory area pale as scape. Otherwise similar to female except shorter gaster.

Ratios. LC/WS = 2.8; MM/LG = 1.5.

##### Hosts and biology.

Feeding on penultimate instar larva of *Tyrissa* Poole01 (Erebidae) feeding on *Senegalia tenuifolia* (Fabaceae), parasitoid cocoons stuck to host larva cuticle and leaf.

##### Distribution.

Costa Rica (Guanacaste Province).

##### Etymology.

This species is named after Annette K. Walker, in recognition of her contribution to the understanding of ACG Hymenoptera taxonomy.

#### Euplectrus billbrowni

Taxon classificationAnimaliaHymenopteraEulophidae

Hansson sp. n.

http://zoobank.org/3A632EE4-0D95-4BD2-B6AD-B3FF2F7E89B3

Figures 121–131 , 27 , 132–134 , 739

##### Material.

Holotype a female labeled “COSTA RICA: Alajuela, ACG, Rio Blanco Abajo, 23.vi.2005, E. Araya, ex *Dyops* chromatophilaDHJ01 eating *Coussapoa nymphaeifolia*, sibling of wasp DHJPAR0028838, 05-SRNP-3556” (BMNH). Paratypes: 44♀ 21♂ with same label data as holotype (BMNH, CNC, INBio, MZLU, MIUCR, USNM).

##### Diagnosis.

Lower face medially reddish-brown (female, Fig. 122) or yellowish-white (male, Fig. 123), pale area reaching to outer lateral margin of toruli; scutellum with sides and posterior margin smooth (Fig. 134); female legs yellowish-brown (Fig. 121), male with fore and mid legs predominantly yellowish-white and hind leg yellowish-brown; male antenna with scape slightly expanded, widest in the middle, and 2.8× as long as wide (Fig. 127); petiole 0.8× as long as wide; gaster with anterior ½ yellowish-white with dark brown lateral margins, posterior ½ dark brown (Figs 124, 125).

##### Description.

*Female*. Length of body 2.2 mm. Antenna with scape and pedicel yellowish-brown, flagellomeres 1–2 dark brown dorsally and yellowish-brown ventrally, 3–6 dark brown (Fig. 126). Mandibles yellowish-brown with base dark brown, palpi white. Head black and shiny, lower face medially reddish-brown, pale area reaching to outer lateral margin of toruli, and with parts lateral to pale area black (Fig. 122). Frons close to eyes with three rows of setae (Fig. 132). Vertex with very weak reticulation (Fig. 133). Occipital margin with a carina behind ocellar triangle (Fig. 133).

Mesosoma black and shiny (Fig. 121). Scutellum 1.0× as long as wide; with rather weak reticulation and with sides and posterior part smooth (Fig. 134). Dorsellum along anterior margin with a groove that is divided by longitudinal carinae (Fig. 739), groove medially 0.3× as long as length of dorsellum. Propodeum with very weak reticulation (Fig. 739); anteromedially with a semicircular cup; propodeal callus with ten setae. Legs yellowish-brown (Fig. 121). Fore wing: costal cell on ventral surface with two rows of setae in basal ½ and one row in apical ½, and margin with one seta close to marginal vein; with 13 admarginal setae in one row.

Gaster with anterior ½ yellowish-white with dark brown lateral margins, posterior ½ dark brown (Fig. 124).

Ratios. HE/MS/WM = 1.9/1.0/1.1; POL/OOL/POO = 8.0/4.1/1.0; OOL/DO = 1.2; WE/WF/WH/HH = 1.0/2.9/5.0/3.7; WH/WT = 1.1; PM/ST = 1.6; TS1/TS2/LT/LT1/LT2/LT3/LT4 = 4.9/3.6/7.1/2.7/1.7/1.0/1.6; LP/WP = 0.8; MM/LG = 1.1.

*Male*. Length of body 1.8 mm. Scape white, somewhat expanded and widest in the middle (Fig. 127); sensory pores confined to apicoventral ⅔, sensory area white. Similar to female except pedicel and flagellomeres 1–2 yellowish-white and 3–6 brown dorsally and yellowish-brown ventrally (Fig. 127), lower face medially yellowish-white (Fig. 123); fore and mid legs predominantly yellowish-white, gaster shorter.

Ratios. LC/WS = 2.8; MM/LG = 1.3.

##### Hosts and biology.

Feeding on last instar larva of *Dyops* chromatophilaDHJ01 (Erebidae) feeding on *Coussapoa nymphaeifolia* (Urticaceae), parasitoid cocoons stuck to dead larva and substrate.

##### Distribution.

Costa Rica (Alajuela Province).

##### Etymology.

This species is named after Bill L. Brown, in recognition of his contribution to the understanding of ACG Hymenoptera taxonomy.

#### Euplectrus bobwhartoni

Taxon classificationAnimaliaHymenopteraEulophidae

Hansson sp. n.

http://zoobank.org/CC481A0C-0F29-4AFD-97DF-CC2D588015A5

Figures 60–63 , 67–69 , 740

##### Material.

Holotype a female labeled “COSTA RICA: Guanacaste, ACG, Sector Murcielago, Camino Bahia Hachal, 13.vi.2006, G. Pereira, ex *Bagisara tristicta* eating *Herissantia crispa*, sibling of wasp DHJPAR0028861, 06-SRNP-17307” (BMNH). PARATYPES: 8♀: 1♀ with same label data as holotype (BMNH, INBio); **Guanacaste:** Parque Nacional Palo Verde, 50 m, LN 260952/385020, 5-16.vi.1999, malaise trap, I. Jiménez (6♀, CNC, INBio, USNM); **Puntarenas:** Peninsula Osa, Estación Agujas, 250-350 m, LS 276750/526550, 4-20.vi.1999, swept, J. Azofeifa (1♀, INBio).

##### Diagnosis.

Lower face medially yellowish-white with median part yellowish-brown, pale area reaching slightly outside outer lateral margin of toruli (Fig. 61); scutellum smooth medially and with very weak engraved reticulation laterally (Fig. 69); legs yellowish-brown (Fig. 60); petiole 0.5× as long as wide; gaster yellowish-brown with dark brown lateral margins (Fig. 62).

##### Description.

*Female*. Length of body 2.6 mm. Antenna with scape yellowish-white in basal ½ and yellowish-brown in apical ½, pedicel yellowish-brown, flagellomeres 1–4 yellowish-brown ventrally and dark brown dorsally, 5–6 dark brown (Fig. 58). Mandibles and palpi white. Head black and shiny, lower face medially yellowish-white with median part yellowish-brown, pale area reaching slightly outside outer lateral margin of toruli, with parts between pale area and eyes black (Fig. 61). Frons close to eyes with two rows of setae (Fig. 67). Vertex smooth (Fig. 68). Occipital margin rounded (Fig. 68).

Mesosoma black and shiny (Fig. 60). Each sidelobe of mesoscutum with 13 setae. Scutellum 1.0× as long as wide; smooth medially and with very weak engraved reticulation laterally (Fig. 69). Dorsellum along anterior margin with a groove that is very narrow laterally and wide medially, medially 0.4× as long as length of dorsellum, groove is divided by longitudinal carinae (Fig. 740). Propodeum partly with very weak reticulation and partly smooth (Fig. 740); anteromedially with a short and wide triangular cup; propodeal callus with 12 setae. Legs yellowish-brown (Fig. 60). Fore wing: costal cell with two rows of setae on ventral surface, and margin with two setae close to marginal vein; with 24 admarginal setae, in two rows.

Gaster yellowish-brown with dark brown lateral margins (Fig. 62).

Ratios. HE/MS/WM = 2.1/1.0/1.3; POL/OOL/POO = 6.7/3.8/1.0; OOL/DO = 1.4; WE/WF/WH/HH = 1.0/2.6/4.6/3.5; WH/WT = 1.1; PM/ST = 1.2; TS1/TS2/LT/LT1/LT2/LT3/LT4 = 3.2/1.7/6.0/1.8/1.3/1.0/1.7; LP/WP = 0.5; MM/LG = 1.0.

*Male*. Unknown.

##### Hosts and biology.

Feeding on penultimate instar larva of *Bagisara tristicta* (Noctuidae) feeding on *Herissantia crispa* (Malvaceae), parasitoid cocoons stuck to dead larva and substrate.

##### Distribution.

Costa Rica (Guanacaste and Puntarenas Provinces).

##### Etymology.

This species is named after Bob A. Wharton, in recognition of his contribution to the understanding of ACG Hymenoptera taxonomy.

#### Euplectrus carlosarmientoi

Taxon classificationAnimaliaHymenopteraEulophidae

Hansson sp. n.

http://zoobank.org/92833AB7-6C57-4294-94AC-CBDAF6CC0167

Figures 138–144 , 149–151 , 741

##### Material.

Holotype a female labeled “COSTA RICA: Guanacaste, ACG, Sector Cacao, 18.xii.2007, D. Garcia, ex *Oxidia apidania* eating *Inga punctata*, sibling of wasp DHJPAR0023273, 07-SRNP-47481” (BMNH). PARATYPES: 4♀ 3♂ with same label data as holotype (BMNH, INBio).

##### Diagnosis.

Entire lower face yellowish-white with median part yellowish-brown (female, Fig. 139) or completely white (male, Fig. 140), parts close to eyes extending above level of upper margin of toruli; dorsellum without groove or foveae along anterior margin (Fig. 741); fore and mid legs yellowish-white, hind leg yellowish-brown (Fig. 138); petiole 1.0× as long as wide; gaster with anterior ½ white with wide dark brown lateral margins, posterior ½ dark brown (Figs 141, 142); male antenna with scape slightly expanded and widest in the middle, 2.8× as long as wide (Fig. 144), flagellomeres 2–5 with a basal whorl of erect setae.

##### Description.

*Female*. Length of body 2.3 mm. Antenna with scape yellowish-white, pedicel yellowish-brown, flagellomeres pale brown (Fig. 143). Mandibles and palpi yellowish-white. Head black and shiny, entire lower face yellowish-white with median part yellowish-brown, parts close to eyes extending above level of upper margin of toruli (Fig. 139); vertex with parts lateral to ocellar triangle dark yellowish-brown. Frons close to eyes with two rows of setae (Fig. 149). Vertex smooth (Fig. 150). Occipital margin with a weak carina behind ocellar triangle (Fig. 150).

Mesosoma black and shiny (Fig. 138). Each sidelobe of mesoscutum with 14 setae. Scutellum 1.0× as long as wide; with strong engraved reticulation (Fig. 151). Dorsellum without groove along anterior margin (Fig. 741). Propodeum smooth (Fig. 741); anteromedially with a semicircular cup; propodeal callus with seven setae. Fore and mid legs yellowish-white, hind leg yellowish-brown (Fig. 138). Fore wing: costal cell with two rows of setae on ventral surface, and margin with four setae close to marginal vein; with 15 admarginal setae, in one row.

Gaster with anterior ½ white with wide dark brown lateral margins, posterior ½ dark brown (Fig. 141).

Ratios. HE/MS/WM = 2.3/1.0/1.8; POL/OOL/POO = 4.5/2.3/1.0; OOL/DO = 1.4; WE/WF/WH/HH = 1.0/2.3/4.4/3.1; WH/WT = 1.1; PM/ST = 1.6; TS1/TS2/LT/LT1/LT2/LT3/LT4 = 4.7/2.7/7.1/2.9/1.6/1.0/1.6; LP/WP = 1.0; MM/LG = 1.1.

*Male*. Length of body 1.9 mm. Scape slightly expanded and widest in the middle (Fig. 144), sensory pores confined to ventral margin. Otherwise similar to female except flagellomeres 2–5 with a basal whorl of erect setae (Fig. 144), lower face completely white (Fig. 140); scutellum with very weak reticulation in posterior ¾; gaster shorter.

Ratios. LC/WS = 2.8; MM/LG = 1.2.

##### Hosts and biology.

Feeding on penultimate instar larva of *Oxidia apidania* (Geometridae) feeding on *Inga punctata* (Fabaceae), parasitoid cocoons stuck to dead larva and substrate.

##### Distribution.

Costa Rica (Guanacaste Province).

##### Etymology.

This species is named after Carlos E. Sarmiento, in recognition of his contribution to the understanding of ACG Hymenoptera taxonomy.

#### Euplectrus carlrettenmeyeri

Taxon classificationAnimaliaHymenopteraEulophidae

Hansson sp. n.

http://zoobank.org/02FA8070-DF34-4A16-AAF2-36230B8999C0

Figures 155–161 , 166–168 , 742

##### Material.

Holotype a female labeled “COSTA RICA: Alajuela, ACG, Sector Rincon Rain Forest, Sendero Tucan, 25.xi.2011, P. Umana, ex *Antiblemma ceras* eating *Conostegia xalapensis*, sibling of wasp DHJPAR0046912, 11-SRNP-44933” (BMNH). PARATYPES: 8♀ 1♂ with same label data as holotype (BMNH, CNC, INBio, MZLU, USNM).

##### Diagnosis.

Lower face with median part yellowish-brown, pale area reaching to level of outer lateral margins of toruli (Figs 156, 157); antennal flagellum long, e.g. 1^st^ flagellomere 3.0× as long as wide (Figs 160, 161); submarginal vein with four setae; legs yellowish-white with fore and mid coxae white (Fig. 155); petiole 1.3× as long as wide; gaster in female with anterior ½ white with dark brown lateral margins, followed by a wide dark brown transverse band, and with apex yellowish-brown (Fig. 158), in male with anterior ½ white with dark brown lateral margins and posterior ½ dark brown (Fig. 159); male antenna with scape slightly expanded and widest in the middle, 2.5× as long as wide (Fig. 161).

##### Description.

*Female*. Length of body 2.2 mm. Antenna with scape yellowish-white, pedicel yellowish-brown, flagellomeres 1–2 pale brown, 3–6 dark brown (Fig. 160); flagellum long, e.g. 1^st^ flagellomere 3.0× as long as wide. Mandibles and palpi white. Head black and shiny, lower face with median part yellowish-brown, pale area reaching to level of outer lateral margins of toruli, with parts between pale area and eyes black (Fig. 156). Frons close to eyes with two rows of setae (Fig. 166). Vertex with very weak reticulation inside ocellar triangle, smooth outside (Fig. 167). Occipital margin rounded (Fig. 167).

Mesosoma black and shiny (Fig. 155). Each sidelobe of mesoscutum with 13 setae. Scutellum 1.0× as long as wide; with very weak engraved reticulation, posterior margin smooth (Fig. 168). Dorsellum anteriorly with a very narrow groove that is divided by longitudinal carinae (Fig. 742), groove medially 0.1× as long as length of dorsellum. Propodeum with very weak reticulation (Fig. 742); anteromedially with a transverse semicircular cup; propodeal callus with eight setae. Legs yellowish-white with fore and mid coxae white (Fig. 155). Fore wing: submarginal vein with four setae; costal cell on ventral surface with one row of setae, and margin with five setae close to marginal vein; with 20 admarginal setae, partly in two rows.

Gaster with anterior ½ white with dark brown lateral margins, followed by a wide dark brown transverse band, and with apex yellowish-brown (Fig. 158).

Ratios. HE/MS/WM = 2.3/1.0/1.3; POL/OOL/POO = 4.7/1.8/1.0; OOL/DO = 0.9; WE/WF/WH/HH = 1.0/2.0/4.2/3.0; WH/WT = 1.1; PM/ST = 1.5; TS1/TS2/LT/LT1/LT2/LT3/LT4 = 3.4/2.2/5.7/1.3/1.3/1.0/1.7; LP/WP = 1.3; MM/LG = 1.2.

*Male*. Length of body 2.1 mm. Scape slightly expanded and widest in the middle (Figs 161), sensory pores confined to apicoventral ¾. Otherwise similar to female except gaster with posterior ½ dark brown (Fig. 159) and shorter.

Ratios. LC/WS = 2.5; MM/LG = 1.4.

##### Hosts and biology.

Feeding on intermediate instar larva of *Antiblemma ceras* (Erebidae) feeding on *Conostegia xalapensis* (Melastomataceae), parasitoid cocoons stuck to dead larva and substrate.

##### Distribution.

Costa Rica (Alajuela Province).

##### Etymology.

This species is named after Carl W. Rettenmeyer, in recognition of his contribution to the understanding of ACG Hymenoptera taxonomy.

#### Euplectrus charlesmicheneri

Taxon classificationAnimaliaHymenopteraEulophidae

Hansson sp. n.

http://zoobank.org/5B279C51-68FE-48F4-B794-B33852AC9B7B

Figures 16 , 172–178 , 183–185 , 743

##### Material.

Holotype a female labeled “COSTA RICA: Guanacaste, ACG, Sector Cacao, Sendero Nayo, 22.vi.2008, M. Pereira, ex *Sicya medangula* eating *Crossopetalum parviflorum*, sibling of wasp DHJPAR0031152, 08-SRNP-35772” (BMNH). PARATYPES: 11♀ 1♂: COSTA RICA: **Guanacaste:** 6♀ 1♂ with same label data as holotype (BMNH, INBio, MZLU); Sector Cacao, Estación Cacao, 25.vi.2008, H. Ramirez, ex *Prochoerodes marciana* eating *Prunus annularis*, sibling of wasp DHJPAR0031155, 08-SRNP-35835 (3♀, in CNC, USNM); Estación Pitilla, LN 330200/380200, 700 m, vi.1996, C. Moraga & P. Rios (2♀, in INBio).

##### Diagnosis.

Lower face with median part yellowish-brown (female, Fig. 173) or yellowish-white (male, Fig. 174) reaching to level of outer margin of toruli; dorsellum without groove or foveae along anterior margin (Fig. 743); female with legs yellowish-brown with fore coxa yellowish-white (Fig. 172), male legs white with tarsi, apical ⅓ of hind femur and hind tibia yellowish-brown; petiole 1.3× as long as wide; gaster dark brown, anterior ½ white with dark brown lateral margins (Figs 175, 176); male antenna with scape expanded and widest in the middle, 2.3× as long as wide (Fig. 178).

##### Description.

*Female*. Length of body 2.2 mm. Antenna with scape yellowish-white, pedicel yellowish-brown, flagellomeres 1–2 pale brown, 3–6 dark brown (Fig. 177). Mandibles and palpi yellowish-white. Head black and shiny, lower face with median part yellowish-brown reaching to level of outer margin of toruli and parts between pale area and eyes black (Fig. 173). Frons close to eyes with two rows of setae (Fig. 183). Vertex smooth (Fig. 184). Occipital margin with a weak carina (Fig. 184).

Mesosoma black and shiny (Fig. 172). Each sidelobe of mesoscutum with 11 setae. Scutellum 0.9× as long as wide; with weak engraved reticulation (Fig. 185). Dorsellum without groove or foveae along anterior margin (Fig. 743). Propodeum smooth (Fig. 743); anteromedially with a semicircular cup; propodeal callus with nine setae. Legs yellowish-brown with fore coxa yellowish-white (Fig. 172). Fore wing: costal cell with two rows of setae on ventral surface, and margin with seven setae close to marginal vein; with 17 admarginal setae, in one row.

Gaster dark brown, anterior ½ white with dark brown lateral margins (Fig. 175).

Ratios. HE/MS/WM = 2.3/1.0/1.2; POL/OOL/POO = 7.0/5.0/1.0; OOL/DO = 1.4; WE/WF/WH/HH = 1.0/2.5/4.6/3.2; WH/WT = 1.1; PM/ST = 1.8; TS1/TS2/LT/LT1/LT2/LT3/LT4 = 4.0/2.2/6.7/2.6/1.4/1.0/1.4; LP/WP = 1.3; MM/LG = 1.2.

*Male*. Length of body 1.6 mm. Scape white, expanded and widest in the middle (Fig. 178), sensory pores confined to apicoventral ¾, sensory area pale as scape. Otherwise similar to female except pale area on lower face yellowish-white (Fig. 174); legs white with tarsi, apical ⅓ of hind femur and entire hind tibia yellowish-brown.

Ratios. LC/WS = 2.3; MM/LG = 1.2.

##### Hosts and biology.

Feeding on intermediate instar larvae of *Prochoerodes marciana* feeding on *Prunus annularis* (Rosaceae), and *Sicya medangula* feeding on *Crossopetalum parviflorum* (Celastraceae) (both hosts are Geometridae), parasitoid cocoons stuck to dead larva and substrate.

##### Distribution.

Costa Rica (Guanacaste Province).

##### Etymology.

This species is named after Charles D. Michener, in recognition of his contribution to the understanding of ACG Hymenoptera taxonomy.

#### Euplectrus charlesporteri

Taxon classificationAnimaliaHymenopteraEulophidae

Hansson sp. n.

http://zoobank.org/D80823B2-02FC-4A0D-92B6-F3F7ECE279F9

Figures 77–80 , 84–86 , 744

##### Material.

Holotype a female labeled “COSTA RICA: Guanacaste, ACG, Sector Horizones, 14.x.1997, gusaneros, ex *Diastema tigris* eating *Lantana camara*, no barcode, 97-SRNP-9717” (BMNH). PARATYPES: 3♀ with same label data as holotype (BMNH, INBio).

##### Diagnosis.

Lower face yellowish-white with median part yellowish-brown, leaving a black area close to eye as wide as shortest diameter of torulus (Fig. 78); vertex dark reddish-brown; legs yellowish-brown, fore and mid coxae yellowish-white (Fig. 77); petiole 1.0× as long as wide; gaster with anterior ½ yellowish-white and posterior ½ yellowish-brown, with dark brown lateral margins throughout (Fig. 79).

##### Description.

*Female*. Length of body 2.5 mm. Antenna with scape white with apical ½ yellowish-brown, pedicel yellowish-brown, flagellomeres pale brown (Fig. 80). Mandibles and palpi white. Head black and shiny, lower face yellowish-white with median part yellowish-brown, leaving a black area close to eye as wide as shortest diameter of torulus (Fig. 78); vertex dark reddish-brown outside ocellar triangle, black inside ocellar triangle. Frons close to eyes with two rows of setae (Fig. 84). Vertex smooth outside ocellar triangle, with very weak reticulation inside ocellar triangle (Fig. 82). Occipital margin with a carina (Fig. 82).

Mesosoma black and shiny (Fig. 77). Each sidelobe of mesoscutum with 15 setae. Scutellum 1.0× as long as wide; with rather strong engraved reticulation with elongate meshes (Fig. 86). Dorsellum anteriorly with a wide groove that is divided by longitudinal carinae (Fig. 744), groove medially 0.5× as long as length of dorsellum. Propodeum smooth (Fig. 744); anteromedially with a semicircular cup; propodeal callus with 11 setae. Legs yellowish-brown, fore and mid coxae yellowish-white (Fig. 77). Fore wing: costal cell on ventral surface with two rows of setae in basal ½ and one row in distal ½, and margin with three setae close to marginal vein; with 15 admarginal setae, in one row.

Gaster with anterior ½ yellowish-white and posterior ½ yellowish-brown, with dark brown lateral margins throughout (Fig. 79).

Ratios. HE/MS/WM = 2.0/1.0/1.5; POL/OOL/POO = 4.8/2.8/1.0; OOL/DO = 1.6; WE/WF/WH/HH = 1.0/2.4/4.5/3.3; WH/WT = 1.1; PM/ST = 1.6; TS1/TS2/LT/LT1/LT2/LT3/LT4 = 3.4/2.0/6.4/2.3/1.4/1.0/1.1; LP/WP = 1.0; MM/LG = 1.1.

*Male*. Unknown.

##### Hosts and biology.

Feeding on last instar larva of *Diastema tigris* (Noctuidae) feeding on *Lantana camara* (Verbenaceae), parasitoid cocoons stuck to dead larva and substrate.

##### Distribution.

Costa Rica (Guanacaste Province).

##### Etymology.

This species is named after Charles C. Porter, in recognition of his contribution to the understanding of ACG Hymenoptera taxonomy.

#### Euplectrus chrisdarlingi

Taxon classificationAnimaliaHymenopteraEulophidae

Hansson sp. n.

http://zoobank.org/337C0824-9AEA-4528-AC5C-6FF0917AAFC4

Figures 189–195 , 200–202 , 745

##### Material.

Holotype a female labeled “COSTA RICA: Guanacaste, ACG, Sector Pitilla, Pasmompa, 26.xii.2007, C. Moraga & M. Rios, ex *Letis mycerina* eating *Inga oerstediana*, sibling of wasp DHJPAR0028698, 07-SRNP-34320” (BMNH). PARATYPES: 9♀ 1♂: **Guanacaste:** 6♀ 1♂ with same label data as holotype (BMNH, INBio, MZLU); 3♀ with same locality and host data but collected 8.xii.2007, sibling of wasp DHJPAR0028700, 07-SRNP-34119 (CNC, USNM).

##### Diagnosis.

Lower face with median part dark reddish-brown with two small yellowish-brown spots, pale area not reaching outside of outer lateral margins of toruli (Figs 190, 191); dorsellum anteriorly with a very narrow groove that is divided by longitudinal carinae (Fig. 745); legs yellowish-brown with fore and mid coxae yellowish-white (Fig. 189); gaster with anterior ½ white with dark brown lateral margins, posterior ½ dark brown (Figs 192, 193); petiole 0.8× as long as wide in female, 1.0× in male; male antenna with scape slightly expanded and widest in the middle, 2.9× as long as wide (Fig. 195).

##### Description.

*Female*. Length of body 2.0 mm. Antenna with scape yellowish-brown with base yellowish-white, pedicel yellowish-brown, flagellomere 1 pale brown, 2–6 dark brown (Fig. 194). Mandibles and palpi yellowish-white. Head black and shiny, lower face with median part dark reddish-brown with two small yellowish-brown spots, pale area not reaching outside of outer lateral margins of toruli, and with parts between pale area and eyes black (Fig. 190). Frons close to eyes with one row of setae (Fig. 200). Vertex smooth (Fig. 201). Occipital margin with a carina (Fig. 201).

Mesosoma black and shiny (Fig. 189). Each sidelobe of mesoscutum with nine setae. Scutellum 0.9× as long as wide; with very weak engraved reticulation (Fig. 202). Dorsellum anteriorly with a narrow groove that is divided by longitudinal carinae (Fig. 745), groove medially 0.1× as long as length of dorsellum. Propodeum with very weak reticulation (Fig. 745); anteromedially with a transverse triangular cup; propodeal callus with 13 setae. Legs yellowish-brown with fore and mid coxae yellowish-white (Fig. 189). Fore wing: costal cell with one row of setae on ventral surface, and margin with two setae close to marginal vein; with 13 admarginal setae, in one row.

Gaster with anterior ½ white with dark brown lateral margins, posterior ½ dark brown (Fig. 192).

Ratios. HE/MS/WM = 2.4/1.0/1.1; POL/OOL/POO = 8.0/4.6/1.0; OOL/DO = 1.3; WE/WF/WH/HH = 1.0/2.4/4.4/3.3; WH/WT = 1.1; PM/ST = 1.6; TS1/TS2/LT/LT1/LT2/LT3/LT4 = 3.6/2.2/5.6/1.7/1.3/1.0/1.6; LP/WP = 0.8; MM/LG = 1.2.

*Male*. Length of body 1.5 mm. Scape slightly expanded and widest in the middle (Fig. 195), sensory pores confined to apicoventral ¾, sensory area pale as scape. Otherwise similar to female except longer petiole.

Ratios. LC/WS = 2.9; LP/WP = 1.0: MM/LG = 1.2.

##### Hosts and biology.

Feeding on penultimate instar larva of *Letis mycerina* (Erebidae) feeding on *Inga oerstediana* (Fabaceae), parasitoid cocoons stuck to dead larva and substrate.

##### Distribution.

Costa Rica (Guanacaste Province).

##### Etymology.

This species is named after D. Chris Darling, in recognition of his contribution to the understanding of ACG Hymenoptera taxonomy.

#### Euplectrus chrisgrinteri

Taxon classificationAnimaliaHymenopteraEulophidae

Hansson sp. n.

http://zoobank.org/B3572DF2-2FF3-405D-A39D-0FAD15AC8E48

Figures 94–97 , 101–103 , 746

##### Material.

Holotype a female labeled “COSTA RICA: Guanacaste, ACG, Sector Santa Rosa, 7.v.1995, gusaneros, ex *Eulepidotis caeruleilinea* eating *Hymenaea courbaril*, no barcode, 95-SRNP-3415” (BMNH).

##### Diagnosis.

Lower face medially yellowish-white with median ⅓ pale yellowish-brown, pale area reaching outside level of outer lateral margin of toruli, leaving a wide black stripe between pale area and eye (Fig. 95); fore and mid legs yellowish-white, hind leg yellowish-brown (Fig. 94); petiole 0.9× as long as wide; gaster with anterior ½ yellowish-brown with dark brown anterolateral margins and with posterior part of margins yellowish-brown, posterior ½ dark brown (Fig. 96). *Euplectrus chrisgrinteri* is very similar to *Euplectrus ronniei*, the only differences are the longer petiole in *Euplectrus chrisgrinteri* and different host preferences.

##### Description.

*Female*. Length of body 2.1 mm. Antenna with scape yellowish-white, pedicel and flagellomeres yellowish-brown (Fig. 97). Mandibles and palpi white. Head black and shiny, lower face medially yellowish-white with median ⅓ pale yellowish-brown, pale area reaching outside of level of outer lateral margins of toruli, leaving a wide black stripe between pale area and eyes (Fig. 95). Frons close to eyes with one row of setae (Fig. 101). Vertex smooth (Fig. 102). Occipital margin with a carina (Fig. 102).

Mesosoma black and shiny (Fig. 94). Each sidelobe of mesoscutum with 10 setae. Scutellum 1.0× as long as wide; with weak engraved reticulation (Fig. 103). Dorsellum anteriorly with a narrow groove that is divided by longitudinal carinae (Fig. 746), groove medially 0.3× as long as length of dorsellum. Propodeum with weak reticulation (Fig. 746); anteromedially with a semicircular cup; propodeal callus with eight setae. Fore and mid legs yellowish-white, hind leg yellowish-brown (Fig. 94). Fore wing: costal cell on ventral surface with one row of setae, partly with two rows close to base, and margin with three setae close to marginal vein; with 14 admarginal setae, in one row.

Gaster with anterior ½ yellowish-brown with dark brown anterolateral margins and with posterior part of margins yellowish-brown, posterior ½ dark brown (Fig. 96).

Ratios. HE/MS/WM = nm; POL/OOL/POO = 5.0/2.2/1.0; OOL/DO = 1.2; WE/WF/WH/HH = 1.0/2.8/4.9/3.9; WH/WT = 1.0; PM/ST = 1.2; TS1/TS2/LT/LT1/LT2/LT3/LT4 = 4.2/2.4/7.6/2.4/1.4/1.0/2.0; LP/WP = 0.9; MM/LG = 1.0.

*Male*. Unknown.

##### Hosts and biology.

Feeding on last instar larva of *Eulepidotis caeruleilinea* (Erebidae) feeding on *Hymenaea courbaril* (Fabaceae), parasitoid cocoons stuck to dead larva and substrate.

##### Distribution.

Costa Rica (Guanacaste Province).

##### Etymology.

This species is named after Chris C. Grinter, in recognition of his contribution to the understanding of ACG Hymenoptera taxonomy.

#### Euplectrus corriemoreauae

Taxon classificationAnimaliaHymenopteraEulophidae

Hansson sp. n.

http://zoobank.org/0EDF7C69-E7FC-4E60-BF10-D375E33EFE1F

Figures 206–212 , 217–219 , 747

##### Material.

Holotype a female labeled “COSTA RICA: Alajuela, ACG, Sector Rincón Rain Forest, Palomo, 18.vii.2013, K. Aragón, ex same as Noctuidae 11-SRNP-33495 eating *Rhipidocladum racemiflorum*, sibling of wasp DHJPAR0053131, 13-SRNP-68860” (BMNH). Paratypes: 4♀ 1♂ with same label data as holotype (BMNH, CNC, INBio, USNM).

##### Diagnosis.

Lower face with median part dark yellowish-brown reaching to level of middle of toruli (Figs 207, 208); legs yellowish-brown (Fig. 206); petiole 0.9× as long as wide; female gaster yellowish-white in anterior ½, yellowish-brown in posterior ½, lateral margins dark brown (Fig. 209); male scape slightly enlarged (Fig. 212), widest medially, 3.0× as long as wide, sensory area brown and reaching along ventral margin.

##### Description.

*Female*. Length of body 1.9 mm. Antenna with scape yellowish-white with apical ⅓yellowish-brown, pedicel yellowish-brown, flagellum dark brown with ventral part pale brown (Fig. 211). Mandibles and palpi yellowish-white. Head black and shiny, lower face with median part dark yellowish-brown reaching to level of middle of toruli (Fig. 207). Frons close to eyes with one row of setae (Fig. 217). Vertex smooth and shiny (Fig. 218). Occipital margin with a carina behind ocellar triangle (Fig. 218).

Mesosoma black and shiny (Fig. 206). Scutellum 1.0× as long as wide; with very weak reticulation (Fig. 219). Dorsellum along anterior margin with a deep groove (Fig. 747), groove medially 0.4× as long as length of dorsellum. Propodeum smooth and shiny (Fig. 747); anteromedially with a semicircular cup that is strongly raised in posterior part; propodeal callus with eight setae. Legs yellowish-brown (Fig. 206). Fore wing: costal cell with two rows of setae on ventral surface, and margin with two setae close to marginal vein; with 13 admarginal setae.

Gaster yellowish-white in anterior ½, yellowish-brown in posterior ½, lateral margins dark brown (Fig. 209).

Ratios. HE/MS/WM = 2.0/1.0/1.2; POL/OOL/POO = 4.5/2.3/1.0; OOL/DO = 1.4; WE/WF/WH/HH = 1.0/3.0/5.2/3.8; WH/WT = 1.1; PM/ST = 1.3; TS1/TS2/LT/LT1/LT2/LT3/LT4 = 3.7/2.0/6.3/2.2/1.3/1.0/1.8; LP/WP = 0.9–1.0; MM/LG = 1.0.

*Male*. Length of body 1.7 mm. Scape slightly enlarged, widest medially, with sensory pores along entire ventral margin, sensory area brown (Fig. 212). Similar to female except gaster with anterior ½ white with dark brown lateral margins, posterior ½ dark brown (Fig. 210).

Ratios. LC/WS = 3.0, LP/WP = 1.0.

##### Hosts and biology.

Feeding on penultimate instar larva of noctuid 11-SRNP-33495 (Noctuidae) feeding on *Rhipidocladum racemiflorum* (Poaceae), parasitoid cocoons stuck to dead larva and substrate.

##### Distribution.

Costa Rica (Alajuela Province).

##### Etymology.

This species is named after Corrie S. Moreau, in recognition of her contribution to the understanding of ACG Hymenoptera taxonomy.

#### Euplectrus daveroubiki

Taxon classificationAnimaliaHymenopteraEulophidae

Hansson sp. n.

http://zoobank.org/7751A6F2-067B-4DC7-9F67-D596633C47F4

Figures 111–114 , 118–120 , 748

##### Material.

Holotype a female labeled “COSTA RICA: Guanacaste, ACG, Sector Del Oro, Rio Chon, 6.i.2008, R. Moraga, ex *Hemiceras vecina* eating *Inga oerstediana*, sibling of wasp DHJPAR0023280, 08-SRNP-20135” (BMNH). PARATYPES: 8♀: COSTA RICA (ACG): **Guanacaste:** 3♀ with same label data as holotype (BMNH, INBio); 4♀ with same data as holotype but sibling of wasp DHJPAR0023278, 08-SRNP-20132 (CNC, MZLU, USNM); Sector Brasilia, Piedrona, 24.vi.2008, D. Briceno, ex *Hemiceras nigrescens* eating *Inga oerstediana*, sibling of wasp DHJPAR0031185, 08-SRNP-65614 (1♀, in BMNH).

##### Diagnosis.

Lower face with median part yellowish-brown, pale area reaching to outer lateral margins of toruli (Fig. 112); fore and mid coxa yellowish-white, remaining parts of fore and mid legs and entire hind leg yellowish-brown (Fig. 111); scutellum 0.9× as long as wide with rather strong reticulation (Fig. 120); dorsellum with a very narrow groove along anterior margin (Fig. 748); median carina on propodeum narrow (Fig. 748); petiole 1.0× as long as wide; gaster dark brown, anterior ½ with a large yellowish-brown ±T-shaped spot (Fig. 113), and with tergites 5–6 reddish-brown posteromedially.

##### Description.

*Female*. Length of body 2.5 mm. Antenna with scape yellowish-white in basal ½ and yellowish-brown in apical ½, pedicel yellowish-brown, flagellomere 1 dark brown with ventral part yellowish-brown, 2–6 dark brown (Fig. 114). Mandibles and palpi white. Head black and shiny, lower face with median part yellowish-brown, pale area reaching to outer lateral margins of toruli, with parts between pale area and eyes black (Fig. 112). Frons close to eyes with one row of setae (Fig. 118). Vertex with very weak reticulation (Fig. 119). Occipital margin with a weak carina (Fig. 119).

Mesosoma black and shiny (Fig. 111). Each sidelobe of mesoscutum with 12 setae. Scutellum 0.9× as long as wide; with rather strong reticulation, meshes slightly elongate (Fig. 120). Dorsellum with a very narrow groove along anterior margin (Fig. 748), medially less than 0.1× as long as length of dorsellum. Propodeum smooth (Fig. 748); anteromedially with a short triangular cup, 0.3× as long as wide; propodeal callus with nine setae. Legs (Fig. 111): fore and mid coxae yellowish-white, remaining parts of fore and mid legs and entire hind leg yellowish-brown. Fore wing: costal cell with one row of setae on ventral surface, and margin with one seta close to marginal vein; with 15 admarginal setae, in one row.

Gaster dark brown, anterior ½ with a large yellowish-brown ±T-shaped spot (Fig. 113), and with tergites 5–6 reddish-brown posteromedially.

Ratios. HE/MS/WM = 2.3/1.0/1.3; POL/OOL/POO = 6.8/4.2/1.0; OOL/DO = 1.2; WE/WF/WH/HH = 1.0/2.5/4.5/3.3; WH/WT = 1.0; PM/ST = 2.3; TS1/TS2/LT/LT1/LT2/LT3/LT4 = 3.7/2.3/6.6/2.3/1.7/1.0/4.6; LP/WP = 1.0; MM/LG = 1.3.

*Male*. Unknown.

##### Hosts and biology.

Feeding on third instar larvae of *Hemiceras vecina* and *Hemiceras nigrescens* (Notodontidae) feeding on *Inga oerstediana* (Fabaceae), parasitoid cocoons stuck to dead larva and substrate.

##### Distribution.

Costa Rica (Guanacaste Province).

##### Etymology.

This species is named after Dave W. Roubik, in recognition of his contribution to the understanding of ACG Hymenoptera taxonomy.

##### Remarks.

In the analyzed material there are three females, not included here, from same locality and host as holotype but collected 23.xii.2007 (sibling of wasp DHJPAR0038555, 07-SRNP-24811). These specimens appear similar to *Euplectrus daveroubiki*, but the barcode is different from other samples of *Euplectrus daveroubiki* (Suppl. material 1, sample called E. Hansson32). All three specimens are damaged and because of this the morphological analysis can not be done with sufficient accuracy. Therefore we leave these specimens untreated for now, but in the future, as more material becomes available, this puzzle will be dealt with.

Another puzzle associated with the samples assigned to this species concern samples DHJPAR0031185 and DHJPAR0023280. Specimens from DHJPAR0031185 and DHJPAR0023280 are morphologically identical and have a similar biology (both target *Hemiceras* spp.), but with different barcodes (Suppl. material 1). Since the morphology and biology is the same we prefer to treat these as the same species.

See also remarks under *Euplectrus roysnellingi*.

#### Euplectrus davesmithi

Taxon classificationAnimaliaHymenopteraEulophidae

Hansson sp. n.

http://zoobank.org/B14167FC-E550-4E6C-A034-854C96D2696D

Figures 128–131 , 135–137 , 749

##### Material.

Holotype a female labeled “COSTA RICA: Guanacaste, ACG, Sector Pitilla, Pasmompa, 24.ix.2005, gusaneros, ex *Ceromacra* Poole01 eating *Guatteria diospyroides*, sibling of wasp DHJPAR0028811, 05-SRNP-34118” (BMNH). PARATYPE: 1♀ with same label data as holotype (INBio).

##### Diagnosis.

Lower face medially yellowish-brown, pale area reaching to outer lateral margins of toruli (Fig. 129); scutellum transverse, 0.8× as long as wide, with very weak engraved reticulation, partly smooth (Fig. 137); dorsellum without spines posteromedially (Fig. 749); legs yellowish-white (Fig. 128); propodeum anteromedially with triangular cup short and wide (Fig. 749); petiole 1.0× as long as wide; gaster dark brown, anterior ½ with a large yellowish-white spot (Fig. 130).

##### Description.

*Female*. Length of body 2.0 mm. Antenna with scape and pedicel yellowish-brown, flagellomere 1 pale brown, 2–6 dark brown (Fig. 131). Mandibles and palpi yellowish-white. Head black and shiny, lower face medially yellowish-brown, pale area reaching to outer lateral margins of toruli, with parts between pale area and eyes black (Fig. 129), vertex dark brown. Frons close to eyes with one sparse row of setae (Fig. 135). Vertex smooth (Fig. 136). Occipital margin with a weak carina behind ocellar triangle (Fig. 136).

Mesosoma black and shiny (Fig. 128). Each sidelobe of mesoscutum with seven setae. Scutellum 0.8× as long as wide; with very weak engraved reticulation, partly smooth (Fig. 137). Dorsellum along anterior margin with a groove that is divided by longitudinal carinae (Fig. 749), groove medially 0.2× as long as length of dorsellum, median posterior margin of dorsellum without spines. Propodeum smooth (Fig. 749); anteromedially with a short and wide semicircular cup; propodeal callus with eight setae. Legs yellowish-white (Fig. 128). Fore wing: costal cell with two rows of setae on ventral surface, and margin with five setae close to marginal vein; with 25 admarginal setae, in two irregular rows.

Gaster dark brown, anterior ½ with a large yellowish-white spot (Fig. 130).

Ratios. HE/MS/WM = 1.8/1.0/1.1; POL/OOL/POO = 3.7/1.8/1.0; OOL/DO = 1.3; WE/WF/WH/HH = 1.0/2.6/4.4/3.3; WH/WT = 1.1; PM/ST = 2.0; TS1/TS2/LT/LT1/LT2/LT3/LT4 = 6.3/2.8/7.7/2.3/1.7/1.0/2.0; LP/WP = 1.0; MM/LG = 0.9.

*Male*. Unknown.

##### Hosts and biology.

Feeding on second instar larva of *Ceromacra* Poole02 (Erebidae) feeding on *Guatteria diospyroides* (Annonaceae), parasitoid cocoons stuck to leaf substrate.

##### Distribution.

Costa Rica (Guanacaste Province).

##### Etymology.

This species is named after Dave R. Smith, in recognition of his contribution to the understanding of ACG Hymenoptera taxonomy.

##### Remarks.

**Figure 35.**
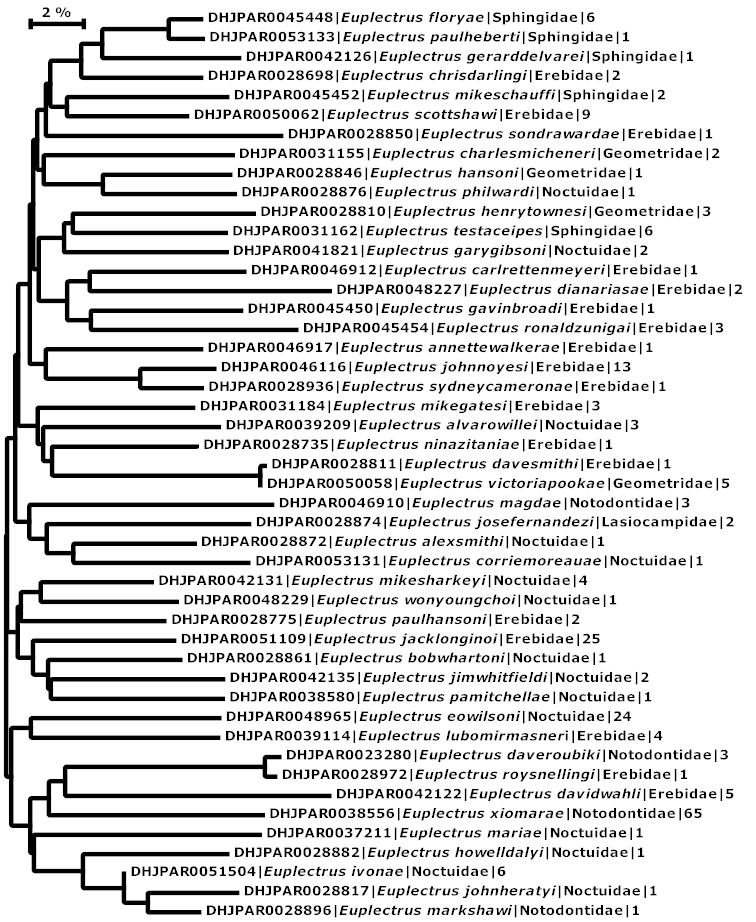
Interspecific variation of DNA barcodes among species of ACG *Euplectrus* displayed using the Neighbor-Joining method (Saitou and Nei 1987) with distances computed using the Kimura 2-parameter method (Kimura 1980) conducted in MEGA6 (Tamura et al 2013). When there was more than one sequence available for each species, the representative sequence was selected based on quality (longest read length with the fewest ambiguities). Line labels are the voucher code|species name|host family|number of barcoded specimens in the ACG database at the time of writing. In the two cases where two species have nearly identical barcodes, morphological differences suggest that each pair indeed contains two species, the veracity of which will be the subject of further research when more samples are available.

**Figures 36–46.**
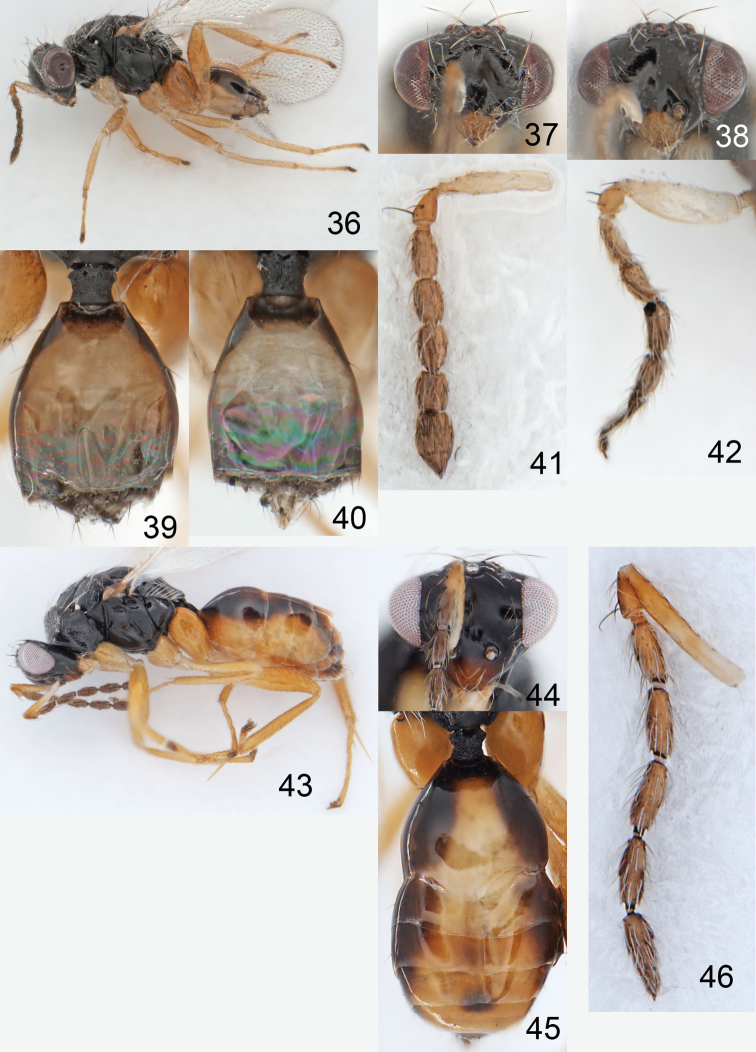
*Euplectrus* spp. **36–42** *Euplectrus alejandrovalerioi* (DHJPAR0053946): **36** habitus in lateral view, female **37** head in frontal view, female **38** head in frontal view, male **39** gaster in dorsal view, female **40** gaster in dorsal view, male **41** antenna in lateral view, female **42** antenna in lateral view, male; **43–46** *Euplectrus alexsmithi* (DHJPAR0028872), female: **43** habitus in lateral view **44** head in frontal view **45** gaster in dorsal view **46** antenna in lateral view.

**Figures 47–52.**
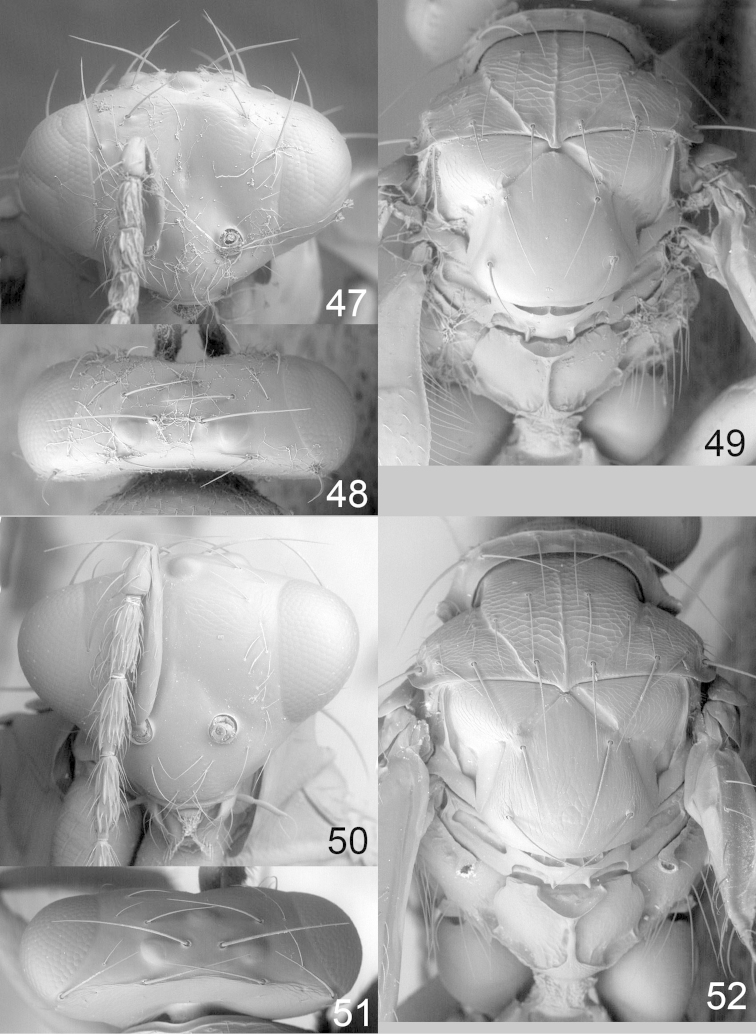
*Euplectrus* spp., females. **47–49** *Euplectrus alejandrovalerioi* (DHJPAR0053946): **47** head in frontal view **48** vertex **49** mesosoma in dorsal view **50–52** *Euplectrus alexsmithi* (DHJPAR0028872): **50** head in frontal view **51** vertex **52** mesosoma in dorsal view.

**Figures 53–63.**
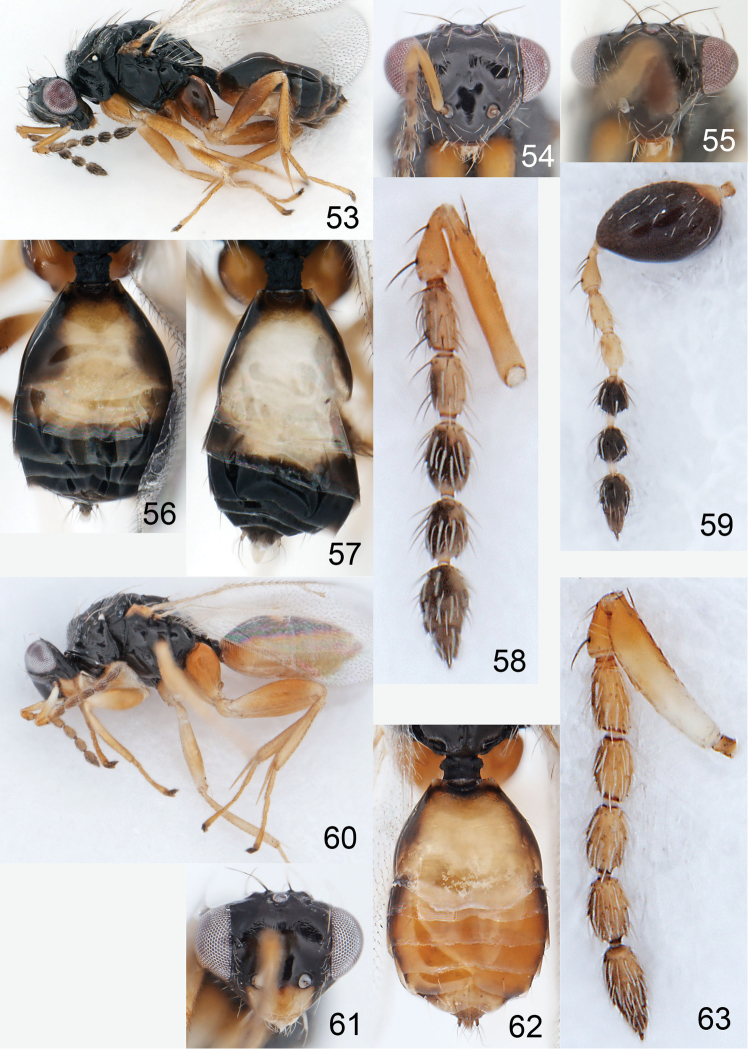
*Euplectrus* spp. **53–59** *Euplectrus alvarowillei* (DHJPAR0028881): **53** habitus in lateral view, female **54** head in frontal view, female **55** head in frontal view, male **56** gaster in dorsal view, female **57** gaster in dorsal view, male **58** antenna in lateral view, female **59** antenna in lateral view, male **60–63** *Euplectrus bobwhartoni* (DHJPAR0028861), female: **60** habitus in lateral view **61** head in frontal view **62** gaster in dorsal view **63** antenna in lateral view.

**Figures 64–69.**
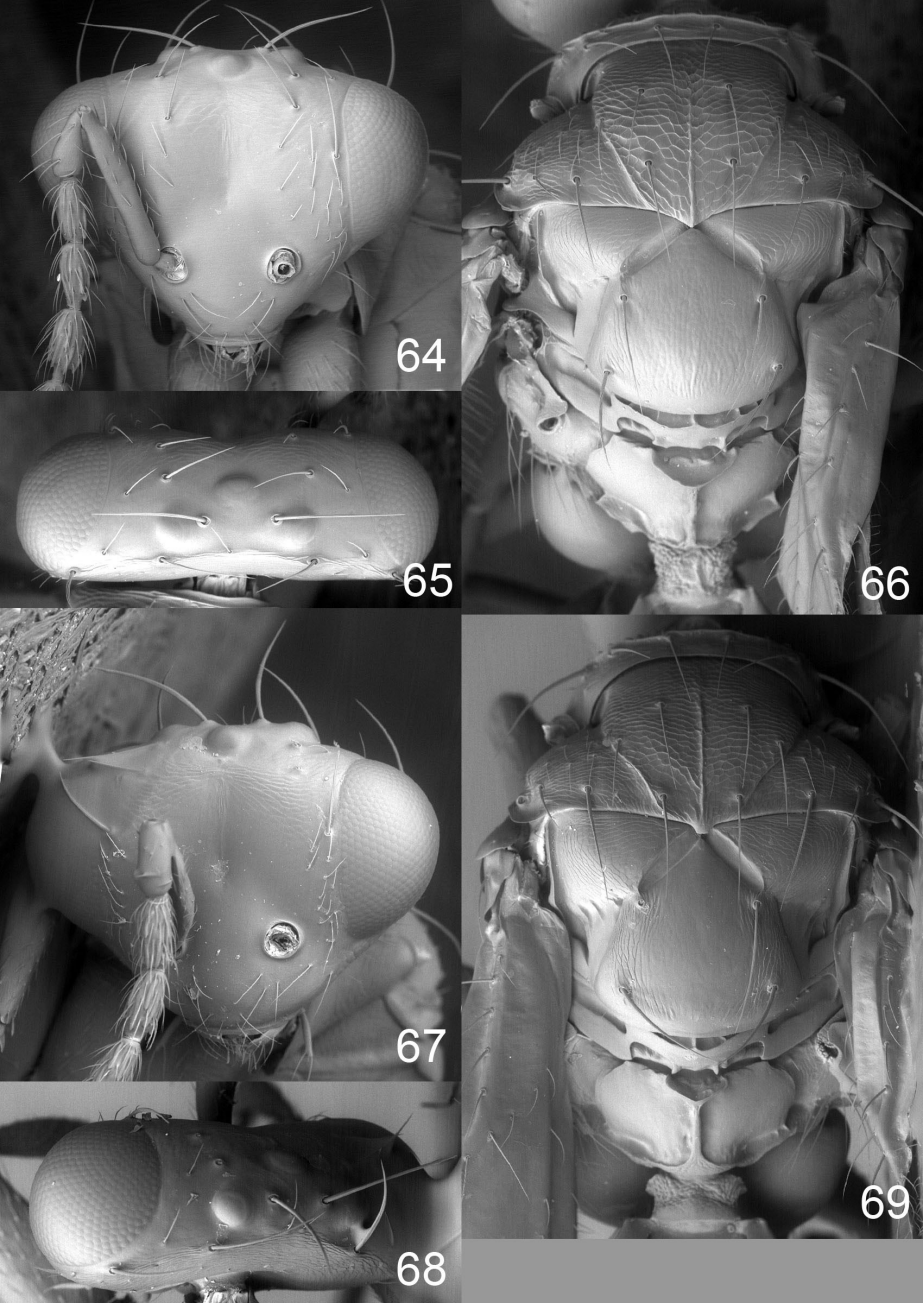
*Euplectrus* spp., females. **64–66** *Euplectrus alvarowillei* (DHJPAR0028881): **64** head in frontal view **65** vertex **66** mesosoma in dorsal view **67–69** *Euplectrus bobwhartoni* (DHJPAR0028861): **67** head in frontal view **68** vertex **69** mesosoma in dorsal view.

**Figures 70–80.**
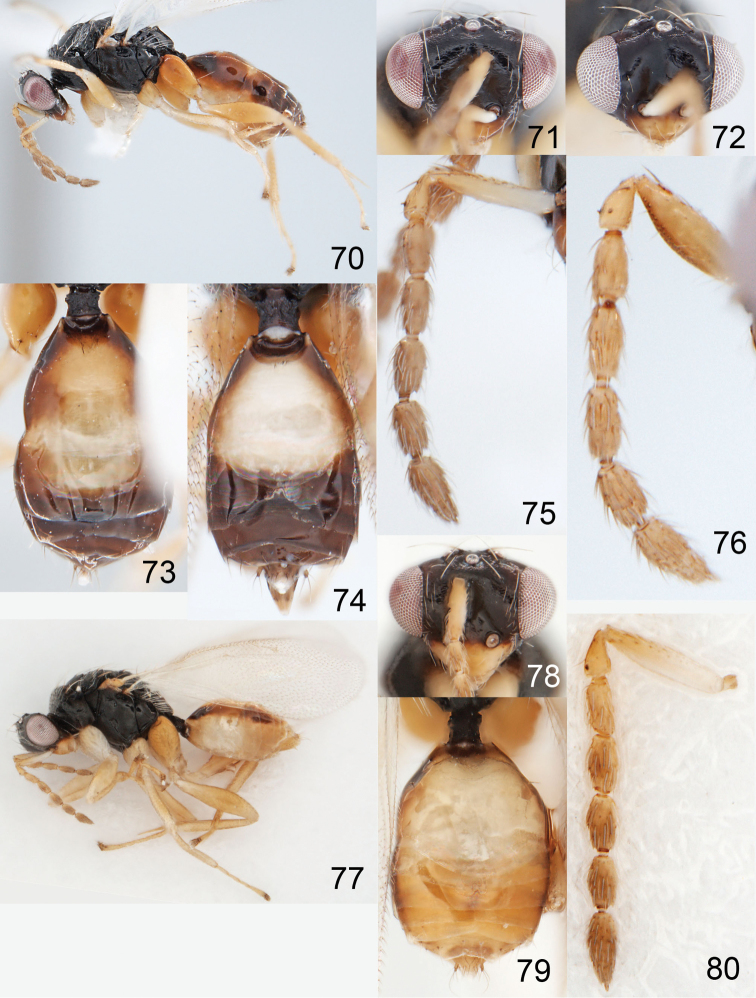
*Euplectrus* spp. **70–76** *Euplectrus andybennetti* (94-SRNP-5588, no barcode): **70** habitus in lateral view, female **71** head in frontal view, female **72** head in frontal view, male **73** gaster in dorsal view, female **74** gaster in dorsal view, male **75** antenna in lateral view, female **76** antenna in lateral view, male **77–80** *Euplectrus charlesporteri* (97-SRNP-9717, no barcode), female: **77** habitus in lateral view **78** head in frontal view **79** gaster in dorsal view **80** antenna in lateral view.

**Figures 81–86.**
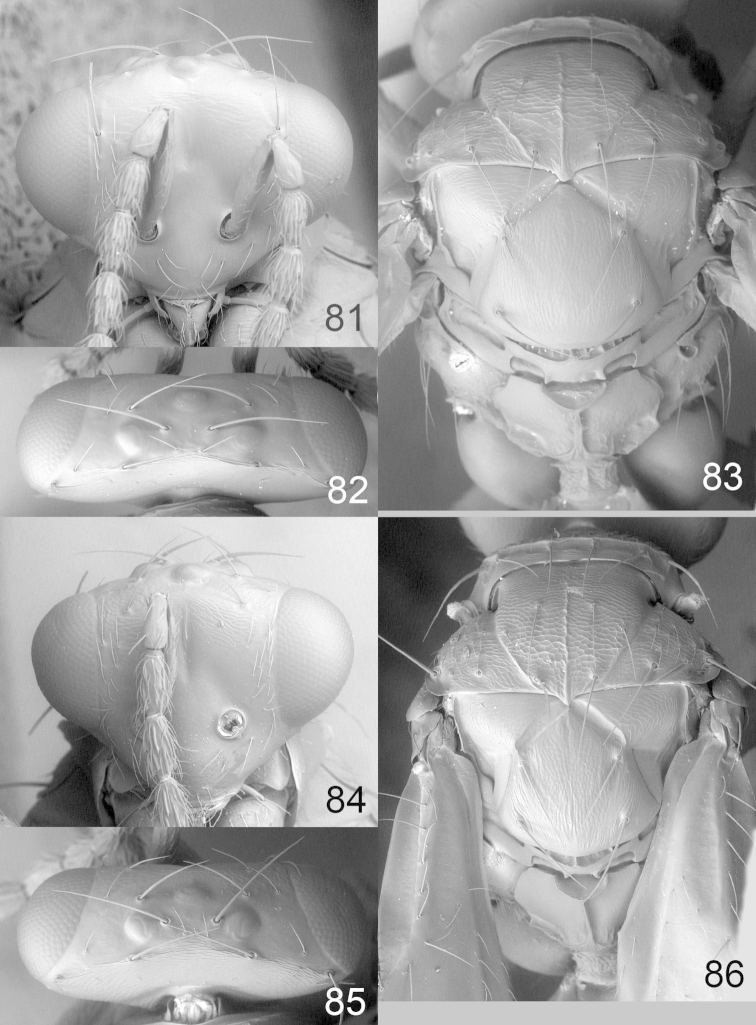
*Euplectrus* spp., females. **81–83** *Euplectrus andybennetti* (94-SRNP-5588, no barcode): **81** head in frontal view **82** vertex **83** mesosoma in dorsal view **84–86** *Euplectrus charlesporteri* (97-SRNP-9717, no barcode): **84** head in frontal view **85** vertex **86** mesosoma in dorsal view.

**Figures 87–97.**
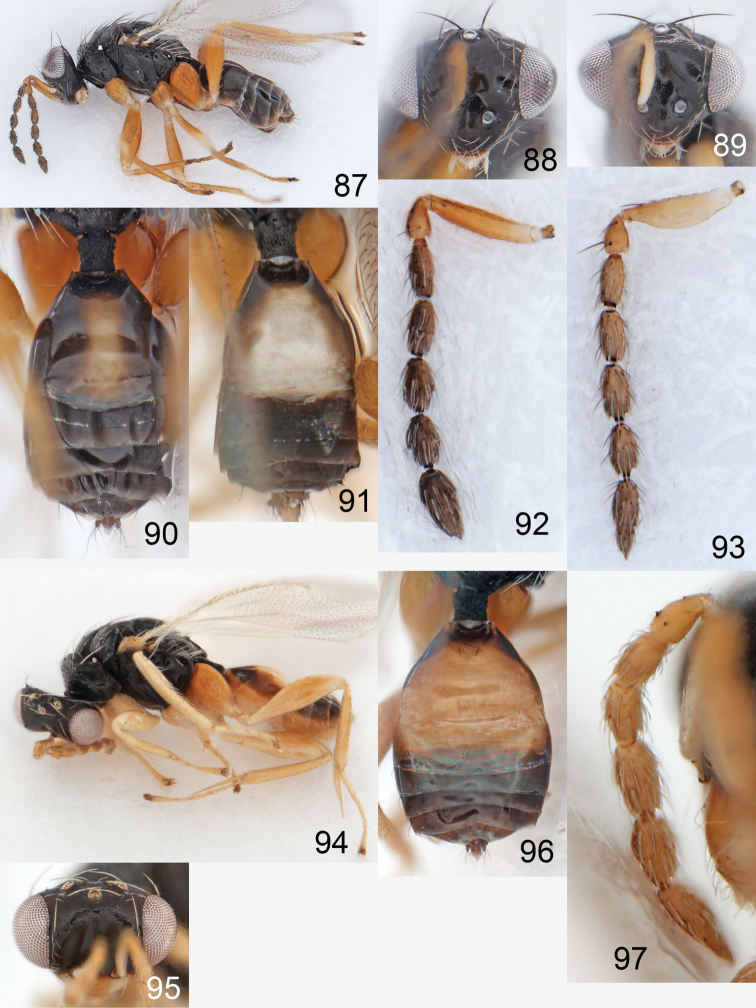
*Euplectrus* spp. **87–93** *Euplectrus andydeansi* (DHJPAR0046907): **87** habitus in lateral view, female **88** head in frontal view, female **89** head in frontal view, male **90** gaster in dorsal view, female **91** gaster in dorsal view, male **92** antenna in lateral view, female **93** antenna in lateral view, male **94–97** *Euplectrus chrisgrinteri* (95-SRNP-3415, no barcode), female: **94** habitus in lateral view **95** head in frontal view **96** gaster in dorsal view **97** antenna in lateral view.

**Figures 98–103.**
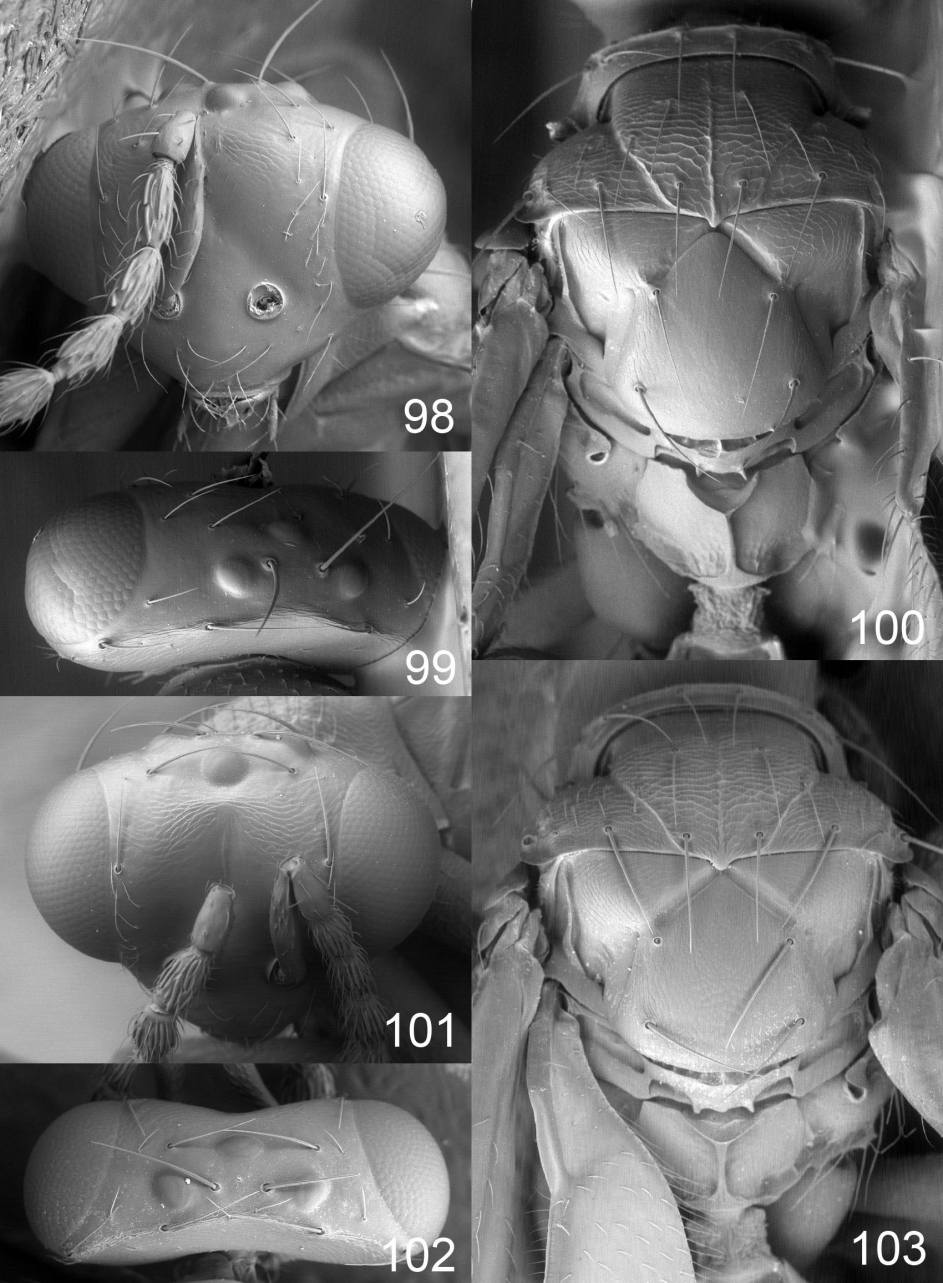
*Euplectrus* spp., females. **98–100** *Euplectrus andydeansi* (DHJPAR0046907): **98** head in frontal view **99** vertex **100** mesosoma in dorsal view **101–103** *Euplectrus chrisgrinteri* (95-SRNP-3415, no barcode): **101** head in frontal view **102** vertex **103** mesosoma in dorsal view.

**Figures 104–114.**
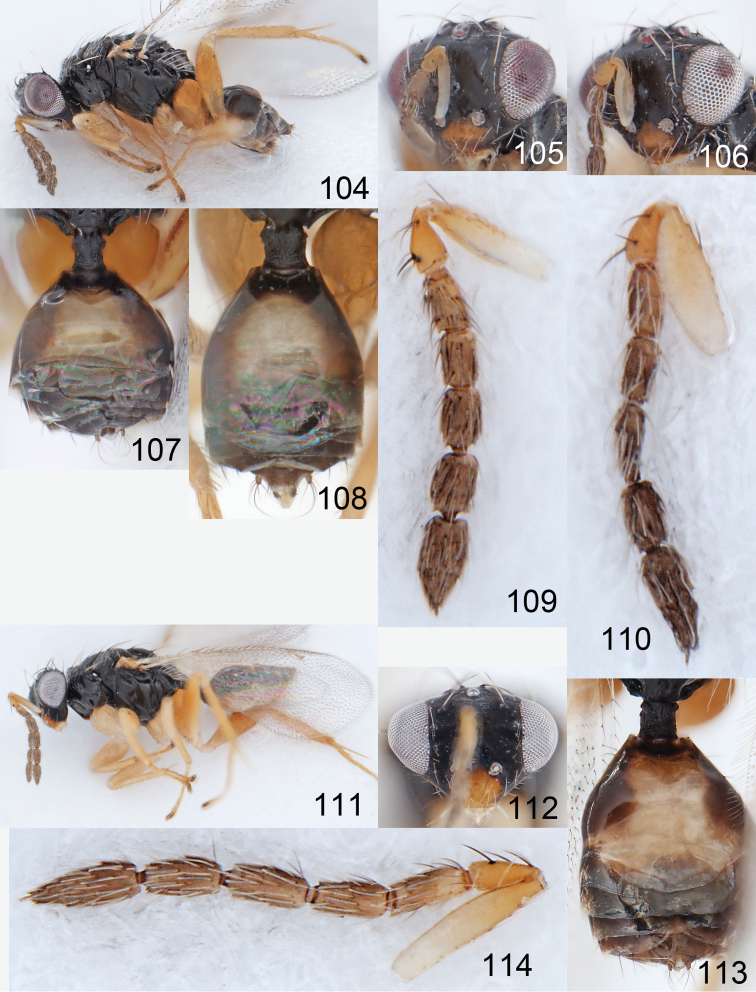
*Euplectrus* spp. **104–110** *Euplectrus annettewalkerae* (DHJPAR0046917): **104** habitus in lateral view, female **105** head in frontal view, female **106** head in frontal view, male **107** gaster in dorsal view, female **108** gaster in dorsal view, male **109** antenna in lateral view, female **110** antenna in lateral view, male; **111–114** *Euplectrus daveroubiki* (DHJPAR0023280), female: **111** habitus in lateral view **112** head in frontal view **113** gaster in dorsal view **114** antenna in lateral view.

**Figures 115–120.**
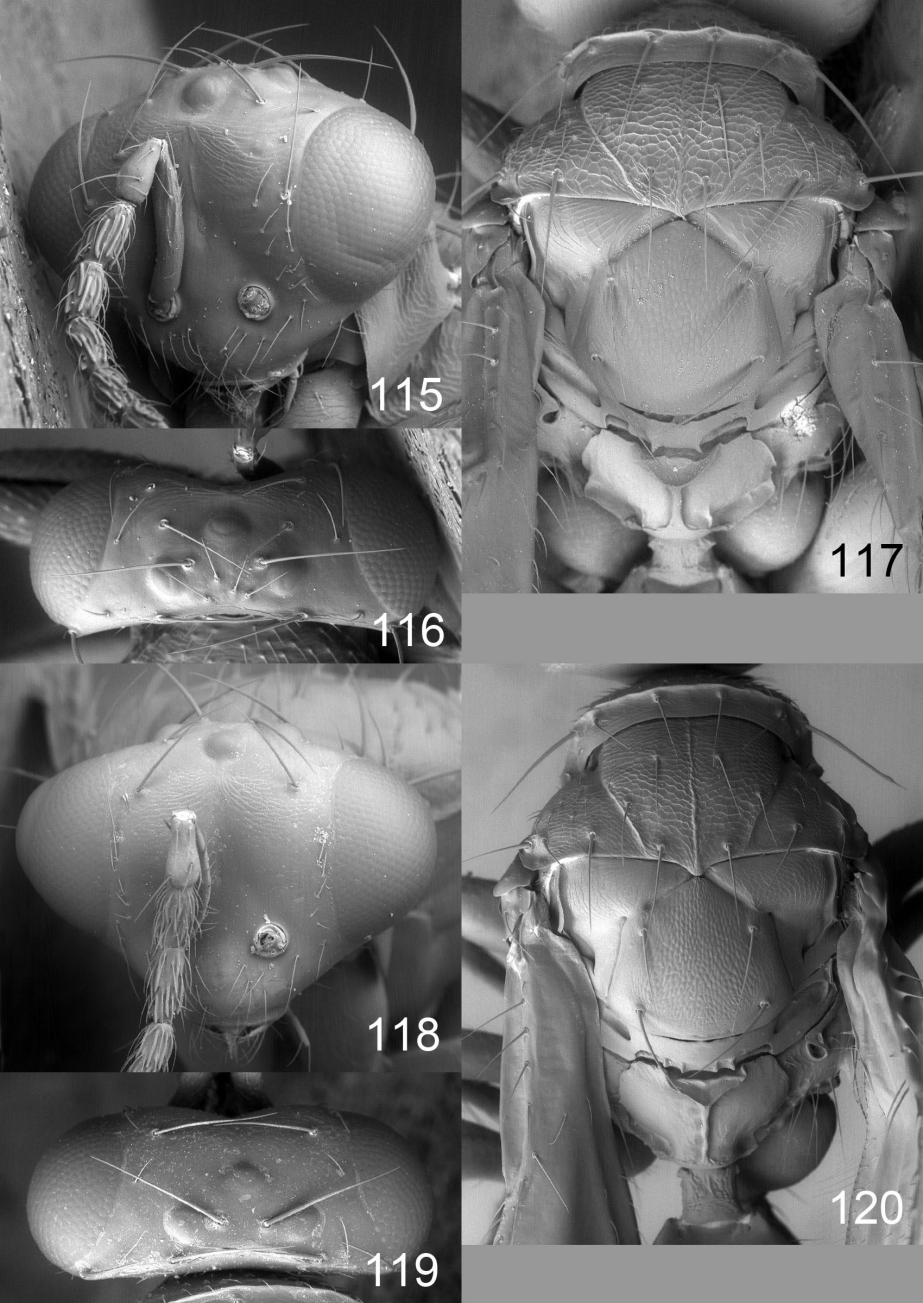
*Euplectrus* spp., females. **115–117** *Euplectrus annettewalkerae* (DHJPAR0046917): **115** head in frontal view **116** vertex **117** mesosoma in dorsal view; **118–120** *Euplectrus daveroubiki* (DHJPAR0023280): **118** head in frontal view **119** vertex **120** mesosoma in dorsal view.

**Figures 121–131.**
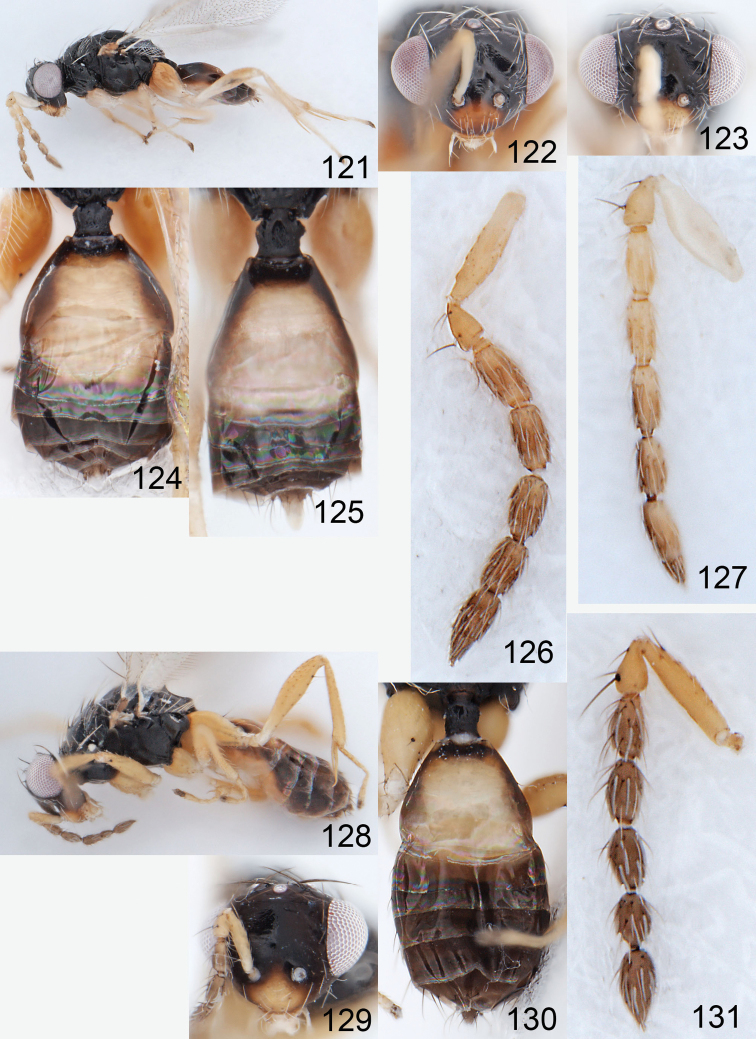
*Euplectrus* spp. **121–127** *Euplectrus billbrowni* (DHJPAR0028838): **121** habitus in lateral view, female **122** head in frontal view, female **123** head in frontal view, male **124** gaster in dorsal view, female **125** gaster in dorsal view, male **126** antenna in lateral view, female **127** antenna in lateral view, male; **128–131** *Euplectrus davesmithi* (DHJPAR0028811), female: **128** habitus in lateral view **129** head in frontal view **130** gaster in dorsal view **131** antenna in lateral view.

**Figures 132–137.**
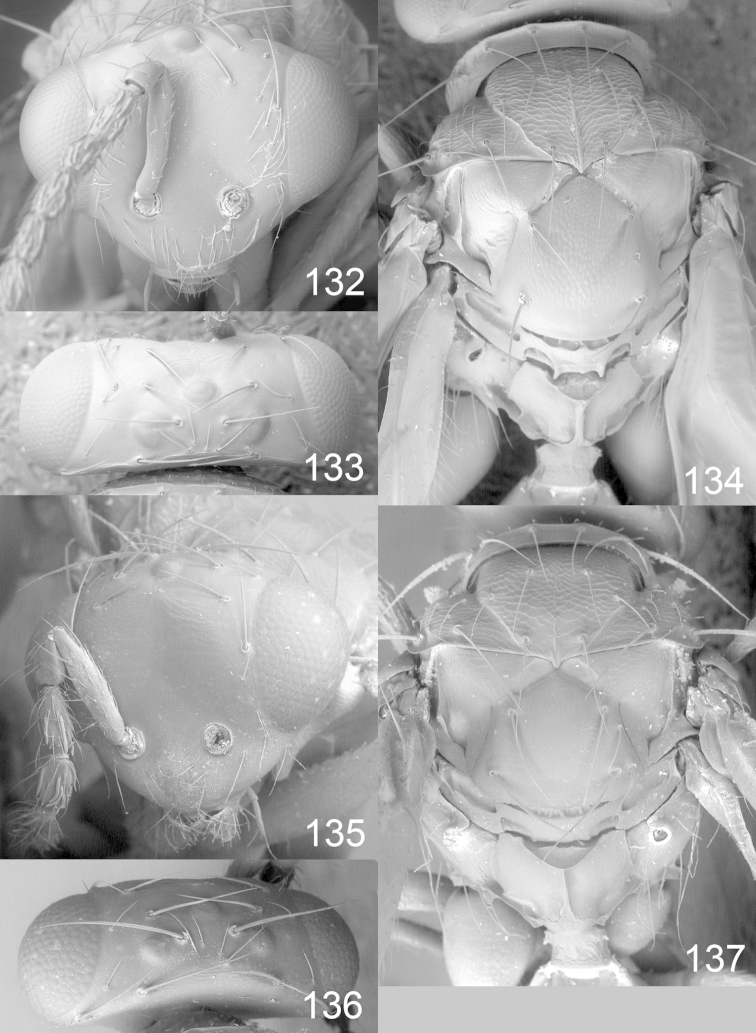
*Euplectrus* spp., females. **132–134** *Euplectrus billbrowni* (DHJPAR0028838): **132** head in frontal view **133** vertex **134** mesosoma in dorsal view; **135–137** *Euplectrus davesmithi* (DHJPAR0028811): **135** head in frontal view **136** vertex **137** mesosoma in dorsal view.

**Figures 138–148.**
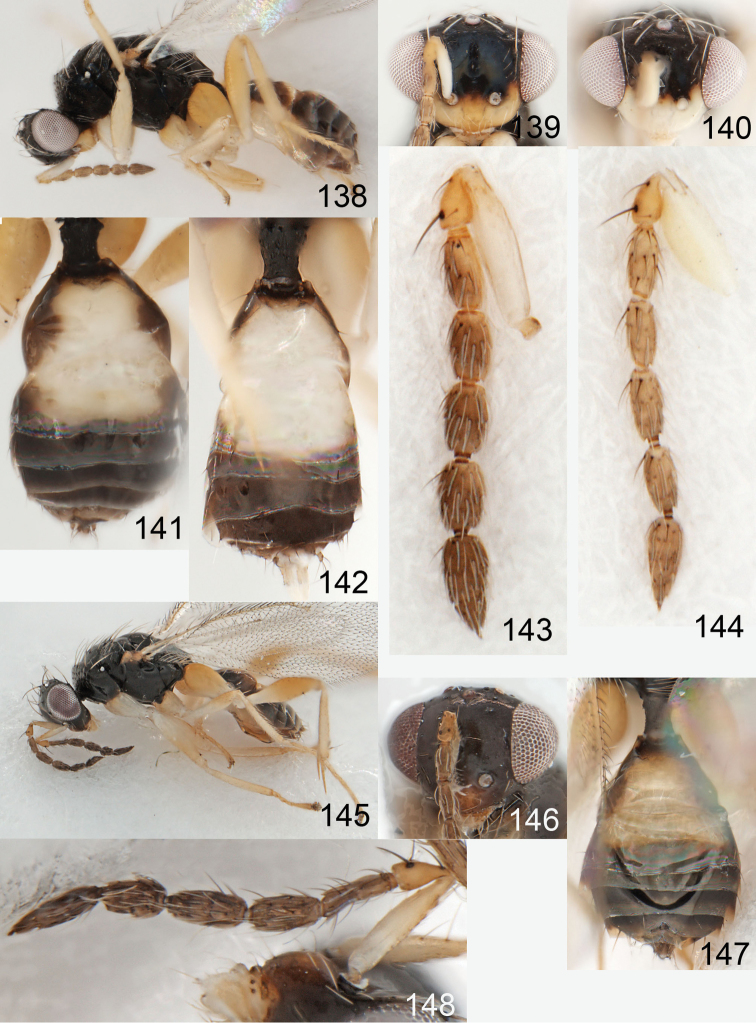
*Euplectrus* spp. **138–144** *Euplectrus carlosarmientoi* (DHJPAR0023273): **138** habitus in lateral view, female **139** head in frontal view, female **140** head in frontal view, male **141** gaster in dorsal view, female **142** gaster in dorsal view, male **143** antenna in lateral view, female **144** antenna in lateral view, male; **145–148** *Euplectrus dianariasae* (DHJPAR0046906), female: **145** habitus in lateral view **146** head in frontal view **147** gaster in dorsal view **148** antenna in lateral view.

**Figures 149–154.**
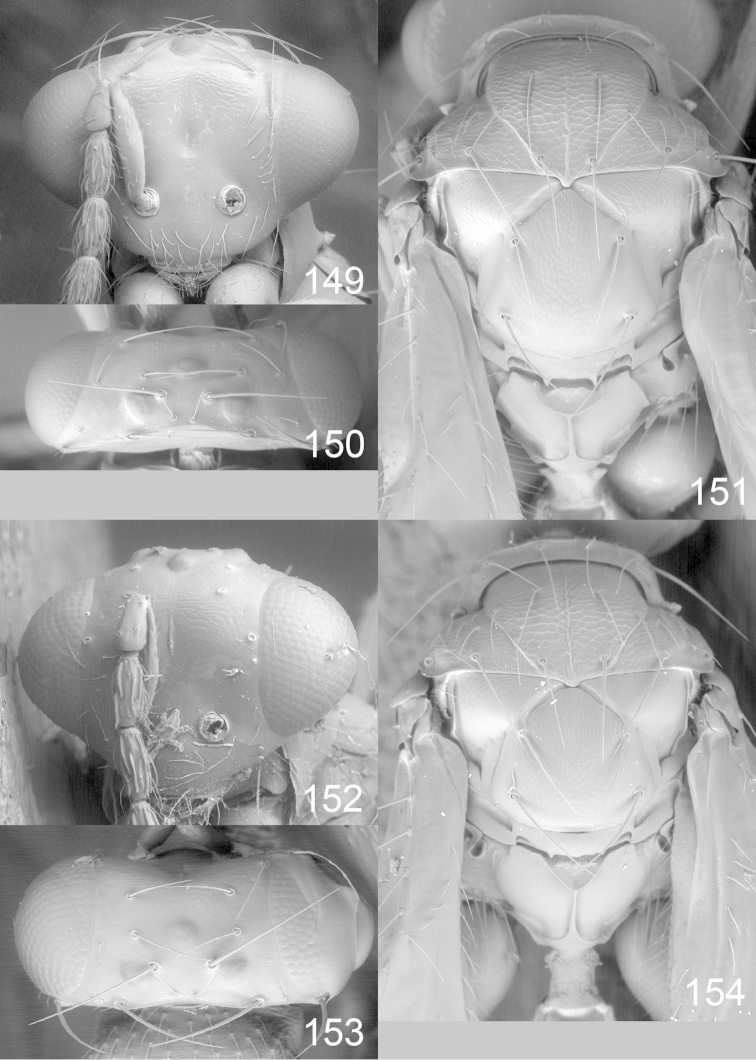
*Euplectrus* spp., females. **149–151** *Euplectrus carlosarmientoi* (DHJPAR0023273): **149** head in frontal view **150** vertex **151** mesosoma in dorsal view; **152–154** *Euplectrus dianariasae* (DHJPAR0046906): **152** head in frontal view **153** vertex **154** mesosoma in dorsal view.

**Figures 155–165.**
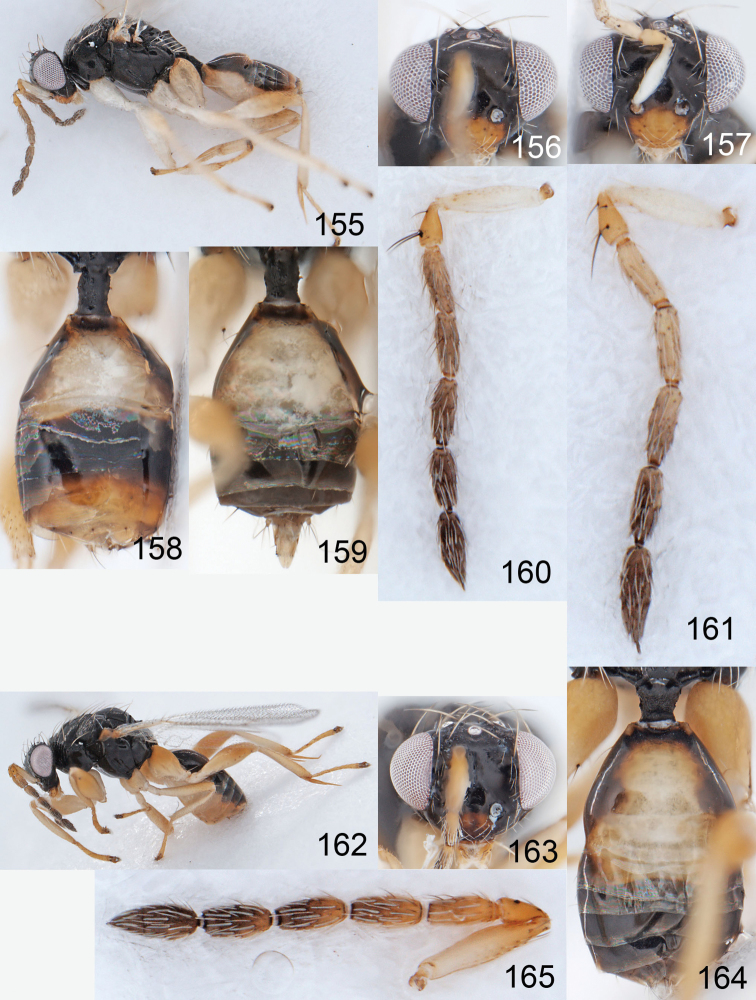
*Euplectrus* spp. **155–161** *Euplectrus carlrettenmeyeri* (DHJPAR0046912): **155** habitus in lateral view, female **156** head in frontal view, female **157** head in frontal view, male **158** gaster in dorsal view, female **159** gaster in dorsal view, male **160** antenna in lateral view, female **161** antenna in lateral view, male **162–165** *Euplectrus garygibsoni* (DHJPAR0042124), female: **162** habitus in lateral view **163** head in frontal view **164** gaster in dorsal view **165** antenna in lateral view.

**Figures 166–171.**
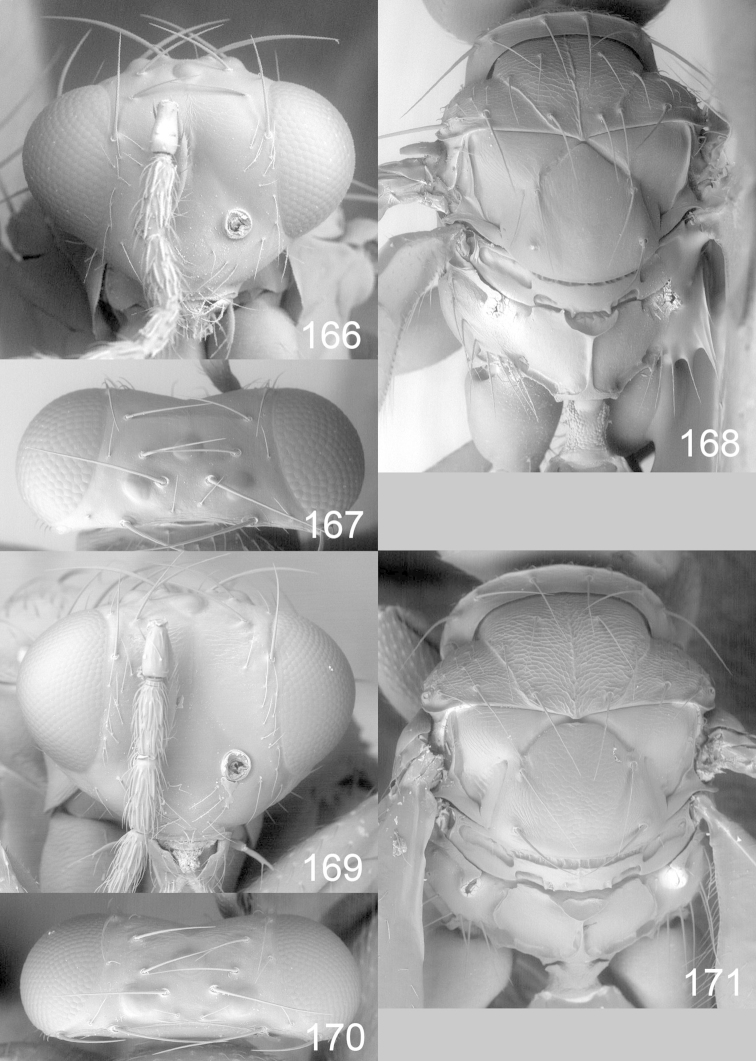
*Euplectrus* spp., females. **166–168** *Euplectrus carlrettenmeyeri* (DHJPAR0046912): **166** head in frontal view **167** vertex **168** mesosoma in dorsal view; **169–171** *Euplectrus garygibsoni* (DHJPAR0042124): **169** head in frontal view **170** vertex **171** mesosoma in dorsal view.

**Figures 172–182.**
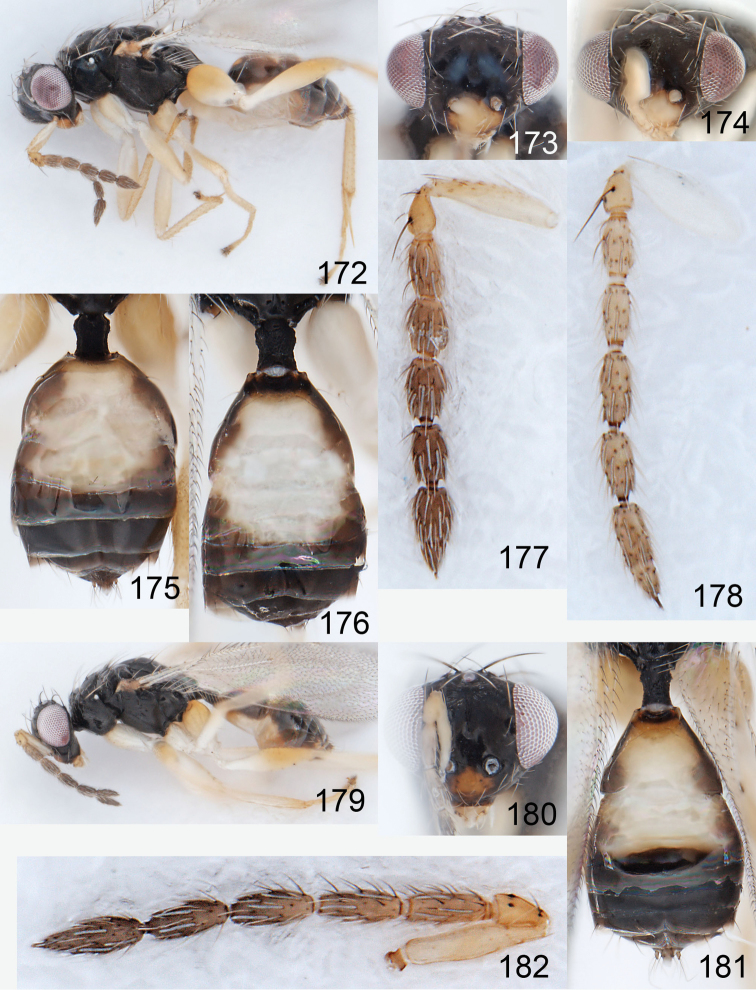
*Euplectrus* spp. **172–178** *Euplectrus charlesmicheneri* (DHJPAR0031152): **172** habitus in lateral view, female **173** head in frontal view, female **174** head in frontal view, male **175** gaster in dorsal view, female **176** gaster in dorsal view, male **177** antenna in lateral view, female **178** antenna in lateral view, male; **179–182** *Euplectrus gavinbroadi* (DHJPAR0045450), female: **179** habitus in lateral view **180** head in frontal view **181** gaster in dorsal view **182** antenna in lateral view.

**Figures 183–188.**
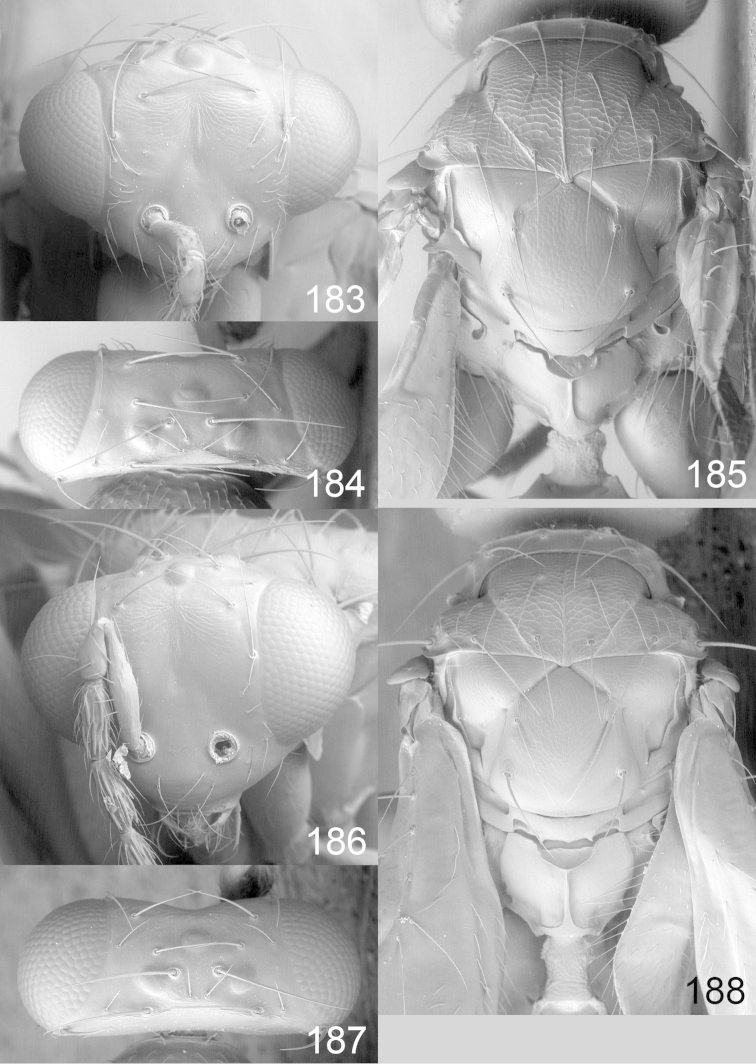
*Euplectrus* spp., females. **183–185** *Euplectrus charlesmicheneri* (DHJPAR0031152): **183** head in frontal view **184** vertex **185** mesosoma in dorsal view; **186–188** *Euplectrus gavinbroadi* (DHJPAR0045450): **186** head in frontal view **187** vertex **188** mesosoma in dorsal view.

**Figures 189–199.**
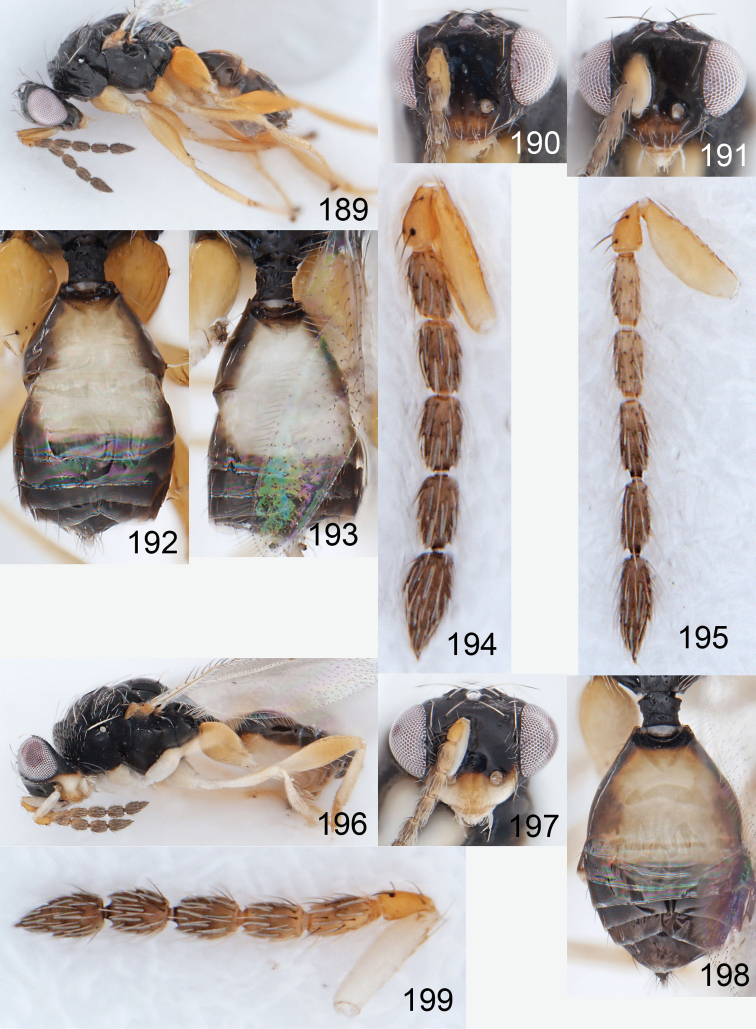
*Euplectrus* spp. **189–195** *Euplectrus chrisdarlingi* (DHJPAR0028698): **189** habitus in lateral view, female **190** head in frontal view, female **191** head in frontal view, male **192** gaster in dorsal view, female **193** gaster in dorsal view, male **194** antenna in lateral view, female **195** antenna in lateral view, male; **196–199** *Euplectrus gerarddelvarei* (DHJPAR0042126), female: **196** habitus in lateral view **197** head in frontal view **198** gaster in dorsal view **199** antenna in lateral view.

**Figures 200–205.**
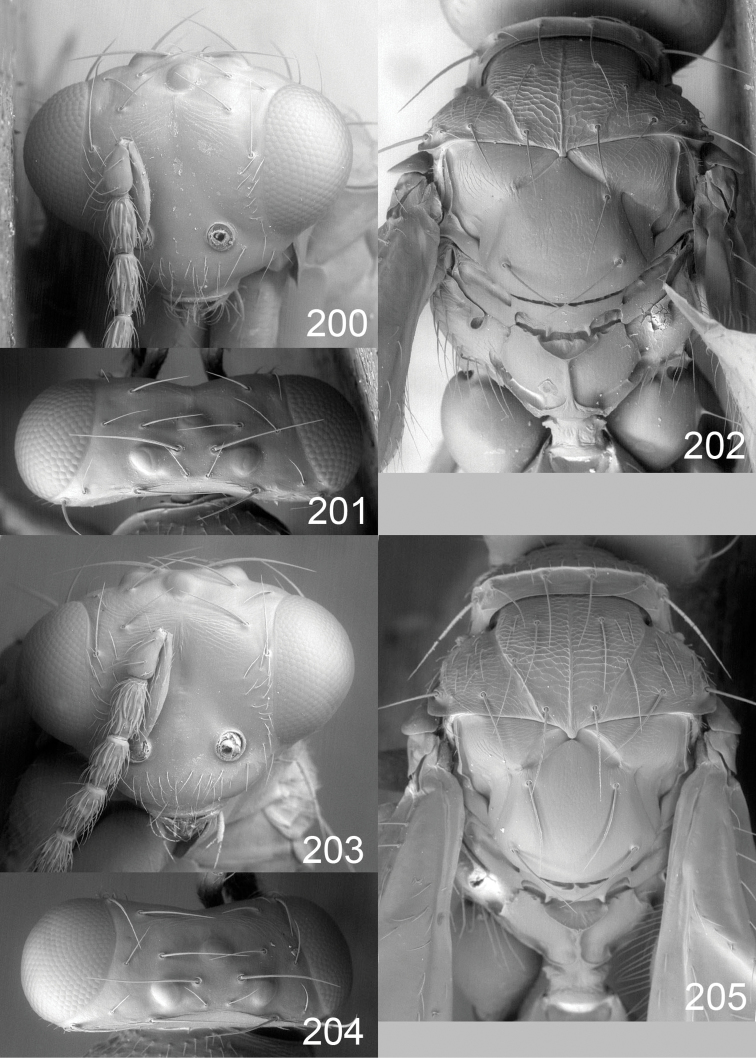
*Euplectrus* spp., females. **200–202** *Euplectrus chrisdarlingi* (DHJPAR0028698): **200** head in frontal view **201** vertex **202** mesosoma in dorsal view; **203–205** *Euplectrus gerarddelvarei* (DHJPAR0042126): **203** head in frontal view **204** vertex **205** mesosoma in dorsal view.

**Figures 206–216.**
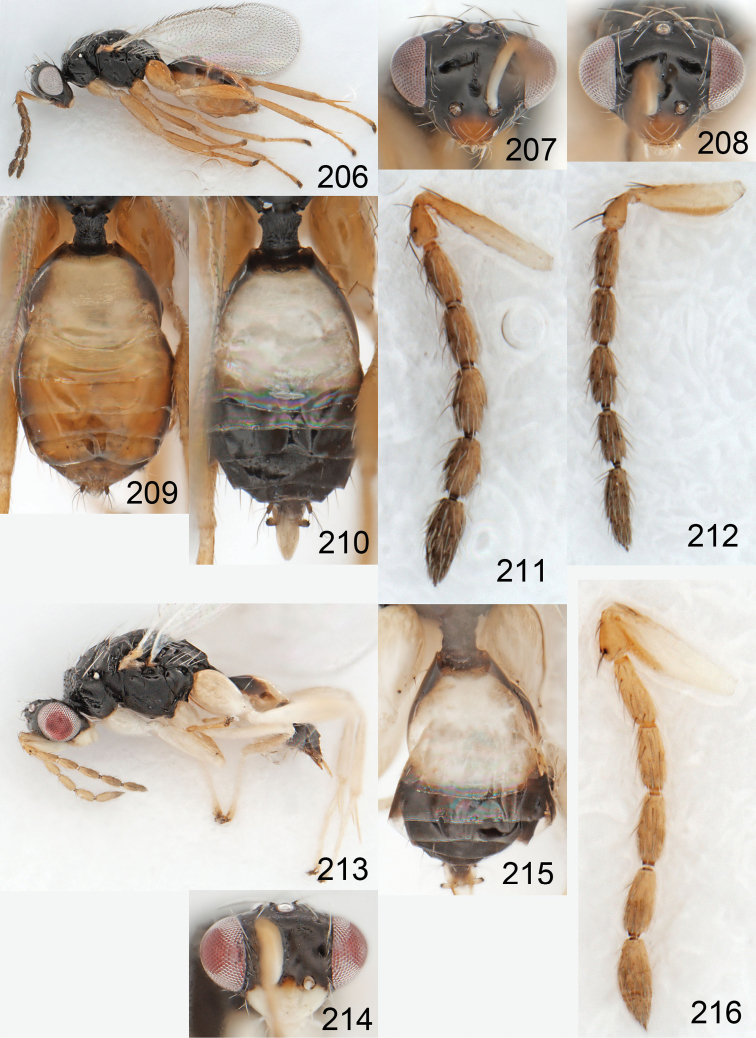
*Euplectrus* spp. **206–212** *Euplectrus corriemoreauae* (DHJPAR0053131): **206** habitus in lateral view, female **207** head in frontal view, female **208** head in frontal view, male **209** gaster in dorsal view, female **210** gaster in dorsal view, male **211** antenna in lateral view, female **212** antenna in lateral view, male **213–216** *Euplectrus howelldalyi* (DHJPAR0028882), male: **213** habitus in lateral view **214** head in frontal view **215** gaster in dorsal view **216** antenna in lateral view.

**Figures 217–222.**
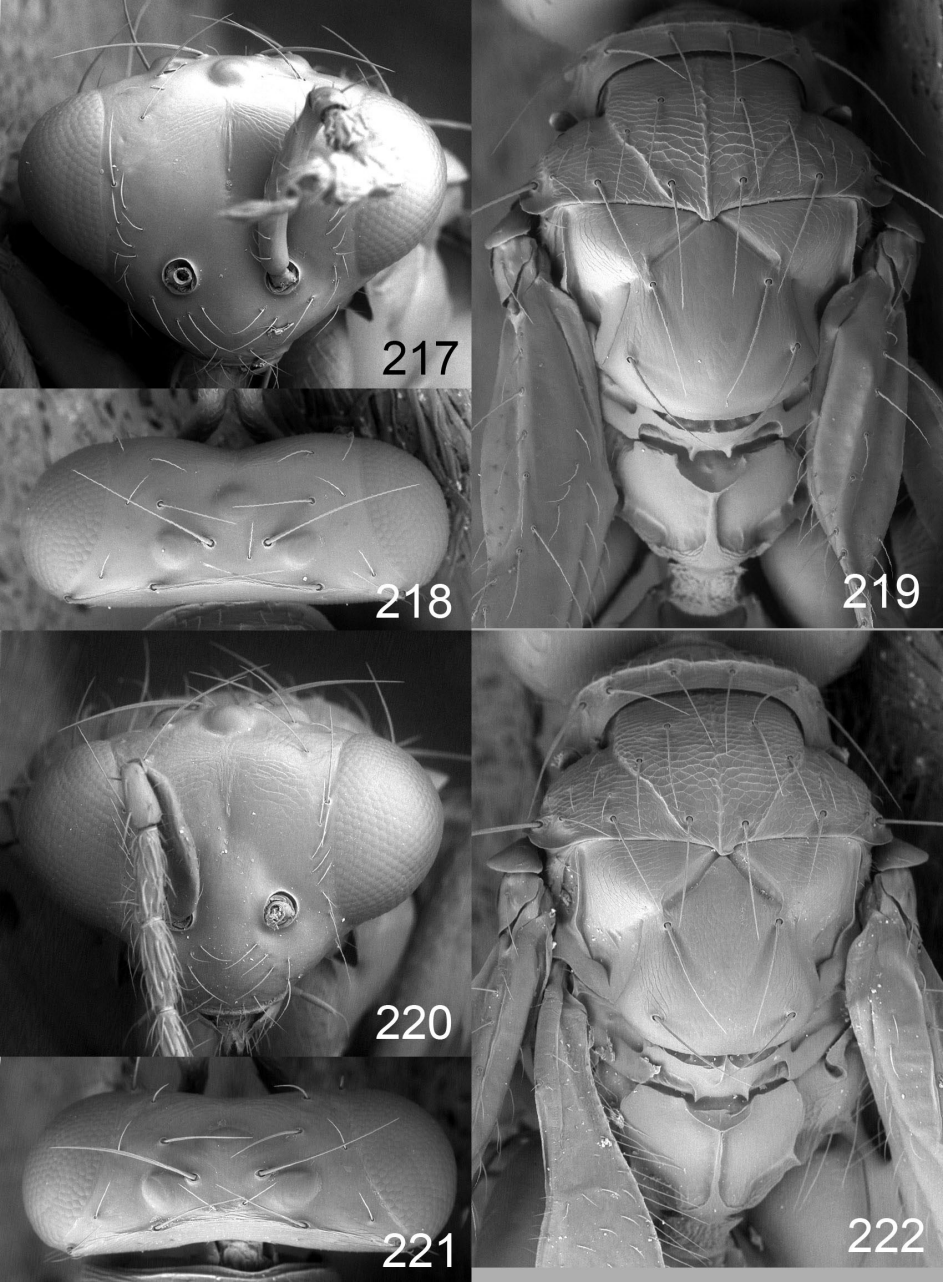
*Euplectrus* spp. **217–219** *Euplectrus corriemoreauae*, female (DHJPAR0053131): **217** head in frontal view **218** vertex **219** mesosoma in dorsal view; **220–222** *Euplectrus howelldalyi*, male (DHJPAR0028882): **220** head in frontal view **221** vertex **222** mesosoma in dorsal view.

*Euplectrus davesmithi* and *Euplectrus victoriapookae* have the same barcode (Fig. 35, Suppl. material 1), but as they are morphologically distinct and their hosts are very different we treat them as separate species.

#### Euplectrus davidwahli

Taxon classificationAnimaliaHymenopteraEulophidae

Hansson sp. n.

http://zoobank.org/0F86C864-2769-4C95-8167-833B08A1A7AB

Figures 14–15 , 223–229 , 234–236 , 750

##### Material.

Holotype a female labeled “COSTA RICA: Guanacaste, ACG, Sector San Cristobal, Tajo Angeles, 25.x.2010, G. Sihezar, ex *Hypena* Poole36 eating *Drymonia macrophylla*, sibling of wasp DHJPAR0042122, 10-SRNP-6218” (BMNH). PARATYPES: 10♀ 5♂, COSTA RICA (ACG): **Guanacaste:** 6♀ 1♂ with same label data as holotype (BMNH, INBio, MZLU, USNM); Sector Pitilla: Estación Pitilla, 1.iii.2011, M. Rios, ex *Hypena* Poole36 eating *Drymonia alloplectoides*, sibling of wasp DHJPAR0042397, 11-SRNP-30665 (1♀ 1♂, in INBio); Estación Quica, 8.i.2013, R. Calero, ex *Hypena* Poole36 eating *Drymonia alloplectoides*, sibling of wasp DHJPAR0051510, 13-SRNP-70030 (1♀ 2♂, in BMNH); Quebradona, 7.i.2012, R. Calero, ex *Hypena* Poole36 eating *Columnea hirta*, 12-SRNP-70083 and 12-SRNP-70084, no barcode (2♀ 1♂, in CNC, INBio).

##### Diagnosis.

**Figures 223–233.**
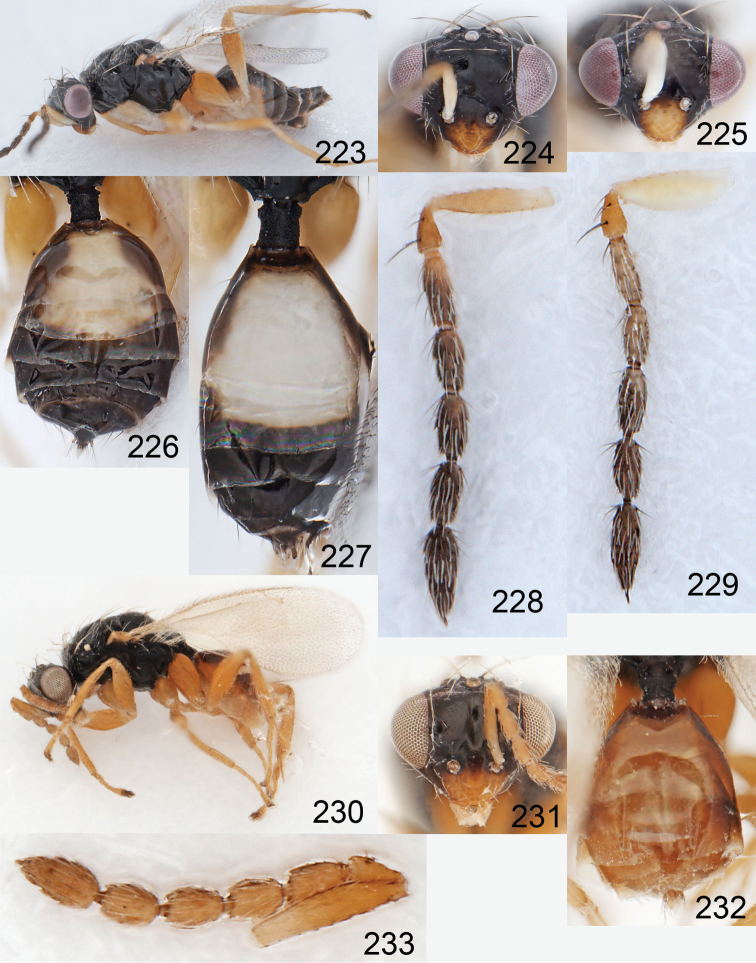
*Euplectrus* spp. **223–229** *Euplectrus davidwahli* (DHJPAR0042122): **223** habitus in lateral view, female **224** head in frontal view, female **225** head in frontal view, male **226** gaster in dorsal view, female **227** gaster in dorsal view, male **228** antenna in lateral view, female **229** antenna in lateral view, male; **230–233** *Euplectrus jesusugaldei* (97-SRNP-9370, no barcode), female: **230** habitus in lateral view **231** head in frontal view **232** gaster in dorsal view **233** antenna in lateral view.

Lower face with median part reddish-brown (female, Fig. 224) or yellowish-brown (male, Fig. 225), pale area reaching to outer lateral margins of toruli; legs yellowish-brown, fore and mid coxae yellowish-white in female (Fig. 223), white in male; petiole 1.0–1.1× as long as wide; gaster dark brown with a yellowish-white ±T-shaped spot in anterior ½ in female (Fig. 226), with anterior ½ white with dark brown lateral margins and posterior ½ dark brown in male (Fig. 227); male antenna with scape slightly expanded and widest in the middle, 3.0× as long as wide (Fig. 229).

##### Description.

**Figures 234–239.**
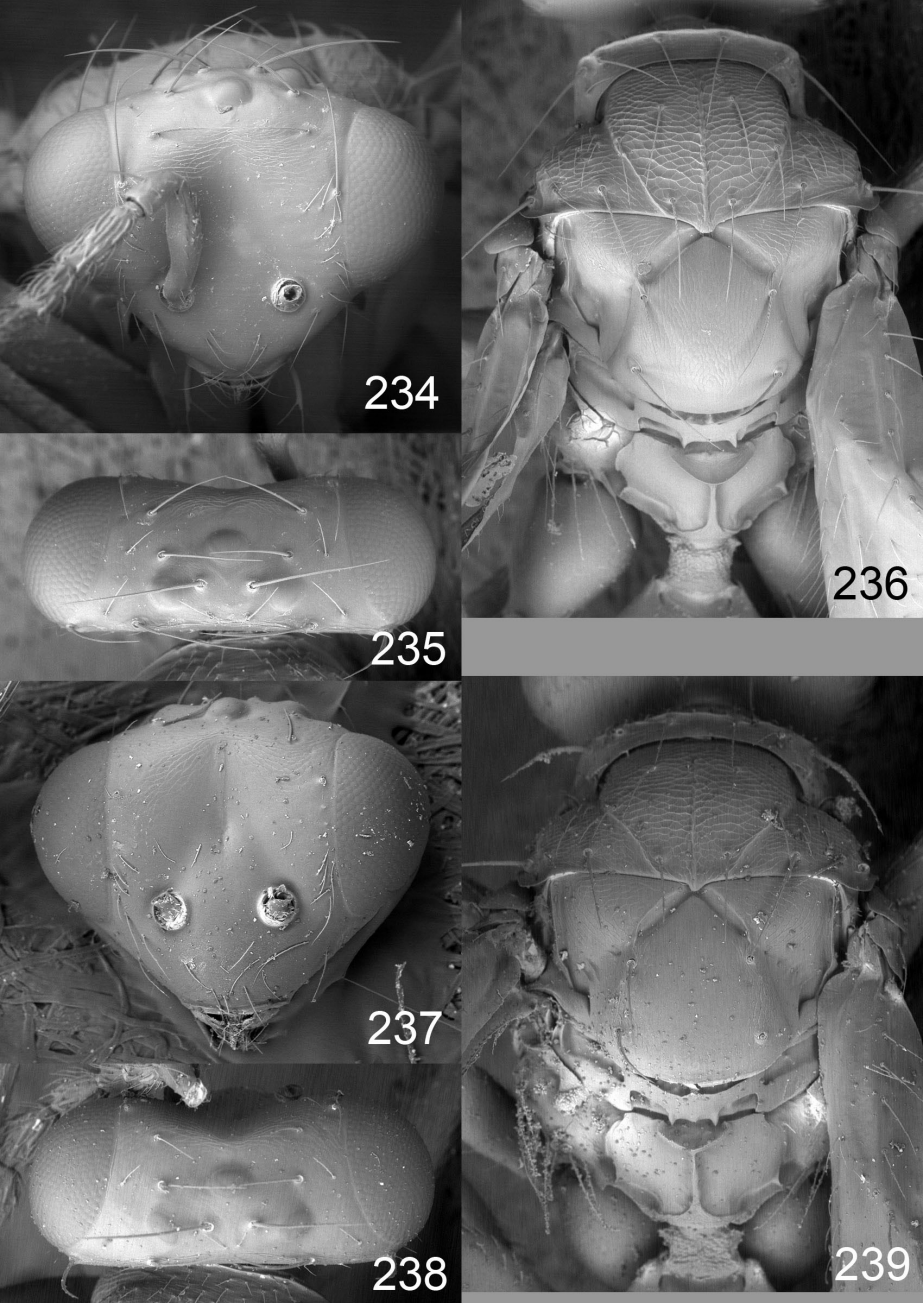
*Euplectrus* spp., females. **234–236** *Euplectrus davidwahli* (DHJPAR0042122): **234** head in frontal view **235** vertex **236** mesosoma in dorsal view; **237–239** *Euplectrus jesusugaldei* (97-SRNP-9370, no barcode): **237** head in frontal view **238** vertex **239** mesosoma in dorsal view.

*Female*. Length of body 2.5 mm. Antenna with scape yellowish-brown with base yellowish-white, pedicel yellowish-brown, flagellomeres dark brown (Fig. 228). Mandibles and palpi yellowish-white. Head black and shiny, lower face with median part reddish-brown, pale area reaching to outer lateral margins of toruli, with parts between pale area and eyes black (Fig. 224). Frons close to eyes with two rows of setae (Fig. 234). Vertex with very weak reticulation (Fig. 235). Occipital margin with a carina behind ocellar triangle (Fig. 235).

Mesosoma black and shiny (Fig. 223). Each sidelobe of mesoscutum with 14 setae. Scutellum 0.9× as long as wide; with weak engraved reticulation with slightly elongate meshes (Fig. 236). Dorsellum anteriorly with a groove that is divided by longitudinal carinae (Fig. 750), groove medially 0.3× as long as length of dorsellum. Propodeum with very weak reticulation (Fig. 750); anteromedially with a transverse semicircular cup; propodeal callus with eight setae. Legs yellowish-brown with fore and mid coxae yellowish-white (Fig. 223). Fore wing: costal cell with two rows of setae on ventral surface, and margin with five setae close to marginal vein; with 15 admarginal setae, in one row.

Gaster dark brown with a yellowish-white ±T-shaped spot in anterior ½ (Fig. 226).

Ratios. HE/MS/WM = 2.1/1.0/1.1; POL/OOL/POO = 7.4/4.2/1.0; OOL/DO = 1.2; WE/WF/WH/HH = 1.0/2.5/4.6/3.5; WH/WT = 1.1; PM/ST = 2.0; TS1/TS2/LT/LT1/LT2/LT3/LT4 = 3.7/2.0/6.0/2.0/1.4/1.0/1.4; LP/WP = 1.0–1.1; MM/LG = 0.9.

*Male*. Length of body 2.1 mm. Scape yellowish-white, slightly expanded and widest in the middle (Fig. 229), sensory pores confined to apicoventral ¾, sensory area pale as scape. Otherwise similar to female except lower face with pale part yellowish-brown (Fig. 225), fore and mid coxae white; gaster with anterior ½ white with dark brown lateral margins (Fig. 227).

Ratios. LC/WS = 3.0; MM/LG = 0.9.

##### Hosts and biology.

Feeding on last instar larva of *Hypena* Poole36 (Erebidae) feeding on *Drymonia macrophylla*, *Drymonia alloplectoides* and *Columnea hirta* (Gesneriaceae), parasitoid cocoons stuck to cadaver and leaf substrate.

##### Distribution.

Costa Rica (Guanacaste Province).

##### Etymology.

This species is named after David B. Wahl, in recognition of his contribution to the understanding of ACG Hymenoptera taxonomy.

#### Euplectrus dianariasae

Taxon classificationAnimaliaHymenopteraEulophidae

Hansson sp. n.

http://zoobank.org/E8C16AA7-82C2-43B6-A239-7DEA2D7CCDF1

Figures 145–148 , 152–154 , 751

##### Material.

Holotype a female labeled “COSTA RICA: Guanacaste, ACG, Sector Pitilla, Sendero Nacho, 27.ix.2011, M. Rios, ex *Antiblemma* Poole21DHJ02 eating *Meriania phlomoides*, sibling of wasp DHJPAR0048227, 11-SRNP-32924” (BMNH). PARATYPE: 1♀ “COSTA RICA: Guanacaste, ACG, Sector Pitilla, Sendero Laguna, 29.ix.2011, F. Quesada, ex Antiblemma Poole21DHJ02 eating Meriania phlomoides, sibling of wasp DHJPAR0046906, 11-SRNP-32973” (INBio).

##### Diagnosis.

Lower face with median part dark reddish-brown, pale area reaching to level of median part of toruli (Fig. 146); dorsellum without a groove or foveae along anterior margin (Fig. 751); fore and mid coxae white, remaining parts of fore and mid legs predominantly yellowish-white; hind leg yellowish-white (Fig. 145); petiole 1.3× as long as wide; gaster with anterior ½ yellowish-white with dark brown lateral margins, posterior ½ dark brown (Fig. 147). Morphologically identical to *Euplectrus gavinbroadi*.

##### Description.

*Female*. Length of body 2.2 mm. Antenna with scape and pedicel yellowish-brown, flagellomeres 1–2 pale brown, 3–6 dark brown (Fig. 148). Mandibles and palpi yellowish-white. Head black and shiny, lower face with median part dark reddish-brown, pale area reaching to level of median part of toruli, with parts between pale area and eyes black (Fig. 146). Frons close to eyes with one sparse row of setae (Fig. 152). Vertex with very weak reticulation (Fig. 153). Occipital margin with a carina behind ocellar triangle (Fig. 153).

Mesosoma black and shiny (Fig. 145). Each sidelobe of mesoscutum with eight setae. Scutellum 1.0× as long as wide, with weak reticulation (Fig. 154). Dorsellum without a groove or foveae along anterior margin (Fig. 751). Propodeum with very weak reticulation (Fig. 751); anteromedially with a semicircular cup; propodeal callus with nine setae. Legs (Fig. 145): fore and mid coxae white, remaining parts of fore and mid legs predominantly yellowish-white; hind leg yellowish-white. Fore wing: costal cell on ventral surface with one complete row of setae and with a short row with three setae in front of complete row, and margin with five setae in apical ½; with 19 admarginal setae, mainly in one row but with three rows in apical ⅓.

Gaster with anterior ½ yellowish-white with dark brown lateral margins, posterior ½ dark brown (Fig. 147).

Ratios. HE/MS/WM = 2.4/1.0/1.3; POL/OOL/POO = 4.3/1.9/1.0; OOL/DO = 1.2; WE/WF/WH/HH = 1.0/2.1/4.3/3.4; WH/WT = 1.1; PM/ST = 1.5; TS1/TS2/LT/LT1/LT2/LT3/LT4 = 3.8/2.4/6.2/1.6/1.3/1.0/1.8; LP/WP = 1.3; MM/LG = 1.0.

*Male*. Unknown.

##### Hosts and biology.

Feeding on intermediate and third instar larvae of *Antiblemma* Poole21DHJ02 (Erebidae) feeding on *Meriania phlomoides* (Melastomataceae), parasitoid cocoons stuck to dead larva and substrate.

##### Distribution.

Costa Rica (Guanacaste Province).

##### Etymology.

This species is named after Diana C. Arias-Penna, in recognition of her contribution to the understanding of ACG Hymenoptera taxonomy.

#### Euplectrus donquickei

Taxon classificationAnimaliaHymenopteraEulophidae

Hansson sp. n.

http://zoobank.org/FA6AA06E-D3F0-4506-87EA-BE34FE4B1B7F

Figures 240–246 , 251–253 , 752

##### Material.

Holotype a female labeled “COSTA RICA: Guanacaste, ACG, Sector Cacao, Quebrada Otilio, 29.vii.2005, M. Pereira, ex *Hemicephalis* alesaDHJ01 eating *Varronia inermis*, sibling of wasp DHJPAR0028814, 05-SRNP-46906” (BMNH). PARATYPES: 7♀ 1♂ with same label data as holotype (BMNH, INBio, MZLU, USNM).

##### Diagnosis.

**Figures 240–250.**
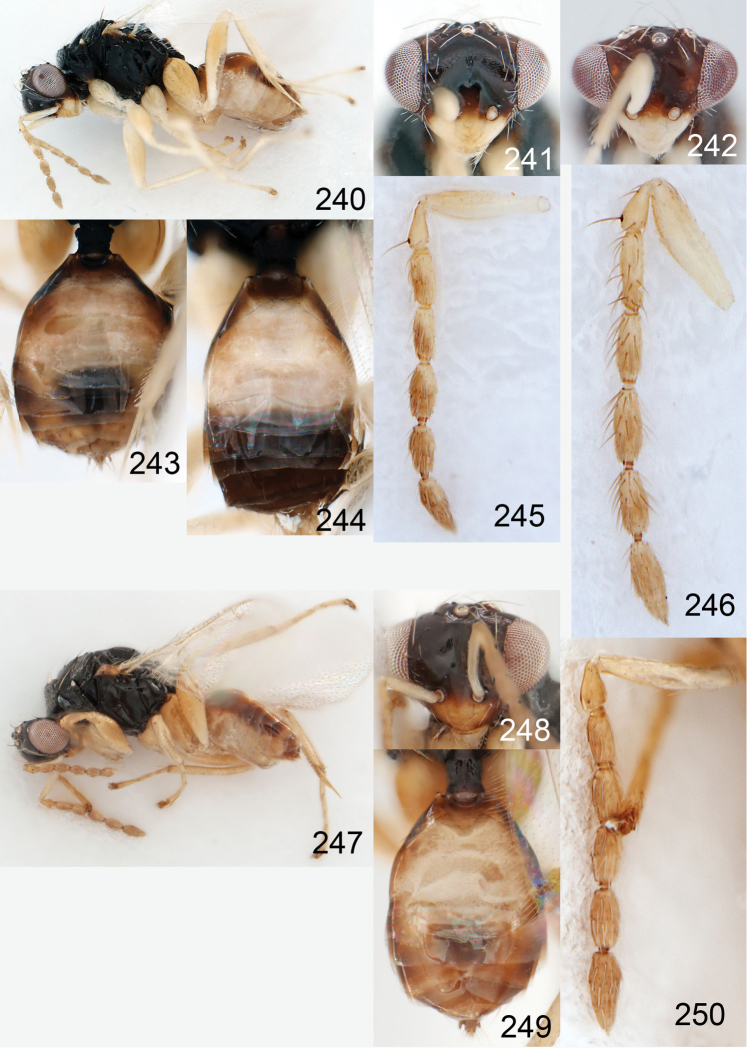
*Euplectrus* spp. **240–246** *Euplectrus donquickei* (DHJPAR0028814): **240** habitus in lateral view, female **241** head in frontal view, female **242** head in frontal view, male **243** gaster in dorsal view, female **244** gaster in dorsal view, male **245** antenna in lateral view, female **246** antenna in lateral view, male; **247–250** *Euplectrus jjrodriguezae* (94-SRNP-7129, no barcode), female: **247** habitus in lateral view **248** head in frontal view **249** gaster in dorsal view **250** antenna in lateral view.

Head dark reddish-brown with lower face medially white with median part yellowish-white (female, Fig. 241) or completely white (male, Fig. 242), pale area reaching outside level of outer margins of toruli; scutellum with a small hump posteromedially (Fig. 253); legs yellowish-white with hind coxa and hind femur yellowish-brown (Fig. 240); petiole 0.8× as long as wide; female gaster with anterior ½ yellowish-brown and posterior ½ pale brown with a dark brown spot anteromedially, entire gaster with dark brown lateral margins (Fig. 243), male gaster with anterior ½ yellowish-white with dark brown anterolateral margins and posterior ½ dark brown (Fig. 244); male antenna with scape slightly expanded and widest in the middle, 3.2× as long as wide (Fig. 246), and with sensory area pale brown.

##### Description.

**Figures 251–256.**
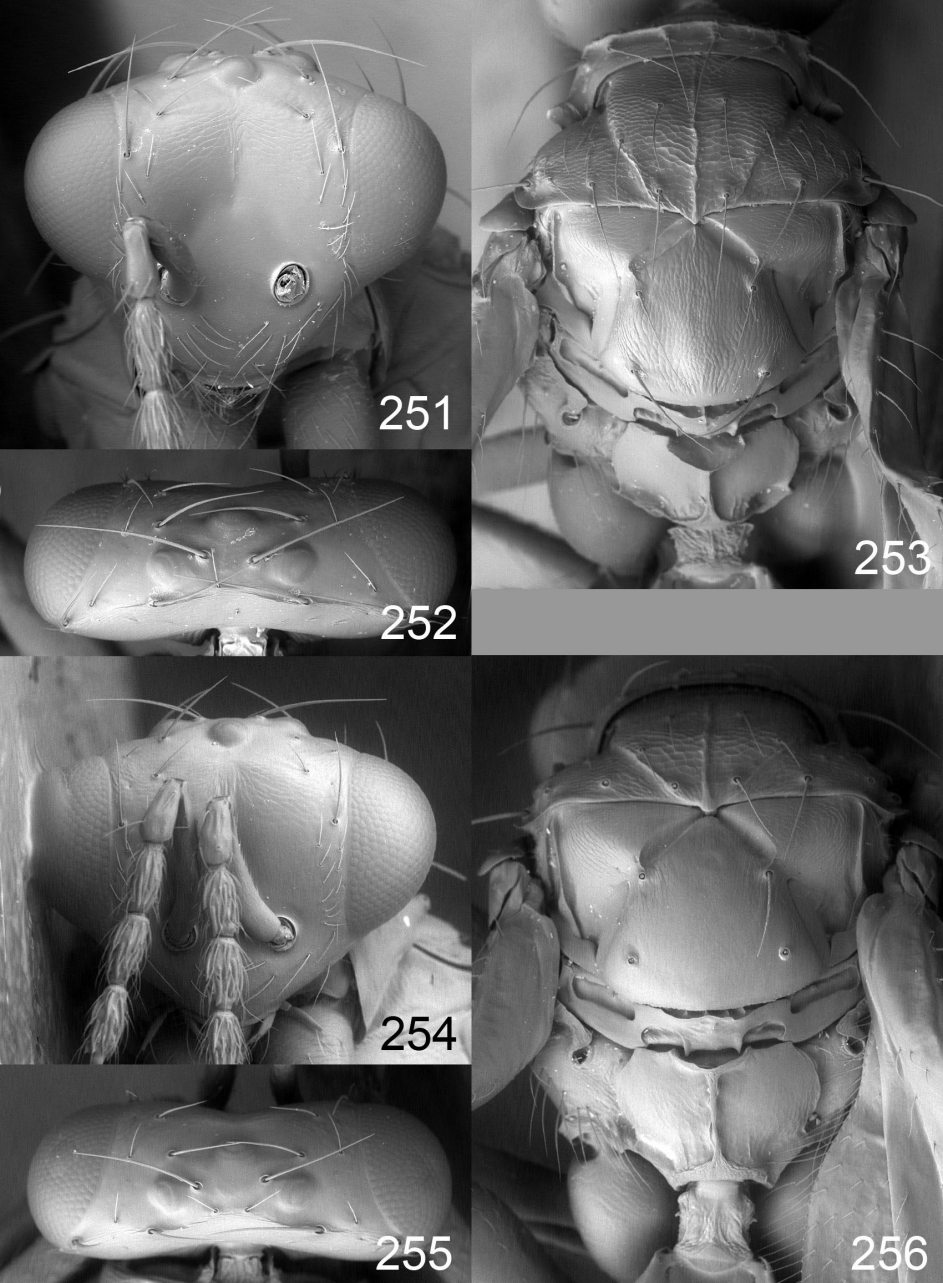
*Euplectrus* spp., females. **251–253** *Euplectrus donquickei* (DHJPAR0028814: **251** head in frontal view **252** vertex **253** mesosoma in dorsal view; **254–256** *Euplectrus jjrodriguezae* (94-SRNP-7129, no barcode): **254** head in frontal view **255** vertex **256** mesosoma in dorsal view.

*Female*. Length of body 2.4 mm. Antenna with scape, pedicel and flagellomeres 1–2 yellowish-white, flagellomeres 3–4 pale brown, 5–6 dark brown (Fig. 245). Mandibles and palpi yellowish-white. Head dark reddish-brown and shiny, lower face medially white with median part yellowish-white, pale area reaching outside level of outer margin of toruli, and parts between pale area and eyes dark reddish-brown (Fig. 241). Frons close to eyes with two rows of setae (Fig. 251). Vertex with very weak reticulation inside ocellar triangle, smooth outside (Fig. 252). Occipital margin with a carina (Fig. 252).

Mesosoma black and shiny (Fig. 240). Each sidelobe of mesoscutum with 11 setae. Scutellum convex, with a small hump posteromedially and with a weak groove anteromedially (Fig. 253); 0.9× as long as wide; with weak engraved reticulation. Dorsellum anteriorly with a groove that is divided into two by a median longitudinal carina (Fig. 752), groove medially 0.4× as long as length of dorsellum. Propodeum smooth (Fig. 752); anteromedially with a transverse triangular cup; propodeal callus with seven setae. Legs yellowish-white with hind coxa and hind femur yellowish-brown (Fig. 240). Fore wing: costal cell with two rows of setae on ventral surface, and margin with four setae close to marginal vein; with 20 admarginal setae, in basal ⅓ in two rows and in apical ⅔ in one row.

Gaster with anterior ½ yellowish-brown and posterior ½ pale brown with a dark brown spot anteromedially, entire gaster with dark brown lateral margins (Fig. 243).

Ratios. HE/MS/WM = 1.8/1.0/1.1; POL/OOL/POO = 5.1/3.1/1.0; OOL/DO = 1.3; WE/WF/WH/HH = 1.0/2.4/4.5/3.6; WH/WT = 1.1; PM/ST = 1.4; TS1/TS2/LT/LT1/LT2/LT3/LT4 = 3.1/2.2/7.1/2.7/1.7/1.0/1.7; LP/WP = 0.8; MM/LG = 1.1.

*Male*. Length of body 2.2 mm. Scape slightly expanded and widest in the middle (Fig. 246), sensory pores confined to apicoventral ⅔. Otherwise similar to female except pale part of lower face completely white (Fig. 242), gaster with anterior ½ yellowish-white with dark brown anterolateral margins and posterior ½ dark brown (Fig. 244).

Ratios. LC/WS = 3.2.

##### Hosts and biology.

Feeding on last instar larva of *Hemicephalis* alesaDHJ01 (Noctuidae) feeding on *Varronia inermis* (Boraginaceae), parasitoid cocoons stuck to dead larva and substrate.

##### Distribution.

Costa Rica (Guanacaste Province).

##### Etymology.

This species is named after Don L. J. Quicke, in recognition of his contribution to the understanding of ACG Hymenoptera taxonomy.

#### Euplectrus eowilsoni

Taxon classificationAnimaliaHymenopteraEulophidae

Hansson sp. n.

http://zoobank.org/4D240C7B-A2C7-4EB1-B04A-CCDF9D2F2B70

Figures 22 , 257–263 , 268–270 , 425 , 753

##### Material.

Holotype a female labeled “COSTA RICA: Alajuela, ACG, Brisanta, 25.vii.2007, D.Briceno, ex *Argyrosticta vauaurea* eating *Phlebodium pseudoaureum*, 07-SRNP-65277, sibling of wasp DHJPAR0028932” (BMNH). Paratypes: 173♀ 26♂: COSTA RICA (ACG): **Alajuela:** 45♀ 3♂ with same label data as holotype (BMNH, CNC, INBio, MZLU, MIUCR, USNM); Sendero Juntas, ex *Argyrosticta bellinita* eating *Campyloneurum brevifolium*, 1.v.2008, J.Perez, 08-SRNP-40975, sibling of wasp DHJPAR0031158 (30♀ 3♂, in BMNH, CNC, INBio, MIUCR, USNM); **Guanacaste:** Sector Pitilla: **Estación Quica:** ex *Argyrosticta bellinita* eating *Microgramma percussa*, 13.ii.2010, R.Calero, sibling of wasp DHJPAR0039215, 10-SRNP-70776 (11♀ 1♂, in BMNH, INBio); ex *Argyrosticta bellinita* eating *Microgramma 22028*, 24.i.2012, R.Calero, sibling of wasp DHJPAR0048966, 12-SRNP-70198 (6♀, in BMNH, INBio), from same host as previous but collected 21.ii.2012, sibling of wasp DHJPAR0048965, 12-SRNP-70197 (9♀, in BMNH, CNC, INBio, USNM); ex *Argyrosticta bellinita* eating *Campyloneurum angustifolium*, 20.x.2010, R. Calero, sibling of wasp DHJPAR0042138, 10-SRNP-73132 (2♂, in INBio); 20.x.2010, R. Calero, sibling of wasp DHJPAR0042136, 10-SRNP-73145 (2♂, in BMNH); ex *Argyrosticta aurifundensDHJ02* eating *Polypodium fraxinifolium*, 21.vi.2010, R. Calero, 10-SRNP-71891, sibling of wasp DHJPAR0040316 (7♀, in CNC, USNM); ex *Lophomyra tacitaDHJ02* eating *Microgramma percussa*, 15.ii.2010, R. Calero, 10-SRNP-70813, sibling of wasp DHJPAR0039211 (1♀ 1♂, in INBio); ex *Lophomyra tacita* eating *Microgramma percussa*, 11.iii.2013, sibling of wasp DHJPAR0052362, 13-SRNP-70422, (1♂, in BMNH); ex Noctuidae indet. eating *Campyloneurum angustifolium*, 28.ii.2011, M. Rios, 11-SRNP-70529, sibling of wasp DHJPAR0042398 (21♀ 3 ♂, in BMNH, CNC, INBio, MIUCR, USNM); 28.ii.2011, M. Rios, 11-SRNP-70530, sibling of wasp DHJPAR0042522 (1♀ 1♂, in BMNH); 5.v.2011, R. Calero, 11-SRNP-71022, sibling of wasp DHJPAR0043271 (11♀ 1♂, in INBio, USNM); **Quebradona:** ex *Argyrosticta bellinita* eating *Niphidium oblanceolatum*, 13.ii.2010, R. Calero, 10-SRNP-70785, sibling of wasp DHJPAR0039207 (1♀ 1♂, in INBio); 13.ii.2010, R.Calero, 10-SRNP-70789, sibling of wasp DHJPAR0039206 (1♀, in INBio); 15.iv.2010, R. Calero, 10-SRNP-71431, sibling of wasp DHJPAR0039591 (11♀ 2♂, in BMNH, CNC, USNM); 15.iv.2010, R. Calero, 10-SRNP-71432, sibling of wasp DHJPAR0039593 (1♀, in INBio); 15.iv.2010, R. Calero, 10-SRNP-71433, sibling of wasp DHJPAR0039592 (1♀ 1♂, in BMNH); ex Noctuidae indet. eating *Microgramma percussa*, 11.i.2011, D.Martinez, 11-SRNP-70083, sibling of wasp DHJPAR0042127 (1♀, in INBio); **Calma:** ex *Argyrosticta bellinita* eating *Niphidium oblanceolatum*, 29.i.2010, M. Rios, 10-SRNP-70576, sibling of wasp DHJPAR0039218 (6♀, in CNC, USNM); ex *Lophomyra tacita* eating *Microgramma percussa*, 26.xi.2010, R.Calero, 10-SRNP-73315, sibling of wasp DHJPAR0041745 (1♀ 1♂, in BMNH); **Medrano:** ex *Argyrosticta bellinitaDHJ01* eating *Polypodium fraxinifolium*, 23.vi.2010, R. Calero, 10-SRNP-71904, sibling of wasp DHJPAR0040318 (8♀ 3♂, in CNC, INBio, USNM); **Puntarenas:** Monteverde, 1000–1350 m, 3–31.i.1993, Z. Fuentes, malaise trap, LN 250850/449250, #2584 (1♀, in INBio).

##### Diagnosis.

**Figures 257–267.**
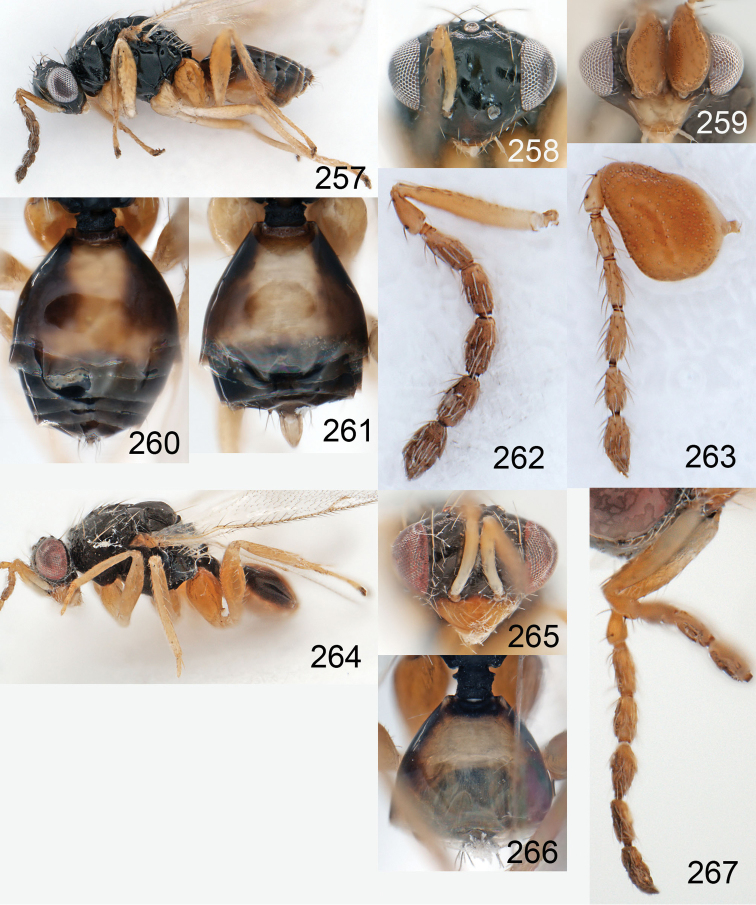
*Euplectrus* spp. **257–263** *Euplectrus eowilsoni* (DHJPAR0028932): **257** habitus in lateral view, female **258** head in frontal view, female **259** head in frontal view, male **260** gaster in dorsal view, female **261** gaster in dorsal view, male **262** antenna in lateral view, female **263** antenna in lateral view, male **264–267** *Euplectrus markshawi* (DHJPAR0028896), female: **264** habitus in lateral view **265** head in frontal view **266** gaster in dorsal view **267** antenna in lateral view.

Female with lower face black with a small very dark reddish-brown spot medially, undelimited from surrounding parts of frons (Fig. 258), male with median part of lower face predominantly yellowish-brown, pale area reaching slightly outside of outer lateral margins of toruli (Fig. 259); legs yellowish-brown to yellowish-white (Fig. 257); petiole 0.5× as long as wide; female gaster with 1^st^ tergite dark brown with a yellowish-brown and inverted “T” medially (Fig. 260), male gaster with 1st tergite white with wide dark brown lateral margins and remaining tergites dark brown (Fig. 261); male antenna with scape with outer lateral surface yellowish-white and inner lateral surface pale brown, strongly swollen (Fig. 263), 1.4× as long as wide, with numerous sensory pores scattered all over the inner lateral surface.

##### Description.

**Figures 268–273.**
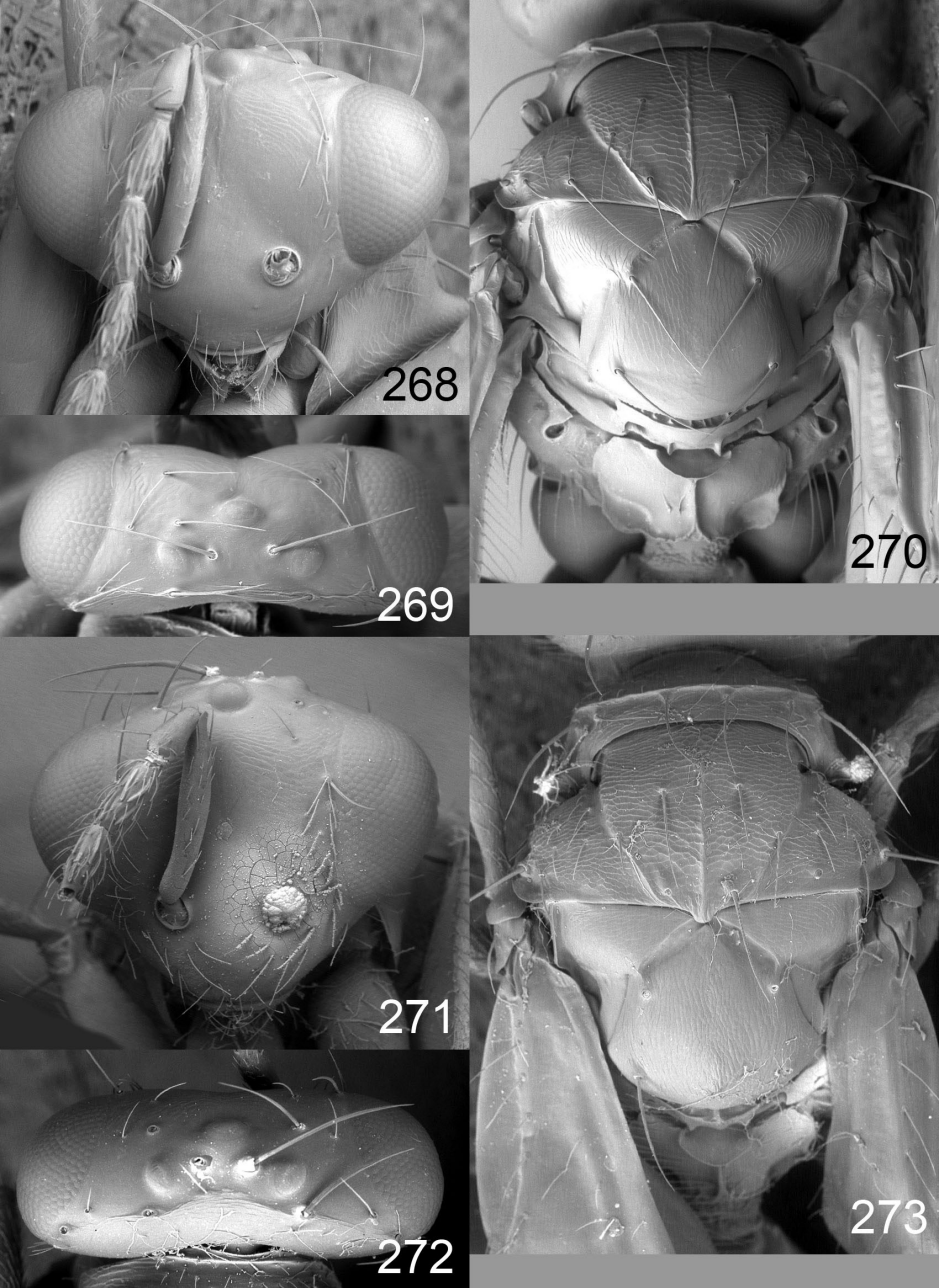
*Euplectrus* spp., females. **268–270** *Euplectrus eowilsoni* (DHJPAR0028932): **268** head in frontal view **269** vertex **270** mesosoma in dorsal view; **271–273** *Euplectrus markshawi* (DHJPAR0028896): **271** head in frontal view **272** vertex **273** mesosoma in dorsal view.

*Female*. Length of body 2.1 mm. Antenna with scape and pedicel yellowish-brown, flagellum dark brown with ventral part of flagellomeres 1–3 yellowish-brown (Fig. 262). Mandibles and palpi yellowish-white. Head black and shiny, lower face black with a small very dark reddish-brown spot medially, that is undelimited from surrounding parts of frons (Fig. 258). Frons close to eyes with an irregular row of setae that is double in parts (Fig. 268). Vertex with very weak reticulation, areas lateral to posterior ocelli smooth (Fig. 269). Occipital margin with a carina behind ocellar triangle (Fig. 269).

Mesosoma black and shiny (Fig. 257). Mesoscutum with weak reticulation (Fig. 270). Scutellum 0.9× as long as wide; with very weak reticulation (Fig. 270). Dorsellum along anterior margin with a deep groove that is divided by longitudinal carinae (Fig. 753), groove medially 0.3× as long as length of dorsellum. Propodeum with very weak reticulation, smooth close to median carina (Fig. 753); anteromedially with a semicircular cup; propodeal callus with eight setae. Legs yellowish-brown with basal ⅓ of mid femur yellowish-white (Fig. 257). Fore wing: submarginal vein with four setae; costal cell with one complete row of setae on ventral surface, and margin with two setae close to marginal vein; with 14 admarginal setae in one row.

Gaster with 1^st^ tergite dark brown with a yellowish-brown inverted “T” medially, remaining tergites dark brown (Fig. 260).

Ratios. HE/MS/WM = 1.9/1.0/1.3; POL/OOL/POO = 8.4/4.1/1.0; OOL/DO = 1.3; WE/WF/WH/HH = 1.0/2.0/4.3/3.0; WH/WT = 1.0; PM/ST = 1.7; TS1/TS2/LT/LT1/LT2/LT3/LT4 = 3.8/2.8/7.0/2.5/1.6/1.0/1.8; LP/WP = 0.5; MM/LG = 1.0.

*Male*. Length of body 1.7 mm. Scape with outer lateral surface yellowish-white and inner lateral surface pale brown, strongly swollen (Fig. 263); with entire inner lateral surface with sensory pores. Similar to female except antenna with flagellomeres 1–2 yellowish-brown and 3–6 pale brown (Fig. 263), lower face with median part of lower face predominantly yellowish-brown, pale area reaching slightly outside of outer lateral margins of toruli, and with lower face drawn out and pointed (Fig. 259), fore and mid legs predominantly yellowish-white, gaster shorter, 1st tergite white with wide dark brown lateral margins and remaining tergites dark brown (Fig. 261).

Ratios. LC/WS = 1.4; MM/LG = 1.3.

##### Hosts and biology.

Feeding on last instar larvae of *Argyrosticta bellinita* feeding on *Campyloneurum brevifolium*, *Campyloneurum angustifolium*, *Microgramma percussa*, *Microgramma 22028* and *Niphidium oblanceolatum*; *Argyrosticta bellinitaDHJ01* feeding on *Polypodium fraxinifolium*; *Argyrosticta vauaurea* feeding on *Phlebodium pseudoaureum*; *Argyrosticta aurifundensDHJ02* feeding on *Polypodium fraxinifolium*; *Lophomyra tacita* feeding on *Microgramma percussa*; *Lophomyra tacitaDHJ02* feeding on *Microgramma percussa*; Noctuidae indet. feeding on *Campyloneurum angustifolium*. All hosts are Noctuidae feeding on different species of Polypodiaceae. Parasitoid cocoons stuck to dead larva and substrate.

##### Distribution.

Costa Rica (Alajuela, Guanacaste and Puntarenas Provinces).

##### Etymology.

This species is named after Ed O. Wilson, in recognition of his contribution to the understanding of ACG Hymenoptera taxonomy.

#### Euplectrus garygibsoni

Taxon classificationAnimaliaHymenopteraEulophidae

Hansson sp. n.

http://zoobank.org/5CEF5313-9A33-41F9-8787-453994D48DCF

Figures 162–165 , 169–171 , 424 , 754

##### Material.

Holotype a female labeled “COSTA RICA: Guanacaste, ACG, Sector Pitilla, Medrano, 1.ix.2010, R. Calero, ex *Oxidercia thaumantis* eating *Machaerium salvadorense*, sibling of wasp DHJPAR0042124, 10-SRNP-72753” (BMNH). PARATYPES: 5♀: 1♀ with same label data as holotype (INBio), 4♀ with same data as holotype, but sibling of wasp DHJPAR0041821, 10-SRNP-72754 (CNC, INBio, USNM).

##### Diagnosis.

Lower face with median part dark reddish-brown, pale part reaching to level of median toruli (Fig. 163); legs yellowish-brown (Fig. 162); fore femur enlarged, 2.6× as long as wide (Fig. 162); hind tarsus laterally flattened with tarsomere 1 being the shortest (Fig. 424); petiole 1.0× as long as wide; gaster with anterior ½ yellowish-brown with dark brown lateral margins, posterior ½ dark brown (Fig. 164).

##### Description.

*Female*. Length of body 2.7 mm. Antenna with scape, pedicel and 1^st^ flagellomere yellowish-brown, flagellomeres 2–3 with basal ½ yellowish-brown and apical ½ dark brown, flagellomeres 4–6 dark brown (Fig. 165). Mandibles and palpi yellowish-brown. Head black and shiny, lower face with median part dark reddish-brown, pale part reaching to level of median toruli, with parts between pale area and eyes black (Fig. 163). Frons close to eyes with two irregular rows of setae (Fig. 169). Vertex with very weak reticulation (Fig. 170). Occipital margin with a carina behind ocellar triangle (Fig. 170).

Mesosoma black and shiny (Fig. 162). Each sidelobe of mesoscutum with 12 setae. Scutellum 0.8× as long as wide; with very weak reticulation (Fig. 171). Dorsellum along anterior margin with a deep groove that is divided by longitudinal carinae (Fig. 754), groove medially 0.4× as long as length of dorsellum. Propodeum with very weak reticulation (Fig. 754); anteromedially with a semicircular cup; propodeal callus with eight setae. Legs yellowish-brown (Fig. 162). Fore wing: costal cell on ventral surface with two complete rows of setae, three rows in some places, and margin with six setae close to marginal vein; with 22 admarginal setae, mainly in one row but with two rows in apical ⅓.

Gaster with anterior ½ yellowish-brown with dark brown lateral margins, posterior ½ dark brown (Fig. 164).

Ratios. HE/MS/WM = 2.1/1.0/1.1; POL/OOL/POO = 8.0/3.0/1.0; OOL/DO = 0.8; WE/WF/WH/HH = 1.0/2.2/4.5/3.3; WH/WT = 0.9; PM/ST = 1.9; TS1/TS2/LT/LT1/LT2/LT3/LT4 = 4.8/2.8/7.8/1.0/1.5/1.5/3.5; LP/WP = 1.0; MM/LG = 1.0.

*Male*. Unknown.

##### Hosts and biology.

Feeding on last instar larva of *Oxidercia thaumantis* (Erebidae) feeding on *Machaerium salvadorense* (Fabaceae), parasitoid cocoons stuck to dead larva and substrate.

##### Distribution.

Costa Rica (Guanacaste Province).

##### Etymology.

This species is after Gary A. P. Gibson, in recognition of his contribution to the understanding of ACG Hymenoptera taxonomy.

#### Euplectrus gavinbroadi

Taxon classificationAnimaliaHymenopteraEulophidae

Hansson sp. n.

http://zoobank.org/EFA36F98-B900-4728-B2D7-2B0BF69008A1

Figures 179–182 , 186–188 , 755

##### Material.

Holotype a female labeled “COSTA RICA: Guanacaste, ACG, Sector Pitilla, Estación Pitilla, 8.viii.2011, C. Moraga, ex *Antiblemma* Poole22 eating *Henriettea tuberculosa*, sibling of wasp DHJPAR0045450, 11-SRNP-32227 (BMNH). PARATYPES: 3♀ with same label data as holotype (BMNH, INBio).

##### Diagnosis.

Lower face with median part reddish-brown, pale area reaching to level of median toruli (Fig. 180); dorsellum without groove or foveae along anterior margin (Fig. 755); fore and mid coxae white, remaining parts of fore and mid legs predominantly yellowish-white, hind leg yellowish-white (Fig. 179); petiole 1.3× as long as wide; gaster with anterior ½ yellowish-white with dark brown lateral margins, posterior ½ dark brown (Fig. 181).

##### Description.

*Female*. Length of body 2.0 mm. Antenna with scape, pedicel and 1^st^ flagellomere yellowish-brown, 2 pale brown, 3–6 dark brown (Fig. 182). Mandibles and palpi yellowish-white. Head black and shiny, lower face with median part reddish-brown, pale area reaching to level of median toruli, with parts between pale area and eyes black (Fig. 180). Frons close to eyes with one sparse row of setae (Fig. 186). Vertex with very weak reticulation (Fig. 187). Occipital margin with a carina behind ocellar triangle (Fig. 187).

Mesosoma black and shiny (Fig. 179). Each sidelobe of mesoscutum with eight setae. Scutellum 1.0× as long as wide, with very weak reticulation (Fig. 188). Dorsellum without groove or foveae along anterior margin (Fig. 755). Propodeum with very weak reticulation (Fig. 755); anteromedially with a semicircular cup; propodeal callus with eight setae. Legs (Fig. 179): fore and mid coxae white, remaining parts of fore and mid legs predominantly yellowish-white; hind leg yellowish-white. Fore wing: costal cell with two complete rows of setae on ventral surface, and margin with seven setae in apical ½; with 22 admarginal setae, mainly in one row but with three rows in apical ⅓.

Gaster with anterior ½ yellowish-white with dark brown lateral margins, posterior ½ dark brown (Fig. 181).

Ratios. HE/MS/WM = 2.6/1.0/1.5; POL/OOL/POO = 4.0/2.1/1.0; OOL/DO = 1.1; WE/WF/WH/HH = 1.0/2.1/4.3/3.4; WH/WT = 1.1; PM/ST = 1.5; TS1/TS2/LT/LT1/LT2/LT3/LT4 = 3.8/2.4/6.7/1.6/1.3/1.0/2.2; LP/WP = 1.3; MM/LG = 1.0.

*Male*. Unknown.

##### Hosts and biology.

Feeding on last instar larva of *Antiblemma* Poole22 (Erebidae) feeding on *Henriettea tuberculosa* (Melastomataceae), parasitoid cocoons stuck to dead larva and substrate.

##### Distribution.

Costa Rica (Guanacaste Province).

##### Etymology.

This species is named after Gavin R. Broad, in recognition of his contribution to the understanding of ACG Hymenoptera taxonomy.

#### Euplectrus gerarddelvarei

Taxon classificationAnimaliaHymenopteraEulophidae

Hansson sp. n.

http://zoobank.org/21E33C17-0751-4A10-83F9-D1BE3FF4CBEB

Figures 196–199 , 203–205 , 756

##### Material.

Holotype a female labeled “COSTA RICA: Guanacaste, ACG, Sector Pitilla, Quebradona, 11.i.2011, D. Martinez, ex *Erinnyis alope* eating *Carica papaya*, sibling of wasp DHJPAR0042126, 11-SRNP-70078” (BMNH).

##### Diagnosis.

Lower face pale, medially yellowish-brown and laterally yellowish-white, with a narrow black stripe along eye margin (Fig. 197); fore and mid legs yellowish-white, hind leg yellowish-brown (Fig. 196); petiole 0.7× as long as wide; gaster with anterior ½ yellowish-brown with dark brown lateral margins, posterior ½ dark brown (Fig. 198).

##### Description.

*Female*. Length of body 2.5 mm. Antenna with scape white, pedicel yellowish-brown, flagellomeres 1–3 dark brown with ventral part yellowish-brown, 4–6 dark brown (Fig. 199). Mandibles and palpi yellowish-white. Head black and shiny, lower face pale, medially yellowish-brown and laterally yellowish-white, with a narrow black stripe along eye margin (Fig. 197). Frons close to eyes with two rows of setae (Fig. 203). Vertex smooth (Fig. 204). Occipital margin rounded (Fig. 204).

Mesosoma black and shiny (Fig. 196). Each sidelobe of mesoscutum with 18 setae. Scutellum 0.9× as long as wide; with very weak reticulation (Fig. 205). Dorsellum along anterior margin with a narrow groove that is divided by longitudinal carinae (Fig. 756), groove medially 0.2× as long as length of dorsellum. Propodeum smooth (Fig. 756); anteromedially with a semicircular cup; propodeal callus with ten setae. Fore and mid legs yellowish-white, hind leg yellowish-brown (Fig. 196). Fore wing: submarginal vein with six setae; costal cell with two rows of setae on ventral surface, and margin with three setae close to marginal vein; with 27 admarginal setae, in two irregular rows.

Gaster with anterior ½ yellowish-brown with dark brown lateral margins, posterior ½ dark brown (Fig. 198).

Ratios. HE/MS/WM = 2.0/1.0/1.2; POL/OOL/POO = 9.8/5.8/1.0; OOL/DO = 1.3; WE/WF/WH/HH = 1.0/2.4/4.5/3.3; WH/WT = 1.1; PM/ST = 1.9; TS1/TS2/LT/LT1/LT2/LT3/LT4 = nm; LP/WP = 0.7; MM/LG = 1.0.

*Male*. Unknown.

##### Hosts and biology.

Feeding on intermediate instar larva of *Erinnyis alope* (Sphingidae) feeding on *Carica papaya* (Caricaceae), parasitoid cocoons stuck to cuticle of host larva.

##### Distribution.

Costa Rica (Guanacaste Province).

##### Etymology.

This species is named after Gerard Delvare, in recognition of his contribution to the understanding of ACG Hymenoptera taxonomy.

#### Euplectrus henrytownesi

Taxon classificationAnimaliaHymenopteraEulophidae

Hansson sp. n.

http://zoobank.org/5B2A396E-53A6-4FEF-9124-8D0BC0F79301

Figures 274–280 , 285–287 , 757

##### Material.

Holotype a female labeled “COSTA RICA: Guanacaste, ACG, Sector Pitilla, Pasmompa, 18.ix.2005, M. Rios, ex *Thysanopyga cermala* eating *Gouania polygama*, sibling of wasp DHJPAR0028805, 05-SRNP-33942” (BMNH). PARATYPES: 11♀ 3♂: 5♀ 1♂ with same label data as holotype (BMNH, INBio, MZLU); following from same locality and host as holotype but collected 18.ix.2005, sibling of wasp DHJPAR0028810, 05-SRNP-33946 (2♀ 1♂, in CNC), 12.ix.2006, sibling of wasp DHJPAR0028862, 06-SRNP-34122 (4♀ 1♂, in BMNH, INBio, USNM).

##### Diagnosis.

**Figures 274–284.**
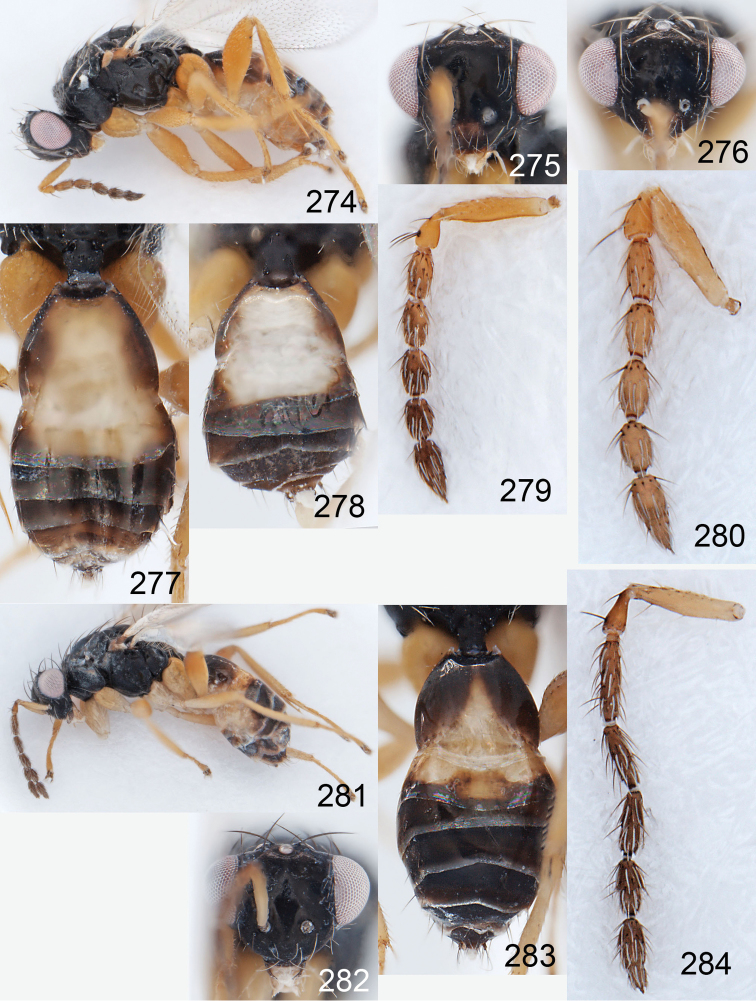
*Euplectrus* spp. **274–280** *Euplectrus henrytownesi* (DHJPAR0028805): **274** habitus in lateral view, female **275** head in frontal view, female **276** head in frontal view, male **277** gaster in dorsal view, female **278** gaster in dorsal view, male **279** antenna in lateral view, female **280** antenna in lateral view, male; **281–284** *Euplectrus ninazitaniae* (DHJPAR0028735), female: **281** habitus in lateral view **282** head in frontal view **283** gaster in dorsal view **284** antenna in lateral view.

Lower face with median part dark reddish-brown, pale part reaching only to inner lateral margins of toruli (Figs 275, 276); legs yellowish-brown (Fig. 274); petiole 1.0× as long as wide; gaster dark brown, anterior ½ with a large yellowish-brown (female, Fig. 277) or white (male, Fig. 278) spot, female with apex of gaster pale brown, in male dark brown; male antenna with scape expanded and widest slightly above the middle, 3.5× as long as wide (Fig. 280), flagellomeres 2–5 with a basal whorl of erect setae.

##### Description.

**Figures 285–290.**
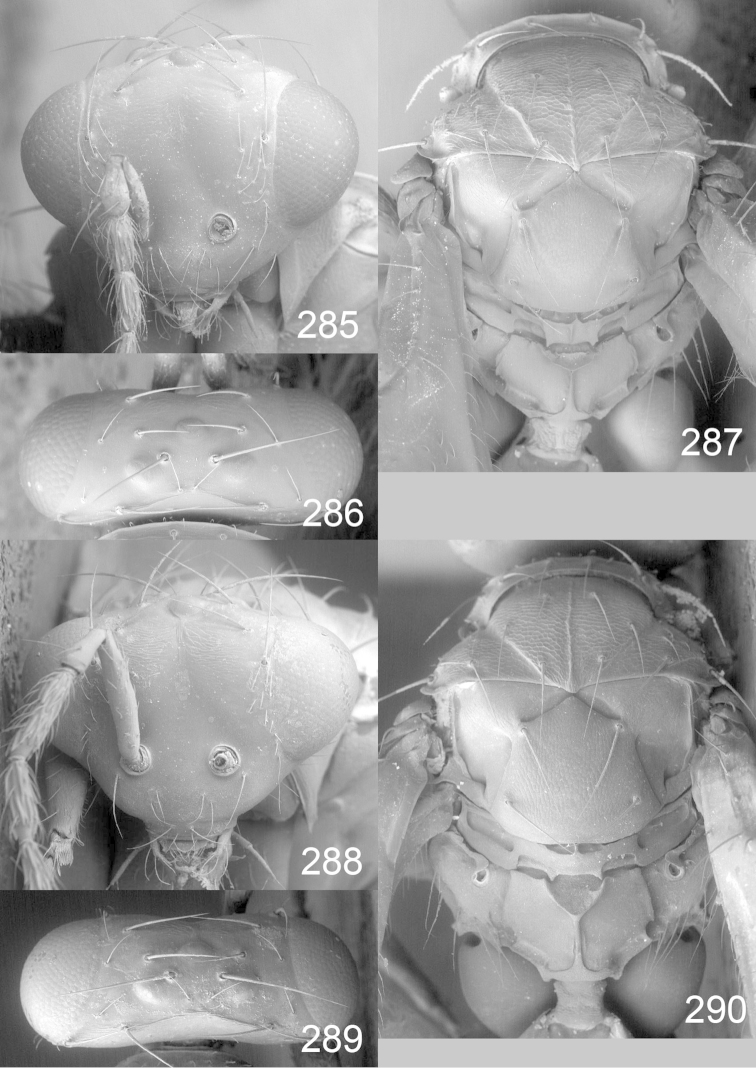
*Euplectrus* spp. **285–287** *Euplectrus henrytownesi* (DHJPAR0028805), female: **285** head in frontal view **286** vertex **287** mesosoma in dorsal view; **288–290** *Euplectrus ninazitaniae* (DHJPAR0028735), female: **288** head in frontal view **289** vertex **290** mesosoma in dorsal view.

*Female*. Length of body 2.5 mm. Antenna with scape and pedicel yellowish-brown, flagellomere 1 pale brown, 2–6 dark brown (Fig. 279). Mandibles and palpi yellowish-white. Head black and shiny, lower face with median part dark reddish-brown, pale part reaching only to inner lateral margins of toruli, with parts between pale area and eyes black (Fig. 275). Frons close to eyes with three rows of setae (Fig. 285). Vertex smooth (Fig. 286). Occipital margin with a carina behind ocellar triangle (Fig. 286).

Mesosoma black and shiny (Fig. 274). Each sidelobe of mesoscutum with 13 setae. Scutellum 1.0× as long as wide; with weak engraved reticulation (Fig. 287). Dorsellum along anterior margin with a wide groove that is divided by longitudinal carinae (Fig. 757), groove medially 0.4× as long as length of dorsellum. Propodeum with very weak reticulation (Fig. 757); anteromedially with a semicircular cup that has posterior part strongly raised and distinctly higher than anterior part; propodeal callus with eight setae. Legs yellowish-brown (Fig. 274). Fore wing: costal cell with two irregular rows of setae on ventral surface, and margin with eight setae in apical ½; with 16 admarginal setae, in one row.

Gaster dark brown with apex pale brown, anterior ½ with a large yellowish-brown spot (Fig. 277).

Ratios. HE/MS/WM = 1.9/1.0/1.1; POL/OOL/POO = 4.0/2.4/1.0; OOL/DO = 1.5; WE/WF/WH/HH = 1.0/2.8/5.0/3.8; WH/WT = 1.1; PM/ST = 1.5; TS1/TS2/LT/LT1/LT2/LT3/LT4 = 4.0/2.4/5.8/2.0/1.2/1.0/1.8; LP/WP = 1.0; MM/LG = 1.0.

*Male*. Length of body 2.1 mm. Scape slightly expanded and widest slightly above the middle (Fig. 280), sensory pores confined to apicoventral ⅔, sensory area pale as scape. Otherwise similar to female except flagellomeres 2–5 with a basal whorl of erect setae (Fig. 280); gaster shorter, and with apex dark brown (Fig. 278).

Ratios. LC/WS = 3.5; MM/LG = 1.2.

##### Hosts and biology.

Feeding on last instar larva of *Thysanopyga cermala* (Geometridae) feeding on *Gouania polygama* (Rhamnaceae), parasitoid cocoons stuck to cuticle of host larva.

##### Distribution.

Costa Rica (Guanacaste Province).

##### Etymology.

This species is named after Henry K. Townes, in recognition of his contribution to the understanding of ACG Hymenoptera taxonomy.

#### Euplectrus howelldalyi

Taxon classificationAnimaliaHymenopteraEulophidae

Hansson sp. n.

http://zoobank.org/E1346EC9-E9F3-482B-AE20-D07800B876C6

Figures 213–216 , 220–222 , 758

##### Material.

Holotype a male labeled “COSTA RICA: Guanacaste, ACG, Sector Cacao, Quebrada Otilio, D. Garcia, ex *Hemicephalis* alesaDHJ01 eating *Varronia inermis*, sibling of wasp DHJPAR0028882, 06-SRNP-45315” (BMNH). PARATYPES: 14♂ with same label data as holotype (BMNH, CNC, INBio, MZLU, MIUCR, USNM).

##### Diagnosis.

Lower face predominantly white, pale area reaches almost to eyes (Fig. 214); scape slightly expanded and widest in apical part, 3.4× as long as wide (Fig. 216), sensory pores confined to apico-ventral part, this area is yellowish-brown; scutellum convex with very strong engraved reticulation (Fig. 222); legs yellowish-white (Fig. 213); petiole 0.8× as long as wide; gaster with anterior ⅔ white with anterolateral margins dark brown, margin broken medially by white stripe, posterior ⅓ dark brown (Fig. 215). With same host as *Euplectrus donquickei*, but male with less pointy lower face in *Euplectrus howelldalyi*.

##### Description.

*Male*. Length of body 2.0 mm. Antenna with scape yellowish-white with apex yellowish-brown, pedicel and flagellomeres 1–4 yellowish-brown, clava pale brown; scape slightly expanded and widest in apical part (Fig. 216), sensory pores confined to apico-ventral part, this area is yellowish-brown. Mandibles and palpi white. Head black and shiny, lower face predominantly white, (Fig. 214), pale area reaches almost to eyes. Frons close to eyes with one row of setae (Fig. 220). Vertex smooth (Fig. 221). Occipital margin with a strong carina behind ocellar triangle (Fig. 221).

Mesosoma black and shiny (Fig. 213). Each sidelobe of mesoscutum with 13 setae. Scutellum 1.0× as long as wide; with strong engraved reticulation (Fig. 222). Dorsellum anteriorly with a narrow groove, medially 0.3× as long as length of dorsellum (Fig. 758). Propodeum smooth (Fig. 758); anteromedially with a semicircular cup; propodeal callus with seven setae. Legs yellowish-white (Fig. 213). Fore wing: costal cell with two rows of setae on ventral surface, and margin with four setae close to marginal vein; with 19 admarginal setae, in one row.

Gaster with anterior ⅔ white with anterolateral margins dark brown, margin broken medially by white stripe, posterior ⅓ dark brown (Fig. 215).

Ratios. HE/MS/WM = 2.1/1.0/1.3; POL/OOL/POO = 5.7/2.3/1.0; OOL/DO = 1.1; WE/WF/WH/HH = 1.0/1.9/3.8/2.7; LC/WS = 3.4; WH/WT = 1.2; PM/ST = 1.4; TS1/TS2/LT/LT1/LT2/LT3/LT4 = 3.6/2.5/6.7/2.5/1.3/1.0/1.5; LP/WP = 0.8; MM/LG = 1.1.

*Female*. Unknown.

##### Hosts and biology.

Feeding on penultimate instar larva of *Hemicephalis* alesaDHJ01 (Noctuidae) feeding on *Varronia inermis* (Boraginaceae), parasitoid cocoons stuck to dead larva and substrate.

##### Distribution.

Costa Rica (Guanacaste Province).

##### Etymology.

This species is named after Howell V. Daly, in recognition of his contribution to the understanding of ACG Hymenoptera taxonomy.

#### Euplectrus hugokonsi

Taxon classificationAnimaliaHymenopteraEulophidae

Hansson sp. n.

http://zoobank.org/A790987D-B156-4C31-A8C7-070514AA63E8

Figures 24 , 291–297 , 302–304 , 759

##### Material.

Holotype a female labeled “COSTA RICA: Guanacaste, ACG, Sector Santa Rosa, Sendero Natural, 13.xi.1990, gusaneros, ex *Dasylophia guarana* eating *Platymiscium parviflorum*, no barcode, 90-SRNP-2035” (BMNH). PARATYPES: 2♀ 1♂ with same label data as holotype (BMNH, INBio).

##### Diagnosis.

**Figures 291–301.**
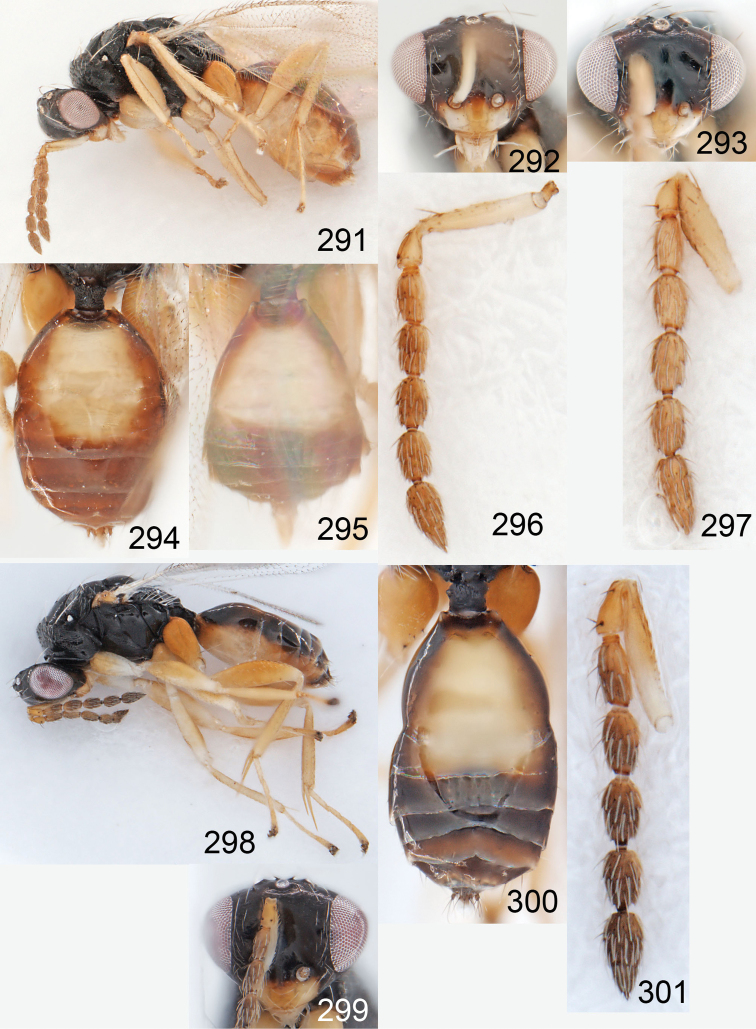
*Euplectrus* spp. **291–297** *Euplectrus hugokonsi* (90-SRNP-2035, no barcode): **291** habitus in lateral view, female **292** head in frontal view, female **293** head in frontal view, male **294** gaster in dorsal view, female **295** gaster in dorsal view, male **296** antenna in lateral view, female **297** antenna in lateral view, male; **298–301** *Euplectrus pammitchellae* (DHJPAR0038580), female: **298** habitus in lateral view **299** head in frontal view **300** gaster in dorsal view **301** antenna in lateral view.

Lower face with median part white laterally and yellowish-brown medially, pale area reaching outside of level of outer lateral margins of toruli, with a black area the width of 1.5× the width of scape between pale area and eye margin (Figs 292, 293); vertex with parts lateral to ocellar triangle dark reddish-brown; fore and mid legs yellowish-white, hind leg yellowish-brown (Fig. 291); dorsellum along anterior margin with a wide groove, medially 0.5× as long as length of dorsellum (Fig. 759); petiole 0.8× as long as wide; gaster dark brown, anterior ½ with a large yellowish-white (female, Fig. 294) or white (male, Fig. 295).

##### Description.

**Figures 302–307.**
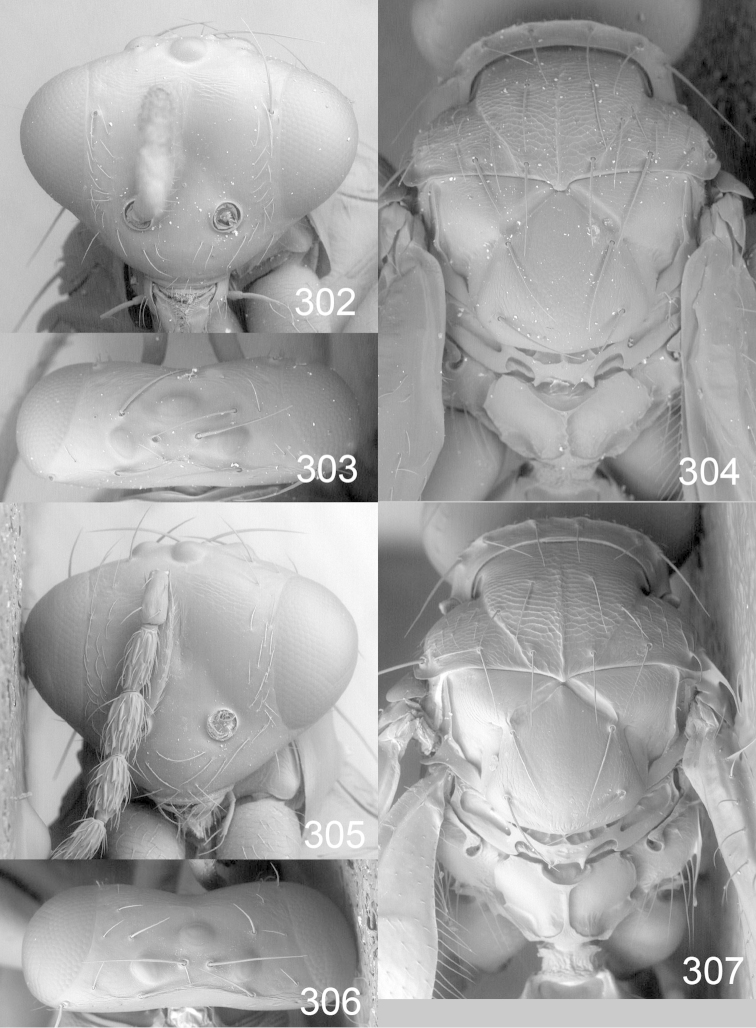
*Euplectrus* spp., females. **302–304** *Euplectrus hugokonsi* (90-SRNP-2035, no barcode): **302** head in frontal view **303** vertex **304** mesosoma in dorsal view; **305–307** *Euplectrus pammitchellae* (DHJPAR0038580): **305** head in frontal view **306** vertex **307** mesosoma in dorsal view.

*Female*. Length of body 2.6–2.7 mm. Antenna with scape yellowish-white, pedicel yellowish-brown, flagellomeres 1–4 pale brown with ventral part yellowish-brown, 5–6 pale brown (Fig. 296). Mandibles and palpi white. Head black and shiny, vertex with parts lateral to ocellar triangle dark reddish-brown, lower face with median part white laterally and yellowish-brown medially, pale area reaching outside of outer lateral margins of toruli, with a black area the width of 1.5× the width of scape between pale area and eye margin (Fig. 292). Frons close to eyes with two rows of setae (Fig. 302). Vertex with very weak reticulation inside ocellar triangle, outside ocellar triangle smooth (Fig. 303). Occipital margin with a weak carina (Fig. 303).

Mesosoma black and shiny (Fig. 291). Each sidelobe of mesoscutum with nine setae. Scutellum 1.0× as long as wide; with engraved weak reticulation (Fig. 304). Dorsellum with a wide groove along anterior margin, medially 0.4× as long as length of dorsellum (Fig. 759). Propodeum with weak reticulation (Fig. 759); anteromedially with a semicircular cup; propodeal callus with six setae. Legs (Fig. 291): fore and mid legs yellowish-white, hind leg yellowish-brown. Fore wing: submarginal vein with four setae; costal cell with two irregular and sparse rows of setae on ventral surface, and margin with two setae close to marginal vein; with 22 admarginal setae, in one row.

Gaster dark brown, anterior ½ with a large yellowish-white ovate spot (Fig. 294).

Ratios. HE/MS/WM = 2.4/1.3/1.0; POL/OOL/POO = 6.3/3.3/1.0; OOL/DO = 1.3; WE/WF/WH/HH = 1.0/2.6/4.7/3.5; WH/WT = 1.0; PM/ST = 1.4; TS1/TS2/LT/LT1/LT2/LT3/LT4 = 4.4/2.6/6.8/2.5/1.6/1.0/2.0; LP/WP = 0.8; MM/LG = 1.1.

*Male*. Length of body 1.9 mm. Scape slightly expanded and widest medially (Fig. 297), sensory pores confined to anteroventral ¾, sensory area pale brown. Otherwise similar to female except gaster with anterior ½ white with dark brown lateral margins (margins narrower than in female) (Fig. 295), and shorter.

Ratios. LC/WS = 3.3; MM/LG = 1.3.

##### Hosts and biology.

Feeding on intermediate instar larva of *Dasylophia guarana* (Notodontidae) feeding on *Platymiscium parviflorum* (Fabaceae), parasitoid cocoons stuck to dead larva and substrate.

##### Distribution.

Costa Rica (Guanacaste Province).

##### Etymology.

This species is named after Hugo Kons, in recognition of his contribution to the understanding of ACG Hymenoptera taxonomy.

#### Euplectrus iangauldi

Taxon classificationAnimaliaHymenopteraEulophidae

Hansson sp. n.

http://zoobank.org/749EEE1A-D5B5-4891-8DED-BD75BF0504B6

Figures 25 , 308–314 , 319–321 , 760

##### Material.

Holotype a female labeled “COSTA RICA: Guanacaste, ACG, Quebrada Otilio, 10.ix.2004, H. Ramirez, ex *Perigonia lusca* eating *Guettarda macrosperma*, sibling of wasp DHJPAR0028769, 04-SRNP-48716” (BMNH). Paratypes: 32♀ 15♂: COSTA RICA (ACG): **Guanacaste:** 10♀ 11♂ with same label data as holotype (BMNH, CNC, INBio, MZLU, USNM); **Sector del Oro, Uncaria:** ex *Perigonia lusca* eating *Uncaria tomentosa*, 16.vi.2004, C. Moraga, sibling of wasp DHJPAR0028740, 04-SRNP-22738 (11♀ 3♂, in BMNH, INBio, MIUCR); 24.viii.2006, R. Moraga, sibling of wasp DHJPAR0028888, 06-SRNP-22585 (11♀ 1♂, in CNC, INBio, USNM).

##### Diagnosis.

**Figures 308–318.**
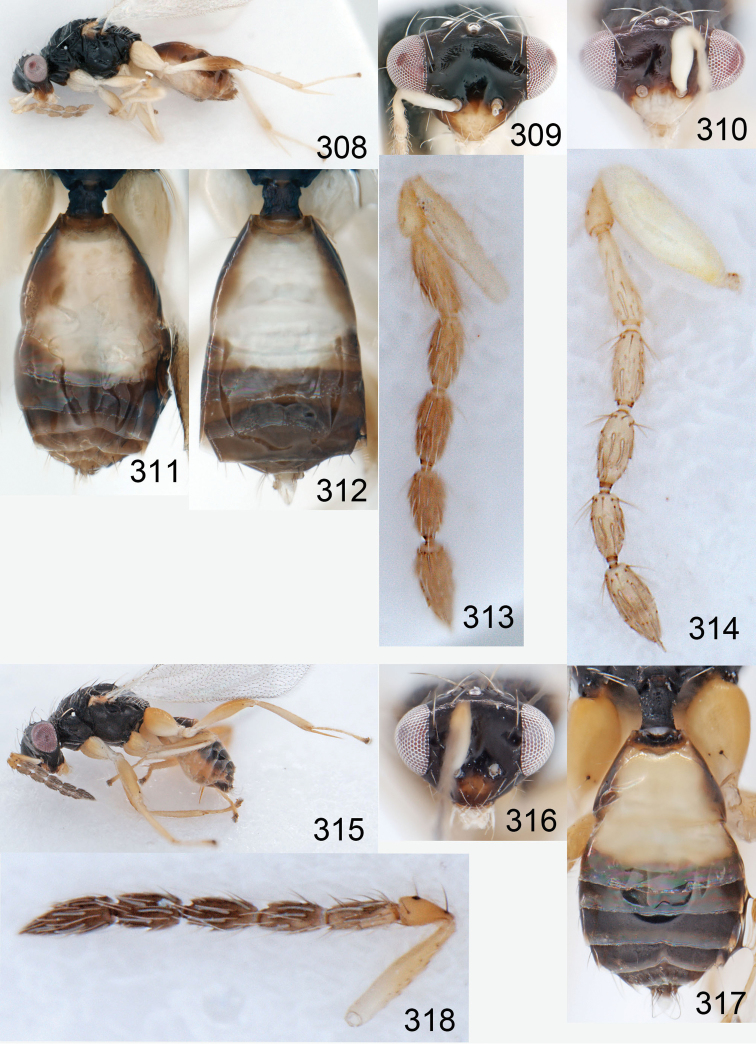
*Euplectrus* spp. **308–314** *Euplectrus iangauldi* (DHJPAR0028769): **308** habitus in lateral view, female **309** head in frontal view, female **310** head in frontal view, male **311** gaster in dorsal view, female **312** gaster in dorsal view, male **313** antenna in lateral view, female **314** antenna in lateral view, male **315–318** *Euplectrus ronaldzunigai* (DHJPAR0028824), female: **315** habitus in lateral view **316** head in frontal view **317** gaster in dorsal view **318** antenna in lateral view.

Head dark brown, lower face with median part yellowish-brown (female, Fig. 309), or white (male, Fig. 310), pale area reaching slightly outside lateral margin of toruli; dorsellum with two large foveae anteriorly (Fig. 760); fore coxa white, hind femur with apical ½ pale brown, remaining parts of legs yellowish-white (Fig. 308); petiole 1.0× as long as wide; gaster with anterior ½ white with dark brown lateral margins, posterior ½ dark brown (Figs 311, 312); male antenna with scape expanded, widest in the middle, and 2.7× as long as wide (Fig. 314), 1^st^ flagellomere 3.1× as long as wide, narrow at base and expanded in apical ⅓.

##### Description.

**Figures 319–324.**
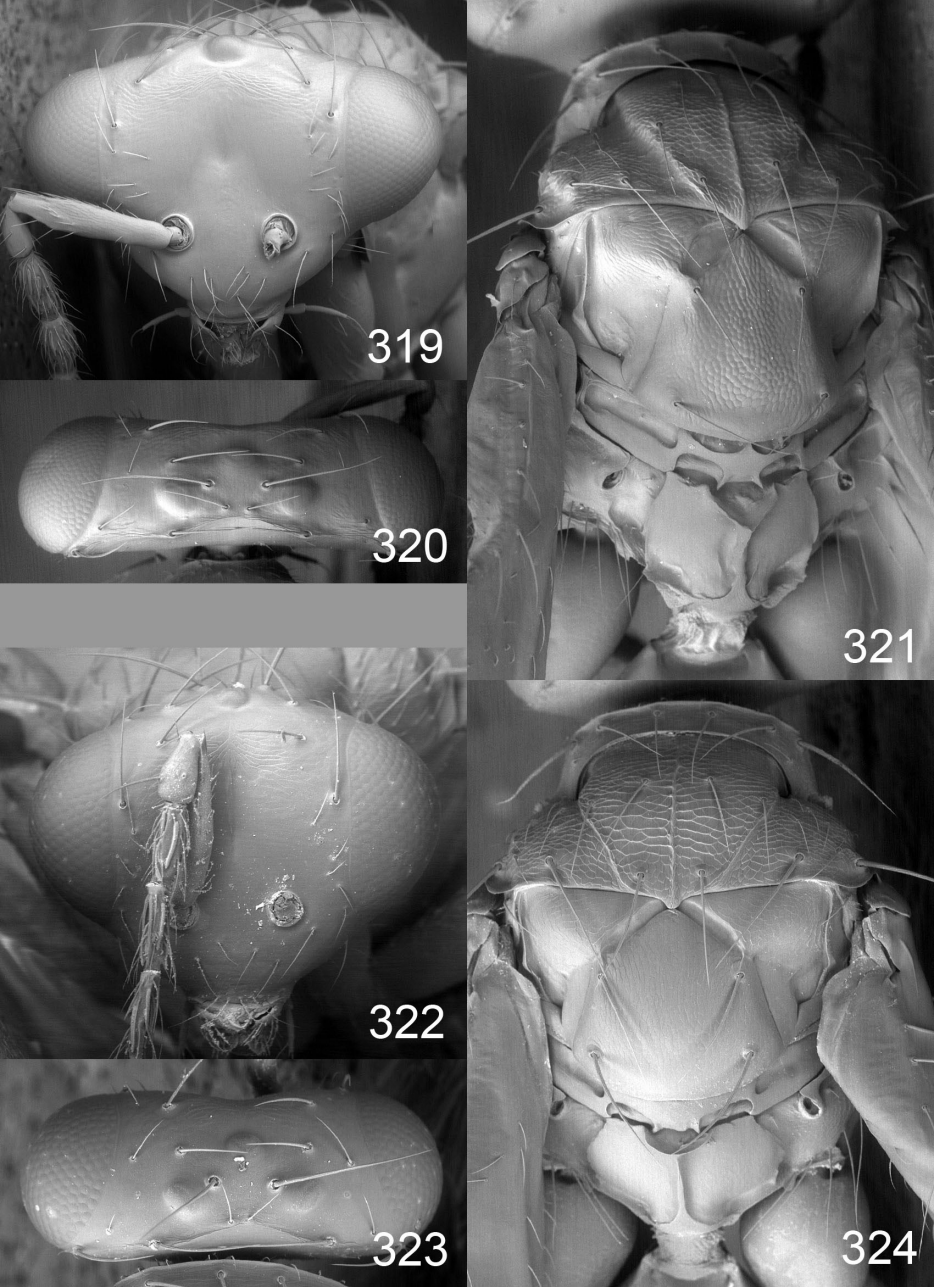
*Euplectrus* spp., females. **319–321** *Euplectrus iangauldi* (DHJPAR0028769): **319** head in frontal view **320** vertex **321** mesosoma in dorsal view; **322–324** *Euplectrus ronaldzunigai* (DHJPAR0028824): **322** head in frontal view **323** vertex **324** mesosoma in dorsal view.

*Female*. Length of body 2.7 mm. Antenna with scape white, pedicel yellowish-white, flagellomere 1 yellowish-brown, 2–6 pale brown (Fig. 313). Mandibles and palpi white. Head dark brown and shiny, lower face with median part yellowish-brown, pale area reaching slightly outside lateral margin of toruli, parts between pale area and eyes dark brown (Fig. 309). Frons close to eyes with two irregular rows of setae (Fig. 319). Vertex smooth (Fig. 320). Occipital margin with a carina behind ocellar triangle (Fig. 320).

Mesosoma black and shiny (Fig. 308). Each sidelobe of mesoscutum with 12 setae. Scutellum 1.0× as long as wide; with rather strong reticulation (Fig. 321). Dorsellum anteriorly with two large foveae (Fig. 760). Propodeum smooth (Fig. 760); anteromedially with a triangular cup; propodeal callus with nine setae. Legs (Fig. 308): fore coxa white, hind femur with apical ½ pale brown, remaining parts of legs yellowish-white. Fore wing: costal cell with two irregular rows of setae on ventral surface, and margin with three setae close to marginal vein; with 24 admarginal setae in irregular two rows.

Gaster with anterior ½ white with dark brown lateral margins, posterior ½ dark brown (Fig. 311).

Ratios. HE/MS/WM = 1.6/1.0/1.1; POL/OOL/POO = 7.4/4.4/1.0; OOL/DO = 1.2; WE/WF/WH/HH = 1.0/2.9/5.0/3.3; WH/WT = 1.2; PM/ST = 2.1; TS1/TS2/LT/LT1/LT2/LT3/LT4 = 4.7/2.9/7.6/2.4/1.9/1.0/1.9; LP/WP = 1.0; MM/LG = 1.1.

*Male*. Length of body 2.4 mm. Scape white, slightly expanded and widest in the middle (Fig. 314), sensory pores confined to apicoventral ⅔, sensory area white. Similar to female except antenna with 1^st^ flagellomere longer, 3.1× as long as wide, narrow at base and expanded in apical ⅓ (Fig. 314), lower face with pale area white (Fig. 310), gaster shorter.

Ratios. LC/WS = 2.7; MM/LG = 1.2.

##### Hosts and biology.

Feeding on last instar larva of *Perigonia lusca* (Sphingidae) feeding on *Guettarda macrosperma* and *Uncaria tomentosa* (Rubiaceae), parasitoid cocoons stuck to larva and leaf substrate.

##### Distribution.

Costa Rica (Guanacaste Province).

##### Etymology.

This species is named after Ian D. Gauld, in recognition of his contribution to the understanding of ACG Hymenoptera taxonomy.

#### Euplectrus jacklonginoi

Taxon classificationAnimaliaHymenopteraEulophidae

Hansson sp. n.

http://zoobank.org/0228F764-55AF-455F-9638-D2968B5CF33C

Figures 325–331 , 336–338 , 761

##### Material.

Holotype a female labeled “COSTA RICA: Guanacaste, ACG, Sector Pitilla, Estación Quica, 17.x.2009, C. Moraga et al., ex *Mursa* maricaDHJ01 eating *Panicum pilosum*, sibling of wasp DHJPAR0038559, 09-SRNP-73275” (BMNH). PARATYPES: 51♀ 11♂: COSTA RICA (ACG): **Guanacaste:** Sector Pitilla: 37♀ 7♂ from same locality, host and date as holotype (BMNH, CNC, INBio, MZLU, MIUCR, USNM); Medrano, 15.x.2012, R. Calero, ex *Mursa* maricaDHJ01 eating *Panicum pilosum*, sibling of wasp DHJPAR0051503, 12-SRNP-72275 (1♂, INBio), and sibling of wasp DHJPAR0051509, 12-SRNP-72279 (4♀, INBio), DHJPAR0051506, 12-SRNP-72280 (4♀ 1♂, INBio), DHJPAR0051508, 12-SRNP-72287 (6♀ 2♂, BMNH), DHJPAR0051507, 12-SRNP-72276 (1♀, INBio).

##### Diagnosis.

**Figures 325–335.**
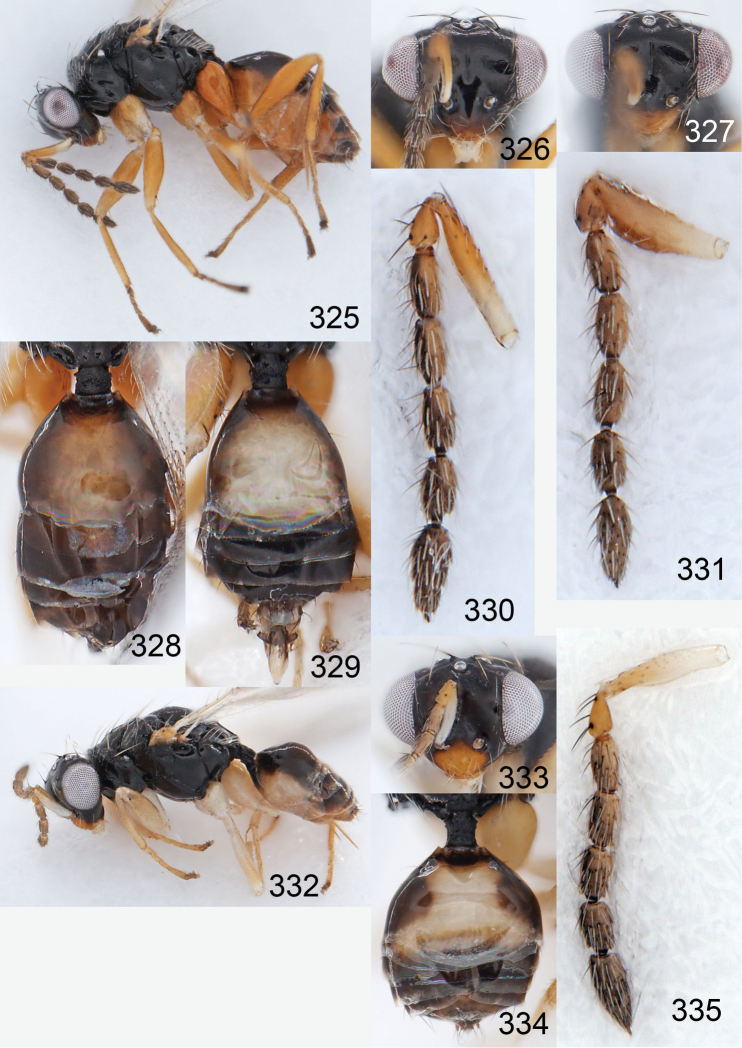
*Euplectrus* spp. **325–331** *Euplectrus jacklonginoi* (DHJPAR0038559): **325** habitus in lateral view, female **326** head in frontal view, female **327** head in frontal view, male **328** gaster in dorsal view, female **329** gaster in dorsal view, male **330** antenna in lateral view, female **331** antenna in lateral view, male; **332–335** *Euplectrus roysnellingi* (DHJPAR0028972), female: **332** habitus in lateral view **333** head in frontal view **334** gaster in dorsal view **335** antenna in lateral view.

Lower face medially dark reddish-brown and undelimited from surrounding parts of frons (female, Fig. 326) or yellowish-brown (male, Fig. 327), pale area reaching to level of outer lateral margins of toruli; scutellum smooth and shiny, laterally with very weak engraved reticulation (Fig. 338); legs yellowish-brown, female with tarsomere 4 on hind leg dark (Fig. 325); fore wing with short postmarginal vein, 1.2× as long as stigmal vein; petiole 0.7× as long as wide; female gaster dark brown, anterior ½ with a narrow pale brown spot (Fig. 328), male with pale spot white and wider (Fig. 329); male antenna with scape slightly expanded and widest in the middle, 3.1× as long as wide, with sensory area dark brown (Fig. 331).

##### Description.

**Figures 336–341.**
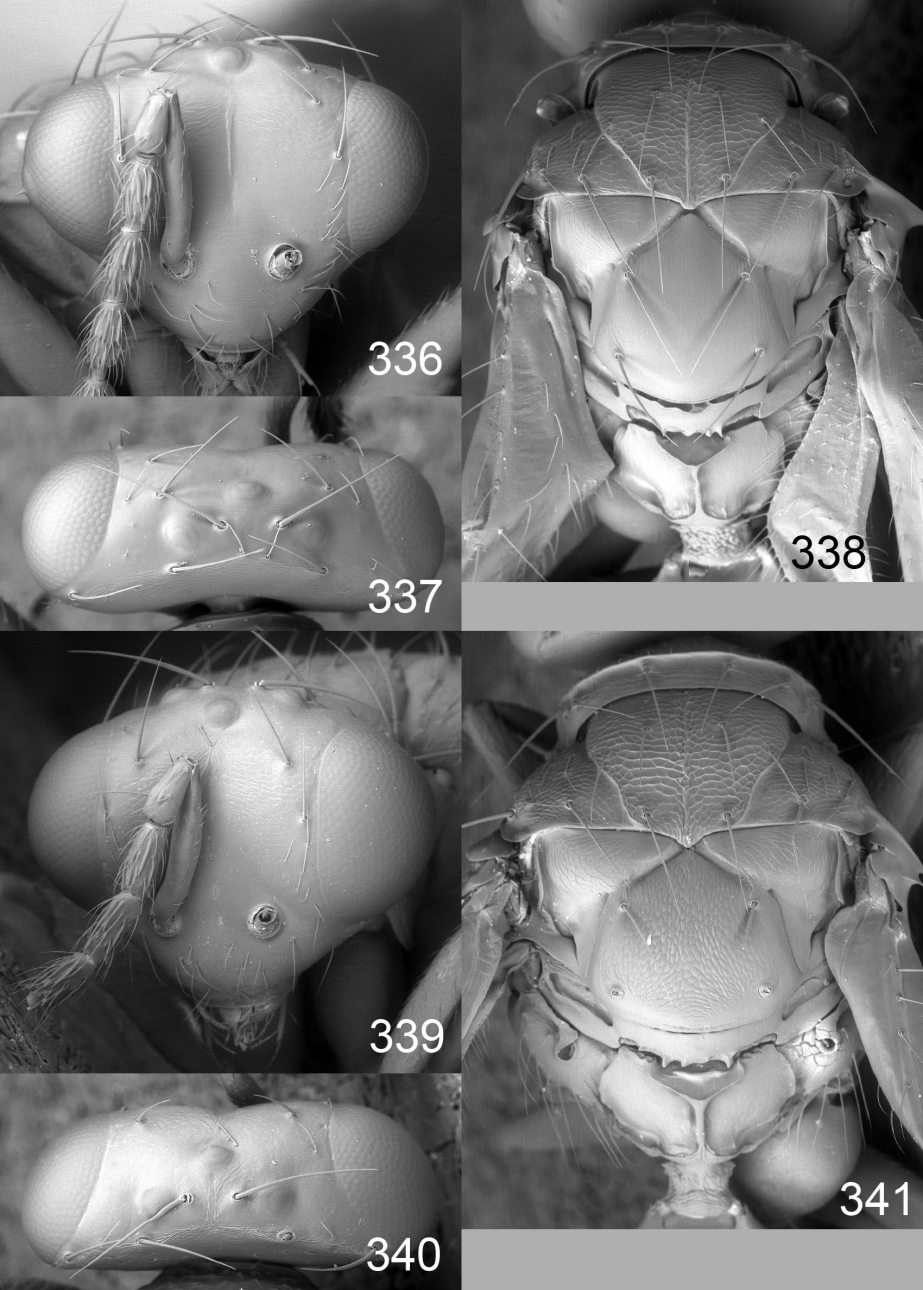
*Euplectrus* spp., females. **336–338** *Euplectrus jacklonginoi* (DHJPAR0038559): **336** head in frontal view **337** vertex **338** mesosoma in dorsal view; **339–341** *Euplectrus roysnellingi* (DHJPAR0028972): **339** head in frontal view **340** vertex **341** mesosoma in dorsal view.

*Female*. Length of body 2.0 mm. Antenna with scape and pedicel yellowish-brown, flagellomeres 1–2 dark brown dorsally and yellowish-brown ventrally, 3–6 dark brown (Fig. 330). Mandibles and palpi yellowish-white. Head black and shiny, lower face medially dark reddish-brown, pale area extending slightly outside level of lateral margins of toruli, with parts between pale area and eyes black (Fig. 326). Frons close to eyes with one row of setae (Fig. 336). Vertex smooth (Fig. 337). Occipital margin rounded (Fig. 337).

Mesosoma black and shiny (Fig. 325). Each sidelobe of mesoscutum with eight setae. Scutellum 1.0× as long as wide; smooth and shiny, laterally with very weak engraved reticulation (Fig. 338). Dorsellum along anterior margin with a narrow groove that is divided by longitudinal carinae (Fig. 761), groove medially 0.3× as long as length of dorsellum. Propodeum smooth (Fig. 761); anteromedially with a triangular cup; propodeal callus with ten setae. Legs yellowish-brown with tarsomeres 1–2 on all legs dark brown (Fig. 325). Fore wing: costal cell with one complete row of setae on ventral surface, and margin with three setae close to marginal vein; with 10 admarginal setae, in one row.

Gaster dark brown, anterior ½ with a narrow pale brown spot (Fig. 328).

Ratios. HE/MS/WM = 1.8/1.0/1.0; POL/OOL/POO = 5.7/3.1/1.0; OOL/DO = 1.4; WE/WF/WH/HH = 1.0/2.6/4.6/3.5; WH/WT = 1.1; PM/ST = 1.2; TS1/TS2/LT/LT1/LT2/LT3/LT4 = 4.3/2.9/7.4/3.0/1.6/1.0/1.9; LP/WP = 0.7; MM/LG = 1.0.

*Male*. Length of body 1.8 mm. Scape slightly expanded and widest above the middle, sensory pores confined to apicoventral ½, sensory area dark brown (Fig. 331). Otherwise similar to female except lower face with median part yellowish-brown (Fig. 327), gaster with pale spot white and wider (Fig. 329), and gaster shorter.

Ratios. LC/WS = 3.1; MM/LG = 1.1.

##### Hosts and biology.

Feeding on penultimate instar larva of *Mursa* maricaDHJ01 (Noctuidae) feeding on *Panicum pilosum* (Poaceae), parasitoid cocoons stuck to cuticle of host larva and leaf substrate.

##### Distribution.

Costa Rica (Guanacaste Province).

##### Etymology.

This species is named after Jack T. Longino, in recognition of his contribution to the understanding of ACG Hymenoptera taxonomy.

#### Euplectrus jesusugaldei

Taxon classificationAnimaliaHymenopteraEulophidae

Hansson sp. n.

http://zoobank.org/F425CCE0-118A-46F7-A148-52BF535203D2

Figures 230–233 , 237–239 , 762

##### Material.

Holotype a female labeled “COSTA RICA: Guanacaste, ACG, Sector Horizones, 28.ix.1997, gusaneros, ex *Tarachidia bicolorata* eating *Heliotropium indicum*, no barcode, 97-SRNP-9370” (BMNH). PARATYPES: 3♀ with same label data as holotype (BMNH, INBio). Note: all paratypes are broken.

##### Diagnosis.

Lower face with median part reddish-brown, pale area reaching slightly outside of outer lateral margins of toruli (Fig. 231); scutellum with very weak and engraved reticulation, hence shiny (Fig. 239); legs yellowish-brown (Fig. 230); petiole 0.7× as long as wide; gaster reddish-brown with dark brown lateral margins (Fig. 232).

##### Description.

*Female*. Length of body 2.0 mm. Antenna with scape and pedicel yellowish-brown, flagellomeres pale brown (Fig. 233). Mandibles and palpi white. Head black and shiny, lower face with median part reddish-brown, pale area reaching slightly outside of outer lateral margins of toruli, with parts between pale area and eyes black (Fig. 231). Frons close to eyes with one row of setae (Fig. 237). Vertex smooth outside ocellar triangle, with very weak reticulation inside ocellar triangle (Fig. 238). Occipital margin rounded (Fig. 238).

Mesosoma black and shiny (Fig. 230). Each sidelobe of mesoscutum with ten setae. Scutellum 1.0× as long as wide; with very weak engraved reticulation and hence shiny (Fig. 239). Dorsellum anteriorly with a narrow groove, medially 0.3× as long as length of dorsellum (Fig. 762). Propodeum smooth (Fig. 762); anteromedially with a semicircular cup; propodeal callus with 11 setae. Legs yellowish-brown (Fig. 230). Fore wing: submarginal vein with four setae; costal cell with one row of setae on ventral surface, and margin with three setae close to marginal vein; with 16 admarginal setae, in one row.

Gaster reddish-brown with dark brown lateral margins (Fig. 232).

Ratios. HE/MS/WM = 1.7/1.0/1.0; POL/OOL/POO = 5.8/3.3/1.0; OOL/DO = 1.6; WE/WF/WH/HH = 1.0/2.3/4.0/3.2; WH/WT = 1.0; PM/ST = 1.4; TS1/TS2/LT/LT1/LT2/LT3/LT4 = 4.0/2.5/6.9/2.4/1.5/1.0/1.8; LP/WP = 0.7; MM/LG = 1.3.

*Male*. Unknown.

##### Hosts and biology.

Feeding on penultimate instar larva of *Tarachidia bicolorata* (Noctuidae) feeding on *Heliotropium indicum* (Boraginaceae), parasitoid cocoons stuck to dead larva and substrate.

##### Distribution.

Costa Rica (Guanacaste Province).

##### Etymology.

This species is named after Jesus A. Ugalde-Gomez, in recognition of his contribution to the understanding of ACG Hymenoptera taxonomy.

#### Euplectrus jimwhitfieldi

Taxon classificationAnimaliaHymenopteraEulophidae

Hansson sp. n.

http://zoobank.org/0999A884-4333-4237-B291-BEFF9BE3E91A

Figures 342–348 , 353–355 , 763

##### Material.

Holotype a female labeled “COSTA RICA: Guanacaste, ACG, Sector Cacao, Sendero a Maritza, 19.viii.2010, M. Pereira, ex *Ctenoplusia oxygramma* eating *Baccharis trinervis*, sibling of wasp DHJPAR0042135, 10-SRNP-35841” (BMNH). PARATYPES: 7♀ 5♂: 6♀ 5♂ with same label data as holotype (BMNH, CNC, INBio, MZLU, USNM); 1♀ from Guanacaste, Estación Cacao, LN 323100/375800, 1000-1400 m, 1-9.ii.1996, malaise trap, A. Masis (INBio).

##### Diagnosis.

**Figures 342–352.**
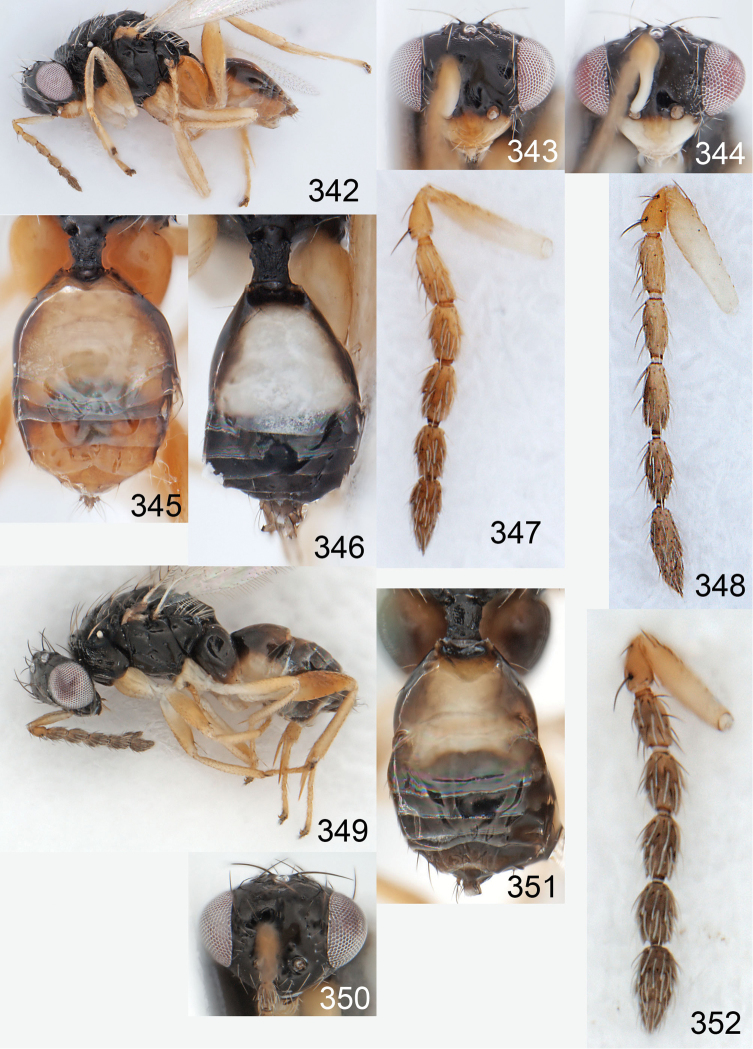
*Euplectrus* spp. **342–348** *Euplectrus jimwhitfieldi* (DHJPAR0042135): **342** habitus in lateral view, female **343** head in frontal view, female **344** head in frontal view, male **345** gaster in dorsal view, female **346** gaster in dorsal view, male **347** antenna in lateral view, female **348** antenna in lateral view, male; **349–352** *Euplectrus wonyoungchoi* (DHJPAR0048229), female: **349** habitus in lateral view **350** head in frontal view **351** gaster in dorsal view **352** antenna in lateral view.

Lower face medially yellowish-white with median part yellowish-brown, pale area reaching half-way between level of lateral margin of toruli and eyes (Figs 343, 344); scutellum with very weak engraved reticulation and with distinctly elongate meshes (Fig. 355); dorsellum along anterior margin with a very wide groove reaching ½ the length of dorsellum, groove is divided by longitudinal carinae, and with posterior margin of groove strongly protruding upwards (Fig. 763); legs yellowish-brown (Fig. 342); petiole 1.0× as long as wide; female gaster with anterior ½ yellowish-white with dark brown lateral margins, posterior ½ yellowish-brown with dark brown lateral margins, and with a dark brown median spot or band (Fig. 345), male gaster with anterior ½ white with dark brown lateral margins, posterior ½ dark brown (Fig. 346); male antenna with scape slightly expanded and widest above the middle, 3.5× as long as wide (Fig. 348).

##### Description.

**Figures 353–358.**
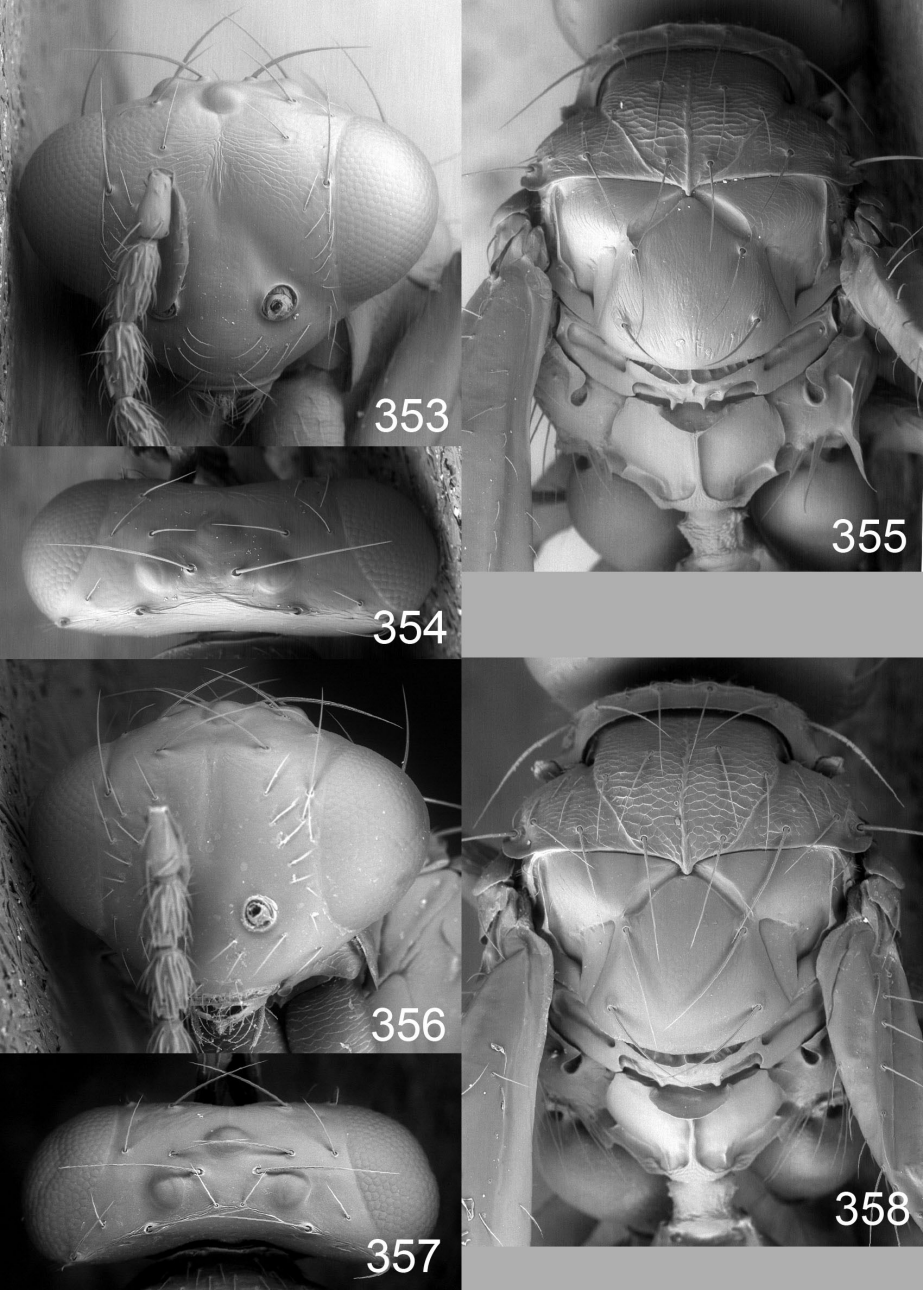
*Euplectrus* spp., females. **353–355** *Euplectrus jimwhitfieldi* (DHJPAR0042135): **353** head in frontal view **354** vertex **355** mesosoma in dorsal view; **356–358** *Euplectrus wonyoungchoi* (DHJPAR0048229): **356** head in frontal view **357** vertex **358** mesosoma in dorsal view.

*Female*. Length of body 2.3 mm. Antenna with scape and pedicel yellowish-brown, flagellomeres 1–2 yellowish-brown ventrally and dark brown dorsally, 3–6 dark brown (Fig. 347). Mandibles and palpi yellowish-white. Head black and shiny, lower face medially yellowish-white with median part yellowish-brown, pale area reaching half-way between level of lateral margin of toruli and eyes, part between pale area and eyes black (Fig. 343). Frons close to eyes with one row of setae, upper part of row moved away from eye margin (Fig. 353). Vertex smooth (Fig. 354). Occipital margin rounded (Fig. 354).

Mesosoma black and shiny (Fig. 342). Each sidelobe of mesoscutum with 10 setae. Scutellum 0.9× as long as wide; with very weak engraved reticulation and with distinctly elongate meshes (Fig. 355). Dorsellum along anterior margin with a very wide groove that is divided by longitudinal carinae, groove medially 0.5× as long as length of dorsellum, and with posterior margin of groove strongly protruding upwards (Fig. 763). Propodeum with very weak reticulation and partly smooth (Fig. 763); anteromedially with a transverse circular cup; propodeal callus with 12 setae. Legs yellowish-brown (Fig. 342). Fore wing: costal cell with one row of setae on ventral surface, and margin with four setae close to marginal vein; with 12 admarginal setae, in one row.

Gaster with anterior ½ yellowish-white with dark brown lateral margins, posterior ½ yellowish-brown with dark brown lateral margins, and with a dark brown median spot or band (Fig. 345).

Ratios. HE/MS/WM = 1.6/1.1/1.0; POL/OOL/POO = 5.0/3.3/1.0; OOL/DO = 1.5; WE/WF/WH/HH = 1.0/2.5/4.5/3.3; WH/WT = 1.1; PM/ST = 1.6; TS1/TS2/LT/LT1/LT2/LT3/LT4 = 3.7/2.5/6.7/2.7/1.4/1.0/1.5; LP/WP = 1.0; MM/LG = 1.1.

*Male*. Length of body 2.0 mm. Scape slightly expanded and widest above the middle (Fig. 348), sensory pores confined to apicoventral ½, sensory area pale as scape. Otherwise similar to female except gaster shorter and anterior ½ white with dark brown lateral margins, posterior ½ dark brown (Fig. 346).

Ratios. LC/WS = 3.5; MM/LG = 1.3.

##### Hosts and biology.

Feeding on penultimate instar larva of *Ctenoplusia oxygramma* (Noctuidae) feeding on *Baccharis trinervis* (Asteraceae), parasitoid cocoons stuck to dead larva and substrate.

##### Distribution.

Costa Rica (Guanacaste Province).

##### Etymology.

This species is named after Jim B. Whitfield, in recognition of his contribution to the understanding of ACG Hymenoptera taxonomy.

#### Euplectrus jjrodriguezae

Taxon classificationAnimaliaHymenopteraEulophidae

Hansson sp. n.

http://zoobank.org/773BABCF-CFA9-4C17-82A1-7578553A868F

Figures 247–250 , 254–256 , 764

##### Material.

Holotype a female labeled “COSTA RICA: Guanacaste, ACG, Sector Horizontes, La Dama, 2.ix.1994, gusaneros, ex *Ozarba geta* eating *Dyschoriste quadrangularis*, no barcode, 94-SRNP-7129” (BMNH). PARATYPES: 2♀ with same label data as holotype (INBio).

##### Diagnosis.

Lower face medially yellowish-brown, pale area extending slightly outside of outer lateral margin of toruli (Fig. 248); scutellum with very weak and engraved reticulation and smooth medially, hence shiny (Fig. 256); legs yellowish-brown (Fig. 247); petiole 0.9× as long as wide; gaster with anterior ½ yellowish-brown with dark brown lateral margins, posterior ½ brown with a dark brown round spot medially (Fig. 249).

##### Description.

*Female*. Length of body 2.1 mm. Antenna with scape yellowish-white, pedicel yellowish-brown, flagellomeres pale brown (Fig. 250). Mandibles and palpi white. Head black and shiny, lower face medially yellowish-brown, pale area extending slightly outside of outer lateral margin of toruli, parts between pale area and eyes black (Fig. 248). Frons close to eyes with one row of setae (Fig. 254). Vertex smooth outside ocellar triangle, with very weak reticulation inside ocellar triangle (Fig. 255). Occipital margin with a weak carina (Fig. 255).

Mesosoma black and shiny (Fig. 247). Each sidelobe of mesoscutum with seven setae. Scutellum 1.0× as long as wide; with very weak engraved reticulation, smooth medially, hence shiny (Fig. 256). Dorsellum with a groove in anterior part that is divided by longitudinal carinae (Fig. 764), groove medially 0.2× as long as length of dorsellum. Propodeum with very weak reticulation (Fig. 764); anteromedially with a semicircular cup; propodeal callus with 12 setae. Legs yellowish-brown (Fig. 247). Fore wing: costal cell with two rows of setae on ventral surface, and margin with two setae close to marginal vein; with 13 admarginal setae, in one row.

Gaster with anterior ½ yellowish-brown with dark brown lateral margins, posterior ½ brown with a dark brown round spot medially (Fig. 249).

Ratios. HE/MS/WM = 1.8/1.0/1.2; POL/OOL/POO = 5.3/2.8/1.0; OOL/DO = 1.4; WE/WF/WH/HH = 1.0/2.7/4.6/3.5; WH/WT = 1.0; PM/ST = 1.6; TS1/TS2/LT/LT1/LT2/LT3/LT4 = 3.3/1.8/6.0/1.7/1.3/1.0/1.8; LP/WP = 0.9; MM/LG = 1.1.

*Male*. Unknown.

##### Hosts and biology.

Feeding on last instar larva of *Ozarba geta* (Noctuidae) feeding on *Dyschoriste quadrangularis* (Acanthaceae), parasitoid cocoons stuck to dead larva and substrate.

##### Distribution.

Costa Rica (Guanacaste Province).

##### Etymology.

This species is named after Josephine J. Rodriguez, in recognition of her contribution to the understanding of ACG Hymenoptera taxonomy.

#### Euplectrus johnheratyi

Taxon classificationAnimaliaHymenopteraEulophidae

Hansson sp. n.

http://zoobank.org/515DFD26-3FF8-48C9-85F6-1A433CEA6263

Figures 359–365 , 370–372 , 765

##### Material.

Holotype a female labeled “COSTA RICA: Alajuela, ACG, Sector Pitilla, Pasmompa, 10.vi.2005, C. Moraga, ex *Cropia rivulosa* eating *Cordia bicolor*, sibling of wasp DHJPAR0028817, 05-SRNP-32014” (BMNH). PARATYPES: 5♀ 3♂ with same label data as holotype (BMNH, CNC, INBio, USNM).

##### Diagnosis.

**Figures 359–369.**
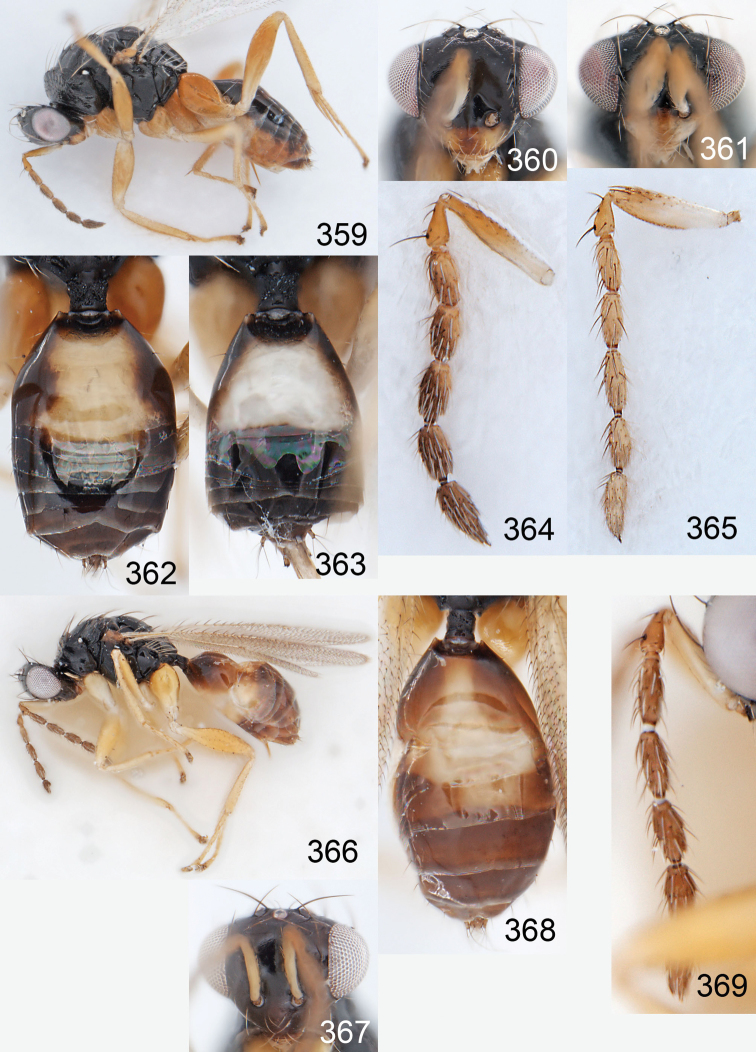
*Euplectrus* spp. **359–365** *Euplectrus johnheratyi* (DHJPAR0028817): **359** habitus in lateral view, female **360** head in frontal view, female **361** head in frontal view, male **362** gaster in dorsal view, female **363** gaster in dorsal view, male **354** antenna in lateral view, female **365** antenna in lateral view, male; **366–369** *Euplectrus carlowae* (paratype), female: **366** habitus in lateral view **367** head in frontal view **368** gaster in dorsal view **369** antenna in lateral view.

Lower face medially yellowish-brown (female, Fig. 360) or white (male, Fig. 361) with median part pale brown, pale area reaching slightly outside level of lateral margins of toruli; scutellum with distinctly elongate meshes (Fig. 372); dorsellum along anterior margin with a very wide groove, 0.5× as long as length of dorsellum, surface behind groove with a median carina (Fig. 765); legs yellowish-brown in female (Fig. 359), paler in male; propodeum anteromedially with a triangular cup that has posterior part strongly raised and distinctly higher than anterior part (Fig. 765); petiole 0.7× as long as wide; gaster in anterior ½ yellowish-brown (female, Fig. 362) or white (male, Fig. 363) with dark brown lateral margins, posterior ½ dark brown; male antenna with scape slightly expanded and widest in the middle, 3.5× as long as wide, sensory area yellowish-brown and slightly darker than scape (Fig. 365).

##### Description.

**Figures 370–375.**
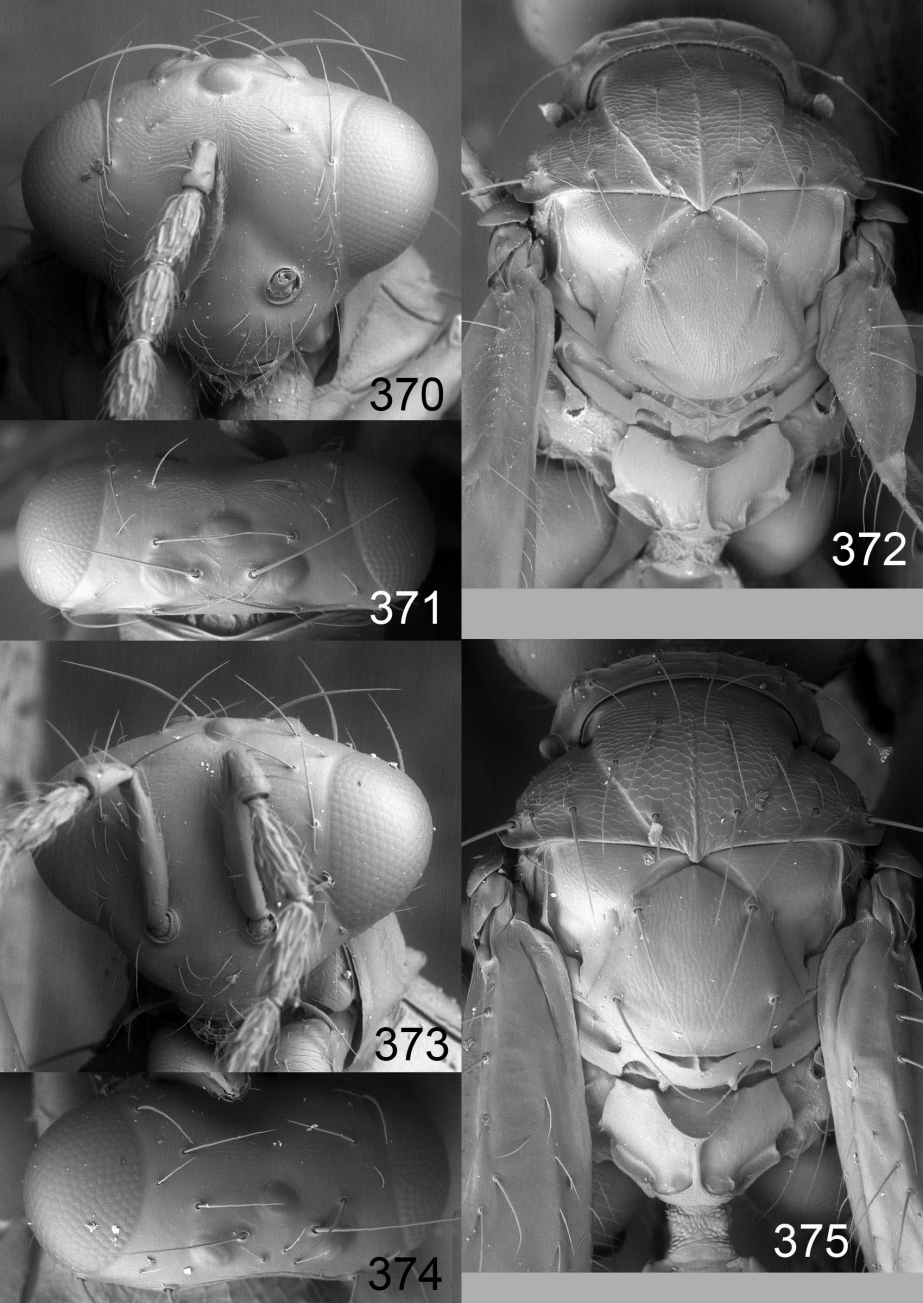
*Euplectrus* spp., females. **370–372** *Euplectrus johnheratyi* (DHJPAR0028817): **370** head in frontal view **371** vertex **372** mesosoma in dorsal view; **373–375** *Euplectrus carlowae* (paratype): **373** head in frontal view **374** vertex **375** mesosoma in dorsal view.

*Female*. Length of body 2.8 mm. Antenna with scape yellowish-white in basal ½ and yellowish-brown in apical ½, pedicel yellowish-brown, flagellomeres 1–3 yellowish-brown ventrally and dark brown dorsally, 4–6 dark brown (Fig. 364). Mandibles and palpi yellowish-white. Head black and shiny, lower face medially yellowish-brown with median part pale brown, reaching slightly outside level of lateral margin of toruli, parts between pale area and eyes black (Fig. 360). Frons close to eyes with two rows of setae (Fig. 370). Vertex smooth (Fig. 371). Occipital margin with a weak carina (Fig. 371).

Mesosoma black and shiny (Fig. 359). Each sidelobe of mesoscutum with 12 setae. Scutellum 1.0× as long as wide; with weak engraved reticulation and with distinctly elongate meshes (Fig. 372). Dorsellum along anterior margin with a very wide groove that is divided by longitudinal carinae, groove 0.5× as long as length of dorsellum, surface behind groove with a median carina (Fig. 765). Propodeum with very weak reticulation (Fig. 765); anteromedially with a transverse circular cup that has posterior part strongly raised and is distinctly higher than anterior part; propodeal callus with ten setae. Legs yellowish-brown (Fig. 359). Fore wing: costal cell on ventral surface with two rows of setae in basal ⅔ and one row in apical ⅓, and margin with two setae close to marginal vein; with 15 admarginal setae, in one row.

Gaster in anterior ½ yellowish-brown with dark brown lateral margins, posterior ½ dark brown (Fig. 362).

Ratios. HE/MS/WM = 2.0/1.0/1.2; POL/OOL/POO = 7.2/4.0/1.0; OOL/DO = 1.2; WE/WF/WH/HH = 1.0/2.6/4.6/3.4; WH/WT = 1.0; PM/ST = 1.5; TS1/TS2/LT/LT1/LT2/LT3/LT4 = 4.2/2.5/6.7/2.7/1.5/1.0/1.5; LP/WP = 0.7; MM/LG = 1.1.

*Male*. Length of body 1.9 mm. Scape slightly expanded and widest in the middle (Fig. 365), sensory pores confined to apicoventral ¾, sensory area yellowish-brown and slightly darker than scape. Otherwise similar to female except lower face with pale part white (Fig. 361), legs paler, petiole longer, gaster shorter and with lateral parts of pale area white (Fig. 363).

Ratios. LC/WS = 3.5; LP/WP = 1.0; MM/LG = 1.5.

##### Hosts and biology.

Feeding on last instar larva of *Cropia rivulosa* (Noctuidae) feeding on *Cordia bicolor* (Boraginaceae), parasitoid cocoons stuck to dead larva and substrate.

##### Distribution.

Costa Rica (Alajuela Province).

##### Etymology.

This species is named after John M. Heraty, in recognition of his contribution to the understanding of ACG Hymenoptera taxonomy.

#### Euplectrus johnlasallei

Taxon classificationAnimaliaHymenopteraEulophidae

Hansson sp. n.

http://zoobank.org/F2311B5C-3C46-4941-A249-0BCE2E909538

Figures 31 , 376–382 , 387–389 , 766

##### Material.

Holotype a female labeled “COSTA RICA: Guanacaste, ACG, Sector Pitilla, Colocho, 21.ii.2007, C. Moraga, ex *Sericochroa* Janzen01 eating *Vochysia guatemalensis*, sibling of wasp DHJPAR0028927, 07-SRNP-31411” (BMNH). PARATYPES: 15♀ 2♂ with same label data as holotype (BMNH, CNC, INBio, MZLU, USNM).

##### Diagnosis.

**Figures 376–386.**
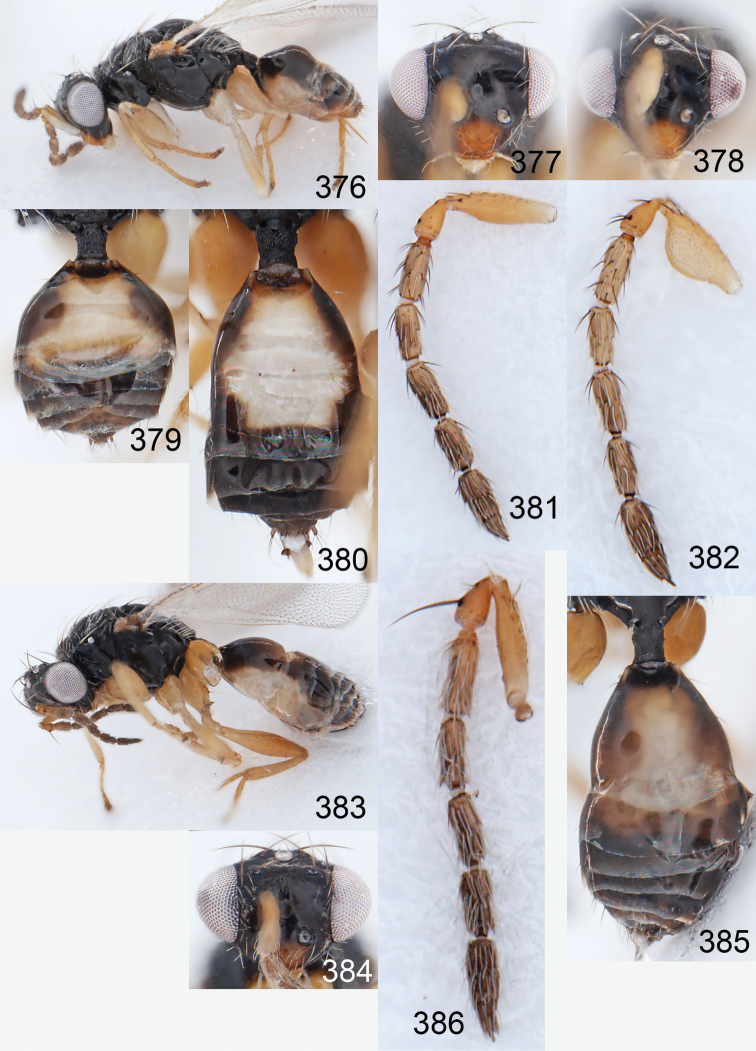
*Euplectrus* spp. **376–382** *Euplectrus johnlasallei* (DHJPAR0028927): **376** habitus in lateral view, female **377** head in frontal view, female **378** head in frontal view, male **379** gaster in dorsal view, female **380** gaster in dorsal view, male **381** antenna in lateral view, female **382** antenna in lateral view, male; **383–386** *Euplectrus hansoni* (non-type), female: **383** habitus in lateral view **384** head in frontal view **385** gaster in dorsal view **386** antenna in lateral view.

Lower face medially yellowish-brown, pale area reaching to level of middle of toruli (Figs 377, 378); legs yellowish-brown (Fig. 376); propodeum with a wide median carina (Fig. 766); petiole 1.0× as long as wide; gaster dark brown, anterior ½ with a large yellowish-brown (female, Fig. 379) or white (male, Fig. 380) spot; male antenna with scape expanded and widest slightly above the middle, 2.2× as long as wide, narrow at base and apex (Fig. 382), sensory pores scattered all over outer lateral surface.

##### Description.

**Figures 387–392.**
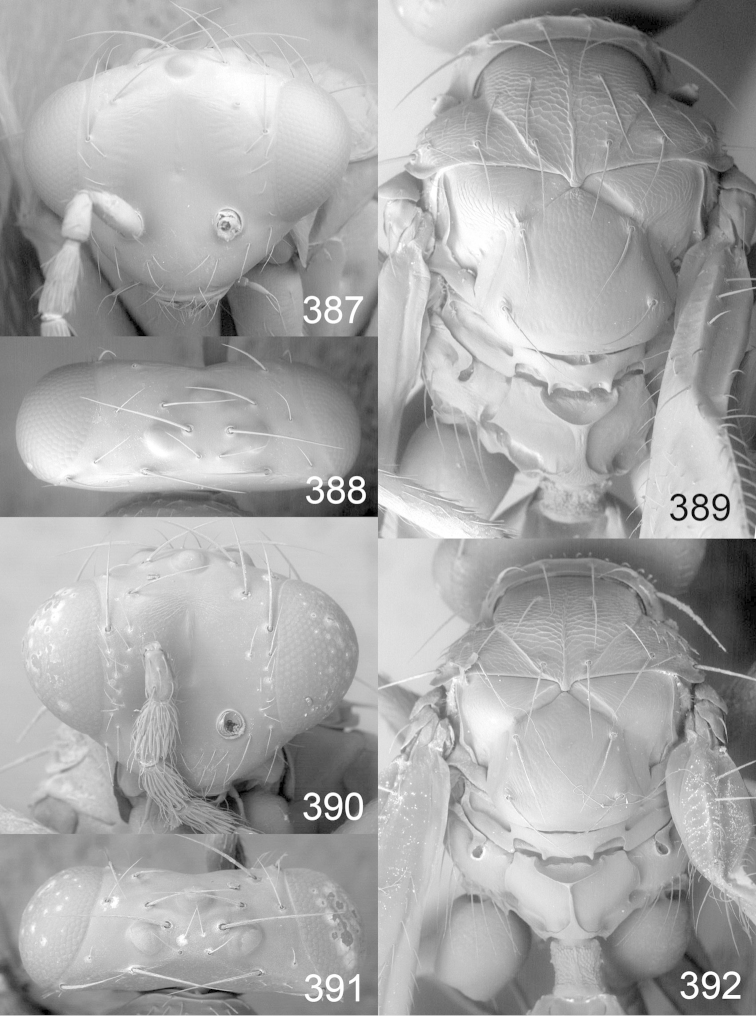
*Euplectrus* spp., females. **387–389** *Euplectrus johnlasallei* (DHJPAR0028927): **387** head in frontal view **388** vertex **389** mesosoma in dorsal view; **390–392** *Euplectrus hansoni* (non-type, no codes): **390** head in frontal view **391** vertex **392** mesosoma in dorsal view.

*Female*. Length of body 2.2 mm. Antenna with scape and pedicel yellowish-brown, flagellomere 1 yellowish-brown, 2 dark brown dorsally and yellowish-brown ventrally, 3–6 dark brown (Fig. 381). Mandibles and palpi yellowish-white. Head black and shiny, lower face medially yellowish-brown, pale area reaching to level of middle of toruli, parts outside level of lateral margin of toruli and eyes black (Fig. 377). Frons close to eyes with one sparse row of setae (Fig. 387). Vertex smooth (Fig. 388). Occipital margin rounded (Fig. 388).

Mesosoma black and shiny (Fig. 376). Each sidelobe of mesoscutum with eight setae. Scutellum 0.9× as long as wide; with weak engraved reticulation (Fig. 389). Dorsellum along anterior margin with a groove, medially 0.3× as long as length of dorsellum (Fig. 766). Propodeum smooth (Fig. 766); anteromedially with a semicircular cup; propodeal callus with seven setae. Legs yellowish-brown (Fig. 376). Fore wing: costal cell with two irregular rows of setae on ventral surface, and margin with three setae close to marginal vein; with 17 admarginal setae, in one row.

Gaster in anterior ½ with a yellowish-brown spot and with lateral margins dark brown, posterior ½ dark brown (Fig. 379).

Ratios. HE/MS/WM = 1.9/1.0/1.2; POL/OOL/POO = 4.9/3.6/1.0; OOL/DO = 1.7; WE/WF/WH/HH = 1.0/3.0/5.2/4.0; WH/WT = 1.1; PM/ST = 1.5; TS1/TS2/LT/LT1/LT2/LT3/LT4 = 4.9/3.1/6.9/2.6/1.6/1.0/1.4; LP/WP = 1.0; MM/LG = 1.1.

*Male*. Length of body 2.0 mm. Scape white, expanded and widest slightly above the middle (Figs 382), narrow at base and apex, sensory pores scattered all over outer lateral surface. Otherwise similar to female.

Ratio. LC/WS = 2.2.

##### Hosts and biology.

Feeding on third instar larva of *Sericochroa* Janzen01 (Notodontidae) feeding on *Vochysia guatemalensis* (Vochysiaceae), parasitoid cocoons stuck to dead larva and substrate.

##### Distribution.

Costa Rica (Guanacaste Province).

##### Etymology.

This species is named after John La Salle, in recognition of his contribution to the understanding of ACG Hymenoptera taxonomy.

#### Euplectrus johnnoyesi

Taxon classificationAnimaliaHymenopteraEulophidae

Hansson sp. n.

http://zoobank.org/F394049C-BBBE-48BD-8CF9-E94174A5FE4C

Figures 17 , 393–399 , 404–406 , 581 , 767

##### Material.

Holotype a female labeled “COSTA RICA: Alajuela, ACG, Sector Pitilla, Pasmompa, 1.xi.2007, P. Rios, ex *Gonodonta sinaldus* eating *Cissampelos pareira*, sibling of wasp DHJPAR0028699, 07-SRNP-33766” (BMNH). PARATYPES: 16♀ 10♂: COSTA RICA (ACG): **Alajuela:** Sector Pitilla, Pasmompa: 3♀ 1♂ with same label data as holotype (BMNH, INBio); 17.x.2006, P. Rios, ex *Oraesia serpens* eating *Cissampelos pareira*, sibling of wasp DHJPAR0028863, 06-SRNP-34907 (2♀ 1♂, in INBio); 18.x.2007, M. Rios, ex *Gonodonta sinaldus* eating *Cissampelos pareira*, sibling of wasp DHJPAR0028952, 07-SRNP-33589 (1♀ 1♂, in CNC); 11.vii.2008, M. Rios, ex *Gonodonta holosericea* eating *Cissampelos pareira*, sibling of wasp DHJPAR0031154, 08-SRNP-31542 (1♀, in INBio); Sector Pitilla, Coneja: 17.vii.2005, C. Moraga, ex *Gonodonta sicheas* eating *Cissampelos pareira*, sibling of wasp DHJPAR0028807, 05-SRNP-32687 (3♀ 1♂, in BMNH); 6.xi.2005, P. Rios, ex *Gonodonta holosericea* eating *Cissampelos pareira*, sibling of wasp DHJPAR0028834, 05-SRNP-34610 (1♀ 1♂, in MIUCR); 6.xi.2005, P. Rios, ex *Gonodonta holosericea* on *Cissampelos pareira*, sibling of wasp DHJPAR0028823, 05-SRNP-34616 (3♂, in BMNH, INBio); 11.x.2007, M. Rios, ex *Gonodonta sinaldus* eating *Cissampelos pareira*, sibling of wasp DHJPAR0028954, 07-SRNP-33516 (1♀ 1♂, in USNM); 1.xi.2006, M. Rios, ex *Oraesia serpens* on *Cissampelos tropaeolifolia*, sibling of wasp DHJPAR0028858, 06-SRNP-65085 (4♀ 1♂, in BMNH, INBio).

##### Diagnosis.

**Figures 393–403.**
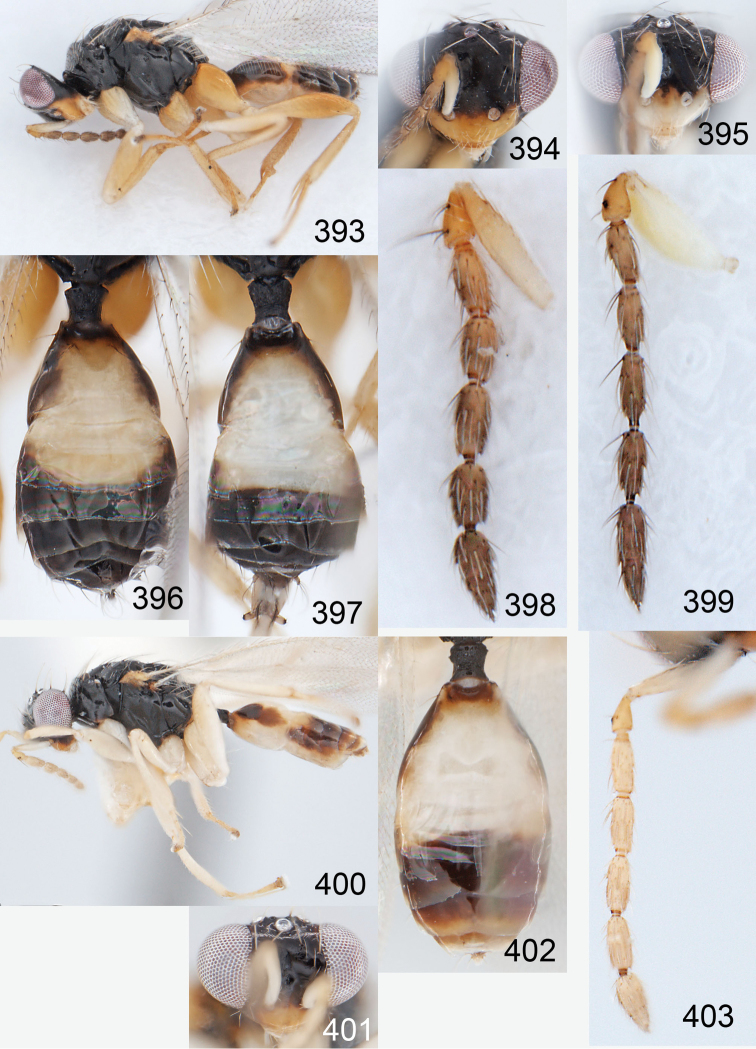
*Euplectrus* spp. **393–399** *Euplectrus johnnoyesi* (DHJPAR0028699): **393** habitus in lateral view, female **394** head in frontal view, female **395** head in frontal view, male **396** gaster in dorsal view, female **397** gaster in dorsal view, male **398** antenna in lateral view, female **399** antenna in lateral view, male; **400–403** *Euplectrus ireneae* (paratype), female: **400** habitus in lateral view **401** head in frontal view **402** gaster in dorsal view **403** antenna in lateral view.

Lower face entirely yellowish-brown (female, Fig. 394) or white (male, Fig. 395), pale part not reaching hypostomal carina and with area close to mouth cavity dark (Fig. 581); fore coxa yellowish-white, remaining fore leg and mid and hind legs yellowish-brown (Fig. 393); petiole 1.0× as long as wide; gaster dark brown, anterior ½ yellowish-white (female, Fig. 396) or white (male, Fig. 397) with dark brown lateral margins; male antenna with scape slightly expanded and widest in the middle, 2.6× as long as wide (Fig. 399), flagellomeres 2–5 with a basal whorl of erect setae.

##### Description.

**Figures 404–409.**
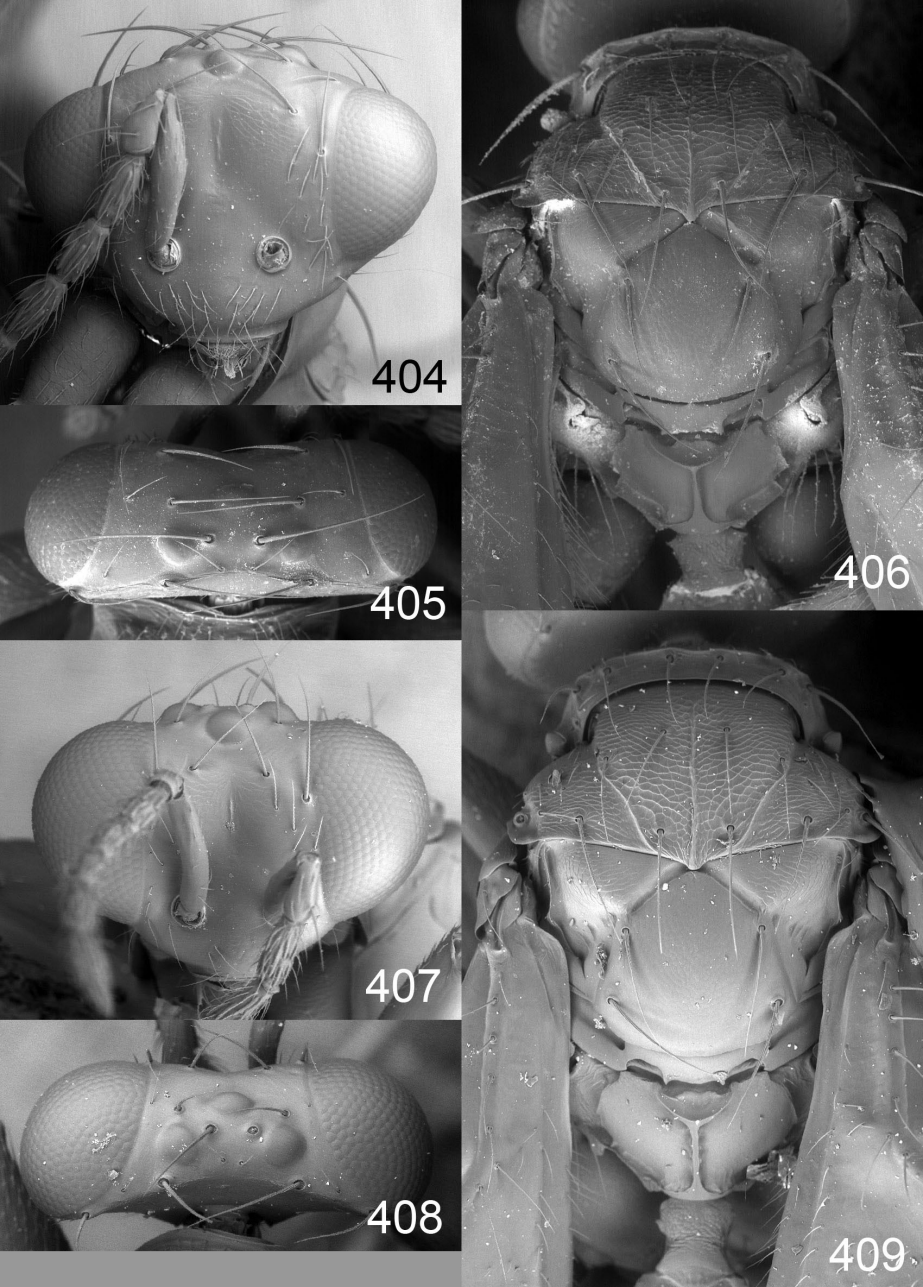
*Euplectrus* spp., females. **404–406** *Euplectrus johnnoyesi* (DHJPAR0028699): **404** head in frontal view **405** vertex **406** mesosoma in dorsal view; **407–409** *Euplectrus ireneae* (95-SRNP-6049, no barcode): **407** head in frontal view **408** vertex **409** mesosoma in dorsal view.

*Female*. Length of body 2.3 mm. Antenna with scape yellowish-white, pedicel yellowish-brown, flagellomere 1 yellowish-brown, 2 pale brown, 3–6 dark brown (Fig. 398). Mandibles and palpi yellowish-white. Head black and shiny, entire lower face yellowish-brown with median part darker (Fig. 394). Frons close to eyes with two rows of setae (Fig. 404). Vertex smooth (Fig. 405). Occipital margin rounded (Fig. 405).

Mesosoma black and shiny (Fig. 393). Each sidelobe of mesoscutum with 20 setae. Scutellum 0.9× as long as wide; with very weak engraved reticulation (Fig. 406). Dorsellum along anterior margin with a very narrow groove (Fig. 767), medially 0.1× as long as length of dorsellum. Propodeum with very weak reticulation (Fig. 767); anteromedially with a semicircular cup that has posterior part strongly raised and is distinctly higher than anterior part; propodeal callus with eight setae. Legs (Fig. 393): fore coxa yellowish-white, remaining fore leg and mid and hind legs yellowish-brown. Fore wing: costal cell on ventral surface with two rows of setae in basal ⅔ and one row in apical ⅓, and margin with six setae close to marginal vein; with 16 admarginal setae, in one row.

Gaster dark brown, anterior ½ yellowish-white with dark brown lateral margins (Fig. 396).

Ratios. HE/MS/WM = 2.1/1.0/1.4; POL/OOL/POO = 4.9/2.9/1.0; OOL/DO = 1.4; WE/WF/WH/HH = 1.0/2.5/4.5/3.2; WH/WT = 1.1; PM/ST = 1.6; TS1/TS2/LT/LT1/LT2/LT3/LT4 = 4.0/2.4/6.4/2.0/1.4/1.0/1.8; LP/WP = 1.0; MM/LG = 0.9.

*Male*. Length of body 1.8 mm. Scape slightly expanded and widest in the middle (Fig. 399), sensory pores confined to apicoventral ¾, sensory area pale as scape. Otherwise similar to female except flagellomeres 2–5 with a basal whorl of erect setae (Fig. 399); entire lower face white (Fig. 395); scutellum with posterior ¼ smooth; gaster shorter and with pale part white.

Ratios. LC/WS = 2.6; MM/LG = 1.2.

##### Hosts and biology.

Feeding on various instars of *Gonodonta holosericea*, *Gonodonta sicheas*, *Gonodonta sinaldus*, *Oraesia serpens* (all are Erebidae), feeding on *Cissampelos pareira* and *Cissampelos tropaeolifolia* (Menispermaceae), parasitoid cocoons stuck to dead larva and substrate.

##### Distribution.

Costa Rica (Alajuela Province).

##### Etymology.

This species is named after John S. Noyes, in recognition of his contribution to the understanding of ACG Hymenoptera taxonomy.

##### Remarks.

*Euplectrus johnnoyesi* is morphologically identical to *Euplectrus sydneycameronae*, but differs in the barcode and biology. See remarks under *Euplectrus sydneycameronae*.

#### Euplectrus josefernandezi

Taxon classificationAnimaliaHymenopteraEulophidae

Hansson sp. n.

http://zoobank.org/6FC88B1B-97C2-4179-968A-AB9F66B2CAC0

Figures 32–33 , 410–416 , 421–423 , 768

##### Material.

Holotype a female labeled “COSTA RICA: Guanacaste, ACG, Sector San Cristobal, Quebrada Cementerio, 18.vi.2007, G. Sihezar, ex *Euglyphis jessiehillae* eating *Nectandra hihua*, sibling of wasp DHJPAR0028908, 07-SRNP-2713” (BMNH). PARATYPES: 83♀ 7♂: COSTA RICA (ACG): **Guanacaste:** Sector San Cristobal: 42♀ 5♂ with same label data as holotype (BMNH, CNC, INBio, MZLU, MIUCR, USNM); Sendero Pinyal, 17.vi.2006, O. Espinoza, ex *Euglyphis jessiehillae* eating *Nectandra hihua*, sibling of wasp DHJPAR0028874, 06-SRNP-4798 (41♀ 2♂, in BMNH, CNC, INBio, MIUCR, USNM).

##### Diagnosis.

**Figures 410–420.**
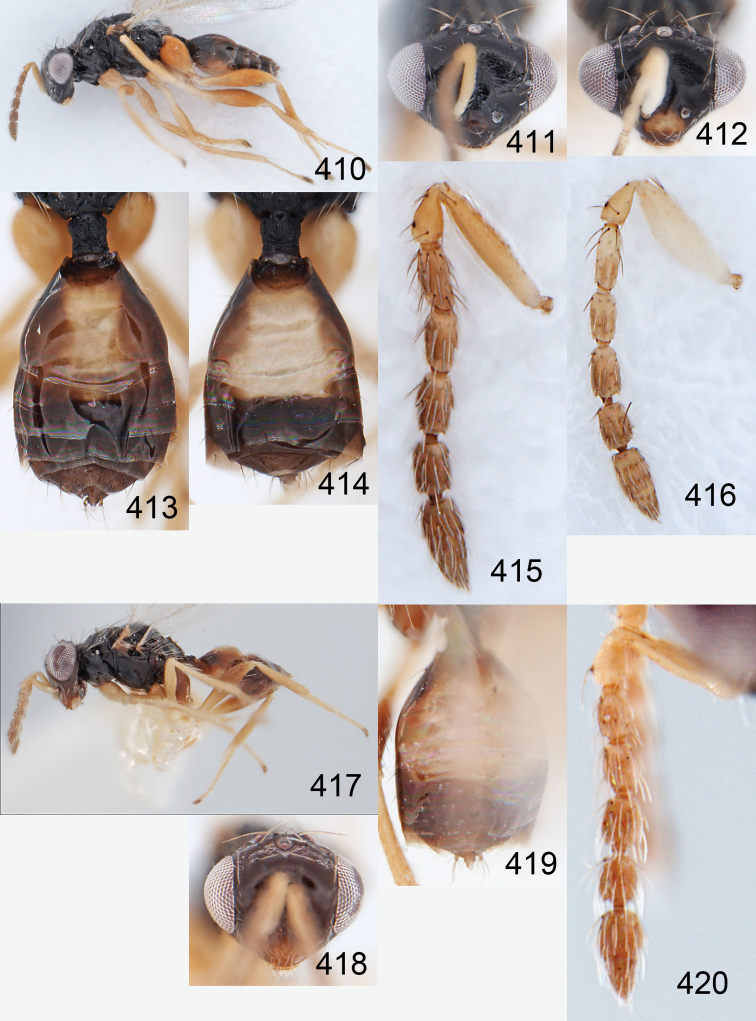
*Euplectrus* spp. **410–416** *Euplectrus josefernandezi* (DHJPAR0028908): **410** habitus in lateral view, female **411** head in frontal view, female **412** head in frontal view, male **413** gaster in dorsal view, female **414** gaster in dorsal view, male **415** antenna in lateral view, female **416** antenna in lateral view, male **417–420** *Euplectrus orias* (paratype), female: **417** habitus in lateral view **418** head in frontal view **419** gaster in dorsal view **420** antenna in lateral view.

Lower face medially very dark reddish-brown - almost black (female, Fig. 411), or yellowish-brown (male, Fig. 412), pale area reaching to level of middle of toruli; legs yellowish-brown to yellowish-white (Fig. 410); petiole 1.0× as long as wide; female gaster dark brown, anterior ½ with a narrow pale brown spot (Fig. 413), in male with pale spot wider, with dark brown lateral margins (Fig. 414); male antenna with scape slightly expanded and widest in the middle, 3.5× as long as wide (Fig. 416).

##### Description.

**Figures 421–427.**
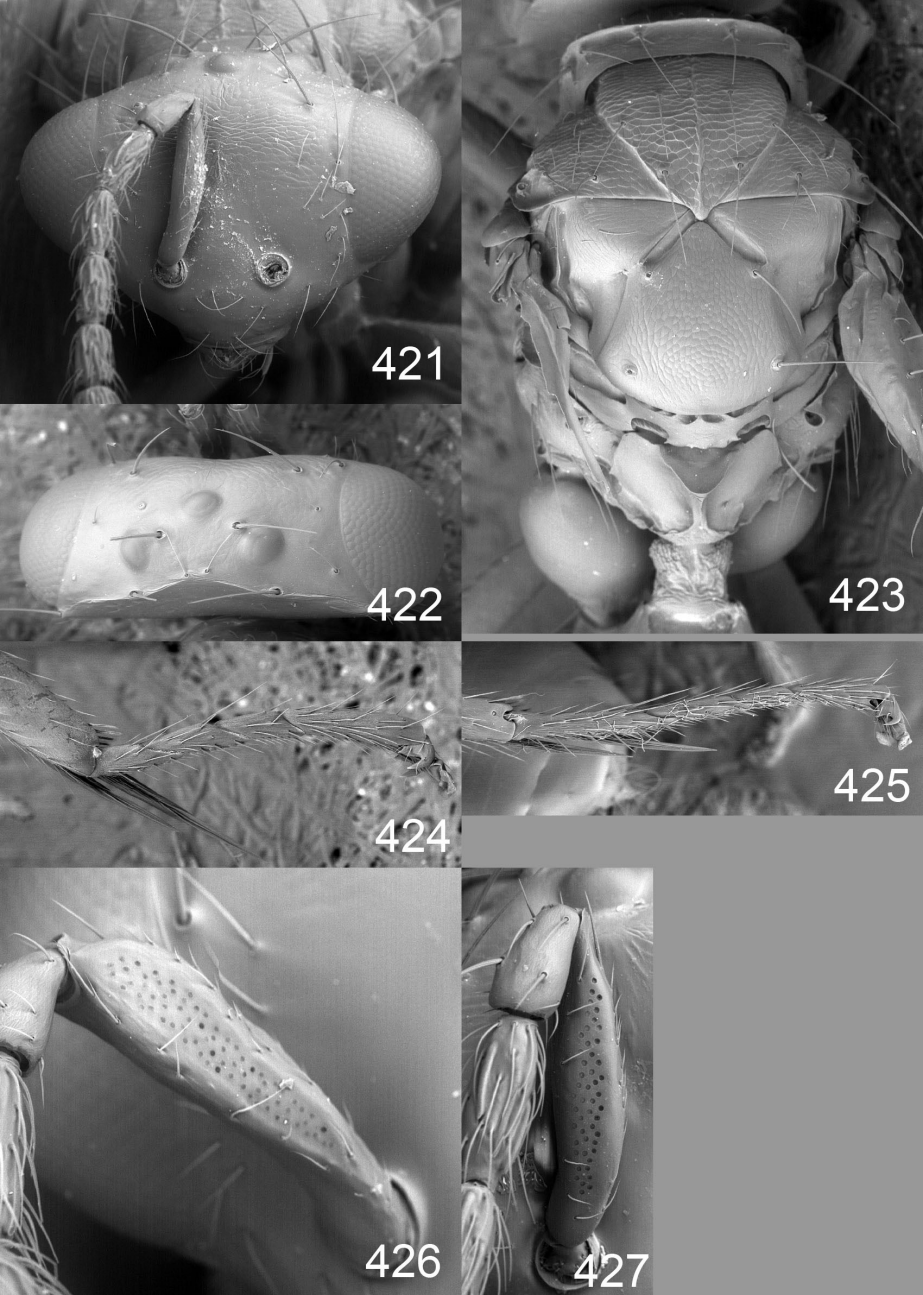
*Euplectrus* spp. **421–423** *Euplectrus josefernandezi* (DHJPAR0028908): **421** head in frontal view **422** vertex **423** mesosoma in dorsal view; **424–425** hind tarsus in lateral view, females **424** *Euplectrus garygibsoni* (DHJPAR0042124) **425** *Euplectrus eowilsoni* (DHJPAR0028932) **426-427** scape in ventral view, males **426** *Euplectrus ivonae* **427** *Euplectrus andybennetti*.

*Female*. Length of body 2.1 mm. Antenna with scape and pedicel yellowish-brown, flagellomere 1 pale brown, 2–6 dark brown (Fig. 415). Mandibles and palpi yellowish-white. Head black and shiny, lower face medially very dark reddish-brown - almost black, parts between pale area and eyes black (Fig. 411). Frons close to eyes with one row of setae (Fig. 421). Vertex smooth (Fig. 422). Occipital margin with a carina (Fig. 422).

Mesosoma black and shiny (Fig. 410). Each sidelobe of mesoscutum with eight setae. Scutellum 1.0× as long as wide; with weak engraved reticulation (Fig. 423). Dorsellum along anterior margin with a narrow groove that is divided by longitudinal carinae (Fig. 768), groove medially 0.3× as long as length of dorsellum. Propodeum with very weak reticulation (Fig. 768); anteromedially with a triangular cup that has posterior part strongly raised and distinctly higher than anterior part; propodeal callus with nine setae. Legs yellowish-brown (Fig. 410). Fore wing: costal cell with one complete row of setae on ventral surface, and margin with three setae close to marginal vein; with 15 admarginal setae, in one row.

Gaster dark brown, anterior ½ with a narrow yellowish-brown spot (Fig. 413).

Ratios. HE/MS/WM = 1.8/1.0/1.2; POL/OOL/POO = 3.6/2.4/1.0; OOL/DO = 1.3; WE/WF/WH/HH = 1.0/3.0/5.1/3.6; WH/WT = 1.2; PM/ST = 1.4; TS1/TS2/LT/LT1/LT2/LT3/LT4 = 4.5/2.8/6.8/2.8/1.5/1.0/1.5; LP/WP = 1.0; MM/LG = 1.1.

*Male*. Length of body 1.8 mm. Scape slightly expanded and widest in the middle (Fig. 416), sensory pores confined to apicoventral ¾, sensory area pale as scape. Otherwise similar to female except lower face with median part yellowish-brown (Fig. 412); fore and mid legs yellowish-white; gaster with pale spot wider, reaching to dark brown lateral margins (Fig. 414), and shorter.

Ratios. LC/WS = 3.5; MM/LG = 1.2.

##### Hosts and biology.

Feeding on penultimate instar larva of *Euglyphis jessiehillae* (Lasiocampidae) feeding on *Nectandra hihua* (Lauraceae), parasitoid cocoons stuck to leaf substrate.

##### Distribution.

Costa Rica (Guanacaste Province).

##### Etymology.

This species is named after Jose Fernandez-Triana, in recognition of his contribution to the understanding of ACG Hymenoptera taxonomy.

#### Euplectrus lubomirmasneri

Taxon classificationAnimaliaHymenopteraEulophidae

Hansson sp. n.

http://zoobank.org/E271D0F5-AFF8-4E01-ADDC-1EA00F1B0A85

Figures 29–30 , 428–434 , 435–437 , 769

##### Material.

Holotype a female labeled “COSTA RICA: Guanacaste, ACG, Sector Pitilla, Pasmompa, 3.xi.2004, M. Rios, ex *Pseudoplusia includens* eating *Acalypha macrostachya*, sibling of wasp DHJPAR0028760, 04-SRNP-56044” (BMNH). PARATYPES: 36♀ 7♂: COSTA RICA (ACG): **Guanacaste:** Sector Pitilla, Pasmompa: 19♀ 4♂ with same label data as holotype (BMNH, CNC, INBio, MZLU, MIUCR, USNM); 29.vii.2005, P. Rios, ex *Pseudoplusia includens* eating *Acalypha macrostachya*, sibling of wasp DHJPAR0028822, 05-SRNP-32941 (17♀ 2♂, in BMNH, CNC, INBio, MIUCR, USNM); 1.xii.2008, P. Rios, ex *Cecharismena zoum* eating *Acalypha macrostachya*, sibling of wasp DHJPAR0030530, 08-SRNP-32998 (1♂, in INBio).

##### Diagnosis.

**Figures 428–434.**
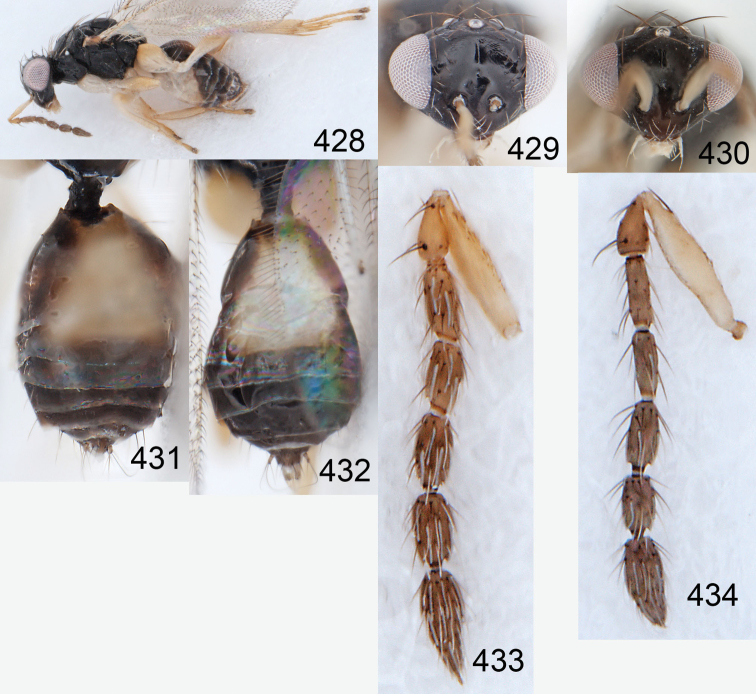
*Euplectrus lubomirmasneri* (DHJPAR0028760): **428** habitus in lateral view, female **429** head in frontal view, female **430** head in frontal view, male **431** gaster in dorsal view, female **432** gaster in dorsal view, male **433** antenna in lateral view, female **434** antenna in lateral view, male.

Lower face black, median part very dark reddish-brown, almost black (Fig. 429); mandibles dark brown; fore and mid coxae white, remaining parts of fore and mid legs and entire hind leg yellowish-brown (Fig. 428); petiole 1.0× as long as wide; gaster in anterior ½ with a yellowish-brown (female, Fig. 431) or white (male, Fig. 432) spot and with lateral margins dark brown, posterior ½ dark brown; male antenna with scape narrow and widest above the middle, 3.6× as long as wide (Fig. 434).

##### Description.

**Figures 435–440.**
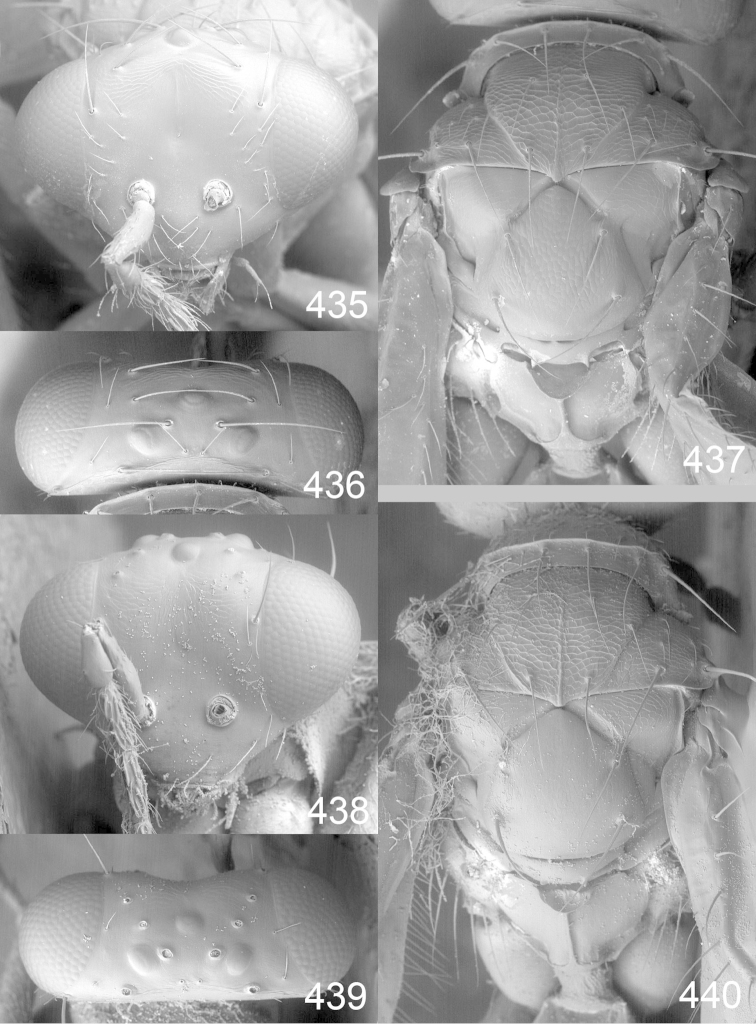
*Euplectrus* spp., females **435–437.** *Euplectrus lubomirmasneri* (DHJPAR0028760): **435** head in frontal view **436** vertex **437** mesosoma in dorsal view **438–440** *Euplectrus mikegatesi* (DHJPAR0031184): **438** head in frontal view **439** vertex **440** mesosoma in dorsal view.

*Female*. Length of body 1.9 mm. Antenna with scape yellowish-white and pedicel yellowish-brown, flagellomere 1 yellowish-brown, 2–6 dark brown (Fig. 433). Mandibles dark brown and palpi yellowish-white. Head black and shiny, lower face black with median part very dark reddish-brown, almost black, not delimited from surrounding parts of frons (Fig. 429). Frons close to eyes with one sparse row of setae, row moving away from eye in upper part (Fig. 435). Vertex smooth (Fig. 436). Occipital margin with a weak carina behind ocellar triangle (Fig. 436).

Mesosoma black and shiny (Fig. 428). Each sidelobe of mesoscutum with eight setae. Scutellum 0.9× as long as wide; with weak engraved reticulation (Fig. 437). Dorsellum along anterior margin with a very narrow groove (Fig. 769), medially 0.1× as long as length of dorsellum. Propodeum with very weak reticulation (Fig. 769); anteromedially with a triangular cup with posterior part strongly raised and distinctly higher than anterior part; propodeal callus with seven setae. Legs (Fig. 428): fore and mid coxae white, remaining parts of fore and mid legs and entire hind leg yellowish-brown. Fore wing: costal cell with one complete row of setae on ventral surface, and margin with seven setae close to marginal vein; with 18 admarginal setae, in one row in the middle, in two rows at base and apically.

Gaster in anterior ½ yellowish-brown with wide dark brown lateral margins, posterior ½ dark brown (Fig. 431).

Ratios. HE/MS/WM = 2.1/1.0/1.3; POL/OOL/POO = 7.0/3.7/1.0; OOL/DO = 1.4; WE/WF/WH/HH = 1.0/2.5/4.6/3.2; WH/WT = 1.2; PM/ST = 2.0; TS1/TS2/LT/LT1/LT2/LT3/LT4 = 4.0/2.4/6.0/1.9/1.3/1.0/1.6; LP/WP = 1.0; MM/LG = 1.1.

*Male*. Length of body 1.8 mm. Scape narrow and widest above the middle (Figs 434), sensory pores confined to apicoventral ⅔, sensory area pale as scape. Similar to female except pale spot on gaster is white (Fig. 432).

Ratios. LC/WS = 3.6.

##### Hosts and biology.

Feeding on last instar larvae of *Pseudoplusia includens* feeding on *Acalypha macrostachya*, *Cecharismena zoum* feeding on *Acalypha macrostachya* (Euphorbiaceae), (both hosts are Noctuidae). Parasitoid cocoons stuck to dead larva and substrate.

##### Distribution.

Costa Rica (Guanacaste Province).

##### Etymology.

This species is named after Lubo Masner, in recognition of his contribution to the understanding of ACG Hymenoptera taxonomy.

#### Euplectrus markshawi

Taxon classificationAnimaliaHymenopteraEulophidae

Hansson sp. n.

http://zoobank.org/B3589668-0992-436C-BA87-81D71E0C9D01

Figures 264–267 , 271–273 , 770

##### Material.

Holotype a female labeled “COSTA RICA: Guanacaste, ACG, Sector Santa Rosa, Quebrada Costa Rica, 12.vi.2006, D. Rivera, ex *Cargida pyrrha* eating *Colubrina elliptica*, sibling of wasp DHJPAR0028896, 06-SRNP-16286” (BMNH). PARATYPES: 2♀ with same label data as holotype (BMNH, INBio).

##### Diagnosis.

Lower face with median part yellowish-brown, slightly darker medially, pale area reaching outside of outer lateral margins of toruli, with a black area the width of width of scape between pale area and eye margin (Fig. 265); scutellum with a small hump posteromedially and reticulate with elongate meshes, scutellum hence appearing striate (Fig. 273); dorsellum anteriorly with a wide groove, 0.5× as long as length of dorsellum (Fig. 770); legs yellowish-brown with hind coxa pale brown (Fig. 257); petiole 0.6× as long as wide; gaster with anterior ½ yellowish-brown with dark brown lateral margins, posterior ½ difficult to see on specimens because apical segments are retracted (Fig. 266). Very similar to *Euplectrus ivonae*, differs in having first tarsomere longer (LT/LT1 = 2.5; = 3.0 in *Euplectrus ivonae*), petiole shorter (LP/WP = 0.6; = 0.8 in *Euplectrus ivonae*), and with hind coxae darker.

##### Description.

*Female*. Length of body 2.1 mm. Antenna with scape yellowish-white in basal ½, yellowish-brown in apical ½, pedicel yellowish-brown, flagellomeres 1–3 dark brown dorsally and yellowish-brown ventrally, 4–6 dark brown (Fig. 267). Mandibles and palpi yellowish-white. Head black and shiny, lower face with median part yellowish-brown, slightly darker medially, pale area reaching outside of outer lateral margins of toruli, with a black area the width of width of scape between pale area and eye margin (Fig. 265). Frons close to eyes with two rows of setae (Fig. 271). Vertex with very weak reticulation inside ocellar triangle, outside triangle smooth (Fig. 272). Occipital margin with a weak carina behind ocellar triangle (Fig. 272).

Mesosoma black and shiny (Fig. 264). Each sidelobe of mesoscutum with 14 setae. Scutellum 1.0× as long as wide; with rather strong engraved reticulation, meshes elongate and scutellum appearing striate, in posteromedian ½ with a small hump (Fig. 273). Dorsellum anteriorly with a wide groove that is divided by longitudinal carinae (Fig. 770), groove medially 0.5× as long as length of dorsellum. Propodeum smooth (Fig. 770); anteromedially with a transverse semicircular cup; propodeal callus with seven setae. Legs yellowish-brown with hind coxa pale brown (Fig. 264). Fore wing: costal cell with one row of setae on ventral surface, and margin with two setae close to marginal vein; with 16 admarginal setae, in one row.

Gaster with anterior ½ yellowish-brown with dark brown lateral margins, posterior ½ difficult to see on specimens (Fig. 266).

Ratios. HE/MS/WM = 1.8/1.0/1.1; POL/OOL/POO = 4.8/2.8/1.0; OOL/DO = 1.7; WE/WF/WH/HH = 1.0/2.5/4.3/3.5; WH/WT = 1.1; PM/ST = 1.4; TS1/TS2/LT/LT1/LT2/LT3/LT4 = 4.6/2.6/7.4/3.0/1.5/1.0/1.6; LP/WP = 0.6; MM/LG = not measurable, gaster with retracted apical segments.

*Male*. Unknown.

##### Hosts and biology.

Feeding on last instar larva of *Cargida pyrrha* (Notodontidae) feeding on *Colubrina elliptica* (Rhamnaceae), parasitoid cocoons stuck to dead larva and substrate.

##### Distribution.

Costa Rica (Guanacaste Province).

##### Etymology.

This species is named after Mark R. Shaw, in recognition of his contribution to the understanding of ACG Hymenoptera taxonomy.

#### Euplectrus mikegatesi

Taxon classificationAnimaliaHymenopteraEulophidae

Hansson sp. n.

http://zoobank.org/203CA6F9-698E-4AC0-9894-39DB327DD6F6

Figures 438–440 , 441–447 , 771

##### Material.

Holotype a female labeled “COSTA RICA: Alajuela, ACG, Sector Brasilia, Piedrona, 21.vi.2008, D. Briceno, ex *Antiblemma amarga* eating *Vochysia ferruginea*, sibling of wasp DHJPAR0031184, 08-SRNP-65612” (BMNH). PARATYPES: 8♀ 1♂: COSTA RICA (ACG): **Alajuela:** 5♀ 1♂ with same label data as holotype (BMNH, INBio, MZLU); **Guanacaste:** Sector Pitilla, Quebradona, 11.iii.2013, R. Calero, ex *Antiblemma amarga* eating *Vochysia guatemalensis*, sibling wasp of DHJPAR0054879, 13-SRNP-70421 (3♀, in CNC, INBio, USNM).

##### Diagnosis.

**Figures 441–452.**
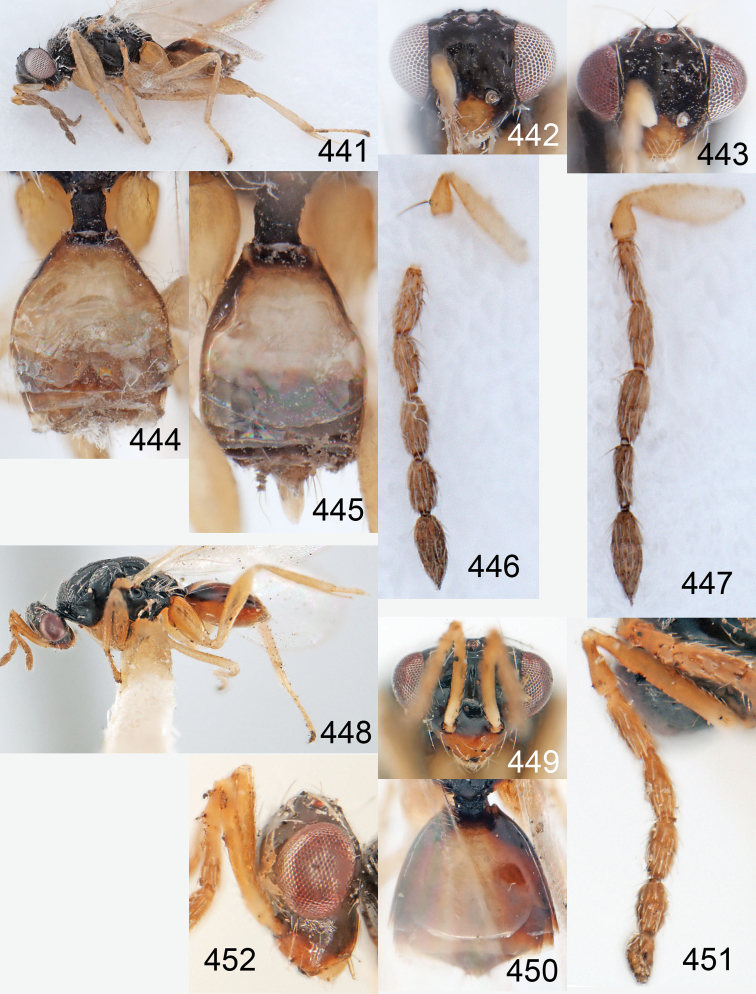
*Euplectrus* spp. **441–447** *Euplectrus mikegatesi* (DHJPAR0031184): **441** habitus in lateral view, female **442** head in frontal view, female **443** head in frontal view, male **444** gaster in dorsal view, female **445** gaster in dorsal view, male **446** antenna in lateral view, female **447** antenna in lateral view, male **448–452** *Euplectrus pachyscaphus* (lectotype), female: **448** habitus in lateral view **449** head in frontal view **450** gaster in dorsal view **451** antenna in lateral view **452** head in lateral view.

Lower face with median part yellowish-brown, pale area reaching to level of middle of toruli (Figs 442, 443); fore wing with four setae on dorsal surface of submarginal vein; dorsellum anteriorly with a very narrow groove (Fig. 771); fore and mid legs yellowish-white, hind leg yellowish-brown (Fig. 441); petiole 1.2× as long as wide; female gaster with anterior ½ yellowish-brown, posterior ½ reddish-brown, entire gaster with dark brown narrow lateral margins (Fig. 444), male gaster with anterior ½ yellowish-white with dark brown lateral margins and posterior ½ dark brown (Fig. 445); male antenna with scape slightly expanded and widest in the middle, 3.1× as long as wide (Fig. 447).

##### Description.

*Female*. Length of body 2.0 mm. Antenna with scape yellowish-white, pedicel yellowish-brown, flagellomeres 1–3 pale brown, 4–6 dark brown (Fig. 446). Mandibles and palpi yellowish-white. Head black and shiny, lower face with median part yellowish-brown, pale area reaching to level of middle of toruli, parts between pale area and eyes black (Fig. 442). Frons close to eyes with one row of setae (Fig. 438). Vertex with very weak reticulation inside ocellar triangle, smooth outside (Fig. 439). Occipital margin rounded (Fig. 439).

Mesosoma black and shiny (Fig. 441). Each sidelobe of mesoscutum with ten setae. Scutellum 0.9× as long as wide; with very weak engraved reticulation, posterior margin smooth (Fig. 440). Dorsellum anteriorly with a very narrow groove (Fig. 771), medially 0.2× as long as length of dorsellum. Propodeum smooth (Fig. 771); anteromedially with a semicircular cup; propodeal callus with seven setae. Legs (Fig. 441): fore and mid legs yellowish-white, hind leg yellowish-brown. Fore wing: submarginal vein with four setae; costal cell with two rows of setae on ventral surface, and margin with four setae close to marginal vein; with 11 admarginal setae, in one row.

Gaster with anterior ½ yellowish-brown, posterior ½ reddish-brown, entire gaster with dark brown narrow lateral margins (Fig. 444).

Ratios. HE/MS/WM = 2.5/1.0/1.4; POL/OOL/POO = 5.3/2.4/1.0; OOL/DO = 0.9; WE/WF/WH/HH = 1.0/2.0/4.0/3.0; WH/WT = 1.1; PM/ST = 1.5; TS1/TS2/LT/LT1/LT2/LT3/LT4 = 3.1/1.8/5.5/1.3/1.3/1.0/1.9; LP/WP = 1.2; MM/LG = 1.4.

*Male*. Length of body 1.6 mm. Scape slightly expanded and widest in the middle (Fig. 447), sensory pores confined to apicoventral ¾, sensory area pale as scape. Otherwise similar to female except gaster with anterior ½ yellowish-white with dark brown lateral margins and posterior ½ dark brown (Fig. 445), and shorter.

Ratios. LC/WS = 3.1; MM/LG = 1.5.

##### Hosts and biology.

Feeding on penultimate instar larva of *Antiblemma amarga* (Erebidae) feeding on *Vochysia ferruginea* and *Vochysia guatemalensis* (Vochysiaceae), parasitoid cocoons stuck to dead larva and substrate.

##### Distribution.

Costa Rica (Alajuela Province).

##### Etymology.

This species is named after Mike W. Gates, in recognition of his contribution to the understanding of ACG Hymenoptera taxonomy.

#### Euplectrus mikeschauffi

Taxon classificationAnimaliaHymenopteraEulophidae

Hansson sp. n.

http://zoobank.org/3FC733AB-C3F4-4272-BA6A-AD088C6D63B2

Figures 26 , 453–459 , 772

##### Material.

Holotype a female labeled “COSTA RICA: Guanacaste, ACG, San Cristobal, Puente Palma, 28.vi.2011, E. Araya, ex *Enyo ocypete* eating *Tetracera hydrophila*, sibling of wasp DHJPAR0045452, 11-SRNP-2500” (BMNH). PARATYPES: 61♀ 6♂: COSTA RICA (ACG): **Guanacaste:** 25♀ 3♂ with same label data as holotype (BMNH, CNC, INBio, MZLU, MIUCR, USNM); Sector Pitilla, Ingas, 20.vi.2011, M. Rios, ex *Enyo ocypete* eating *Davilla nitida*, sibling of wasp DHJPAR0045456, 11-SRNP-31737 (36♀ 3♂, in BMNH, CNC, INBio, MIUCR, USNM).

##### Diagnosis.

**Figures 453–463.**
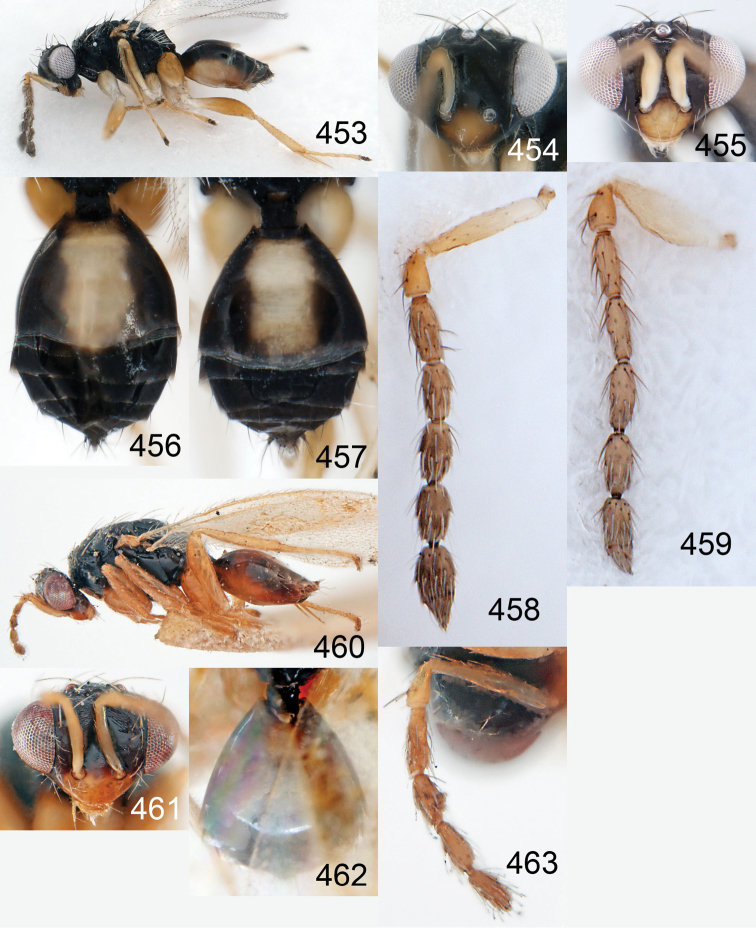
*Euplectrus* spp. **453–459** *Euplectrus mikeschauffi* (DHJPAR0045452): **453** habitus in lateral view, female **454** head in frontal view, female **455** head in frontal view, male **456** gaster in dorsal view, female **457** gaster in dorsal view, male **458** antenna in lateral view, female **459** antenna in lateral view, male; **460–463** *Euplectrus mellipes* (lectotype), female: **460** habitus in lateral view **461** head in frontal view **462** gaster in dorsal view **463** antenna in lateral view.

Lower face medially yellowish-brown, pale area reaching to level of outer lateral margin of toruli (Figs 454, 455); fore and mid coxae yellowish-white, remaining parts of fore and mid legs and entire hind leg yellowish-brown (Fig. 453); dorsellum with a row of foveae along anterior margin (Fig. 772); propodeum with a wide median carina (Fig. 772); petiole 1.0× as long as wide in female, 0.8× in male; gaster in anterior ½ with median part yellowish-white and lateral parts black, posterior ½ black (Figs 456, 457); male antenna with scape enlarged, widest in the middle, and 2.5× as long as wide (Fig. 459).

##### Description.

**Figures 464–469.**
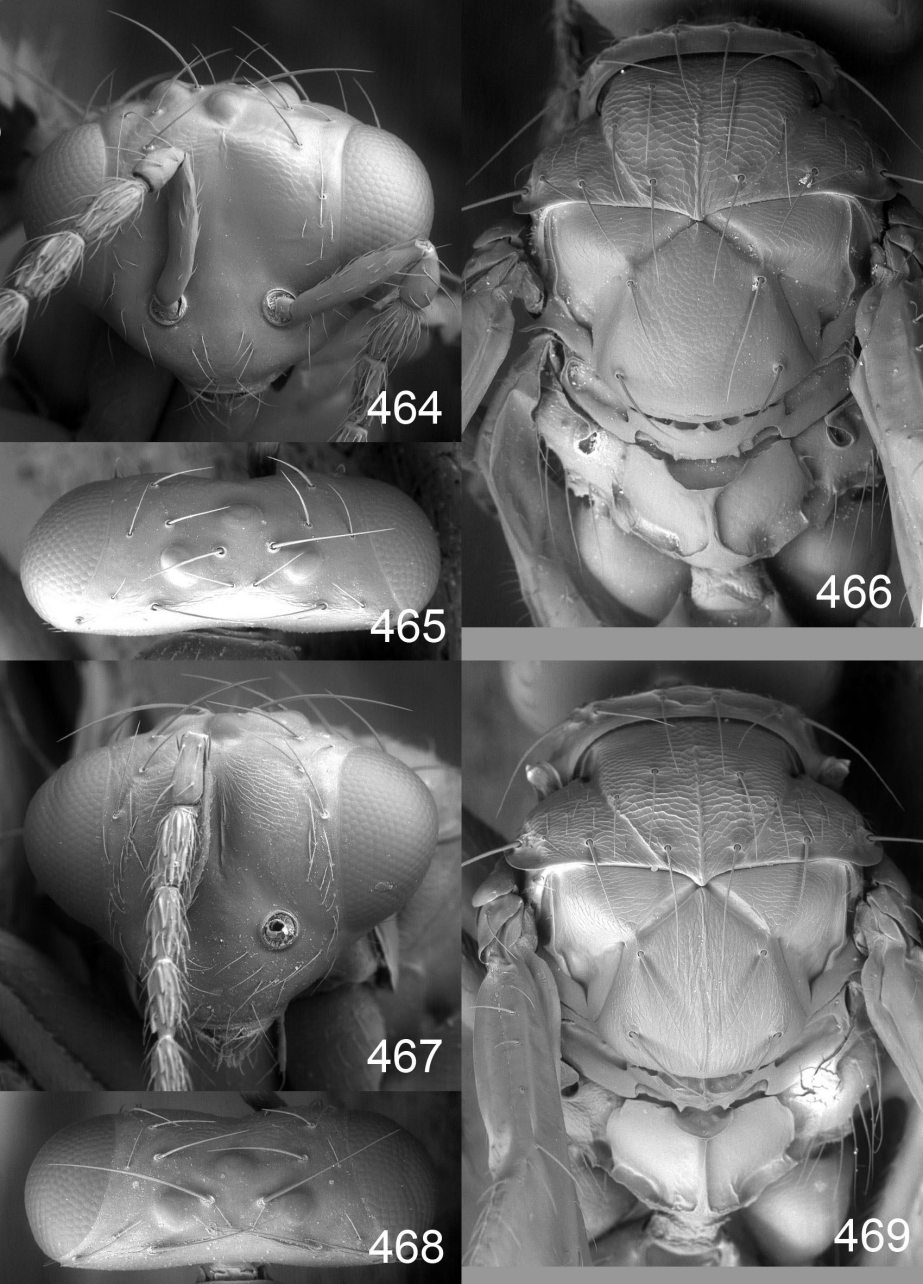
*Euplectrus* spp., females. **464–466** *Euplectrus mikeschauffi* (DHJPAR0045452): **464** head in frontal view **465** vertex **466** mesosoma in dorsal view; **467–469** *Euplectrus mikesharkeyi* (DHJPAR0030516): **467** head in frontal view **468** vertex **469** mesosoma in dorsal view.

*Female*. Length of body 1.9 mm. Antenna with scape yellowish-white, pedicel yellowish-brown, flagellomere 1 pale brown, 2–6 dark brown (Fig. 458). Mandibles and palpi yellowish-white. Head black and shiny, lower face medially yellowish-brown, pale area reaching to level of outer lateral margin of toruli, with parts between pale area and eyes black (Fig. 454). Frons with two irregular rows of setae (Fig. 464). Vertex smooth (Fig. 465). Occipital margin rounded (Fig. 465).

Mesosoma black and shiny (Fig. 453). Each sidelobe of mesoscutum with 11 setae. Scutellum 0.9× as long as wide; with rather weak engraved reticulation (Fig. 466). Dorsellum along anterior margin with a row of foveae (Fig. 772). Propodeum smooth (Fig. 772); anteromedially with a semicircular cup; propodeal callus with eight setae. Legs (Fig. 453): fore and mid coxae yellowish-white, remaining parts of fore and mid legs and entire hind leg yellowish-brown. Fore wing: costal cell with two irregular rows of setae on ventral surface, and margin with four setae close to marginal vein; with 21 admarginal setae, in basal ½ in one row and in apical ½ in two rows.

Gaster in anterior ½ with median part yellowish-white and lateral parts black, posterior ½ black (Fig. 456).

Ratios. HE/MS/WM = 1.9/1.1/1.0; POL/OOL/POO = 6.7/3.8/1.0; OOL/DO = 1.4; WE/WF/WH/HH = 1.0/2.7/4.8/3.6; WH/WT = 1.1; PM/ST = 2.1; TS1/TS2/LT/LT1/LT2/LT3/LT4 = 4.3/2.8/7.3/2.0/1.8/1.0/2.0; LP/WP = 1.0; MM/LG = 1.1.

*Male*. Length of body 1.6 mm. Scape white, enlarged and widest in the middle (Fig. 459), sensory pores confined to apicolateral ¾ on outside facing surface, sensory area white. Similar to female except shorter petiole and gaster.

Ratios. LC/WS = 2.5; LP/WP = 0.8; MM/LG = 1.3.

##### Hosts and biology.

Feeding on third instar larva of *Enyo ocypete* (Sphingidae) feeding on *Tetracera hydrophila* and *Davilla nitida* (Dilleniaceae), parasitoid cocoons stuck to dead larva and substrate.

##### Distribution.

Costa Rica (Guanacaste Province).

##### Etymology.

This species is named after Mike E. Schauff, in recognition of his contribution to the understanding of ACG Hymenoptera taxonomy.

#### Euplectrus mikesharkeyi

Taxon classificationAnimaliaHymenopteraEulophidae

Hansson sp. n.

http://zoobank.org/0802EC27-24B5-4230-8568-873C230850E2

Figures 19 , 467–469 , 470–476 , 773

##### Material.

Holotype a female labeled “COSTA RICA: Guanacaste, ACG, Sector El Hacha, Estación Los Almendros, 11.ix.2008, R. Moraga, ex *Perigea* berindaDHJ02 eating *Lepidaploa tortuosa*, sibling of wasp DHJPAR0030516, 08-SRNP-23276” (BMNH). PARATYPES: 19♀ 4♂: COSTA RICA (ACG): **Guanacaste:** 2♀ 3♂ with same label data as holotype (BMNH, INBio); Sector Pitilla: Pasmompa, 28.i.2006, M. Rios, ex *Perigea* berindaDHJ02 eating *Lepidaploa tortuosa*, sibling of wasp DHJPAR0028869, 06-SRNP-30731 (4♀, in CNC, USNM); Sector Cacao: Sendero a Maritza, D. Garcia, 1.ix.2010, ex *Perigea micrippia* eating *Lepidaploa tortuosa*, sibling of wasp DHJPAR0042134, 10-SRNP-36122 (10♀ 1♂, in BMNH, INBio, MZLU, USNM); Sendero a Maritza, 22.ix.2010, D. Garcia, ex *Perigea micrippia* eating *Lepidaploa tortuosa*, sibling of wasp DHJPAR0042131, 10-SRNP-36497 (3♀, in CNC, INBio).

##### Diagnosis.

**Figures 470–478.**
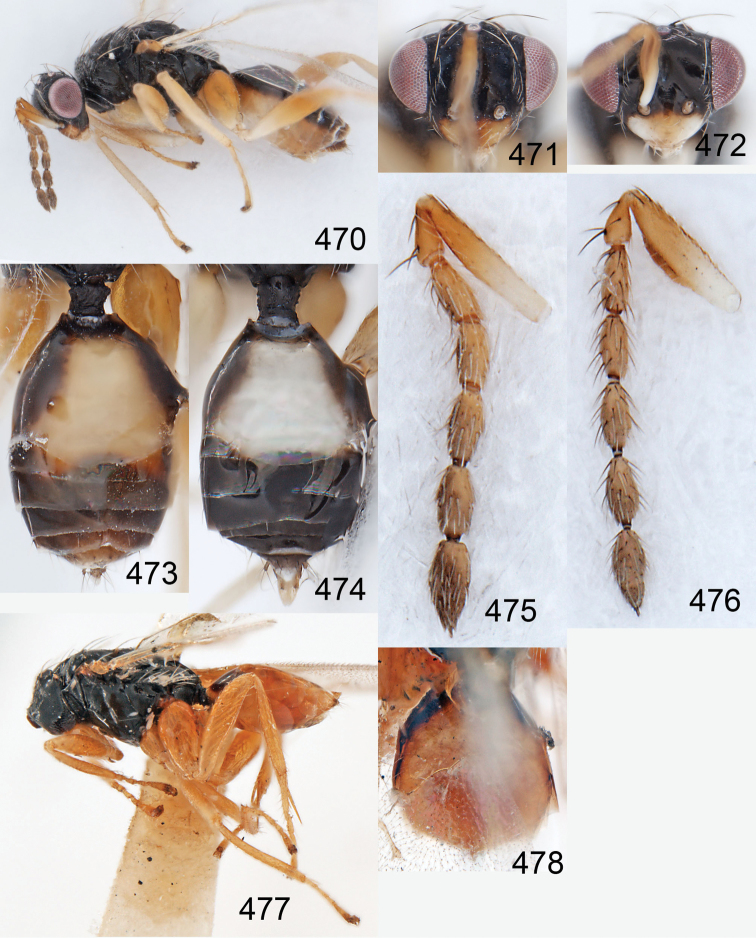
*Euplectrus* spp. **470–476** *Euplectrus mikesharkeyi* (DHJPAR0030516): **470** habitus in lateral view, female **471** head in frontal view, female **472** head in frontal view, male **473** gaster in dorsal view, female **474** gaster in dorsal view, male **475** antenna in lateral view, female **476** antenna in lateral view, male; **477–478** *Euplectrus semimarginatus* (lectotype), female: **477** habitus in lateral view, head missing **478** gaster in dorsal view.

Lower face medially yellowish-brown with median part pale brown (female, Fig. 471), or white with median part yellowish-brown (male, Fig. 472), pale area reaching slightly outside of level of lateral margins of toruli; scutellum reticulate with distinctly elongate meshes and medially with several short longitudinal carinae (Fig. 469); dorsellum anteriorly with two large foveae (alternately with a wide groove that is divided by a median carina) (Fig. 773); legs yellowish-brown with hind coxa and hind femur darker (Fig. 470), male with fore and mid coxae yellowish-white; petiole 0.8× as long as wide; female gaster with anterior ½ yellowish-white with dark brown lateral margins, posterior ½ dark brown with apex reddish-brown (Fig. 473), male gaster with anterior ½ white and with posterior ½ completely dark brown (Fig. 474); male antenna with scape slightly expanded and widest slightly above the middle, 3.3× as long as wide (Fig. 476), with sensory area brown.

##### Description.

*Female*. Length of body 2.4 mm. Antenna with scape yellowish-brown with base yellowish-white, pedicel yellowish-brown, flagellomeres 1–3 yellowish-brown ventrally and dark brown dorsally, 4–6 dark brown (Fig. 475). Mandibles and palpi yellowish-white. Head black and shiny, lower face medially yellowish-brown with median part pale brown, pale area reaching slightly outside of level of lateral margins of toruli, parts between pale area and eyes black (Fig. 471). Frons close to eyes with two rows of setae (Fig. 467). Vertex smooth (Fig. 468). Occipital margin rounded (Fig. 468).

Mesosoma black and shiny (Fig. 470). Each sidelobe of mesoscutum with 11 setae. Scutellum 0.9× as long as wide; with weak engraved reticulation with distinctly elongate meshes; medially with several short longitudinal carinae (Fig. 469). Dorsellum anteriorly with two large foveae (alternately with a wide groove that is divided by a median carina) (Fig. 773). Propodeum with very weak reticulation (Fig. 773); anteromedially with a semicircular cup; propodeal callus with 11 setae. Legs yellowish-brown with hind coxa and hind femur darker (Fig. 470). Fore wing: costal cell on ventral surface with one row of setae, and margin without setae; with 17 admarginal setae, in one row.

Gaster with anterior ½ yellowish-brown with dark brown lateral margins, posterior ½ dark brown with apex reddish-brown (Fig. 473).

Ratios. HE/MS/WM = 2.0/1.0/1.2; POL/OOL/POO = 5.1/2.5/1.0; OOL/DO = 1.3; WE/WF/WH/HH = 1.0/2.6/4.7/3.6; WH/WT = 1.1; PM/ST = 1.4; TS1/TS2/LT/LT1/LT2/LT3/LT4 = 4.2/2.7/7.1/2.6/1.5/1.0/1.9; LP/WP = 0.8; MM/LG = 1.1.

*Male*. Length of body 2.0 mm. Scape slightly expanded and widest slightly above the middle (Fig. 476), sensory pores confined to apicoventral ½ and this area is brown. Otherwise similar to female except lower face with pale parts paler (Fig. 472), fore and mid coxae yellowish-white; gaster shorter, with anterior ½ white and entire posterior ½ dark brown (Fig. 474).

Ratios. LC/WS = 3.3; MM/LG = 1.3.

##### Hosts and biology.

Feeding on penultimate instar larva of *Perigea* berindaDHJ02 and *Perigea micrippia* (Noctuidae), both feeding on *Lepidaploa tortuosa* (Asteraceae), parasitoid cocoons stuck to dead larva and substrate.

##### Distribution.

Costa Rica (Guanacaste Province).

##### Etymology.

This species is named after Mike J. Sharkey, in recognition of his contribution to the understanding of ACG Hymenoptera taxonomy.

#### Euplectrus ninazitaniae

Taxon classificationAnimaliaHymenopteraEulophidae

Hansson sp. n.

http://zoobank.org/DD0FFD99-9ACC-4FAE-9BD8-94ABE2CBBE72

Figures 281–284 , 288–290 , 774

##### Material.

Holotype a female labeled “COSTA RICA: Guanacaste, ACG, Sector Pitilla, Sendero Cuestona, 8.ii.2004, M. Rios, ex *Antapistis* Poole10 eating *Anthurium consobrinum*, sibling of wasp DHJPAR0028735, 04-SRNP-30603” (BMNH). PARATYPES: 3♀ with same label data as holotype (BMNH, INBio).

##### Diagnosis.

Lower face black (Fig. 282); antenna long and slender (Fig. 284), flagellum 3.3× as long as height of eye; mandibles dark brown; dorsellum anteromedially with two large foveae (Fig. 774); legs yellowish-brown with fore and mid coxae yellowish-white (Fig. 281); petiole 1.0× as long as wide; gaster dark brown, anterior ½ with a large yellowish-brown T-shaped spot and with base of T pointed (Fig. 283).

##### Description.

*Female*. Length of body 3.1 mm. Antenna long and slender (Fig. 284), flagellum 3.3× as long as height of eye; scape yellowish-white with apex yellowish-brown, pedicel yellowish-brown, flagellum dark brown. Mandibles dark brown, palpi yellowish-white. Head including lower face black and shiny (Fig. 282). Frons close to eyes with one row of setae (Fig. 288). Vertex smooth (Fig. 289). Occipital margin rounded (Fig. 289).

Mesosoma black and shiny (Fig. 281). Each sidelobe of mesoscutum with seven setae. Scutellum 0.9× as long as wide; with weak engraved reticulation (Fig. 290). Dorsellum anteromedially with two large foveae (Fig. 774). Propodeum smooth (Fig. 774); anteromedially with a triangular cup; propodeal callus with seven setae. Legs yellowish-brown with fore and mid coxae yellowish-white (Fig. 281). Fore wing: costal cell with two rows of setae on ventral surface, and margin with eight setae close to marginal vein; with 14 admarginal setae, in one row.

Gaster dark brown, anterior ½ with a large yellowish-brown T-shaped spot and with base of T pointed (Fig. 283).

Ratios. HE/MS/WM = 1.9/1.0/1.3; POL/OOL/POO = 4.4/2.7/1.0; OOL/DO = 1.0; WE/WF/WH/HH = 1.0/2.2/4.4/3.2; WH/WT = 1.0; PM/ST = 1.7; TS1/TS2/LT/LT1/LT2/LT3/LT4 = 3.6/2.2/5.8/1.6/1.4/1.0/1.8; LP/WP = 1.0; MM/LG = 1.2.

*Male*. Unknown.

##### Hosts and biology.

Feeding on last instar larva of *Antapistis* Poole10 (Erebidae) feeding on *Anthurium consobrinum* (Araceae), parasitoid cocoons stuck to dead larva and substrate.

##### Distribution.

Costa Rica (Guanacaste Province).

##### Etymology.

This species is named after Nina M. Zitani, in recognition of her contribution to the understanding of ACG Hymenoptera taxonomy.

#### Euplectrus pammitchellae

Taxon classificationAnimaliaHymenopteraEulophidae

Hansson sp. n.

http://zoobank.org/6E194029-12DF-46C1-A46E-E0F544994CA5

Figures 298–301 , 305–307 , 775

##### Material.

Holotype a female labeled “COSTA RICA: Guanacaste, ACG, Sector Pitilla, Leonel, 3.i.2010, R. Calero, ex *Bagisara pacifica* eating *Sida glomerata*, sibling of wasp DHJPAR0038580, 10-SRNP-70029” (BMNH). PARATYPES: 4♀ with same label data as holotype (BMNH, INBio).

##### Diagnosis.

Lower face with median part yellowish-brown medially and yellowish-white laterally, pale area reaching slightly outside of level of lateral margins of toruli (Fig. 299); fore coxa yellowish-white, remaining parts of fore leg and entire mid and hind legs yellowish-brown (Fig. 298); dorsellum with a wide groove along anterior margin, groove is medially 0.5× as long as length of dorsellum (Fig. 775); petiole 0.7× as long as wide; gaster with anterior ½ yellowish-brown with dark brown lateral margins, posterior ½ dark brown with tergites 5–6 reddish-brown (Fig. 300).

##### Description.

*Female*. Length of body 2.3 mm. Antenna with scape and pedicel yellowish-brown, flagellomeres 1–4 dark brown with ventral part yellowish-brown, 5–6 dark brown (Fig. 301). Mandibles and palpi white. Head black and shiny, lower face with median part yellowish-brown medially and yellowish-white laterally, pale area reaching slightly outside of level of lateral margins of toruli, parts between pale area and eyes black (Fig. 299). Frons close to eyes with two irregular rows of setae (Fig. 305). Vertex smooth (Fig. 306). Occipital margin with a weak carina (Fig. 306).

Mesosoma black and shiny (Fig. 298). Each sidelobe of mesoscutum with 17 setae. Scutellum 1.0× as long as wide, with very weak reticulation (Fig. 307). Dorsellum along anterior margin with a wide groove that is divided by longitudinal carinae (Fig. 775), groove medially 0.5× as long as length of dorsellum. Propodeum smooth (Fig. 775); anteromedially with a semicircular cup; propodeal callus with ten setae. Legs (Fig. 298): fore coxa yellowish-white, remaining parts of fore leg and entire mid and hind legs yellowish-brown. Fore wing: submarginal vein with four setae; costal cell with two irregular and sparse rows of setae on ventral surface, and margin with two setae close to marginal vein; with 13 admarginal setae, in one row.

Gaster with anterior ½ yellowish-brown with dark brown lateral margins, posterior ½ dark brown with tergites 5–6 reddish-brown (Fig. 300).

Ratios. HE/MS/WM = 2.0/1.0/1.2; POL/OOL/POO = 5.9/3.1/1.0; OOL/DO = 1.2; WE/WF/WH/HH = 1.0/2.4/4.5/3.5; WH/WT = 1.1; PM/ST = 1.6; TS1/TS2/LT/LT1/LT2/LT3/LT4 = 3.3/2.0/6.0/1.6/1.2/1.0/2.0; LP/WP = 0.7; MM/LG = 1.0.

*Male*. Unknown.

##### Hosts and biology.

Feeding on penultimate instar larva of *Bagisara pacifica* (Noctuidae) feeding on *Sida glomerata* (Malvaceae), parasitoid cocoons stuck to dead larva and substrate.

##### Distribution.

Costa Rica (Guanacaste Province).

##### Etymology.

This species is named after Pamela A. Mitchell, in recognition of her contribution to the understanding of ACG Hymenoptera taxonomy.

#### Euplectrus paulhansoni

Taxon classificationAnimaliaHymenopteraEulophidae

Hansson sp. n.

http://zoobank.org/086DB9E2-ED15-4C1D-ADCE-12749CDF19FF

Figures 479–485 , 489–491 , 776

##### Material.

Holotype a female labeled “ COSTA RICA: Guanacaste, ACG, Sendero Cuestona, 8.viii.2004, C. Moraga, ex *Rejectaria splendida* eating *Asplundia microphylla*, sibling of wasp DHJPAR0028775, 04-SRNP-34368” (BMNH). Paratypes: 17♀ 5♂: COSTA RICA: **Alajuela:** San Cristobal, LN 318056/383200, 600-620 m, 23.i–23.ii.1996, malaise trap, F.A. Quesada (1♀, in INBio), 23.ii-25.ii.1996 (1♀, in BMNH), 25.ii–16.iv.1996 (1♀, in INBio); **Guanacaste:** 4♀ 4♂ with same label data as holotype (BMNH, CNC, INBio, USNM); Chon, 25.xi.2009, E. Cantillano, ex *Rejectaria* Janzen06 eating *Bolbitis portoricensis*, 09-SRNP-23523, sibling of wasp DHJPAR0038561 (1♀ 1♂, in MZLU); Finca Marcos Morales, 1100-1200 m, LN 317750/594800, 28.vi-5.vii.1995, M. Segura, malaise trap (2♀, in INBio); **Heredia:** 3 km S Puerto Viejo, OTS-La Selva, 100 m, 2.i.1996, malaise trap (1♀, in INBio); **Puntarenas:** Monteverde, 1000-1350 m, LN 250850/449250, Z. Fuentes, malaise trap, 3-31.i.1993 (2♀, in BMNH), ii.1993 (2♀, in INBio), vii.1993 (2♀, in CNC, USNM).

##### Diagnosis.

**Figures 479–488.**
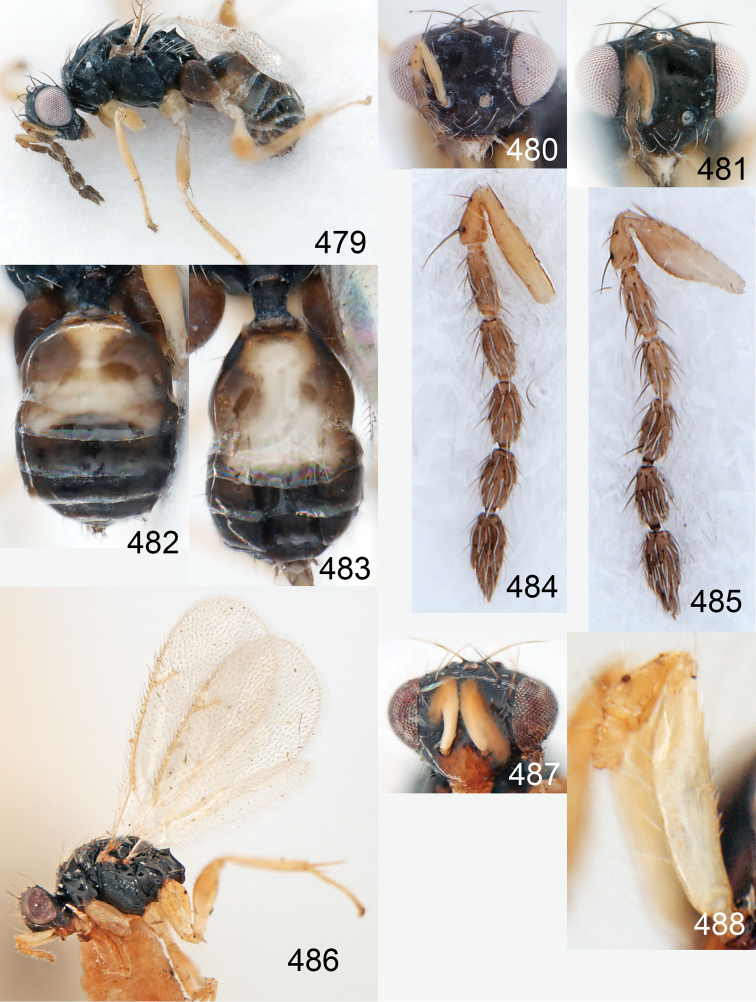
*Euplectrus* spp. **479–485** *Euplectrus paulhansoni* (DHJPAR0028775): **479** habitus in lateral view, female **480** head in frontal view, female **481** head in frontal view, male **482** gaster in dorsal view, female **483** gaster in dorsal view, male **484** antenna in lateral view, female **485** antenna in lateral view, male; **486–488** *Euplectrus solitarius* (holotype), female: **486** habitus in lateral view, gaster missing **487** head in frontal view **488** scape and pedicel in lateral view.

Lower face black (Fig. 480); mandibles dark yellowish-brown to brown; fore and mid legs yellowish-white, hind leg with coxa dark brown, femur, tibia and tarsus yellowish-brown (Fig. 479); petiole 1.0× as long as wide; gaster with anterior ½ dark brown with a white marking shaped like an hour-glass with distal part wider than basal part, posterior ½ dark brown (Figs 482, 483); male scape slightly enlarged (Fig. 485), widest medially, 3.3× as long as wide.

##### Description.

**Figures 489–494.**
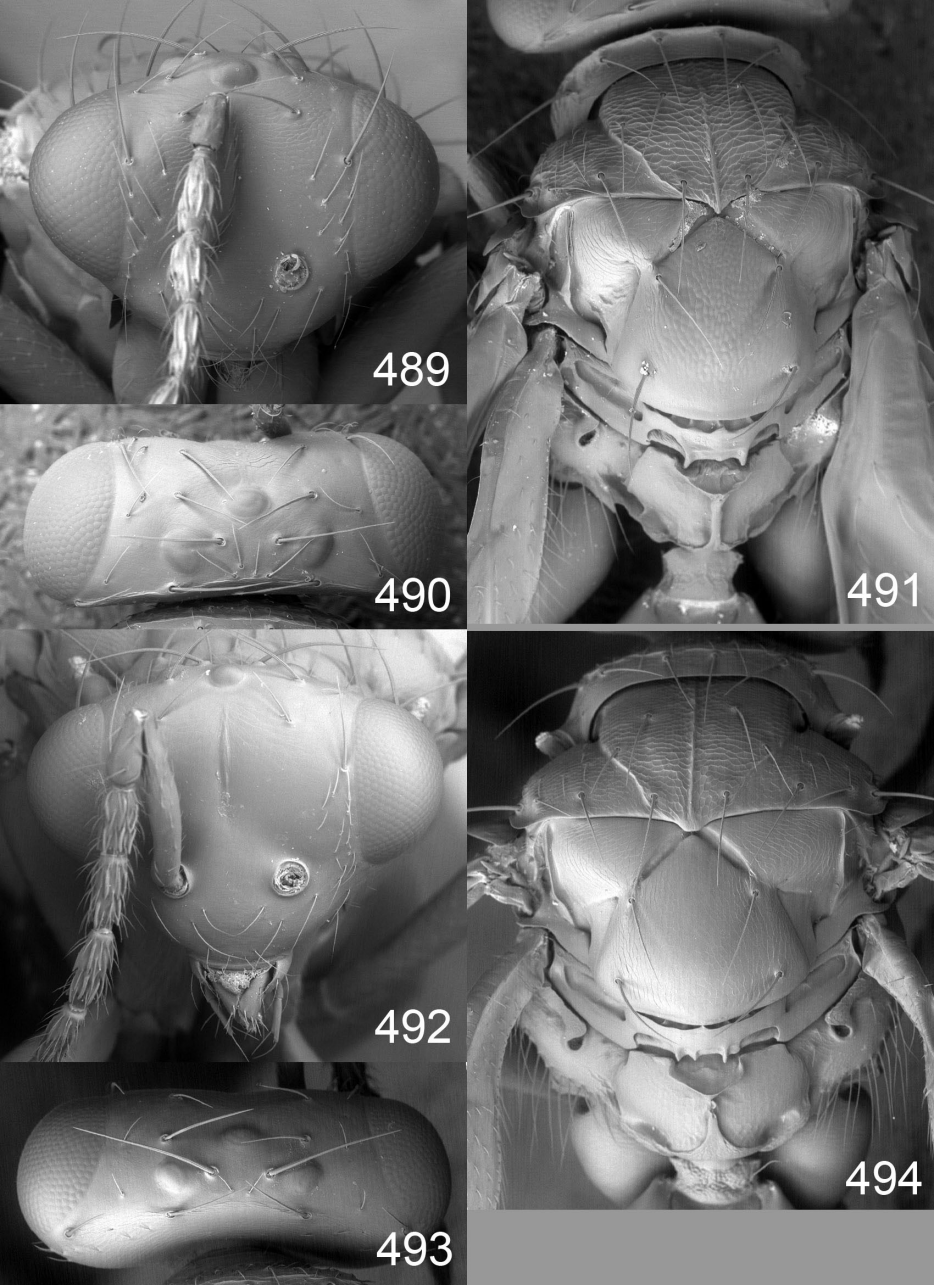
*Euplectrus* spp., females. **489–491** *Euplectrus paulhansoni* (DHJPAR0028775): **489** head in frontal view **490** vertex **491** mesosoma in dorsal view; **492–494** *Euplectrus platyhypenae* (non-types): **492** head in frontal view **493** vertex **494** mesosoma in dorsal view.

*Female*. Length of body 2.3 mm. Antenna with scape and pedicel yellowish-brown, flagellum dark brown (Fig. 484). Mandibles dark yellowish-brown to brown and palpi yellowish-white. Head including lower face black and shiny (Fig. 480). Frons close to eyes with two rows of setae (Fig. 489). Vertex with very weak reticulation (Fig. 490). Occipital margin with a carina behind ocellar triangle (Fig. 490).

Mesosoma black and shiny (Fig. 479). Scutellum 0.9× as long as wide; with very weak reticulation, median part partly smooth (Fig. 491). Dorsellum along anterior margin with a deep groove that is divided by three weak longitudinal carinae (Fig. 776), groove medially 0.3× as long as length of dorsellum. Propodeum smooth and shiny (Fig. 776); anteromedially with a semicircular cup that is strongly raised in posterior part; propodeal callus with six setae. Legs (Fig. 479): fore and mid legs yellowish-white; hind leg with coxa dark brown, femur, tibia and tarsus yellowish-brown. Fore wing: costal cell with one complete row of setae on ventral surface, and margin with seven setae in apical ½; with 23 admarginal setae, in basal ½ in one row in apical ½ in two rows.

Gaster with anterior ½ dark brown with a white marking shaped like an hour-glass with distal part wider than basal part, posterior ½ dark brown (Fig. 482).

Ratios. HE/MS/WM = 2.1/1.1/1.0; POL/OOL/POO = 9.0/5.8/1.0; OOL/DO = 1.2; WE/WF/WH/HH = 1.0/2.6/4.7/3.6; WH/WT = 1.1; PM/ST = 1.7; TS1/TS2/LT/LT1/LT2/LT3/LT4 = 4.0/2.2/6.4/2.0/2.3/1.0/2.0; LP/WP = 1.0; MM/LG = 1.1.

*Male*. Length of body 1.9 mm. Scape yellowish-brown, slightly enlarged, widest medially, with sensory pores limited to apical ⅔ of ventral margin, sensory area yellowish-brown (Fig. 485). Similar to female except shorter gaster.

Ratios. LC/WS = 3.3; MM/LG = 1.2.

##### Hosts and biology.

Feeding on penultimate instar larvae of *Rejectaria splendida* (Erebidae) feeding on *Asplundia microphylla* (Cyclanthaceae), *Rejectaria* Janzen06 feeding on *Bolbitis portoricensis* (Lomariopsidaceae), parasitoid cocoons stuck to dead larva and substrate.

##### Distribution.

Costa Rica (Alajuela, Guanacaste, Heredia and Puntarenas Provinces).

##### Etymology.

This species is named after Paul E. Hanson, in recognition of his contribution to the understanding of ACG Hymenoptera taxonomy.

#### Euplectrus paulheberti

Taxon classificationAnimaliaHymenopteraEulophidae

Hansson sp. n.

http://zoobank.org/4A656869-7629-4DB1-9CDA-87E71EF46AE1

Figures 5 , 495–501 , 506–508 , 582 , 777

##### Material.

Holotype a female labeled “COSTA RICA: Guanacaste, ACG, Sector Santa Rosa, Area Administrativa, 8.x.2013, W. Hallwachs, ex *Aellopos clavipes* eating *Randia aculeata*, sibling of wasp DHJPAR0053133, 13-SRNP-10107” (BMNH). Paratypes: 8♀ 2♂ with same label data as holotype (BMNH, CNC, INBio, MZLU, USNM).

##### Diagnosis.

**Figures 495–505.**
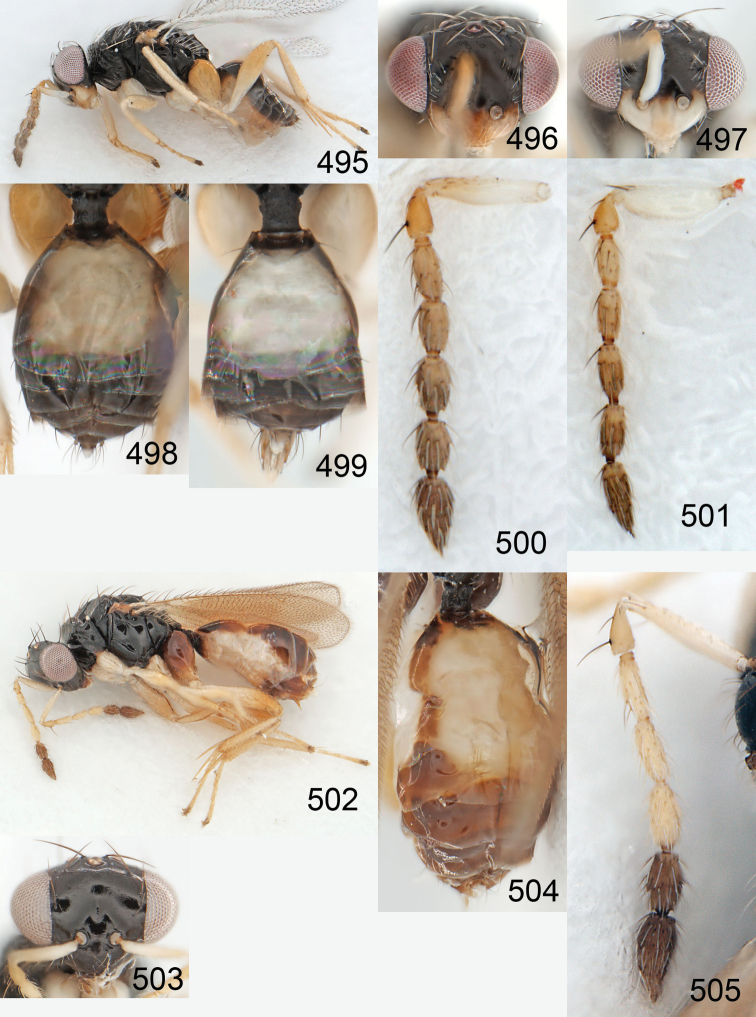
*Euplectrus* spp. **495–501** *Euplectrus paulheberti* (DHJPAR0053133): **495** habitus in lateral view, female **496** head in frontal view, female **497** head in frontal view, male **498** gaster in dorsal view, female **499** gaster in dorsal view, male **500** antenna in lateral view, female **501** antenna in lateral view, male; **502–505** *Euplectrus zamorai* (non-types), female: **502** habitus in lateral view **503** head in frontal view **504** gaster in dorsal view **505** antenna in lateral view.

Lower face completely pale, in female medially yellowish-brown and laterally yellowish-white (Fig. 496), in male white (Fig. 497); fore and mid legs yellowish-white; hind leg yellowish-brown (Fig. 495); petiole 1.0× as long as wide in female, 0.8× in male; gaster with anterior ½ yellowish-white with lateral margins dark brown posterior ½ dark brown (Fig. 498); male scape slightly enlarged (Fig. 501), widest medially, 2.9× as long as wide.

##### Description.

**Figures 506–511.**
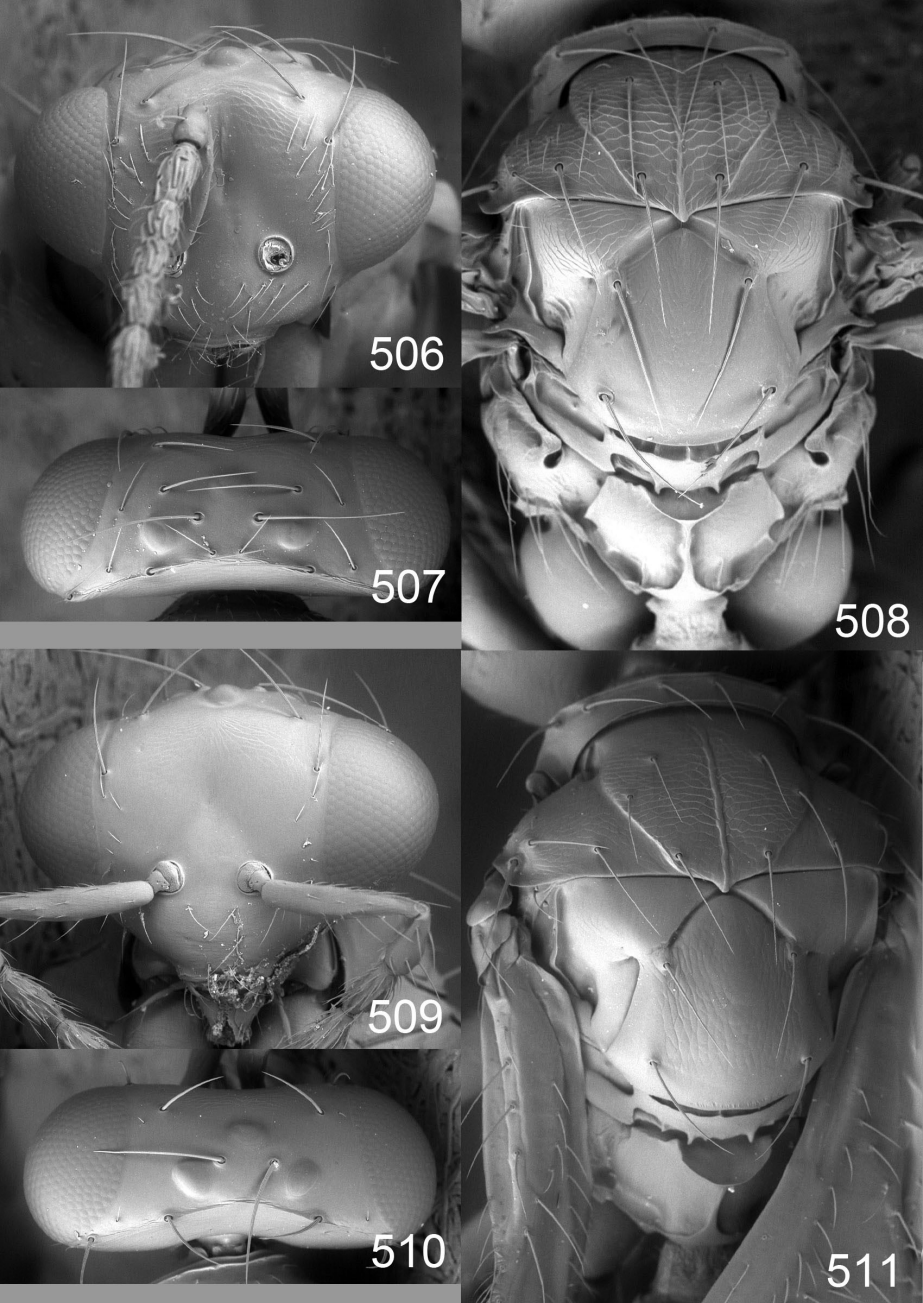
*Euplectrus* spp., females. **506–508** *Euplectrus paulheberti* (DHJPAR0053133): **506** head in frontal view **507** vertex **508** mesosoma in dorsal view; **509–511** *Euplectrus zamorai* (non-types): **509** head in frontal view **510** vertex **511** mesosoma in dorsal view.

*Female*. Length of body 1.8 mm. Antenna with scape yellowish-white, pedicel and flagellomeres 1–2 yellowish-brown, flagellomeres 3–6 dark brown (Fig. 500). Mandibles and palpi white. Head black and shiny, lower face completely pale, medially yellowish-brown and laterally yellowish-white (Fig. 496). Frons close to eyes with a row of setae (Fig. 506). Vertex with very weak reticulation (Fig. 507). Occipital margin with a carina behind ocellar triangle (Fig. 507).

Mesosoma black and shiny (Fig. 495). Scutellum 1.0× as long as wide; with very weak reticulation (Fig. 508). Dorsellum along anterior margin with a deep groove that is divided by longitudinal carinae (Fig. 777), groove medially 0.4× as long as length of dorsellum. Propodeum with very weak reticulation partly smooth (Fig. 777); anteromedially with a semicircular cup that is strongly raised in posterior part; propodeal callus with eight setae. Legs (Fig. 495): fore and mid legs yellowish-white; hind leg yellowish-brown. Fore wing: costal cell with one row of setae on ventral surface, and margin with two setae close to marginal vein; with 14 admarginal setae.

Gaster with anterior ½ yellowish-white with lateral margins dark brown posterior ½ dark brown (Fig. 498).

Ratios. HE/MS/WM = 2.1/1.0/1.3; POL/OOL/POO = 5.9/3.0/1.0; OOL/DO = 1.3; WE/WF/WH/HH = 1.0/2.3/4.3/3.0; WH/WT = 1.2; PM/ST = 1.5; TS1/TS2/LT/LT1/LT2/LT3/LT4 = 4.7/3.8/8.4/3.3/1.8/1.0/1.8; LP/WP = 1.0; MM/LG = 1.3.

*Male*. Length of body 1.5 mm. Scape slightly enlarged, widest medially, with sensory pores limited to apical ⅔ of ventral margin, sensory area yellowish-white (Fig. 501). Similar to female except lower face white (Fig. 497), and shorter petiole and gaster.

Ratios. LC/WS = 2.9; LP/WP = 0.8; MM/LG = 1.5.

##### Hosts and biology.

Feeding on second instar larva of *Aellopos clavipes* (Sphingidae) feeding on *Randia aculeata* (Rubiaceae), parasitoid cocoons stuck to dead larva and substrate.

##### Distribution.

Costa Rica (Guanacaste Province).

##### Etymology.

This species is named after Paul D. N. Hebert, in recognition of his contribution to the understanding of ACG Hymenoptera taxonomy.

#### Euplectrus paulhurdi

Taxon classificationAnimaliaHymenopteraEulophidae

Hansson sp. n.

http://zoobank.org/2F903136-7A7E-40EF-962F-442F24678F67

Figures 512–518 , 526–528 , 778

##### Material.

Holotype a female labeled “COSTA RICA: Guanacaste, ACG, Sector Cacao, Quebrada Otilio, 7.xi.2066, D. Garcia, ex *Tagela cayuga* eating *Cupania guatemalensis*, sibling of wasp DHJPAR0028878, 06-SRNP-48034” (BMNH). PARATYPES: 1♀ 2♂ with same label data as holotype (BMNH, INBio).

##### Diagnosis.

**Figures 512–525.**
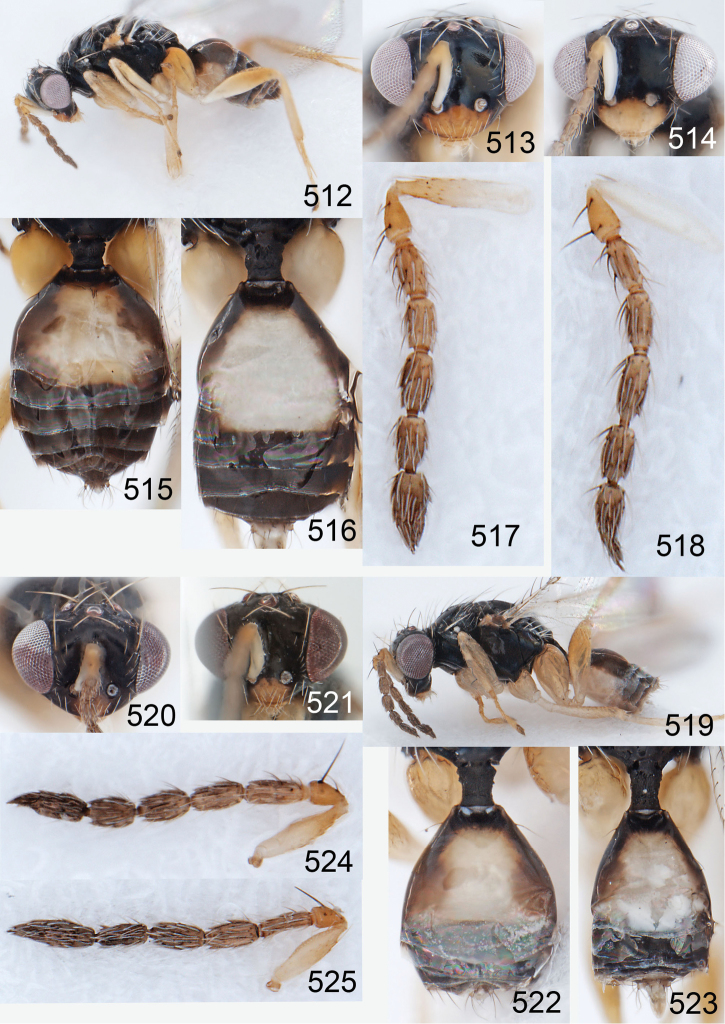
*Euplectrus* spp. **512–518** *Euplectrus paulhurdi* (DHJPAR0028878): **512** habitus in lateral view, female **513** head in frontal view, female **514** head in frontal view, male **515** gaster in dorsal view, female **516** gaster in dorsal view, male **517** antenna in lateral view, female **518** antenna in lateral view, male; **519–525** *Euplectrus philwardi* (DHJPAR0028876): **519** habitus in lateral view, female **520** head in frontal view, female **521** head in frontal view, male **522** gaster in dorsal view, female **523** gaster in dorsal view, male **524** antenna in lateral view, female **525** antenna in lateral view, male.

Lower face medially yellowish-brown (female, Fig. 513) or white (male, Fig. 514), pale area reaching slightly outside of level of outer margins of toruli; female with legs yellowish-brown with fore and mid coxae yellowish-white (Fig. 512), male legs yellowish-white with hind coxa and hind femur slightly darker; petiole 0.8× as long as wide in female, 1.0× in male; gaster dark brown, anterior ½ with a large yellowish-brown (female, Fig. 515) or white (male, Fig. 516) spot; male antenna with scape slightly expanded and widest in the middle, 3.3× as long as wide (Fig. 518).

##### Description.

**Figures 526–531.**
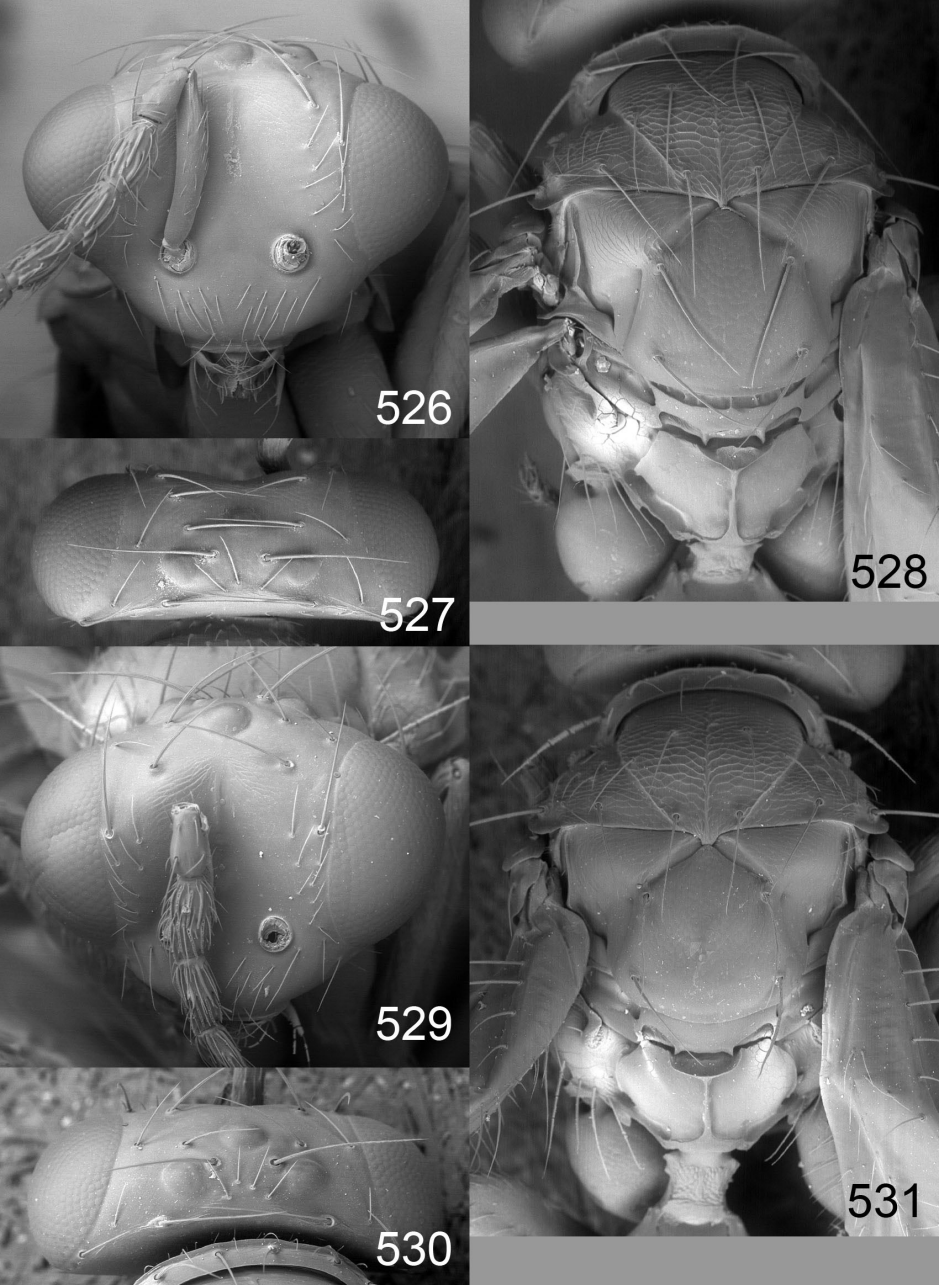
*Euplectrus* spp., females. **526–528** *Euplectrus paulhurdi* (DHJPAR0028878): **526** head in frontal view **527** vertex **528** mesosoma in dorsal view; **529–531** *Euplectrus philwardi* (DHJPAR0028876): **529** head in frontal view **530** vertex **531** mesosoma in dorsal view.

*Female*. Length of body 1.9 mm. Antenna with scape yellowish-brown with base yellowish-white, pedicel yellowish-brown, flagellomeres 1–2 yellowish-brown, 3 yellowish-brown ventrally and dark brown dorsally, 4–6 dark brown (Fig. 517). Mandibles and palpi yellowish-white. Head black and shiny, lower face medially yellowish-brown with pale area reaching slightly outside of level of outer margins of toruli, part between pale area and eyes black (Fig. 513). Frons close to eyes with two rows of setae (Fig. 526). Vertex with very weak reticulation (Fig. 527). Occipital margin rounded (Fig. 527).

Mesosoma black and shiny (Fig. 512). Each sidelobe of mesoscutum with 13 setae. Scutellum 0.9× as long as wide; with very weak engraved reticulation (Fig. 528). Dorsellum anteriorly with a groove that is divided by longitudinal carinae (Fig. 778), groove medially 0.4× as long as length of dorsellum. Propodeum with very weak reticulation (Fig. 778); anteromedially with a transverse triangular cup; propodeal callus with eight setae. Legs yellowish-brown with fore and mid coxae yellowish-white (Fig. 512). Fore wing: costal cell with two rows of setae on ventral surface, and margin with three setae close to marginal vein; with 14 admarginal setae, in one row.

Gaster dark brown, anterior ½ with a large yellowish-brown spot (Fig. 515).

Ratios. HE/MS/WM = 2.0/1.0/1.3; POL/OOL/POO = 6.2/3.3/1.0; OOL/DO = 1.3; WE/WF/WH/HH = 1.0/2.5/4.5/3.3; WH/WT = 1.1; PM/ST = 1.4; TS1/TS2/LT/LT1/LT2/LT3/LT4 = 4.2/2.4/6.0/2.2/1.3/1.0/1.2; LP/WP = 0.8; MM/LG = 1.2.

*Male*. Length of body 1.9 mm. Scape white and slightly expanded and widest in the middle (Fig. 518), sensory pores confined to apicoventral ¾, sensory area pale as scape. Otherwise similar to female except lower face with pale part yellowish-white (Fig. 514), legs yellowish-white with hind coxa and hind femur slightly darker; petiole longer; gaster shorter and with pale part white (Fig. 516).

Ratios. LC/WS = 3.3; LP/WP = 1.0; MM/LG = 1.3.

##### Hosts and biology.

Feeding on third instar larva of *Tagela cayuga* (Notodontidae) feeding on *Cupania guatemalensis* (Sapindaceae), parasitoid cocoons stuck to dead larva and substrate.

##### Distribution.

Costa Rica (Guanacaste Province).

##### Etymology.

This species is named after Paul D. Hurd, in recognition of his contribution to the understanding of ACG Hymenoptera taxonomy.

#### Euplectrus philwardi

Taxon classificationAnimaliaHymenopteraEulophidae

Hansson sp. n.

http://zoobank.org/28E103E1-42E7-45AA-9275-26B8D295A64A

Figures 519–525 , 529–531 , 779

##### Material.

Holotype a female labeled “COSTA RICA: Alajuela, ACG, Sector San Cristobal, Puente Palma, 25.v.2006, E. Araya, ex *Deinopa signiplena* eating *Swartzia costaricensis*, sibling of wasp DHJPAR0028876, 06-SRNP-4161” (BMNH). PARATYPE: 1♂ with same label data as holotype (BMNH).

##### Diagnosis.

Lower face with median part dark reddish-brown, pale area reaching to level of median toruli (Figs 520, 521); dorsellum without a groove or foveae along anterior margin (Fig. 779); fore and mid legs yellowish-white, hind leg yellowish-brown (Fig. 519); petiole 1.1× as long as wide in female, 1,3× in male; gaster dark brown with a ±T-shaped yellowish-white spot in anterior ½ (female, Fig. 522), or with anterior ½ white with dark brown lateral margins and posterior ½ dark brown (male, Fig. 523); male antenna with scape slightly expanded and widest in the middle, 2.9× as long as wide (Fig. 518).

##### Description.

*Female*. Length of body 1.8 mm. Antenna with scape yellowish-brown with base yellowish-white, pedicel yellowish-brown, flagellomeres 1–2 pale brown, 3–6 dark brown (Fig. 524). Mandibles yellowish-brown, palpi yellowish-white. Head black and shiny, lower face with median part dark reddish-brown, pale area reaching to level of median toruli, with parts between pale area and eyes black (Fig. 520). Frons close to eyes with two rows of setae (Fig. 529). Vertex smooth (Fig. 530). Occipital margin rounded (Fig. 530).

Mesosoma black and shiny (Fig. 519). Each sidelobe of mesoscutum with 12 setae. Scutellum 0.9× as long as wide; with very weak engraved reticulation, posterior margin smooth (Fig. 531). Dorsellum without a groove or foveae along anterior margin (Fig. 779). Propodeum smooth (Fig. 779); anteromedially with a transverse semicircular cup; propodeal callus with six setae. Legs (Fig. 519): fore and mid legs yellowish-white, hind leg yellowish-brown. Fore wing: costal cell with two rows of setae on ventral surface, and margin with six setae close to marginal vein; with 16 admarginal setae, in one row.

Gaster dark brown with a ±T-shaped yellowish-white spot in anterior ½ (Fig. 522).

Ratios. HE/MS/WM = 2.6/1.0/1.3; POL/OOL/POO = 4.4/2.4/1.0; OOL/DO = 1.0; WE/WF/WH/HH = 1.0/2.4/4.5/3.4; WH/WT = 1.1; PM/ST = 1.8; TS1/TS2/LT/LT1/LT2/LT3/LT4 = 4.2/2.4/6.7/2.4/1.4/1.0/1.6; LP/WP = 1.1; MM/LG = 1.4.

*Male*. Length of body 1.6 mm. Scape slightly expanded and widest in the middle (Fig. 525), sensory pores confined to apicoventral ¾, sensory area pale as scape. Otherwise similar to female except petiole longer; gaster with anterior ½ white with dark brown lateral margins and posterior ½ dark brown (Fig. 523).

Ratios. LC/WS = 2.9; LP/WP = 1.3.

##### Hosts and biology.

Feeding on third instar larva of *Deinopa signiplena* (Noctuidae) feeding on *Swartzia costaricensis* (Fabaceae), parasitoid cocoons stuck to dead larva and substrate.

##### Distribution.

Costa Rica (Alajuela Province).

##### Etymology.

This species is named after Phil S. Ward, in recognition of his contribution to the understanding of ACG Hymenoptera taxonomy.

#### Euplectrus robbinthorpi

Taxon classificationAnimaliaHymenopteraEulophidae

Hansson sp. n.

http://zoobank.org/50D76759-FB84-40C2-B0F1-28245498CAB7

Figures 532–538 , 546–548 , 780

##### Material.

Holotype a female labeled “COSTA RICA: Alajuela, ACG, Sector Brasilia, Piedrona, 6.xii.2007, L. Rios, ex *Olethreutes* Brown20 eating *Inga oerstediana*, sibling of wasp DHJPAR0023276, 07-SRNP-66139” (BMNH). PARATYPES: 3♀ 2♂ with same label data as holotype (BMNH, INBio).

##### Diagnosis.

**Figures 532–545.**
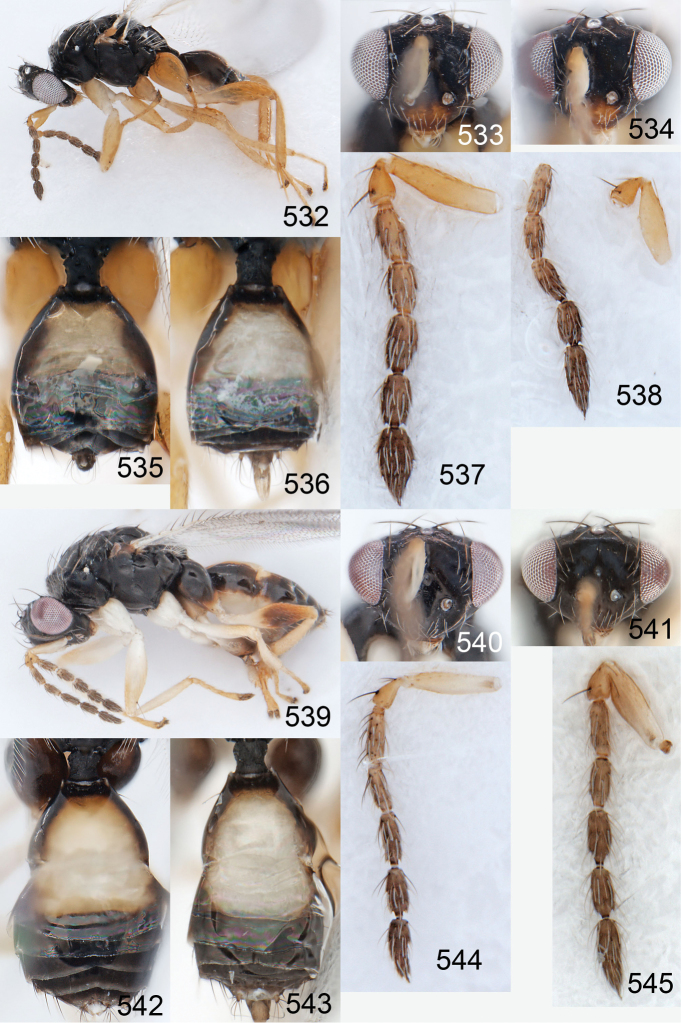
*Euplectrus* spp. **532–538** *Euplectrus robbinthorpi* (DHJPAR0023276): **532** habitus in lateral view, female **533** head in frontal view, female **534** head in frontal view, male **535** gaster in dorsal view, female **536** gaster in dorsal view, male **537** antenna in lateral view, female **538** antenna in lateral view, male **539–545** *Euplectrus scottshawi* (DHJPAR0028877): **539** habitus in lateral view, female **540** head in frontal view, female **541** head in frontal view, male **542** gaster in dorsal view, female **543** gaster in dorsal view, male **544** antenna in lateral view, female **545** antenna in lateral view, male.

Lower face with median part reddish-brown (female, Fig. 533) or yellowish-brown (male, Fig. 534), pale area reaching to level of middle of toruli; legs yellowish-brown with fore coxa yellowish-white (Fig. 532); petiole 0.9× as long as wide; gaster with anterior ½ yellowish-white (female, Fig. 535) or white (male, Fig. 536) with wide dark brown lateral margins, posterior ½ dark brown; male antenna with scape slightly expanded and widest in the middle, 2.9× as long as wide (Fig. 538).

##### Description.

**Figures 546–551.**
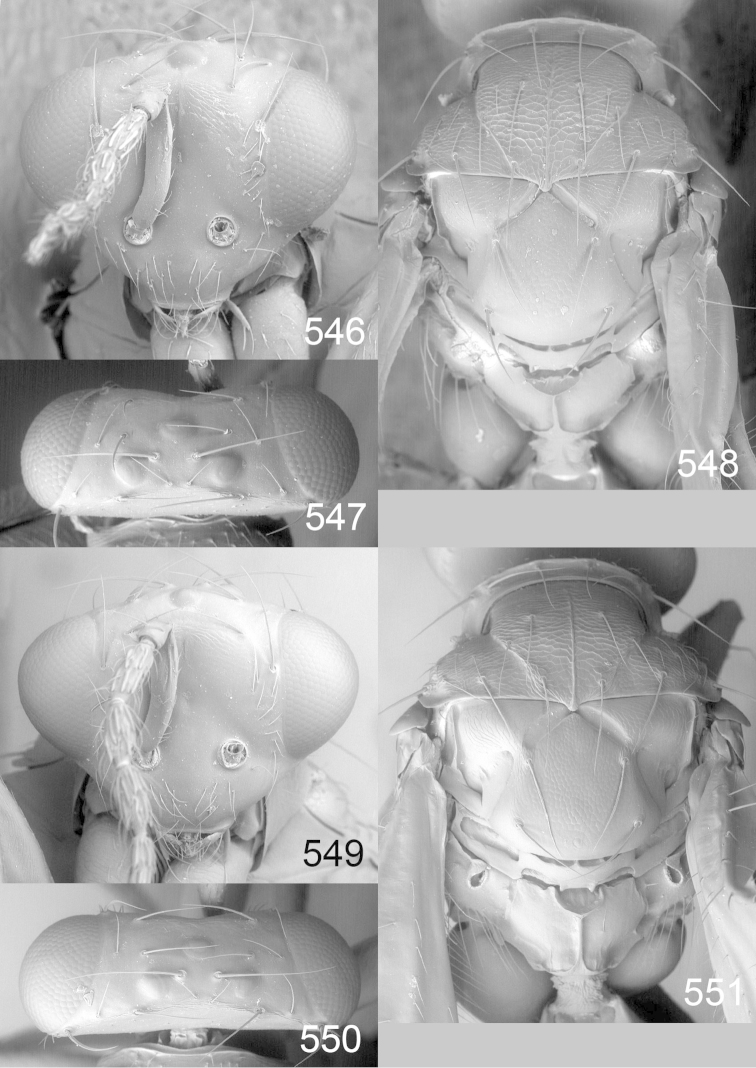
*Euplectrus* spp., females. **546–548** *Euplectrus robbinthorpi* (DHJPAR0023276): **546** head in frontal view **547** vertex **548** mesosoma in dorsal view; **549–551** *Euplectrus scottshawi* (DHJPAR0028877): **549** head in frontal view **550** vertex **551** mesosoma in dorsal view.

*Female*. Length of body 1.9 mm. Antenna with scape and pedicel yellowish-brown, flagellum dark brown (Fig. 537). Mandibles and palpi yellowish-white. Head black and shiny, lower face with median part reddish-brown, pale area reaching to level of middle of toruli, with parts between pale area and eyes black (Fig. 533). Frons close to eyes with one row of setae (Fig. 546). Vertex smooth (Fig. 547). Occipital margin rounded (Fig. 547).

Mesosoma black and shiny (Fig. 532). Each sidelobe of mesoscutum with nine setae. Scutellum 0.9× as long as wide; with weak engraved reticulation, posterior margin smooth (Fig. 548). Dorsellum anteriorly with a narrow groove that is divided by longitudinal carinae (Fig. 780), groove medially 0.2× as long as length of dorsellum. Propodeum with very weak reticulation (Fig. 780); anteromedially with a transverse semicircular cup; propodeal callus with ten setae. Legs yellowish-brown with fore coxa yellowish-white (Fig. 532). Fore wing: costal cell with one row of setae on ventral surface, and margin with three setae close to marginal vein; with 13 admarginal setae, in one row.

Gaster with anterior ½ yellowish-white with wide dark brown lateral margins, posterior ½ dark brown (Fig. 535).

Ratios. HE/MS/WM = 2.2/1.0/1.2; POL/OOL/POO = 8.8/5.8/1.0; OOL/DO = 1.2; WE/WF/WH/HH = 1.0/2.4/4.4/3.5; WH/WT = 1.1; PM/ST = 1.3; TS1/TS2/LT/LT1/LT2/LT3/LT4 = 3.6/2.4/5.6/1.6/1.4/1.0/1.6; LP/WP = 0.9; MM/LG = 1.4.

*Male*. Length of body 1.5 mm. Scape slightly expanded and widest in the middle (Fig. 538), sensory pores confined to apicoventral ¾, sensory area pale as scape. Otherwise similar to female except median part of lower face yellowish-brown (Fig. 534), gaster shorter and with pale part white (Fig. 536).

Ratios. LC/WS = 2.9; MM/LG = 1.6.

##### Hosts and biology.

Feeding on last instar larva of *Olethreutes Brown20* (Tortricidae) feeding on *Inga oerstediana* (Fabaceae), parasitoid cocoons stuck to dead larva and substrate. There remains some doubt as to whether this caterpillar was correctly identified, and only further host records will determine that.

##### Distribution.

Costa Rica (Alajuela Province).

##### Etymology.

This species is named after Robbin W. Thorp, in recognition of his contribution to the understanding of ACG Hymenoptera taxonomy.

#### Euplectrus ronaldzunigai

Taxon classificationAnimaliaHymenopteraEulophidae

Hansson sp. n.

http://zoobank.org/F148EE88-86B6-44EE-A59C-8D7D00F270F8

Figures 315–318 , 322–324 , 781

##### Material.

Holotype a female labeled “COSTA RICA: Guanacaste, ACG, Sector Pitilla, Pasmompa, 15.vii.2005, C. Moraga, ex *Antiblemma* Poole03 eating *Miconia impetiolaris*, sibling of wasp DHJPAR0028824, 05-SRNP-32613” (BMNH). PARATYPES: 7♀: COSTA RICA (ACG): **Guanacaste:** Sector Pitilla, Pasmompa: 2♀ with same label data as holotype (BMNH, INBio); following from same host as holotype but collected 25.vii.2005, sibling of wasp DHJPAR0045454, 11-SRNP-32077 (1♀, INBio) and DHJPAR0045455, 11-SRNP-32079 (1♀, CNC), 25.vii.2011, DHJPAR0045455, 11-SRNP-32079 (3♀, BMNH, MZLU, USNM).

##### Diagnosis.

Lower face black with median part dark reddish-brown, pale area reaching to level of middle of toruli (Fig. 316); dorsellum without a groove or foveae along anterior margin (Fig. 781); legs yellowish-white with hind coxa and apical ¾ of hind femur yellowish-brown (Fig. 315); petiole 1.2× as long as wide; gaster with anterior ½ yellowish-white with dark brown lateral margins, posterior ½ dark brown (Fig. 317).

##### Description.

*Female*. Length of body 2.0 mm. Antenna with scape yellowish-white, pedicel yellowish-brown, flagellomeres 1–3 pale brown, 4–6 dark brown (Fig. 318). Mandibles yellowish-white, palpi white. Head black and shiny, lower face medially dark reddish-brown, pale area reaching to level of middle of toruli (Fig. 316). Frons close to eyes with one row of setae (Fig. 322). Vertex smooth (Fig. 323). Occipital margin with a carina behind ocellar triangle (Fig. 323).

Mesosoma black and shiny (Fig. 315). Each sidelobe of mesoscutum with 11 setae. Scutellum 0.9× as long as wide; with very weak engraved reticulation (Fig. 324). Dorsellum without a groove or foveae along anterior margin (Fig. 781). Propodeum with very weak reticulation (Fig. 781); anteromedially with a very short and wide semicircular cup; propodeal callus with eight setae. Legs yellowish-white with hind coxa and apical ¾ of hind femur yellowish-brown (Fig. 315). Fore wing: submarginal vein with four setae; costal cell with one row of setae on ventral surface, and margin with five setae close to marginal vein; with 14 admarginal setae in one row.

Gaster with anterior ½ yellowish-white with dark brown lateral margins, posterior ½ dark brown (Fig. 317).

Ratios. HE/MS/WM = 2.1/1.0/1.1; POL/OOL/POO = 4.7/2.8/1.0; OOL/DO = 1.6; WE/WF/WH/HH = 1.0/2.7/4.8/3.7; WH/WT = 1.1; PM/ST = 1.7; TS1/TS2/LT/LT1/LT2/LT3/LT4 = 3.8/2.4/6.4/1.8/1.4/1.0/1.9; LP/WP = 1.2; MM/LG = 1.0.

*Male*. Unknown.

##### Hosts and biology.

Feeding on last instar larva of *Antiblemma* Poole03 (Erebidae) feeding on *Miconia impetiolaris* (Melastomataceae), parasitoid cocoons stuck to leaf substrate.

##### Distribution.

Costa Rica (Guanacaste Province).

##### Etymology.

This species is named after Ronald Zuñiga, in recognition of his contribution to the understanding of ACG Hymenoptera taxonomy.

#### Euplectrus roysnellingi

Taxon classificationAnimaliaHymenopteraEulophidae

Hansson sp. n.

http://zoobank.org/158E4E62-3657-4593-8401-2B91656666BE

Figures 332–335 , 339–341 , 782

##### Material.

Holotype a female labeled “COSTA RICA: Guanacaste, ACG, Sector Pitilla, Amonias, 20.ix.2007, P. Rios, ex *Letis mycerina* eating *Inga oerstediana*, sibling of wasp DHJPAR0028972, 07-SRNP-33368” (BMNH).

##### Diagnosis.

Lower face with median part yellowish-brown, pale part reaching slightly outside of level of outer lateral margin of toruli (Fig. 333); fore and mid coxae yellowish-white, remaining parts of fore and mid legs and entire hind leg yellowish-brown (Fig. 332); scutellum 0.8× as long as wide with slightly elongate meshes, very posteromedian part with transverse meshes (Fig. 341); dorsellum without a groove or foveae along anterior margin (Fig. 782); propodeum anteromedially with a transverse triangular cup, median carina on propodeum wide (Fig. 782); petiole 1.0× as long as wide; gaster dark brown, anterior ½ with a large yellowish-white spot (Fig. 334), and with tergites 5–6 reddish-brown posteromedially.

##### Description.

*Female*. Length of body 2.3 mm. Antenna with scape yellowish-white in basal ½ and yellowish-brown in apical ½, pedicel yellowish-brown, flagellomeres 1–4 dark brown with ventral part yellowish-brown, 5–6 dark brown (Fig. 335). Mandibles and palpi white. Head black and shiny, lower face with median part yellowish-brown, pale part reaching slightly outside of level of outer lateral margin of toruli, with parts between pale area and eyes black (Fig. 333). Frons close to eyes with one irregular row of setae (Fig. 339). Vertex with very weak reticulation (Fig. 340). Occipital margin with a weak carina behind ocellar triangle (Fig. 340).

Mesosoma black and shiny (Fig. 332). Each sidelobe of mesoscutum with 12 setae. Scutellum 0.8× as long as wide; with very weak reticulation, meshes slightly elongate (Fig. 341). Dorsellum without a groove or foveae along anterior margin (Fig. 782). Propodeum smooth (Fig. 782); anteromedially with a strongly transverse triangular cup that is 0.3× as long as wide; propodeal callus with nine setae. Legs (Fig. 332): fore and mid coxa yellowish-white, remaining parts of fore and mid legs and entire hind leg yellowish-brown. Fore wing: costal cell with two irregular and sparse rows of setae on ventral surface, and margin with three setae close to marginal vein; with 21 admarginal setae, partly in two rows.

Gaster dark brown, anterior ½ with a large yellowish-white spot (Fig. 334), and with tergites 5–6 reddish-brown posteromedially.

Ratios. HE/MS/WM = 2.6/1.0/1.4; POL/OOL/POO = 5.0/3.0/1.0; OOL/DO = 1.4; WE/WF/WH/HH = 1.0/2.0/3.7/2.8; WH/WT = 1.0; PM/ST = 1.9; TS1/TS2/LT/LT1/LT2/LT3/LT4 = 4.4/2.2/8.0/2.4/1.9/1.0/1.9; LP/WP = 1.0; MM/LG = 1.3.

*Male*. Unknown.

##### Hosts and biology.

Feeding on second instar larva of *Letis mycerina* (Erebidae) feeding on *Inga oerstediana* (Fabaceae), parasitoid cocoons stuck to dead larva and substrate.

##### Distribution.

Costa Rica (Guanacaste Province).

##### Etymology.

This species is named after Roy R. Snelling, in recognition of his contribution to the understanding of ACG Hymenoptera taxonomy.

##### Remarks.

The barcode from the single available specimen from this sample (DHJPAR0028972) is very similar to the barcode of DHJPAR0023280 (Suppl. material 1). Specimens from DHJPAR0023280 are included under the description of *Euplectrus daveroubiki*. Specimens from DHJPAR0028972 are morphologically distinct from those of DHJPAR0023280, and also have a very different host. Therefore we prefer to treat these two samples as different species. See also remarks under *Euplectrus daveroubiki*.

#### Euplectrus scottshawi

Taxon classificationAnimaliaHymenopteraEulophidae

Hansson sp. n.

http://zoobank.org/682B5A51-9C6C-4F4C-9E22-F9D6A96FBA1B

Figures 539–545 , 549–551 , 783

##### Material.

Holotype a female labeled “COSTA RICA: Guanacaste, ACG, Sector Pitilla, Sendero Cuestona, 22.v.2006, C. Moraga, ex *Trauaxa lua* eating *Stemmadenia robinsonii*, sibling of wasp DHJPAR0028877, 06-SRNP-31991” (BMNH). PARATYPES: 24♀ 4♂: COSTA RICA (ACG): **Alajuela:** Sector San Cristobal, Finca San Gabriel, 29.v.2006, C. Cano, ex *Trauaxa lua* eating *Tabernaemontana alba*, sibling of wasp DHJPAR0028857, 06-SRNP-4258 (2♀, in INBio); **Guanacaste:** Sector Pitilla: Sendero Mismo, 18.v.2006, C. Moraga, ex *Trauaxa lua* eating *Stemmadenia robinsonii*, sibling of wasp DHJPAR0028892, 06-SRNP-31937 (3♀ 1♂, in BMNH, INBio); Sendero Cuestona, 22.v.2006, C. Moraga, ex *Trauaxa lua* eating *Stemmadenia robinsonii*, sibling of wasp DHJPAR0028877, 06-SRNP-31991 (2♀, in CNC, USNM); Sendero Evangelista, 15.vii.2006, P. Rios, ex *Trauaxa lua* eating *Stemmadenia robinsonii*, sibling of wasp DHJPAR0028894, 06-SRNP-32841 (10♀, in BMNH, CNC, INBio, MZLU, USNM), with same locality and host as previous but collected 19.v.2012, sibling of wasp DHJPAR0050062, 12-SRNP-30860 (1♀ 1♂, in INBio); Sendero Naciente, 30.iv.2008, P. Rios, ex *Trauaxa lua* eating *Stemmadenia robinsonii*, sibling of wasp DHJPAR0031172, 08-SRNP-31151 (2♀ 1♂, in BMNH); Sendero Nacho, 2.xii.2013, ex *Trauaxa lua* eating *Stemmadenia robinsonii*, sibling of wasp DHJPAR0054868, 13-SRNP-31727, (4♀ 1♂, in BMNH); Sendero Orosilito, 15.iv.2013, F. Quesada, ex *Callionima denticulata* eating *Stemmadenia robinsonii*, sibling of wasp DHJPAR0052363, 13-SRNP-30579 (2♀ 1♂, in BMNH).

##### Diagnosis.

Lower face black (Figs 540, 541); fore and mid coxae white, remaining parts of fore and mid legs yellowish-brown, hind leg with coxa dark brown to almost black, femur with basal ½ yellowish-white and apical ½ dark brown, tibia yellowish-white, tarsus yellowish-brown (Fig. 539); petiole 1.0× as long as wide; gaster with anterior ½ yellowish-white with dark brown lateral margins, posterior ½ dark brown (Figs 542, 543); male scape expanded and widest above the middle (Fig. 545), sensory pores confined to apicoventral ⅔, sensory area pale brown.

##### Description.

*Female*. Length of body 2.4 mm. Antenna with scape yellowish-white, pedicel yellowish-brown, flagellomere 1 pale brown, 2–6 dark brown (Fig. 544). Mandibles yellowish-brown with base brown, palpi yellowish-white. Head including lower face black and shiny (Fig. 540). Frons close to eyes with one row of setae in lower ½, with two rows in upper ½ (Fig. 549). Vertex smooth (Fig. 550). Occipital margin with a weak carina (Fig. 550).

Mesosoma black and shiny (Fig. 539). Each sidelobe of mesoscutum with 10 setae. Scutellum 0.9× as long as wide; with weak engraved reticulation (Fig. 551). Dorsellum along anterior margin with a groove that is divided by longitudinal carinae (Fig. 783), groove medially 0.3× as long as length of dorsellum. Propodeum smooth (Fig. 783); anteromedially with a semicircular cup; propodeal callus with 11 setae. Legs (Fig. 539): fore and mid coxae white, remaining parts of fore and mid legs yellowish-brown; hind leg with coxa dark brown - almost black, femur with basal ½ yellowish-white and apical ½ dark brown, tibia yellowish-white, tarsus yellowish-brown. Fore wing: costal cell with two irregular rows of setae on ventral surface, and margin with three setae close to marginal vein; with 17 admarginal setae, in one row.

Gaster with anterior ½ yellowish-white with dark brown lateral margins, posterior ½ dark brown (Fig. 542).

Ratios. HE/MS/WM = 1.8/1.0/1.1; POL/OOL/POO = 6.5/3.5/1.0; OOL/DO = 1.4; WE/WF/WH/HH = 1.0/2.4/4.5/3.3; WH/WT = 1.0; PM/ST = 1.7; TS1/TS2/LT/LT1/LT2/LT3/LT4 = 4.4/2.6/6.4/2.4/1.2/1.0/2.1; LP/WP = 1.0; MM/LG = 1.4.

*Male*. Length of body 2.1 mm. Scape white, expanded and widest above the middle (Fig. 545), sensory pores confined to apicoventral ⅔, sensory area pale brown. Otherwise similar to female.

Ratio. LC/WS = 3.5.

##### Hosts and biology.

Feeding on last instar larva of *Trauaxa lua* (Erebidae) feeding on *Stemmadenia robinsonii* and *Tabernaemontana alba* (Apocynaceae), and on second instar larva of *Callionima denticulata* (Sphingidae) feeding on *Stemmadenia robinsonii*, parasitoid cocoons stuck to dead larva and substrate.

##### Distribution.

Costa Rica (Guanacaste Province).

##### Etymology.

This species is named after Scott R. Shaw, in recognition of his contribution to the understanding of ACG Hymenoptera taxonomy.

##### Remarks.

The specimens from *Callionima denticulata* are distinctly paler than specimens from *Trauaxa lua* but it is not known whether this is due to some aspect of their treatment, or if they were preserved in ethanol while still teneral. The unexpected host record of *Callionima denticulata*, a normal herbivore for this plant, was confirmed by further inspection and photography of the morphology of the mummy.

#### Euplectrus sondrawardae

Taxon classificationAnimaliaHymenopteraEulophidae

Hansson sp. n.

http://zoobank.org/3780B62D-9B2E-4162-81F2-AE83E58C4713

Figures 552–558 , 566–568 , 784

##### Material.

Holotype a female labeled “COSTA RICA: Guanacaste, ACG, Sector Pitilla, Sendero Mismo, 23.v.2006, C. Moraga, ex *Sanys irrosea* eating *Maytenis recondita*, sibling of wasp DHJPAR0028850, 06-SRNP-31984” (BMNH). PARATYPES: 1♀ 1♂ with same label data as holotype (BMNH, INBio).

##### Diagnosis.

**Figures 552–565.**
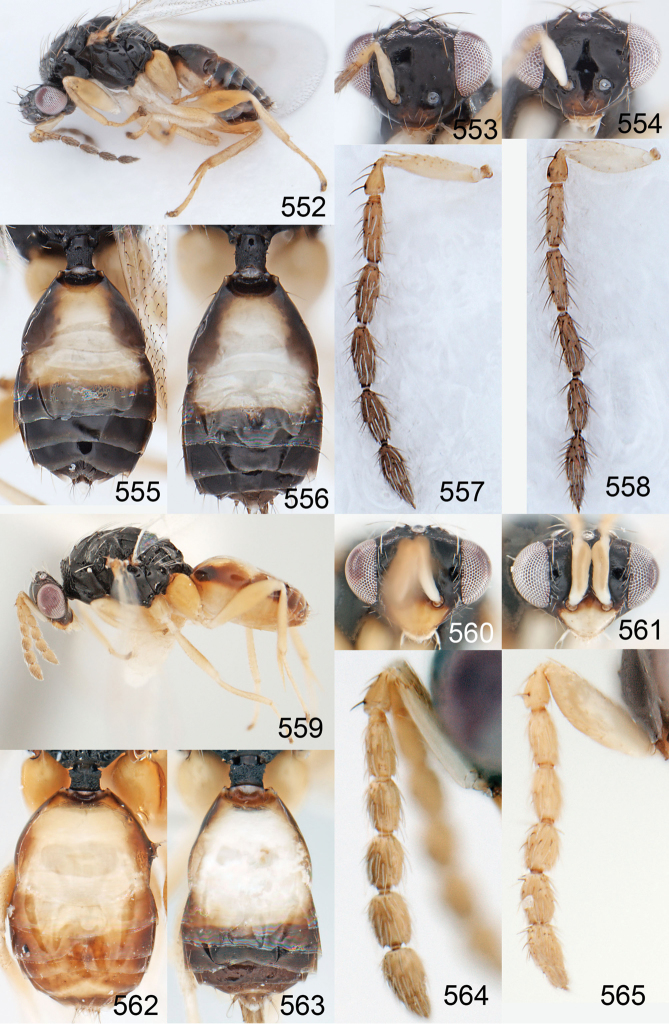
*Euplectrus* spp. **552–558** *Euplectrus sondrawardae* (DHJPAR0028850): **552** habitus in lateral view, female **553** head in frontal view, female **554** head in frontal view, male **555** gaster in dorsal view, female **556** gaster in dorsal view, male **557** antenna in lateral view, female **558** antenna in lateral view, male **559–565** *Euplectrus anae* (paratypes): **559** habitus in lateral view, female **560** head in frontal view, female **561** head in frontal view, male **562** gaster in dorsal view, female **563** gaster in dorsal view, male **564** antenna in lateral view, female **565** antenna in lateral view, male.

Lower face black with a dark reddish-brown spot medially, pale area reaching to level of middle of toruli (Figs 553, 554); antenna long and slender, flagellum 3.2× as long as height of eye in female (Fig. 557), and 3.8× in male (Fig. 558); legs yellowish-brown with fore and mid coxae yellowish-white (Fig. 552); hind tarsus short with 1^st^ tarsomere short, 1.1× as long as 3^rd^ tarsomere; petiole 0.8× as long as wide; gaster dark brown, anterior ½ with a ±T-shaped yellowish-white (female, Fig. 555) or white (male, Fig. 556) spot; male antenna with scape slightly expanded and widest just below the middle, 2.7× as long as wide (Fig. 558).

##### Description.

**Figures 566–571.**
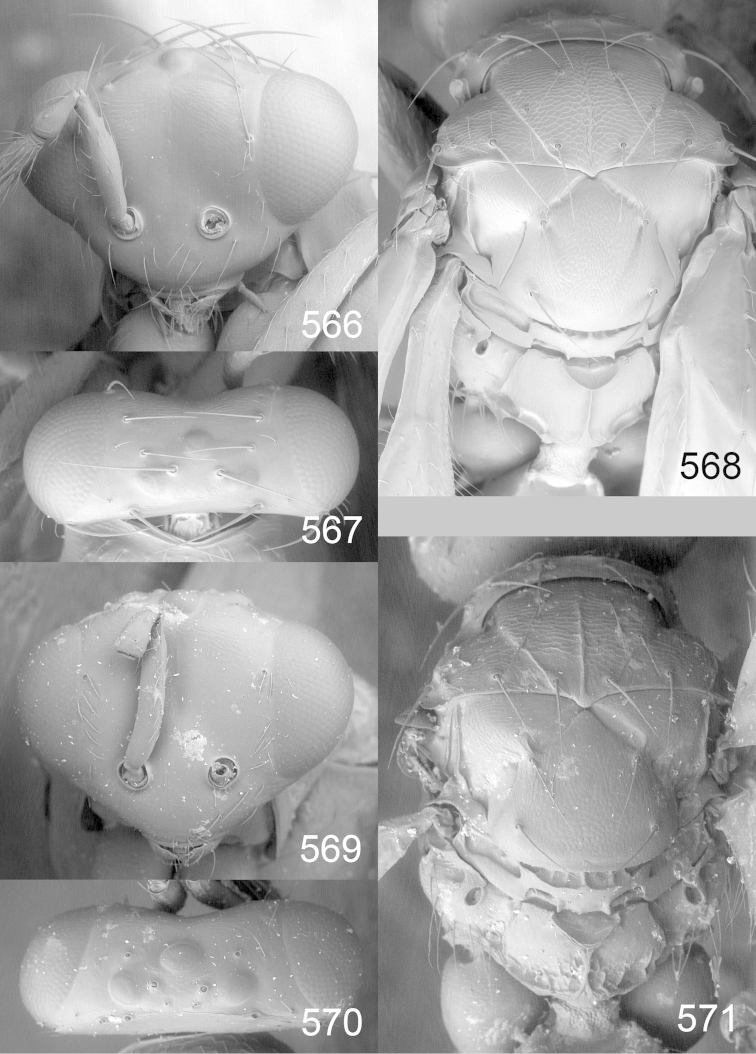
*Euplectrus* spp., females. **566–568** *Euplectrus sondrawardae* (DHJPAR0028850): **566** head in frontal view **567** vertex **568** mesosoma in dorsal view; **569–571** *Euplectrus anae* (92-SRNP-747, no barcode): **569** head in frontal view **570** vertex **571** mesosoma in dorsal view.

*Female*. Length of body 2.7 mm. Antenna long and slender, flagellum 3.2× as long as height of eye (Fig. 557); scape yellowish-white, pedicel yellowish-brown, flagellomere 1 yellowish-brown, 2 yellowish-brown ventrally and dark brown dorsally, 3–6 dark brown. Mandibles yellowish-brown, palpi yellowish-white. Head black and shiny, lower face black with a dark reddish-brown spot medially, pale area reaching to level of middle of toruli (Fig. 553). Frons close to eyes with one row of setae (Fig. 566). Vertex smooth (Fig. 567). Occipital margin rounded (Fig. 567).

Mesosoma black and shiny (Fig. 552). Each sidelobe of mesoscutum with seven setae. Scutellum 0.9× as long as wide; with weak engraved reticulation (Fig. 568). Dorsellum along anterior margin with a groove that is divided by longitudinal carinae (Fig. 784), groove medially 0.3× as long as length of dorsellum. Propodeum with very weak reticulation (Fig. 784); anteromedially with a semicircular cup; propodeal callus with nine setae. Legs yellowish-brown with fore and mid coxae yellowish-white (Fig. 552). Fore wing: costal cell with one row of setae on ventral surface, and margin with five setae close to marginal vein; with 17 admarginal setae, two rows in basal ½ and one row in apical ½.

Gaster dark brown, anterior ½ with a ±T-shaped yellowish-white spot (Fig. 555).

Ratios. HE/MS/WM = 2.5/1.0/1.6; POL/OOL/POO = 7.8/3.8/1.0; OOL/DO = 1.3; WE/WF/WH/HH = 1.0/2.2/4.1/3.0; WH/WT = 1.0; PM/ST = 2.1; TS1/TS2/LT/LT1/LT2/LT3/LT4 = 3.6/2.2/5.6/1.1/1.3/1.0/1.9; LP/WP = 0.8; MM/LG = 1.1.

*Male*. Length of body 2.0 mm. Scape slightly expanded and widest slightly below the middle (Fig. 558), sensory pores confined to ventral margin and scattered along entire margin, sensory area pale as scape. Otherwise similar to female except antennal flagellum 3.8× as long as height of eye (Fig. 558); fore and mid coxae white; gaster shorter and with pale part white (Fig. 556).

Ratios. LC/WS = 2.7; MM/LG = 1.3.

##### Hosts and biology.

Feeding on last instar larva of *Sanys irrosea* (Erebidae) feeding on *Maytenis recondita* (Celastraceae), parasitoid cocoons stuck to dead larva and substrate.

##### Distribution.

Costa Rica (Guanacaste Province).

##### Etymology.

This species is named after Sondra Ward, in recognition of her contribution to the understanding of ACG Hymenoptera taxonomy.

#### Euplectrus sydneycameronae

Taxon classificationAnimaliaHymenopteraEulophidae

Hansson sp. n.

http://zoobank.org/BA14F739-5047-4D17-9265-95963418C745

Figures 17 , 393–399 , 404–406 , 581 , 767

##### Material.

Holotype a female labeled “ COSTA RICA: Alajuela, ACG, Sector Pitilla, Pasmompa, 15.x.2007, P. Rios, ex *Plusiodonta clavifera* eating *Cissampelos tropaeolifolia*, sibling of wasp DHJPAR0028936, 07-SRNP-33543” (BMNH). PARATYPES: 5♀ 1♂ with same label data as holotype (BMNH, INBio, USNM).

##### Diagnosis.

Lower face entirely yellowish-brown (female, as in Fig. 394) or white (male, as in Fig. 395), pale part not reaching hypostomal carina and with area close to mouth cavity dark (as in Fig. 581); fore coxa yellowish-white, remaining fore leg and mid and hind legs yellowish-brown (as in Fig. 393); petiole 1.0× as long as wide; gaster dark brown, anterior ½ yellowish-white (female, as in Fig. 396) or white (male, as in Fig. 397) with dark brown lateral margins; male antenna with scape slightly expanded and widest in the middle, 2.6× as long as wide (as in Fig. 399), flagellomeres 2–5 with a basal whorl of erect setae.

##### Description.

*Female*. Length of body 2.3 mm. Antenna with scape yellowish-white, pedicel yellowish-brown, flagellomere 1 yellowish-brown, 2 pale brown, 3–6 dark brown (as in Fig. 398). Mandibles and palpi yellowish-white. Head black and shiny, entire lower face yellowish-brown with median part darker (as in Fig. 394). Frons close to eyes with two rows of setae (as in Fig. 404). Vertex smooth (as in Fig. 405). Occipital margin rounded (as in Fig. 405).

Mesosoma black and shiny (as in Fig. 393). Each sidelobe of mesoscutum with 20 setae. Scutellum 0.9× as long as wide; with very weak engraved reticulation (as in Fig. 406). Dorsellum along anterior margin with a very narrow groove (as in Fig. 767), medially 0.1× as long as length of dorsellum. Propodeum with very weak reticulation (as in Fig. 767); anteromedially with a semicircular cup that has posterior part strongly raised and is distinctly higher than anterior part; propodeal callus with eight setae. Legs (as in Fig. 393): fore coxa yellowish-white, remaining fore leg and mid and hind legs yellowish-brown. Fore wing: costal cell on ventral surface with two rows of setae in basal ⅔ and one row in apical ⅓, and margin with four setae close to marginal vein; with 16 admarginal setae, in one row.

Gaster dark brown, anterior ½ yellowish-white with dark brown lateral margins (as in Fig. 396).

Ratios. HE/MS/WM = 2.1/1.0/1.4; POL/OOL/POO = 4.9/2.9/1.0; OOL/DO = 1.4; WE/WF/WH/HH = 1.0/2.5/4.5/3.2; WH/WT = 1.1; PM/ST = 1.6; TS1/TS2/LT/LT1/LT2/LT3/LT4 = 4.0/2.4/6.4/2.0/1.7/1.0/1.7; LP/WP = 1.0; MM/LG = 1.0.

*Male*. Length of body 1.9 mm. Scape slightly expanded and widest in the middle (as in Fig. 399), sensory pores confined to apicoventral ¾, sensory area pale as scape. Otherwise similar to female except flagellomeres 2–5 with a basal whorl of erect setae (as in Fig. 399); entire lower face white (as in Fig. 395); scutellum with posterior ¼ smooth; gaster shorter and with pale part white.

Ratios. LC/WS = 2.9; MM/LG = 1.2.

##### Hosts and biology.

Feeding on penultimate instar of *Plusiodonta clavifera* (Erebidae) feeding on *Cissampelos tropaeolifolia* (Menispermaceae), parasitoid cocoons stuck to dead larva and substrate.

##### Distribution.

Costa Rica (Alajuela Province).

##### Etymology.

This species is named after Sydney A. Cameron, in recognition of her contribution to the understanding of ACG Hymenoptera taxonomy.

##### Remarks.

This species is morphologically identical to *Euplectrus johnnoyesi*, but the barcode is significantly different from this species, it is 5% divergent from *Euplectrus johnnoyesi* within the barcode region (24–32 bp (& 1 amino acid) different) (Fig. 35, Suppl. material 1), and also the host is different. In spite of the morphological similarity we therefore regard these as two different species.

#### Euplectrus victoriapookae

Taxon classificationAnimaliaHymenopteraEulophidae

Hansson sp. n.

http://zoobank.org/0FBD808F-DCCA-41F7-BE15-1E0E988B646C

Figures 572–578 , 590–592 , 785

##### Material.

Holotype a female labeled “COSTA RICA: Guanacaste, ACG, Sector Pitilla, Pasmompa, 5.viii.2005, P. Rios, ex *Cyclophora* Janzen14 eating *Siparuna thecophora*, sibling of wasp DHJPAR0028836, 05-SRNP-33114” (BMNH). PARATYPES: 8♀ 3♂: COSTA RICA (ACG): **Guanacaste:** 4♀ with same label data as holotype (CNC, INBio, USNM); **Alajuela:** Sector Rincon Rain Forest: Camino Rio Franca, M. Carmona, 30.iv.2007, ex *Cyclophora* Janzen14 eating *Siparuna thecophora*, sibling of wasp DHJPAR0028928, 07-SRNP-41115 (2♀ 1♂, in BMNH, INBio); 1♂ from same locality as previous but collected 14.vii.2011 on *Cyclophora* Janzen14 eating *Siparuna thecophora*, sibling of wasp DHJPAR0045453, 11-SRNP-80930 (INBio); Jacobo, 25.ix.2012, ex *Cyclophora* Janzen14 eating *Siparuna thecophora*, sibling of wasp DHJPAR0050058, 12-SRNP-81575 (2♀ 1♂, in BMNH, MZLU); Sendero Anonas, 2.ix.2013, J. Perez, ex *Cyclophora* Janzen14 eating *Siparuna thecophora*, sibling of wasp DHJPAR0053943, 13-SRNP-43435 (2♀, in CNC, USNM).

##### Diagnosis.

**Figures 572–582.**
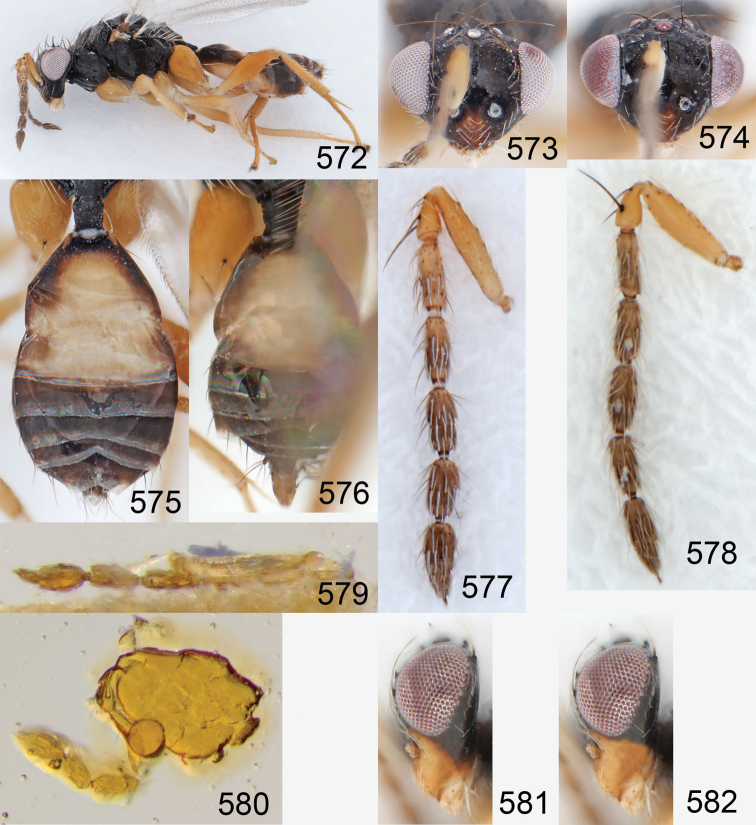
*Euplectrus* spp. **572–578** *Euplectrus victoriapookae* (DHJPAR0028836): **572** habitus in lateral view, female **573** head in frontal view, female **574** head in frontal view, male **575** gaster in dorsal view, female **576** gaster in dorsal view, male **577** antenna in lateral view, female **578** antenna in lateral view, male **579–580** *Euplectrus ronnai*, antennae **579** female paralectotype **580** male lectotype; **581–582** head in lateral view, females **581** *Euplectrus johnnoyesi* **582** *Euplectrus paulheberti*.

Lower face with median part dark reddish-brown, pale area reaching to level of middle of toruli (Figs 573, 574); fore wing with four setae on dorsal surface of submarginal vein and admarginal setae in three rows; legs yellowish-brown (Fig. 572); petiole 1.0× as long as wide; gaster with anterior ½ yellowish-white with dark brown lateral margins, posterior ½ dark brown (Figs 575, 576); male antenna with scape slightly expanded and widest above the middle, 3.3× as long as wide (Fig. 578).

##### Description.

*Female*. Length of body 2.6 mm. Antenna with scape and pedicel yellowish-brown, flagellomere 1 yellowish-brown with infuscations dorsally, 2 dark brown with base yellowish-brown, 3–6 dark brown (Fig. 577). Mandibles and palpi yellowish-white. Head black and shiny, lower face with median part dark reddish-brown, pale area reaching to level of middle of toruli, with parts between pale area and eyes black (Fig. 573). Frons close to eyes with one row of setae (Fig. 590). Vertex with very weak reticulation (Fig. 591). Occipital margin rounded (Fig. 591).

Mesosoma black and shiny (Fig. 572). Each sidelobe of mesoscutum with 13 setae. Scutellum 0.9× as long as wide; with very weak engraved reticulation, posterior margin smooth (Fig. 592). Dorsellum anteriorly with a groove that is divided by longitudinal carinae (Fig. 785), groove medially 0.4× as long as length of dorsellum. Propodeum smooth (Fig. 785); anteromedially with a triangular cup that has posterior part strongly raised; propodeal callus with ten setae. Legs yellowish-brown (Fig. 572). Fore wing: submarginal vein with four setae; costal cell on ventral surface with three rows of setae in basal ½, two rows in apical ½, and margin with eight setae close to marginal vein; with 36 admarginal setae, in three rows.

Gaster with anterior ½ yellowish-white with dark brown lateral margins, posterior ½ dark brown (Fig. 575).

Ratios. HE/MS/WM = 2.2/1.0/1.2; POL/OOL/POO = 6.3/3.3/1.0; OOL/DO = 1.1; WE/WF/WH/HH = 1.0/2.3/4.3/3.0; WH/WT = 1.1; PM/ST = 1.6; TS1/TS2/LT/LT1/LT2/LT3/LT4 = 3.7/2.0/5.5/1.9/1.5/1.0/1.5; LP/WP = 1.0; MM/LG = 0.9.

*Male*. Length of body 1.6 mm. Scape slightly expanded and widest above the middle (Fig. 578), sensory pores confined to apicoventral ¾, sensory area pale as scape. Otherwise similar to female except shorter gaster.

Ratios. LC/WS = 3.3; MM/LG = 1.3.

##### Hosts and biology.

Feeding on penultimate instar larva of *Cyclophora* Janzen14 (Geometridae) feeding on *Siparuna thecophora* (Siparunaceae), parasitoid cocoons stuck to dead larva and substrate.

##### Distribution.

Costa Rica (Alajuela and Guanacaste Provinces).

##### Etymology.

This species is named after Victoria G. Pook, in recognition of her contribution to the understanding of ACG Hymenoptera taxonomy.

##### Remarks.

*Euplectrus victoriapookae* and *Euplectrus davesmithi* have the same barcode (Fig. 35, Suppl. material 1), but as they are morphologically distinct and their hosts are very different we treat them as separate species.

#### Euplectrus wonyoungchoi

Taxon classificationAnimaliaHymenopteraEulophidae

Hansson sp. n.

http://zoobank.org/121E86DD-6759-4224-9819-E645AA665C79

Figures 28 , 349–352 , 356–358 , 786

##### Material.

Holotype a female labeled “COSTA RICA: Guanacaste, ACG, Sector Pitilla, Sendero Cuestona, 26.ix.2011, C. Moraga, ex *Elaphria* Poole08 eating unidentified Lejeuneaceae, sibling of wasp DHJPAR0048229, 11-SRNP-32964” (BMNH). Paratypes: 4♀ with same label data as holotype (CNC, INBio, USNM).

##### Diagnosis.

Lower face black (Fig. 350); mandibles dark brown; fore and mid legs with coxae yellowish-white, femora, tibiae and tarsi yellowish-brown; hind leg with coxa black, femur yellowish-brown with apical ⅓ pale brown, tibia and tarsus yellowish-brown (Fig. 349); petiole 0.9× as long as wide; gaster with anterior ½ yellowish-brown with wide dark brown lateral edges, posterior ½ dark brown (Fig. 351).

##### Description.

*Female*. Length of body 1.9 mm. Antenna with scape yellowish-white, pedicel yellowish-brown, flagellum dark brown (Fig. 352). Mandibles dark brown and palpi yellowish-white. Head including lower face black and shiny (Fig. 350). Frons close to eyes with two rows of setae (Fig. 356). Vertex smooth and shiny (Fig. 357). Occipital margin with a carina behind ocellar triangle (Fig. 357).

Mesosoma black and shiny (Fig. 349). Scutellum 1.0× as long as wide; with very weak reticulation, partly smooth (Fig. 358). Dorsellum along anterior margin with a deep groove (Fig. 786), groove medially 0.4× as long as length of dorsellum. Propodeum smooth and shiny (Fig. 786); anteromedially with short and wide semicircular cup that is strongly raised in posterior part; propodeal callus with ten setae. Legs (Fig. 349): fore and mid legs with coxae yellowish-white, femora, tibiae and tarsi yellowish-brown; hind leg with coxa black, femur yellowish-brown with apical ⅓ pale brown, tibia and tarsus yellowish-brown. Fore wing: costal cell with two rows of setae on ventral surface, and margin with five setae in apical ½; with 13 admarginal setae.

Gaster with anterior ½ yellowish-brown with wide dark brown lateral edges, posterior ½ dark brown (Fig. 351).

Ratios. HE/MS/WM = 2.1/1.0/1.3; POL/OOL/POO = 8.0/4.2/1.0; OOL/DO = 1.1; WE/WF/WH/HH = 1.0/2.5/4.6/3.4; WH/WT = 1.0; PM/ST = 1.8; TS1/TS2/LT/LT1/LT2/LT3/LT4 = 4.4/2.5/6.5/2.5/1.5/1.0/1.5; LP/WP = 0.9; MM/LG = 1.0.

*Male*. Unknown.

##### Hosts and biology.

Feeding on third instar larva of *Elaphria* Poole08 (Noctuidae) feeding on unidentified Lejeuneaceae, parasitoid cocoons stuck to dead larva and substrate.

##### Distribution.

Costa Rica (Guanacaste Province).

##### Etymology.

This species is named after Won-Young Choi, in recognition of his contribution to the understanding of ACG Hymenoptera taxonomy.

### Species treatments – described species from ACG

#### Euplectrus anae

Taxon classificationAnimaliaHymenopteraEulophidae

Schauff

Figures 559–565 , 569–571 , 787

Euplectrus anae Schauff in Schauff & Janzen, 2001: 193–194. Holotype ♀ (INBio), not examined.

##### Material.

Type material: 3♀ 2♂ paratypes of *Euplectrus anae* (USNM). Additional material: 4♀ from same rearing series (voucher 92-SRNP-747) as types (BMNH).

##### Diagnosis.

Lower face yellowish-brown (female, Fig. 560) or white (male, Fig. 561), pale area reaching slightly outside of outer lateral margin of toruli; legs yellowish-white with hind coxae pale yellowish-brown (Fig. 559); petiole 0.7× as long as wide; gaster with anterior ⅔ yellowish-white (female, Fig. 562) or white (male, Fig. 563) with dark brown lateral margins, and with posterior ⅓ pale brown (female) or dark brown (male).

##### Description.

*Female*. Length of body 2.4 mm. Antenna with scape yellowish-white with apex yellowish-brown, pedicel yellowish-brown, flagellum yellowish-brown ventrally and pale brown dorsally (Fig. 564). Mandibles and palpi yellowish-white. Head black and shiny with area lateral to ocellar triangle dark reddish-brown, lower face yellowish-brown, pale area reaches slightly outside of outer lateral margin of toruli (Fig. 560). Frons close to eyes with one row of setae, row partially double in upper ½ (Fig. 569). Vertex smooth with very weak reticulation inside ocellar triangle (Fig. 570). Occipital margin with a weak carina behind ocellar triangle (Fig. 570).

Mesosoma black and shiny (Fig. 559). Each sidelobe of mesoscutum with ten setae. Scutellum 1.0× as long as wide; with weak engraved reticulation, posterior margin smooth (Fig. 571). Dorsellum along anterior margin with a wide groove, medially 0.5× as long as length of dorsellum (Fig. 787). Propodeum smooth (Fig. 787); anteromedially with a triangular cup that has posterior part strongly raised; propodeal callus with six setae. Legs yellowish-white with hind coxae pale yellowish-brown (Fig. 559). Fore wing: submarginal vein with six setae; costal cell on ventral surface predominantly with two rows of setae, apically with a single row, and margin with three setae close to marginal vein; with 14 admarginal setae, in one row.

Gaster yellowish-white with dark brown lateral margins and with posterior ⅓ pale brown (Fig. 562).

Ratios. HE/MS/WM = 2.3/1.0/1.3; POL/OOL/POO = 9.0/4.0/1.0; OOL/DO = 1.0; WE/WF/WH/HH = 1.0/2.2/4.0/3.0; WH/WT = 1.0; PM/ST = 1.7; TS1/TS2/LT/LT1/LT2/LT3/LT4 = 4.4/2.6/7.0/2.6/1.6/1.0/1.6; LP/WP = 0.7; MM/LG = 0.9.

*Male*. Length of body 1.9 mm. Scape slightly expanded and widest apically, sensory pores confined to anteroventral ¾, sensory area pale brown (Fig. 565). Otherwise similar to female except pale area on lower face white (Fig. 561), gaster with anterior ⅔ white with dark brown lateral margins and posterior ⅓ dark brown (Fig. 563), and shorter.

Ratios. LC/WS = 2.8; MM/LG = 1.3.

##### Hosts and biology.

*Sphacelodes vulneraria* (Geometridae) feeding on an unknown plant (Schauff and Janzen 2001).

##### Distribution.

Costa Rica (Guanacaste Province) (Schauff and Janzen 2001).

#### Euplectrus carlowae

Taxon classificationAnimaliaHymenopteraEulophidae

Schauff

Figures 366–369 , 373–375 , 788

Euplectrus carlowae Schauff in Schauff & Janzen, 2001: 194–196. Holotype ♀ (INBio), not examined.

##### Material.

Type material: 1♀ paratype of *Euplectrus carlowae* (BMNH).

##### Diagnosis.

Lower face with median part very dark reddish-brown and not delimited from surrounding black areas (Fig. 367); mandibles dark brown; dorsellum anteriorly with a fovea, medially 0.4× as long as length of dorsellum, that is divided medially by a longitudinal carina (Fig. 788); legs yellowish-brown with fore and mid coxae yellowish-white (Fig. 366); petiole 1.0× as long as wide; gaster dark brown with a yellowish-white T-shaped spot in anterior ½ (Fig. 368).

##### Description.

*Female*. Length of body 2.2 mm. Antenna with scape yellowish-white with apex yellowish-brown, pedicel yellowish-brown, flagellomere 1 yellowish-brown ventrally and dark brown dorsally, flagellomeres 2–6 dark brown (Fig. 369). Mandibles dark brown, palpi yellowish-white. Head black and shiny, lower face with median part very dark reddish-brown and not delimited from surrounding black areas (Fig. 367). Frons close to eyes with one row of setae (Fig. 373). Vertex smooth (Fig. 374). Occipital margin with a weak carina (Fig. 374).

Mesosoma dark brown and shiny (Fig. 366). Each sidelobe of mesoscutum with eight setae. Scutellum 1.0× as long as wide; with weak engraved reticulation, posterior margin smooth (Fig. 375). Dorsellum anteriorly with a fovea, medially 0.4× as long as length of dorsellum, that is divided medially by a longitudinal carina (Fig. 788). Propodeum smooth (Fig. 788); anteromedially with a triangular cup that has posterior part strongly raised; propodeal callus with eight setae. Legs yellowish-brown with fore and mid coxae yellowish-white (Fig. 366). Fore wing: costal cell one row of setae on ventral surface, and margin with four setae close to marginal vein; with ten admarginal setae, in one row.

Gaster dark brown with a yellowish-white T-shaped spot in anterior ½ (Fig. 368).

Ratios. HE/MS/WM = 1.8/1.0/1.0; POL/OOL/POO = 4.0/2.8/1.0; OOL/DO = 1.7; WE/WF/WH/HH = 1.0/2.5/4.2/3.2; WH/WT = 1.1; PM/ST = 2.0; TS1/TS2/LT/LT1/LT2/LT3/LT4 = 4.2/2.7/6.5/1.7/1.5/1.0/2.1; LP/WP = 1.0; MM/LG = 0.9.

*Male*. Unknown.

##### Hosts and biology.

Unknown.

##### Distribution.

Costa Rica (Guanacaste Province) (Schauff and Janzen 2001).

#### Euplectrus floryae

Taxon classificationAnimaliaHymenopteraEulophidae

Schauff

Figures 21 , 583–589 , 593–595 , 789

Euplectrus floryae Schauff in Schauff & Janzen, 2001: 200–203. Holotype ♀ (INBio), not examined.

##### Material.

Type material: 1♀ 1♂ paratypes of *Euplectrus floryae* (BMNH). Additional material: COSTA RICA: 104♀ 15♂, caterpillar project vouchers: 82-SRNP-368, 84-SRNP-1501, 90-SRNP-1946, 91-SRNP-248, 91-SRNP-248, 91-SRNP-251, 91-SRNP-277, 91-SRNP-278, 91-SRNP-282, 91-SRNP-1512, 91-SRNP-1713, 92-SRNP-293, 92-SRNP-524, 92-SRNP-1073, 92-SRNP-1084, 92-SRNP-1115, 92-SRNP-2612, 92-SRNP-3473, 92-SRNP-5353, 93-SRNP-2502, 93-SRNP-7708, 94-SRNP-935, 97-SRNP-3317, 97-SRNP-4188, 97-SRNP-4197, 97-SRNP-4198, 97-SRNP-4210, 97-SRNP-4238, 97-SRNP-4240, 97-SRNP-4242, 97-SRNP-4248, 97-SRNP-4250, 97-SRNP-4251, 97-SRNP-4270, 97-SRNP-4271, 97-SRNP-4275, 97-SRNP-4310, 97-SRNP-4323, 97-SRNP-4324, 97-SRNP-5955, 97-SRNP-9370, 05-SRNP-55990, 05-SRNP-55991, 11-SRNP-20910, 11-SRNP-20928, 11-SRNP-20935, 13-SRNP-20741 (BMNH, CNC, INBio, MZLU, MIUCR, USNM).

##### Diagnosis.

**Figures 583–589.**
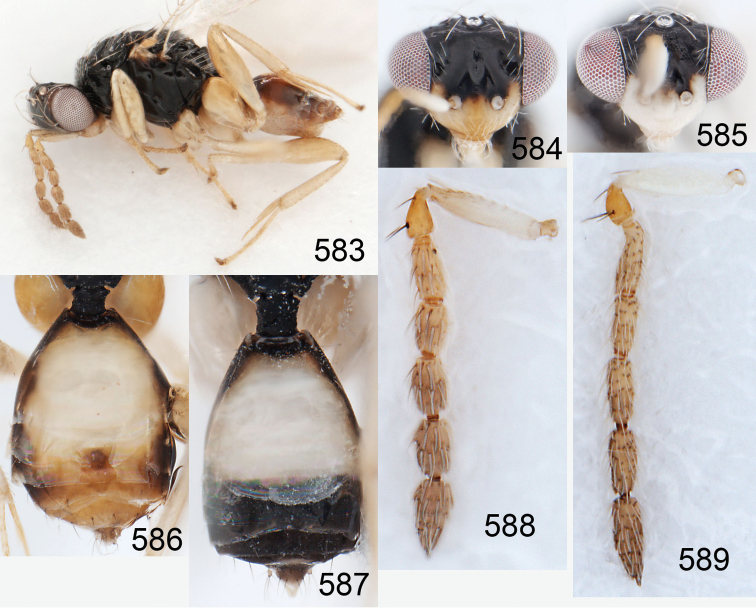
*Euplectrus floryae* (non-types): **583** habitus in lateral view, female **584** head in frontal view, female **585** head in frontal view, male **586** gaster in dorsal view, female **587** gaster in dorsal view, male **588** antenna in lateral view, female **589** antenna in lateral view, male.

**Figures 590–595.**
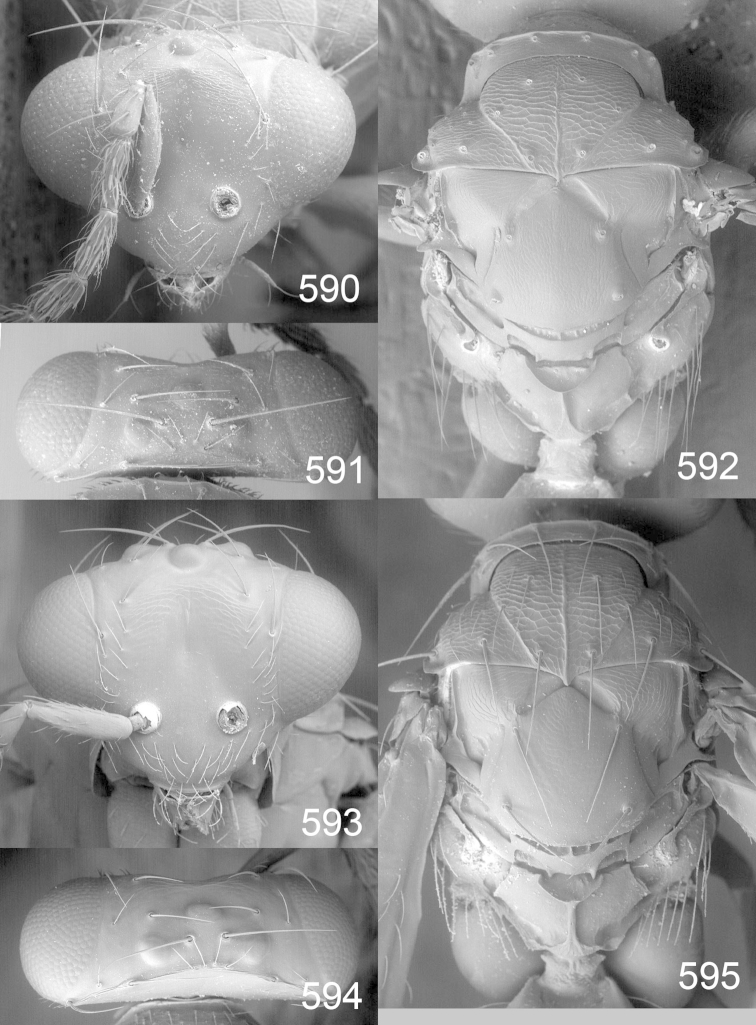
*Euplectrus* spp., females. **590–592** *Euplectrus victoriapookae* (DHJPAR0028836): **590** head in frontal view **591** vertex **592** mesosoma in dorsal view; **593–595** *Euplectrus floryae* (non-types): **593** head in frontal view **594** vertex **595** mesosoma in dorsal view.

Entire lower face yellowish-white with median part yellowish-brown (female, Fig. 584) or completely white (male, Fig. 585), pale part close to eyes extends above level of toruli and laterally to hypostomal carina; vertex dark reddish-brown; legs in female with fore and mid legs white to yellowish-white with tarsi yellowish-brown, hind leg with coxa and tarsus yellowish-brown, femur and tibia yellowish-white (Fig. 583), in male fore and mid legs as in female, but hind leg with coxa and basal ½ of femur white, apical ½ of femur, tibia and tarsus yellowish-white; petiole 1.0× as long as wide in both sexes; female gaster yellowish-white with dark brown lateral margins, with posterior ⅓ pale brown with dark brown lateral margins and with a small round dark brown spot anteromedially (Fig. 586), male gaster with basal ½ white with black lateral margins, posterior ½ black (Fig. 587); male antenna with scape slightly expanded and widest in the middle, 2.8× as long as wide (Fig. 589).

##### Description.

*Female*. Length of body 2.0 mm. Antenna with scape yellowish-white with apex yellowish-brown, pedicel and flagellomere 1 yellowish-brown, 2–3 pale brown, 4–6 dark brown (Fig. 588). Mandibles and palpi white. Head black and shiny, lower face yellowish-white with median part yellowish-brown (Fig. 584), pale part close to eyes extends above level of toruli and laterally to hypostomal carina; vertex dark reddish-brown. Frons close to eyes with two rows of setae (Fig. 593). Vertex smooth (Fig. 594). Occipital margin with a carina behind ocellar triangle (Fig. 594).

Mesosoma black and shiny (Fig. 583). Each sidelobe of mesoscutum with 14 setae. Scutellum 1.0× as long as wide; with very weak engraved reticulation (Fig. 595). Dorsellum anteriorly with a groove that is divided by longitudinal carinae (Fig. 789), groove medially 0.3× as long as length of dorsellum. Propodeum with very weak reticulation (Fig. 789); anteromedially with a semicircular cup; propodeal callus with 12 setae. Legs (Fig. 583): fore and mid legs white to yellowish-white with tarsi yellowish-brown, hind leg with coxa and tarsus yellowish-brown, femur and tibia yellowish-white. Fore wing: costal cell with two rows of setae on ventral surface, and margin with two setae close to marginal vein; with 13 admarginal setae, in one row.

Petiole anterolaterally with a strong seta. Gaster yellowish-white with dark brown lateral margins, posterior ⅓ pale brown with dark brown lateral margins and with a small round dark brown spot anteromedially (Fig. 586).

Ratios. HE/MS/WM = 2.3/1.0/1.3; POL/OOL/POO = 7.4/3.6/1.0; OOL/DO = 0.9; WE/WF/WH/HH = 1.0/2.3/4.2/3.0; WH/WT = 1.2; PM/ST = 1.4; TS1/TS2/LT/LT1/LT2/LT3/LT4 = 4.4/2.9/7.1/2.4/1.9/1.0/1.6; LP/WP = 1.0; MM/LG = 1.2.

*Male*. Length of body 1.9 mm. Scape white, slightly expanded and widest in the middle (Fig. 589), sensory pores confined to apicoventral ⅔. Otherwise similar to female except lower face completely white (Fig. 585); hind coxa and basal ½ of femur white, apical ½ of femur, tibia and tarsus yellowish-white; gaster with basal ½ white with black lateral margins, posterior ½ black (Fig. 587).

Ratios. LC/WS = 2,8; MM/LG = 1.2.

##### Hosts and biology.

*Cautethia spuria*, *Enyo ocypete*, *Perigonia ilusDHJ01*, *Perigonia lusca* (all hosts are Sphingidae).

##### Distribution.

Costa Rica (Guanacaste Province) (Schauff and Janzen 2001).

#### Euplectrus hansoni

Taxon classificationAnimaliaHymenopteraEulophidae

Schauff

Figures 383–386 , 390–392 , 790

Euplectrus hansoni Schauff in Schauff & Janzen, 2001: 203–204. Holotype ♀ (USNM), examined.

##### Material.

Type material: ♀ holotype (USNM). Additional material: COSTA RICA: 1♀ from Guanacaste, ACG, Sector Pitilla, Sendero Mismo, 30.viii.2006, ex Isochromodes sheilaDHJ05 on *Calatola costaricensis*, caterpillar project voucher code 06-SRNP-34036, no barcode (BMNH).

##### Diagnosis.

Lower face medially dark reddish-brown, pale area reaching to level of middle of toruli (Fig. 384); gaster with anterior ½ yellowish-brown with dark brown lateral margins, posterior ½ dark brown (Fig. 385); legs yellowish-brown (Fig. 383); occipital margin rounded (Fig. 391); petiole 1.2× as long as wide.

##### Description.

*Female*. Length of body 2.6 mm. Antenna with scape, pedicel and flagellomere 1 yellowish-brown, 2 pale brown, 3–6 dark brown (Fig. 386). Mandibles yellowish-brown, palpi yellowish-white. Head black and shiny, lower face medially dark reddish-brown, pale area reaching to level of middle of toruli, with part between pale area and eyes black (Fig. 384). Frons close to eyes with two rows of setae (Fig. 390). Vertex smooth (Fig. 391). Occipital margin rounded (Fig. 391).

Mesosoma black and shiny (Fig. 383). Each sidelobe of mesoscutum with 13 setae. Scutellum 0.9× as long as wide; with very weak engraved reticulation (Fig. 392). Dorsellum with a very narrow groove along anterior margin (Fig. 790), medially less than 0.1× as long as length of dorsellum. Propodeum smooth (Fig. 790); anteromedially with a semicircular cup; propodeal callus with eight setae. Legs yellowish-brown (Fig. 383). Fore wing: costal cell with two complete rows of setae on ventral surface, and margin with nine setae in apical ½; with 35 admarginal setae, in two rows in basal ⅔, with three rows in apical ⅓.

Gaster with anterior ½ yellowish-brown with dark brown lateral margins, posterior ½ dark brown (Fig. 385).

Ratios. HE/MS/WM = 2.3/1.0/1.2; POL/OOL/POO = 8.4/5.0/1.0; OOL/DO = 0.9; WE/WF/WH/HH = 1.0/2.3/4.3/3.2; WH/WT = 1.1; PM/ST = 1.9; TS1/TS2/LT/LT1/LT2/LT3/LT4 = 4.7/2.9/7.8/2.7/1.6/1.0/2.0; LP/WP = 1.2; MM/LG = 0.9.

*Male*. Unknown.

##### Hosts and biology.

*Isochromodes* sheilaDHJ05 (Geometridae) feeding on *Calatola costaricensis* (Icacinaceae) (**new record**).

##### Distribution.

Costa Rica (Guanacaste Province) (Schauff and Janzen 2001).

#### Euplectrus ireneae

Taxon classificationAnimaliaHymenopteraEulophidae

Schauff

Figures 400–403 , 407–409 , 791

Euplectrus ireneae Schauff in Schauff & Janzen, 2001: 204–207. Holotype ♀ (INBio), not examined.

##### Material.

Type material: 1♀ paratype of *Euplectrus ireneae* (BMNH).

##### Diagnosis.

Lower face medially yellowish-brown, pale area reaching slightly outside of outer lateral margins of toruli (Fig. 401); vertex with parts close to eyes dark reddish-brown; eyes and ocelli large (Fig. 408), WE/WF = 0.9, OOL/DO = 0.5; dorsellum anteriorly without a groove or foveae (Fig. 791); petiole 0.9× as long as wide; legs white with hind coxa and hind femur yellowish-white (Fig. 400); gaster with anterior ½ white with dark brown anterolateral margins, posterior ½ dark brown with apex reddish-brown (Fig. 400).

##### Description.

*Female*. Length of body 2.6 mm. Antenna with scape white, pedicel and flagellomeres yellowish-white (Fig. 403). Mandibles and palpi white. Head black and shiny, lower face medially yellowish-brown, pale area reaching slightly outside of outer lateral margins of toruli, parts between pale area and eyes black (Fig. 401). Frons close to eyes with one row of setae (Fig. 407). Vertex with parts close to eyes dark reddish-brown; smooth (Fig. 408). Occipital margin rounded (Fig. 408).

Mesosoma black and shiny (Fig. 400). Each sidelobe of mesoscutum with nine setae. Scutellum 1.0× as long as wide; with very weak engraved reticulation, partly smooth (Fig. 409). Dorsellum anteriorly without a groove or foveae (Fig. 791). Propodeum with very weak reticulation (Fig. 791); anteromedially with a transverse semicircular cup; propodeal callus with six setae. Legs white with hind coxa and hind femur yellowish-white (Fig. 400). Fore wing: costal cell with one row of setae on ventral surface, and margin with three setae close to marginal vein; with 15 admarginal setae, in one row.

Gaster with anterior ½ white with dark brown anterolateral margins, posterior ½ dark brown with apex reddish-brown (Fig. 402).

Ratios. HE/MS/WM = 2.8/1.0/1.3; POL/OOL/POO = 4.7/1.3/1.0; OOL/DO = 0.5; WE/WF/WH/HH = 1.0/1.1/2.9/2.0; WH/WT = 1.1; PM/ST = 1.4; TS1/TS2/LT/LT1/LT2/LT3/LT4 = 3.3/2.2/5.8/1.7/1.3/1.0/1.8; LP/WP = 0.9; MM/LG = 1.0.

*Male*. Unknown.

##### Hosts and biology.

*Motya* Poole02 (Nolidae) feeding on *Conocarpus erectus* (Combretaceae) (Schauff and Janzen 2001).

##### Distribution.

Costa Rica (Guanacaste Province) (Schauff and Janzen 2001).

#### Euplectrus ivonae

Taxon classificationAnimaliaHymenopteraEulophidae

Schauff

Figures 426 , 596–602 , 610–612 , 792

Euplectrus ivonae Schauff in Schauff & Janzen, 2001: 207–209. Holotype ♀ (INBio), not examined.

##### Material.

Type material: 6♀ 1♂ paratypes of *Euplectrus ivonae* (USNM). Additional material: COSTA RICA: 68♀ 5♂ feeding on *Cropia cedica*, *Cropia connecta*, *Cropia hadenoides* (BMNH, CNC, INBio, MZLU, MIUCR, USNM); caterpillar project voucher codes 90-SRNP-2196, 05-SRNP-46246, 06-SRNP-56342, 06-SRNP-56345, 12-SRNP-78168 (DHJPAR0051504).

##### Diagnosis.

**Figures 596–602.**
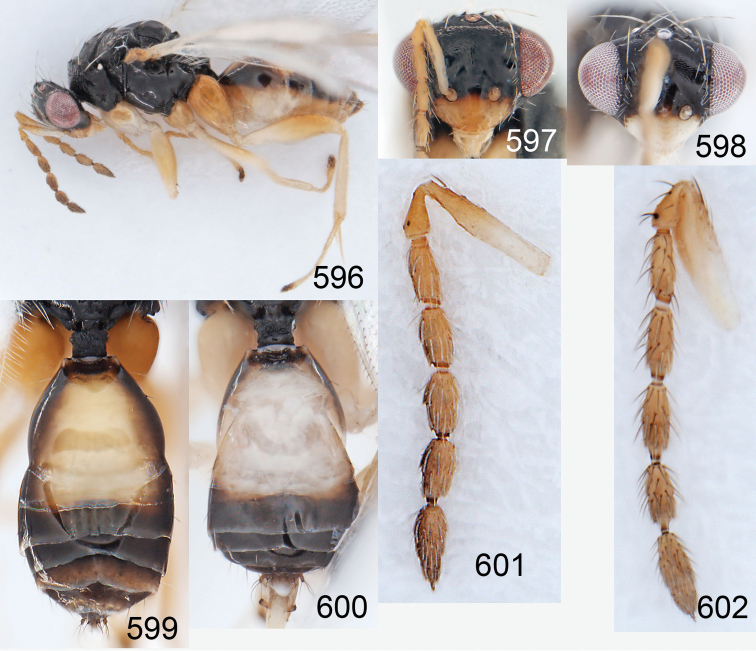
*Euplectrus ivonae* (non-types): **596** habitus in lateral view, female **597** head in frontal view, female **598** head in frontal view, male **599** gaster in dorsal view, female **600** gaster in dorsal view, male **601** antenna in lateral view, female **602** antenna in lateral view, male.

Lower face yellowish-brown (female, Fig. 597) or white (male, Fig. 598), with a black stripe the width of smallest diameter of toruli close to eyes; scutellum with a small hump posteromedially (Fig. 612); dorsellum anteriorly with a very wide groove, medially 0.7× as long as length of dorsellum (Fig. 792); legs yellowish-white with hind coxa and hind femur yellowish-brown (Fig. 596); petiole 0.8× as long as wide; female gaster with anterior ½ yellowish-brown with dark brown lateral margins, posterior ½ dark brown with apex reddish-brown (Fig. 599), male gaster with anterior ½ white with dark brown anterolateral margins and posterior ½ dark brown (Fig. 600); male antenna with scape slightly expanded and widest above the middle and with base narrow, 3.4× as long as wide (Fig. 602).

##### Description.

*Female*. Length of body 2.6 mm. Antenna with scape yellowish-white in basal ½, yellowish-brown in apical ½, pedicel and flagellomere 1 yellowish-brown, 2–6 dark brown dorsally and yellowish-brown ventrally (Fig. 601). Mandibles and palpi yellowish-white. Head black and shiny, lower face yellowish-brown with a black stripe the width of smallest diameter of toruli close to eyes (Fig. 597). Frons close to eyes with one row of setae that deviates away from eyes in upper part (Fig. 613). Vertex smooth (Fig. 614). Occipital margin with a carina behind ocellar triangle (Fig. 614).

Mesosoma black and shiny (Fig. 596). Each sidelobe of mesoscutum with 12 setae. Scutellum 1.0× as long as wide; with weak engraved reticulation (Fig. 612). Dorsellum anteriorly with a very wide groove that is divided by longitudinal carinae (Fig. 792), groove medially 0.7× as long as length of dorsellum. Propodeum with very weak reticulation (Fig. 792); anteromedially with a transverse semicircular cup; propodeal callus with ten setae. Legs yellowish-white with hind coxa and hind femur yellowish-brown (Fig. 596). Fore wing: costal cell on ventral surface with one row of setae, and margin with three setae close to marginal vein; with 13 admarginal setae, in one row.

Gaster with anterior ½ yellowish-brown with dark brown lateral margins, posterior ½ dark brown with apex reddish-brown (Fig. 599).

Ratios. HE/MS/WM = 2.6/1.0/1.4; POL/OOL/POO = 5.6/2.6/1.0; OOL/DO = 1.1; WE/WF/WH/HH = 1.0/2.4/4.2/3.3; WH/WT = 1.0; PM/ST = 1.5; TS1/TS2/LT/LT1/LT2/LT3/LT4 = 4.6/2.8/8.0/2.7/1.7/1.0/2.0; LP/WP = 0.8; MM/LG = 0.9.

*Male*. Length of body 2.2 mm. Scape slightly expanded and widest above the middle and with base narrow (Fig. 602), sensory pores confined to apicoventral ⅔ (Fig. 426). Otherwise similar to female except lower face white (Fig. 598); gaster with anterior ½ white with dark brown anterolateral margins and posterior ½ dark brown (Fig. 600).

Ratios. LC/WS = 3.4; MM/LG = 0.9.

##### Hosts and biology.

*Cropia cedica* feeding on *Cordia alliodora*, *Cropia connecta* feeding on *Cordia alliodora* and *Varronia inermis* (host plants are Boraginaceae), *Cropia hadenoides* feeding on *Hyptis obtusifolia* (Lamiaceae) (**all new records**), *Euscirrhopterus poeyi* feeding on *Pisonia aculeata* (Nyctaginaceae) (Schauff and Janzen 2001) (all hosts are Noctuidae).

##### Distribution.

Costa Rica (Guanacaste Province) (Schauff and Janzen 2001).

#### Euplectrus josei

Taxon classificationAnimaliaHymenopteraEulophidae

Schauff

Figures 603–609 , 613–615 , 793

Euplectrus josei Schauff in Schauff & Janzen, 2001: 209–210. Holotype ♀ (INBio), not examined.

##### Material.

Type material: 1♀ 1♂ paratypes of *Euplectrus josei* (BMNH). Additional material: COSTA RICA: 29♀ 2♂ from Guanacaste Province on *Paectes lunodes* and *Paectes tumida*; caterpillar project voucher codes: 93-SRNP-2869, 93-SRNP-2871, 93-SRNP-3064, 93-SRNP-3093, 95-SRNP-6055 (BMNH, CNC, INBio, MZLU, MIUCR, USNM).

##### Diagnosis.

**Figures 603–609.**
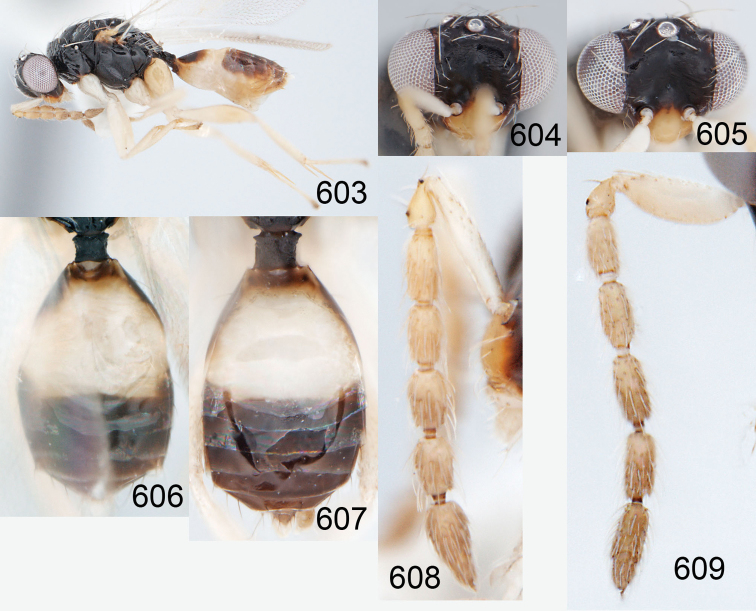
*Euplectrus josei* (paratypes): **603** habitus in lateral view, female **604** head in frontal view, female **605** head in frontal view, male **606** gaster in dorsal view, female **607** gaster in dorsal view, male **608** antenna in lateral view, female **609** antenna in lateral view, male.

Lower face medially yellowish-brown in both sexes, reaching to level of outer lateral margin of toruli (Figs 604, 605); vertex with parts close to eyes dark reddish-brown; eyes and ocelli large (Figs 613, 614), WE/WF = 0.7, OOL/DO = 0.6; petiole 0.7× as long as wide in female, 1.0× in male; legs white with hind coxa yellowish-white (Fig. 603); female gaster with anterior ½ yellowish-white with dark brown anterolateral margins, posterior ½ with anterior ½ dark brown and posterior ½ reddish-brown (Fig. 606), male gaster with anterior ½ white with dark brown anterolateral margins and posterior ½ dark brown (Fig. 607); male antenna with scape slightly expanded and widest in the middle, 3.0× as long as wide (Fig. 609).

##### Description.

*Female*. Length of body 2.4 mm. Antenna with scape white, pedicel and flagellomeres yellowish-brown (Fig. 608). Mandibles and palpi white. Head black and shiny, lower face medially yellowish-brown, reaching to level of outer lateral margin of toruli, parts between pale area and eyes black (Fig. 604). Frons close to eyes with one row of setae (Fig. 613). Vertex with parts close to eyes dark reddish-brown; with very weak reticulation (Fig. 614). Occipital margin with a carina (Fig. 614).

Mesosoma black and shiny (Fig. 603). Each sidelobe of mesoscutum with 13 setae. Scutellum 1.0× as long as wide; with weak engraved reticulation (Fig. 615). Dorsellum anteriorly with a narrow groove that is divided by longitudinal carinae (Fig. 793), groove medially 0.2× as long as length of dorsellum. Propodeum smooth (Fig. 793); anteromedially with a transverse triangular cup; propodeal callus with nine setae. Legs white with hind coxa yellowish-white (Fig. 603). Fore wing: costal cell on ventral surface with two rows of setae, and margin with three setae close to marginal vein; with 19 admarginal setae, in one row.

Gaster with anterior ½ yellowish-white with dark brown anterolateral margins, posterior ½ with anterior ½ dark brown and posterior ½ reddish-brown (Fig. 606).

Ratios. HE/MS/WM = 2.5/1.0/1.3; POL/OOL/POO = 8.0/3.0/1.0; OOL/DO = 0.6; WE/WF/WH/HH = 1.0/1.5/3.3/2.4; WH/WT = 1.1; PM/ST = 1.8; TS1/TS2/LT/LT1/LT2/LT3/LT4 = 4.2/2.6/6.8/2.2/1.8/1.0/1.8; LP/WP = 0.7; MM/LG = 1.0.

*Male*. Length of body 2.2 mm. Scape slightly expanded and widest in the middle (Fig. 609), with sensory pores along entire ventral margin. Otherwise similar to female except petiole longer; gaster shorter with anterior ½ white with dark brown anterolateral margins and posterior ½ dark brown (Fig. 607).

Ratios. LC/WS = 3.0; LP/WP = 1.0; MM/LG = 1.2.

##### Hosts and biology.

*Paectes lunodes* feeding on *Ocotea veraguensis* (Lauraceae) (Schauff and Janzen 2001), *Paectes tumida* feeding on *Bursera simaruba* (Burseraceae) (**new record**) (hosts are Euteliidae).

##### Distribution.

Costa Rica (Guanacaste Province).

#### Euplectrus magdae

Taxon classificationAnimaliaHymenopteraEulophidae

Schauff

Figures 18 , 616–622 , 630–632 , 794

Euplectrus magdae Schauff in Schauff & Janzen, 2001: 210–214. Holotype ♀ (INBio), not examined.

##### Material.

Type material: 1♀ paratype of *Euplectrus magdae* (BMNH). Additional material: COSTA RICA: 42♀ 11♂ from Guanacaste Province; caterpillar project voucher codes: 94-SRNP-6167, 96-SRNP-11096, 99-SRNP-11068, 00-SRNP-19037, 01-SRNP-9851, 01-SRNP-16124, 05-SRNP-46987, 07-SRNP-46045, 11-SRNP-56354 (BMNH, CNC, INBio, MZLU, MIUCR, USNM).

##### Diagnosis.

**Figures 616–622.**
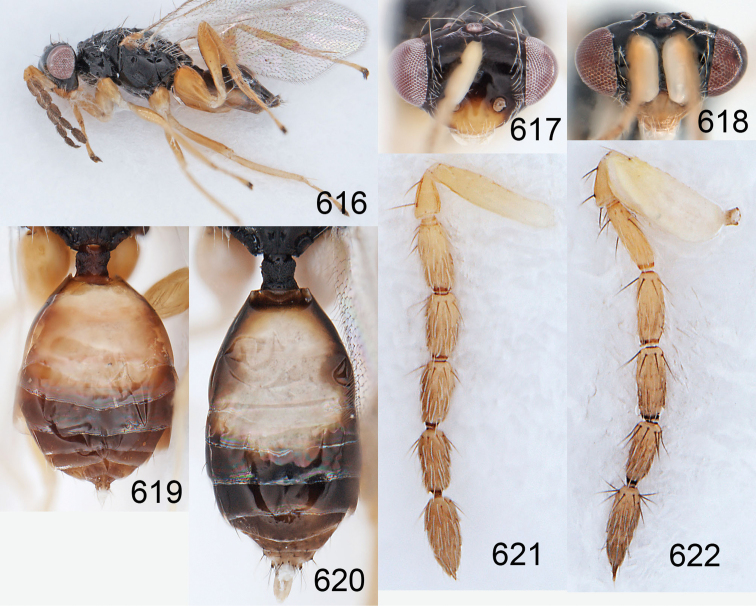
*Euplectrus magdae* (non-types): **616** habitus in lateral view, female **617** head in frontal view, female **618** head in frontal view, male **619** gaster in dorsal view, female **620** gaster in dorsal view, male **621** antenna in lateral view, female **622** antenna in lateral view, male.

Lower face medially yellowish-brown, pale area reaching to level of outer lateral margins of toruli (Fig. 617); vertex reddish-brown; dorsellum anteriorly with two large foveae (Fig. 794); fore and mid legs yellowish-white, hind leg with coxa yellowish-brown, femur with basal ½ yellowish-white and apical ½ yellowish-brown, tibia and tarsus yellowish-white (Fig. 616); petiole 0.9× as long as wide in female, 1.2× in male; gaster with anterior ½ yellowish-white (female, Fig. 619), or dusky white (male, Fig. 620), with dark brown lateral margins, posterior ½ dark brown.

##### Description.

*Female*. Length of body 3.0 mm. Antenna with scape, pedicel and flagellomeres 1–2 yellowish-white, 3–4 pale brown, 5–6 dark brown (Fig. 621). Mandibles and palpi white. Head black and shiny, lower face medially yellowish-brown, pale area reaching to level of outer lateral margins of toruli, with part between pale area and eyes black (Fig. 617), and vertex reddish-brown. Frons close to eyes with three rows of setae (Fig. 630). Vertex smooth (Fig. 631). Occipital margin with a carina behind ocellar triangle (Fig. 631).

Mesosoma black and shiny (Fig. 616). Each sidelobe of mesoscutum with 14 setae. Scutellum 0.9× as long as wide; with weak engraved reticulation (Fig. 632). Dorsellum anteriorly with two large foveae (Fig. 794), surface behind foveae reticulate. Propodeum with very weak reticulation (Fig. 794); anteromedially with a transverse triangular cup; propodeal callus with eight setae. Legs (Fig. 616): fore and mid legs yellowish-white, hind leg with coxa yellowish-brown, femur with basal ½ yellowish-white and apical ½ yellowish-brown, tibia and tarsus yellowish-white. Fore wing with submarginal vein with five setae; costal cell on ventral surface with two rows of setae, and margin with five setae close to marginal vein; with 24 admarginal setae, in two rows.

Gaster with anterior ½ yellowish-white with dark brown lateral margins, posterior ½ dark brown (Fig. 619).

Ratios. HE/MS/WM = 2.2/1.0/1.2; POL/OOL/POO = 5.8/3.8/1.0; OOL/DO = 1.3; WE/WF/WH/HH = 1.0/2.5/4.6/3.1; WH/WT = 1.1; PM/ST = 2.1; TS1/TS2/LT/LT1/LT2/LT3/LT4 = 3.8/2.5/6.2/2.3/1.5/1.0/1.5; LP/WP = 0.9; MM/LG = 1.1.

*Male*. Length of body 1.8 mm. Scape white, expanded and widest close to base (Fig. 622), sensory pores confined to a wide groove along entire ventral margin. Otherwise similar to female except legs paler: fore and mid coxae white, femora yellowish-white in basal ½, yellowish-brown in apical ½, tibiae and tarsi yellowish-brown; petiole longer; gaster with anterior ½ dusky white with dark brown lateral margins (Fig. 620), and shorter.

Ratios. LC/WS = 2.7; LP/WP = 1.2; MM/LG = 1.5.

##### Hosts and biology.

*Dasylophia* maxtlaDHJ06 (Schauff and Janzen 2001); **new records:** *Chliara croesus* feeding on *Andira inermis*, *Colax apulusDHJ01* feeding on *Pterocarpus orbiculatus*, *Dasylophia guarana* feeding on *Platymiscium parviflorum*, *Hapigiodes sigifredomarini* feeding on *Lonchocarpus guatemalensis*, *Pentobesa* pinnaDHJ02 feeding on *Inga vera* and *Inga punctata*. All hosts are Notodontidae, and all host plants are Fabaceae.

##### Distribution.

Costa Rica (Guanacaste Province).

#### Euplectrus mariae

Taxon classificationAnimaliaHymenopteraEulophidae

Schauff

Figures 20 , 623–629 , 633–635 , 795

Euplectrus mariae Schauff in Schauff & Janzen, 2001: 214–216. Holotype ♀ (INBio), not examined.

##### Material.

Type material: 1♀ 1♂ paratypes of *Euplectrus mariae* (BMNH). Additional material: Costa Rica: 41♀ 7♂ from Guanacaste Province; caterpillar project voucher codes: 92-SRNP-993, 92-SRNP-2923, 92-SRNP-2924, 92-SRNP-3032, 92-SRNP-3141, 92-SRNP-3142, 92-SRNP-3297, 93-SRNP-1577, 93-SRNP-1582, 93-SRNP-1585, 94-SRNP-1055, 94-SRNP-1063, 94-SRNP-1071, 94-SRNP-1075, 94-SRNP-1081, 94-SRNP-1088, 94-SRNP-1090, 94-SRNP-1160, 94-SRNP-1216, 94-SRNP-1217, 96-SRNP-1314, 09-SRNP-72128 (BMNH, CNC, INBio, MZLU, MIUCR, USNM).

##### Diagnosis.

**Figures 623–629.**
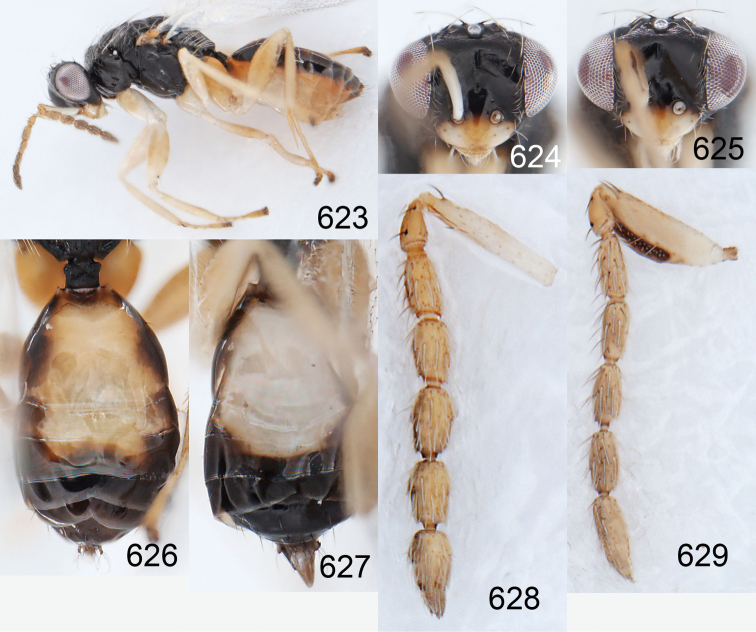
*Euplectrus mariae* (non-types): **623** habitus in lateral view, female **624** head in frontal view, female **625** head in frontal view, male **626** gaster in dorsal view, female **627** gaster in dorsal view, male **628** antenna in lateral view, female **629** antenna in lateral view, male.

**Figures 630–635.**
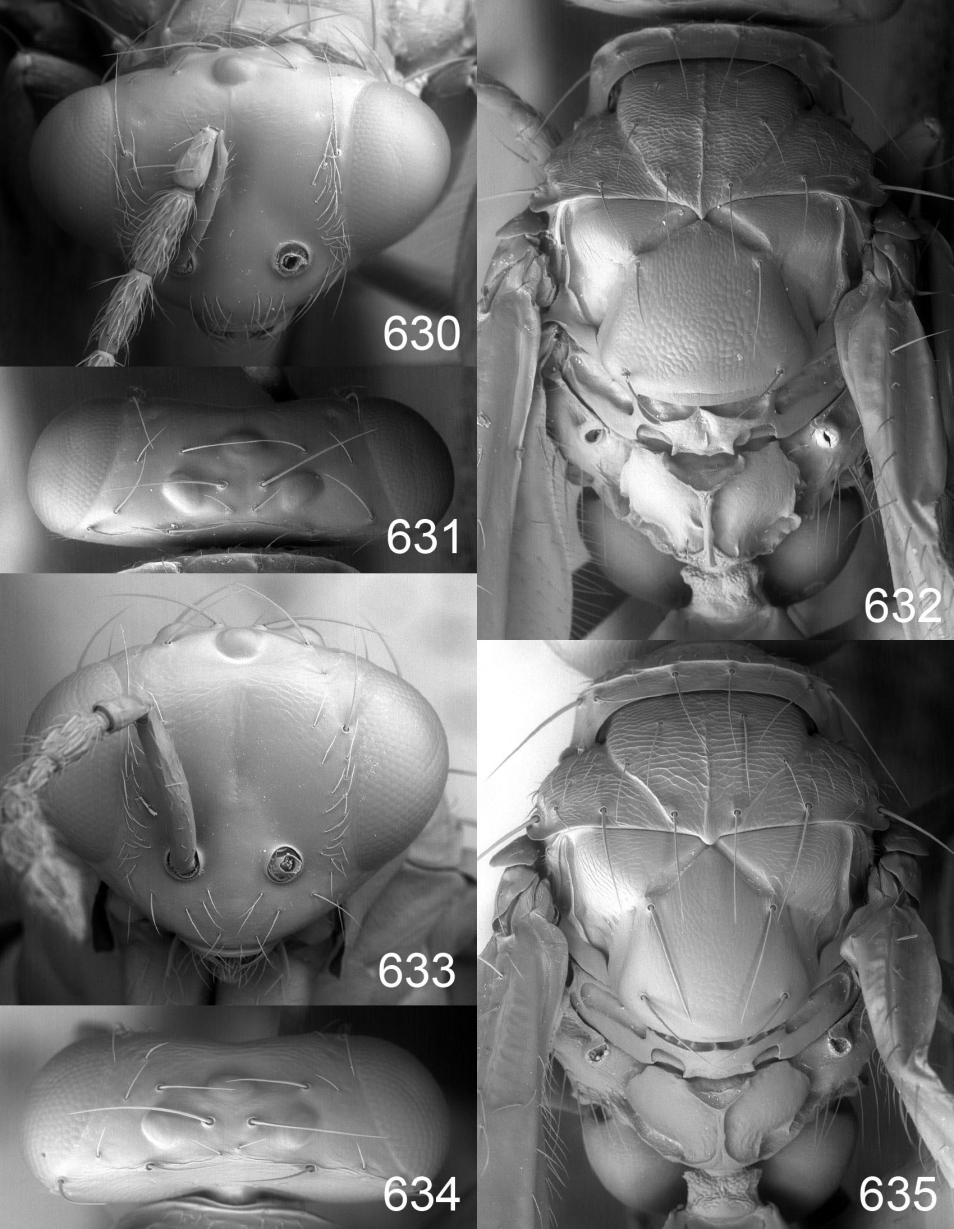
*Euplectrus* spp., females. **630–632** *Euplectrus magdae* (non-types): **630** head in frontal view **631** vertex **632** mesosoma in dorsal view **633–635** *Euplectrus mariae* (non-types): **633** head in frontal view **634** vertex **635** mesosoma in dorsal view.

Lower face medially with median part yellowish-brown and lateral parts white, pale area reaching half-way between outer lateral margins of toruli and eyes (Figs 624, 625); fore and mid legs yellowish-white, hind leg yellowish-brown (Fig. 623); petiole 0.6× as long as wide in female, 0.8× in male; gaster with anterior ½ yellowish-brown (female, Fig. 626) or white (male, Fig. 627) with dark brown lateral margins, posterior ½ dark brown; male antenna with scape slightly expanded and widest in the middle, 2.9× as long as wide (Fig. 629), and with apicoventral sensory area dark brown.

##### Description.

*Female*. Length of body 2.3 mm. Antenna with scape yellowish-white, pedicel and flagellomeres 1–2 yellowish-brown, 3–6 yellowish-brown ventrally and dark brown dorsally (Fig. 628). Mandibles and palpi yellowish-white. Head black and shiny, lower face medially with median part yellowish-brown and lateral parts white, reaching half-way between outer lateral margins of toruli and eyes, parts between pale area and eyes black (Fig. 624). Frons close to eyes with two rows of setae (Fig. 633). Vertex smooth (Fig. 634). Occipital margin with a weak carina (Fig. 634).

Mesosoma black and shiny (Fig. 623). Each sidelobe of mesoscutum with 15 setae. Scutellum 1.0× as long as wide; with very weak engraved reticulation (Fig. 635). Dorsellum anteriorly with a groove that is divided by longitudinal carinae (Fig. 795), groove medially 0.2× as long as length of dorsellum. Propodeum with very weak reticulation (Fig. 795); anteromedially with a transverse triangular cup; propodeal callus with ten setae. Legs (Fig. 623): fore and mid legs yellowish-white, hind leg yellowish-brown. Fore wing: costal cell with one row of setae on ventral surface, and margin with three setae close to marginal vein; with 15 admarginal setae, in one row.

Gaster with anterior ½ yellowish-brown with dark brown lateral margins, posterior ½ dark brown (Fig. 626).

Ratios. HE/MS/WM = 2.1/1.0/1.1; POL/OOL/POO = 8.2/4.2/1.0; OOL/DO = 1.1; WE/WF/WH/HH = 1.0/2.5/4.7/3.7; WH/WT = 1.0; PM/ST = 1.9; TS1/TS2/LT/LT1/LT2/LT3/LT4 = 3.8/2.5/6.3/2.1/1.4/1.0/2.1; LP/WP = 0.6; MM/LG = 0.9.

*Male*. Length of body 1.9 mm. Scape slightly expanded and widest in the middle, sensory pores confined to apicoventral 2/3 and with sensory area dark brown (Fig. 629). Otherwise similar to female except legs yellowish-white with hind coxa and hind femur slightly darker; petiole longer; gaster shorter and with pale parts white (Fig. 627).

Ratios. LC/WS = 2.9; LP/WP = 0.8; MM/LG = 1.3.

##### Hosts and biology.

*Concana hoshea*, *Concana* Poole01, *Concana* Poole02 (Noctuidae) (Schauff and Janzen 2001) feeding on *Byrsonima crassifolia*, *Heteropterys laurifolia*, and *Hiraea reclinata* (all host plants are Malpighiaceae).

##### Distribution.

Costa Rica (Guanacaste Province) (Schauff and Janzen 2001).

#### Euplectrus orias

Taxon classificationAnimaliaHymenopteraEulophidae

Schauff

Figures 417–420

Euplectrus orias Schauff in Schauff & Janzen, 2001: 216–217. Holotype ♀ (INBio), not examined.

##### Material.

Type material: 1♀ paratype of *Euplectrus orias* (BMNH).

##### Diagnosis.

Head dark brown and shiny, lower face medially pale brown (Fig. 418); scutellum almost smooth with only very weak traces of reticulation; legs yellowish-brown with hind coxa dark brown and hind femur pale brown (Fig. 417); fore wing submarginal vein with four setae; petiole 0.6× as long as wide; gaster with anterior ½ yellowish-white with dark brown lateral margins, posterior ½ dark brown (Fig. 419).

##### Description.

*Female*. Length of body 1.3 mm. Antenna with scape and pedicel yellowish-brown, flagellomeres dark brown (Fig. 420). Mandibles and palpi yellowish-white. Head dark brown and shiny, lower face medially pale brown (Fig. 418). Frons close to eyes with one row of setae. Vertex smooth. Occipital margin with a carina.

Mesosoma dark brown and shiny (Fig. 417). Each sidelobe of mesoscutum with eight setae. Scutellum 1.0× as long as wide; almost smooth with only very weak traces of reticulation. Dorsellum anteriorly with a groove that is divided by longitudinal carinae. Propodeum smooth; anteromedially with a transverse semicircular cup; propodeal callus with six setae. Legs yellowish-brown with hind coxa dark brown and hind femur pale brown (Fig. 417). Fore wing: costal cell on ventral surface with one row of setae, and margin with one seta close to marginal vein; with 13 admarginal setae, in one row.

Gaster with anterior ½ yellowish-white with dark brown lateral margins, posterior ½ dark brown (Fig. 419).

Ratios. HE/MS/WM = 2.5/1.3/1.0; POL/OOL/POO = 4.8/3.0/1.0; OOL/DO = 1.5; WE/WF/WH/HH = 1.0/3.5/5.5/4.5; WH/WT = 1.2; PM/ST = 1.5; TS1/TS2/LT/LT1/LT2/LT3/LT4 = 3.8/2.4/6.2/2.5/1.3/1.0/1.3; LP/WP = 0.6; MM/LG = 1.1.

*Male*. Unknown.

##### Hosts and biology.

*Oxydia* sociataDHJ02 (Geometridae) feeding on *Picramnia antidesma* (Simaroubaceae) (Schauff and Janzen 2001).

##### Distribution.

Costa Rica (Guanacaste Province) (Schauff and Janzen 2001).

#### Euplectrus platyhypenae

Taxon classificationAnimaliaHymenopteraEulophidae

Howard

Figures 402–403 , 404–409 , 410–420 , 421–427 , 428–434 , 435–440 , 441–452 , 453–463 , 464–469 , 470–478 , 479–488 , 489–494 , 636–643 , 796

Euplectrus platyhypenae Howard, 1885: 26. Lectotype ♂ (USNM), designated here, examined.

##### Material.

Type material: ♂ lectotype of *Euplectrus platyhypenae* (USNM). Additional material: COSTA RICA: 12♀ 1♂ from Guanacaste on *Spodoptera frugiperda*; caterpillar project voucher code: 05-SRNP-16047, no barcode (BMNH, CNC, INBio, USNM).

##### Diagnosis.

**Figures 636–643.**
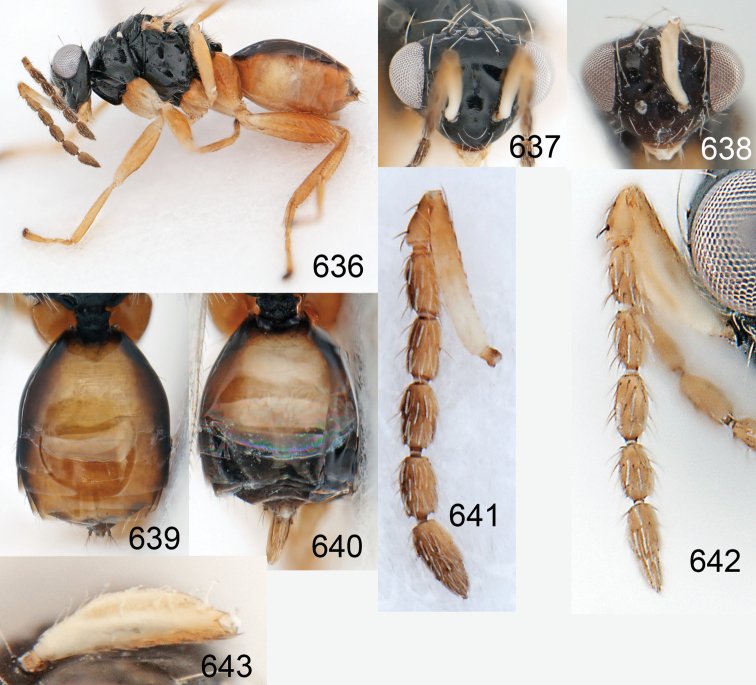
*Euplectrus platyhypenae* (non-types): **636** habitus in lateral view, female **637** head in frontal view, female **638** head in frontal view, male **639** gaster in dorsal view, female **640** gaster in dorsal view, male **641** antenna in lateral view, female **642** antenna in lateral view, male **643** scape in lateral view, male.

Lower face black (Figs 637, 638); legs yellowish-brown (Fig. 636); eyes small (Fig. 492); propodeum with a triangular median carina (Fig. 796); petiole 0.6× as long as wide; gaster yellowish-brown with black lateral margins (female, Fig. 639), or black with a large yellowish-white spot in apical ½ (male, Fig. 640); male antenna with scape slightly expanded, widest in the middle, and 3.1× as long as wide (Fig. 643).

##### Description.

*Female*. Length of body 2.1 mm. Antenna with scape yellowish-white in basal ½ and yellowish-brown in apical ½, pedicel yellowish-brown, flagellomeres yellowish-brown ventrally and dark brown dorsally (Fig. 641). Mandibles and palpi yellowish-white. Head including lower face black and shiny (Fig. 637). Frons close to eyes with two irregular rows of setae (Fig. 492). Vertex with very weak reticulation, smooth lateral to posterior ocelli (Fig. 493). Occipital margin rounded (Fig. 493).

Mesosoma black and shiny (Fig. 636). Each sidelobe of mesoscutum with eight setae. Scutellum 0.9× as long as wide; with weak reticulation (Fig. 494). Dorsellum along anterior margin with a very narrow groove that is divided by longitudinal carinae (Fig. 796), groove medially 0.1× as long as length of dorsellum. Propodeum with very weak reticulation (Fig. 796); anteromedially with a triangular cup; propodeal callus with 15 setae. Legs yellowish-brown (Fig. 636). Fore wing: costal cell with one complete row of setae on ventral surface, and margin with three setae close to marginal vein; with 18 admarginal setae mainly in one row, in apical ⅓ in two rows.

Gaster yellowish-brown with lateral margins black (Fig. 639).

Ratios. HE/MS/WM = 1.5/1.0/1.0; POL/OOL/POO = 6.1/3.6/1.0; OOL/DO = 1.5; WE/WF/WH/HH = 1.0/2.8/4.6/3.4; WH/WT = 1.1; PM/ST = 1.4; TS1/TS2/LT/LT1/LT2/LT3/LT4 = 4.7/2.4/7.8/3.1/1.6/1.0/1.9; LP/WP = 0.6; MM/LG = 1.0.

*Male*. Length of body 1.7 mm. Scape yellowish-white, slightly expanded and widest in the middle (Fig. 643), sensory pores confined to apicoventral ⅔. Similar to female except gaster shorter and black with a large yellowish-white spot in apical ½ (Fig. 640).

Ratios. LC/WS = 3.1; MM/LG = 1.3.

##### Hosts and biology.

*Plathypena scabra* (Howard 1885), *Spodoptera frugiperda* feeding on *Cipura campanulata* (Iridaceae) (**new record**) (both hosts are Noctuidae).

##### Distribution.

USA (D.C.) (Howard 1885), Costa Rica (Guanacaste Province) (**new record**).

##### Remarks.

*Euplectrus platyhypenae* was described from one female and one male but no primary type was selected. The male labeled “Euplectrus platyhypenae How. ms.”, “No.657, P.o, July 11.82”, “Type No. 1655, U.S.N.M.”, is hereby selected as lectotype.

The name of this species is frequently misspellt in the literature, as “*Euplectrus plathypenae*”, as is also the host *Spodoptera frugiperda*, as “*Spodoptera fugiperda*”.

#### Euplectrus ronniei

Taxon classificationAnimaliaHymenopteraEulophidae

Schauff

Figures 651–655

Euplectrus ronniei Schauff, in Schauff & Janzen, 2001: 219–220. Holotype ♀ (INBio), not examined.

##### Material.

Type material: 1♀ paratype of *Euplectrus ronniei* (USNM). Additional material: 1♀ 1♂ with same label data as female paratype (USNM), but not included in the original description.

##### Diagnosis.

Lower face medially white with median ⅓ pale yellowish-brown, extending to half-way between level of outer lateral margins of toruli and eyes (Fig. 652); legs yellowish-white with hind coxae pale yellowish-brown (Fig. 651); petiole 0.7× as long as wide; gaster with anterior ½ yellowish-white (female, Fig. 653) or white (male, Fig. 654) with dark brown anterior and lateral margins, posterior ½ dark brown. Very similar to *Euplectrus chrisgrinteri* the only differences are that *Euplectrus ronniei* has shorter petiole (both sexes) and different host preferences, possibly also in the characters of male scape – but the appearance of the male scape in *Euplectrus ronniei* is not known (head is missing in single known male specimen).

##### Description.

*Female*. Length of body 2.2 mm. Antenna (missing in non-type specimen) with scape white with apex and pedicel yellowish-brown, flagellum yellowish-brown with ventral parts pale yellowish-brown (Fig. 655). Mandibles and palpi white. Head black and shiny, lower face medially white with median ⅓ pale yellowish-brown, extending to half-way between level of outer lateral margins of toruli and eye, parts between pale area and eyes black (Fig. 652). Frons close to eyes with two rows of setae. Vertex smooth. Occipital margin rounded.

Mesosoma black and shiny (Fig. 651). Each sidelobe of mesoscutum with 12 setae. Scutellum 0.9× as long as wide; with weak engraved reticulation, posterior margin smooth. Dorsellum along anterior margin with a groove that is divided by longitudinal carinae. Propodeum with very weak reticulation; anteromedially with a triangular cup that has posterior part strongly raised; propodeal callus with seven setae. Legs yellowish-white with hind coxa pale yellowish-brown (Fig. 651). Fore wing: costal cell on ventral surface predominantly with one row of setae, two rows at base, and margin with three setae close to marginal vein; with 15 admarginal setae, in one row.

Gaster with anterior ½ yellowish-white with dark brown anterior and lateral margins, posterior ½ dark brown (Fig. 653).

Ratios. HE/MS/WM = 2.0/1.0/1.1; POL/OOL/POO = 6.0/2.7/1.0; OOL/DO = 1.2; WE/WF/WH/HH = 1.0/2.8/5.1/3.7; WH/WT = 1.0; PM/ST = 1.3; TS1/TS2/LT/LT1/LT2/LT3/LT4 = 4.4/2.7/7.1/2.4/1.6/1.0/1.9; LP/WP = 0.7; MM/LG = 1.1.

*Male*. Length of body without head 1.5 mm. Similar to female except shorter gaster with pale area white (Fig. 654). The head is missing in single available male specimen.

Ratios. MM/LG = 1.2.

##### Hosts and biology.

*Cautethia spuria* (Sphingidae) feeding on *Exostema mexicanum* (Rubiaceae) (Schauff and Janzen 2001).

##### Distribution.

Costa Rica (Guanacaste Province) (Schauff and Janzen 2001).

##### Remarks.

The type series of *Euplectrus ronniei* contains two females, one the holotype, from *Cautethia spuria* (Sphingidae), and three males reared from *Oxidercia toxea* (Noctuidae). The specimens from *Cautethia*, among them the holotype, belong to a different species than the specimens from *Oxidercia*. As the holotype of *Euplectrus ronniei* is from *Cautethia spuria*, being the name-bearing type, these two females are *Euplectrus ronniei*. The male specimens from *Oxidercia* belong to a different species, possibly *Euplectrus garygibsoni*, which has the same host, but some morphological features do not agree between the females of *Euplectrus garygibsoni* and the males that formerly were *Euplectrus ronniei*. There are no barcode for the specimens from *Oxidercia*. The identity of the males from *Oxidercia* remains unsolved until further information becomes available.

#### Euplectrus testaceipes

Taxon classificationAnimaliaHymenopteraEulophidae

(Cameron)

Figures 6–8 , 9–11 , 12–13 , 644–650 , 656–658 , 797

Rekabia testaceipes Cameron, 1904: 66. Lectotype ♂ (BMNH), examined.
Euplectrus testaceipes (Cameron) (Kerrich 1974: 636).
Elachistus carinatus Cameron, 1913: 126. Lectotype ♀ (BMNH), examined. Synonymized by Bouček (1977: 11).
Euplectrus walteri Schauff in Schauff & Janzen, 2001: 224–225. Holotype ♀ (INBio), not examined. **New synonym.**

##### Material.

Type material: male lectotype of *Rekabia testaceipes* (BMNH), female lectotype of *Elachistus carinatus* (BMNH), 1♀ 1♂ paratypes of *Euplectrus walteri* (BMNH). Additional material: COSTA RICA: 31♀ 8♂ from Guanacaste Province; caterpillar project voucher codes: 84-SRNP-623, 84-SRNP-805, 91-SRNP-1636, 92-SRNP-2262, 92-SRNP-3018, 92-SRNP-3174, 92-SRNP-3708, 92-SRNP-3811, 92-SRNP-4114, 93-SRNP-2239, 95-SRNP-7544, 96-SRNP-6830, 96-SRNP-10435, 97-SRNP-3135, 98-SRNP-10806, 01-SRNP-14301, 02-SRNP-7759, 05-SRNP-46152, 07-SRNP-31879, 08-SRNP-16396, 08-SRNP-13885, 10-SRNP-72733 (BMNH, CNC, INBio, MZLU, MIUCR, USNM).

##### Diagnosis.

**Figures 644–655.**
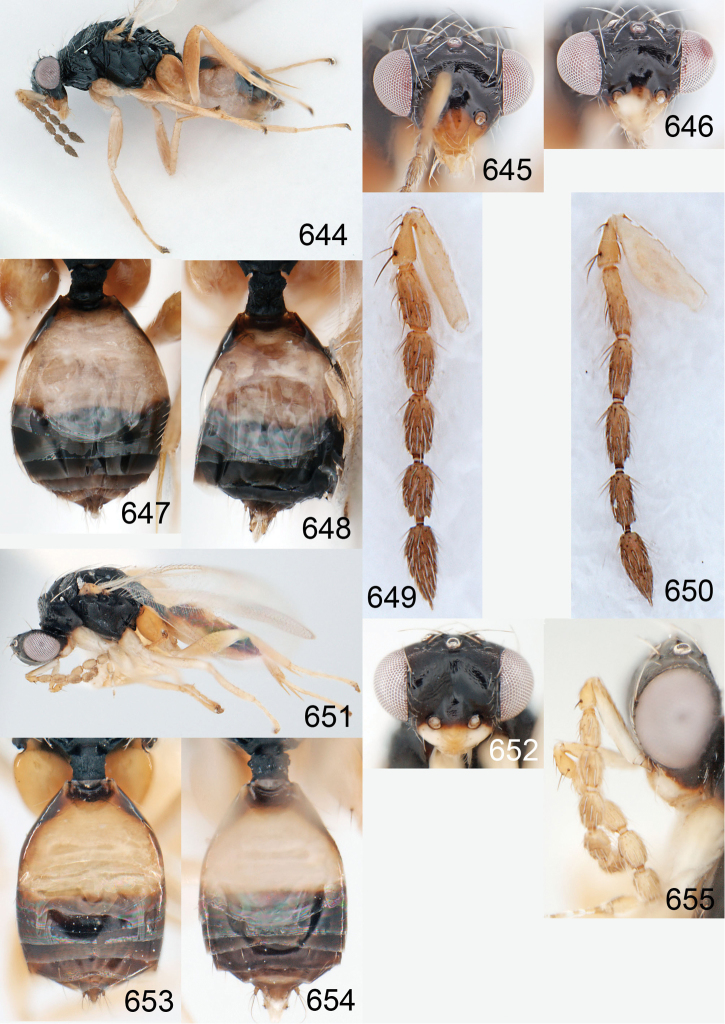
*Euplectrus* spp. **644–650** *Euplectrus testaceipes* (non-types): **644** habitus in lateral view, female **645** head in frontal view, female **646** head in frontal view, male **647** gaster in dorsal view, female **648** gaster in dorsal view, male **649** antenna in lateral view, female **650** antenna in lateral view, male; **651–655** *Euplectrus ronniei*: **651** habitus in lateral view, female (paratype) **652** head in frontal view, female (non-type) **653** gaster in dorsal view, female (non-type) **654** gaster in dorsal view, male (non-type) **655** antenna in lateral view, female (paratype).

Lower face pointed downwards, medially yellowish-brown (female, Fig. 645) or yellowish-white (male, Fig. 646), pale area reaching to level of outer lateral margin of toruli and up between toruli; legs yellowish-brown (Fig. 644); dorsellum with a wide groove along anterior margin (Fig. 797), medially 0.4× as long as length of dorsellum; propodeum with a wide median carina (Fig. 797); gaster with anterior ½ white with dark brown lateral margins that are broken posteriorly, posterior ½ black, in female with apex reddish-brown (Fig. 647); male antenna with scape slightly expanded, widest in the middle, 3.0× as long as wide (Fig. 650); petiole 1.0× as long as wide in female, 1.1× in male, with a strong seta anterolaterally.

##### Description.

**Figures 656–661.**
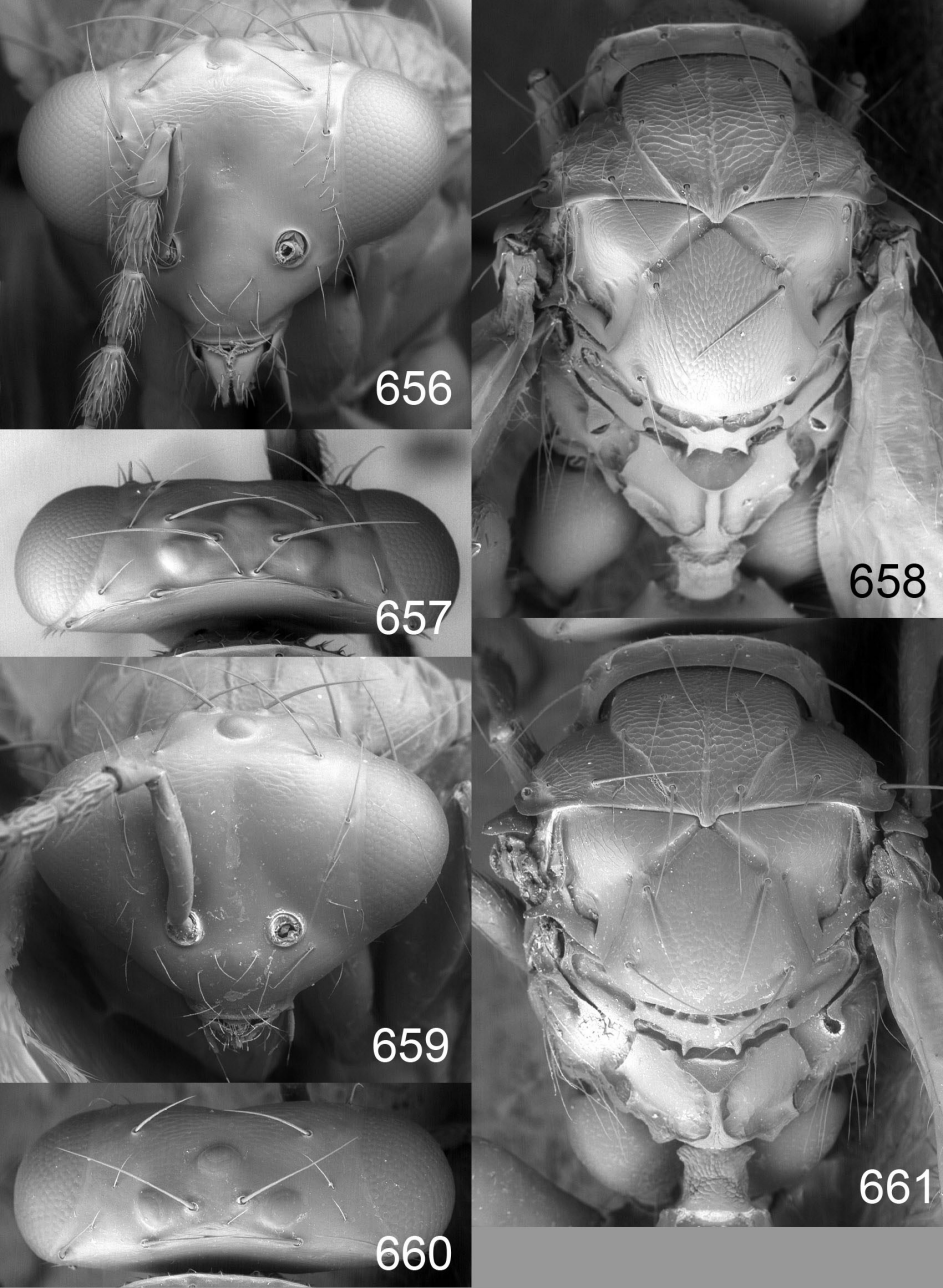
*Euplectrus* spp., females. **656–658** *Euplectrus testaceipes* (non-types): **656** head in frontal view **657** vertex **658** mesosoma in dorsal view; **659–661** *Euplectrus xiomarae* (non-types): **659** head in frontal view **660** vertex **661** mesosoma in dorsal view.

*Female*. Length of body 2.9 mm. Antenna with scape yellowish-white, pedicel yellowish-brown, flagellomere 1 pale brown, 2–6 dark brown (Fig. 649). Mandibles and palpi yellowish-white. Head black and shiny, lower face medially yellowish-brown, pale area reaching to level of outer margin of toruli and up between toruli (Fig. 645). Frons close to eyes with one row of setae in lower ½, with two irregular rows in upper ½ (Fig. 656). Vertex smooth (Fig. 657). Occipital margin with a carina behind ocellar triangle (Fig. 657).

Mesosoma black and shiny (Fig. 644). Each sidelobe of mesoscutum with 15 setae. Scutellum 0.9× as long as wide; with rather strong engraved reticulation (Fig. 658). Dorsellum along anterior margin with a wide groove (Fig. 797), medially 0.4× as long as length of dorsellum. Propodeum smooth (Fig. 797); anteromedially with a semicircular cup; propodeal callus with seven setae. Legs yellowish-brown (Fig. 644). Fore wing: costal cell with two complete rows of setae on ventral surface, and margin with five setae close to marginal vein; with 19 admarginal setae in one row.

Petiole with a strong seta anterolaterally. Gaster with anterior ½ white with dark brown lateral margins that are broken posteriorly, posterior ½ black with apex reddish-brown (Fig. 647).

Ratios. HE/MS/WM = 2.1/1.0/1.3; POL/OOL/POO = 8.4/4.4/1.0; OOL/DO = 1.3; WE/WF/WH/HH = 1.0/2.3/4.3/3.1; WH/WT = 1.1; PM/ST = 1.7; TS1/TS2/LT/LT1/LT2/LT3/LT4 = 4.0/2.5/7.3/2.8/1.8/1.0/1.5; LP/WP = 1.0; MM/LG = 1.1.

*Male*. Length of body 2.5 mm. Scape slightly expanded and widest in the middle (Fig. 650), sensory pores confined to apicoventral ⅔. Similar to female except antenna with flagellum longer and more slender (Fig. 650); lower face with pale area yellowish-white (Fig. 646); petiole longer; gaster shorter.

Ratios. LC/WS = 3.0; LP/WP = 1.1; MM/LG = 1.3.

##### Hosts and biology.

*Manduca dilucida* feeding on *Tabebuia ochracea* (Bignoniaceae); *Manduca florestan* feeding on *Aegiphila martinicensis*, *Cornutia grandifolia* (Lamiaceae), *Cydista heterophylla*, *Pithecoctenium crucigerum*, *Tabebuia ochracea* (Bignoniaceae), *Cordia alliodora* (Boraginaceae), *Gmelina arborea* (Verbenaceae); *Manduca lanuginosa* feeding on *Cydista heterophylla*; *Manduca rustica* feeding on *Aegiphila martinicensis* (Lamiaceae), *Merremia umbellata* (Convolvulaceae); *Manduca sexta* feeding on *Capsicuum annuum* (Solanaceae) (all hosts are Sphingidae).

##### Distribution.

Costa Rica (Guanacaste Province) (Schauff and Janzen 2001), Guyana (Cameron 1913), Nicaragua (Cameron 1904).

#### Euplectrus xiomarae

Taxon classificationAnimaliaHymenopteraEulophidae

Schauff

Figures 27 , 659–661 , 662–668 , 798

Euplectrus xiomarae Schauff in Schauff & Janzen, 2001: 226–227. Holotype ♀ (INBio), not examined.

##### Material.

Type material: 1♀ 1♂ paratypes of *Euplectrus xiomarae* (BMNH). Additional material: COSTA RICA: 59♀ 5♂ (dried and mounted) from Guanacaste Province; caterpillar project voucher codes: 92-SRNP-4614, 92-SRNP-4642, 05-SRNP-19390, 05-SRNP-19416, 05-SRNP-19573, 07-SRNP-24233, 07-SRNP-46304, 07-SRNP-47004 (BMNH, CNC, INBio, MZLU, MIUCR, USNM). Additional material in alcohol with following caterpillar project voucher codes: 92-SRNP-4602, 92-SRNP-4609, 92-SRNP-4652, 93-SRNP-4384, 93-SRNP-4388, 05-SRNP-19377, 05-SRNP-19354, 05-SRNP-19351, 05-SRNP-19352, 05-SRNP-19558, 05-SRNP-19375, 05-SRNP-47274, 05-SRNP-19987, 05-SRNP-19363, 07-SRNP-24233, 07-SRNP-46365, 07-SRNP-46741, 07-SRNP-46944, 07-SRNP-46365, 07-SRNP-46944, 07-SRNP-46741, 07-SRNP-47385, 07-SRNP-15585, 07-SRNP-46712, 07-SRNP-15652, 07-SRNP-46169, 07-SRNP-15320, 07-SRNP-15327, 07-SRNP-46373, 07-SRNP-47448, 07-SRNP-46810, 07-SRNP-47104, 07-SRNP-47085, 07-SRNP-46475, 07-SRNP-46443, 07-SRNP-46074, 07-SRNP-15675, 07-SRNP-46140, 07-SRNP-15354, 07-SRNP-36651, 07-SRNP-46480, 07-SRNP-46075, 07-SRNP-46459, 07-SRNP-65671, 07-SRNP-46407, 07-SRNP-15731, 07-SRNP-46138, 07-SRNP-46031, 07-SRNP-46011, 07-SRNP-15657, 07-SRNP-46378, 07-SRNP-15661, 07-SRNP-15654, 07-SRNP-33250, 07-SRNP-15326, 07-SRNP-15735, 07-SRNP-33390, 07-SRNP-47261, 07-SRNP-47399, 07-SRNP-47390, 07-SRNP-46721, 07-SRNP-15736, 07-SRNP-46555, 07-SRNP-15819, 07-SRNP-46612, 07-SRNP-24257, 07-SRNP-47216, 07-SRNP-34433.

##### Diagnosis.

**Figures 662–668.**
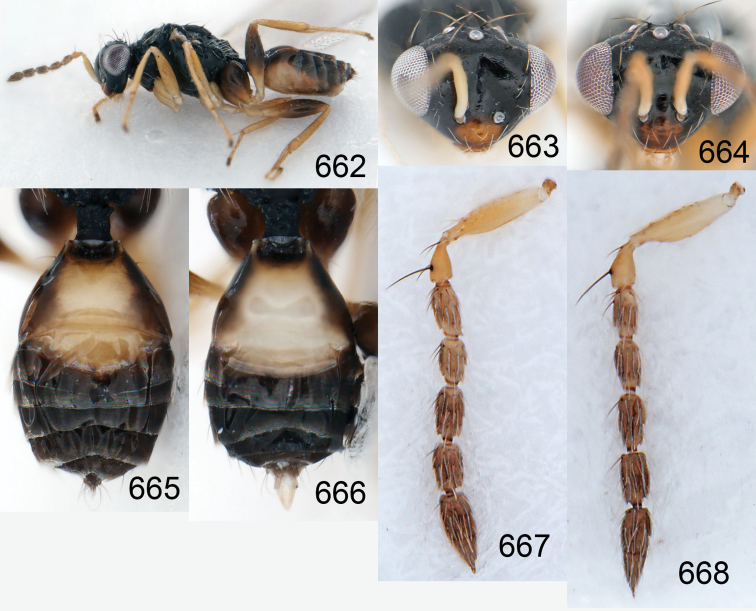
*Euplectrus xiomarae* (non-types): **662** habitus in lateral view, female **663** head in frontal view, female **664** head in frontal view, male **665** gaster in dorsal view, female **666** gaster in dorsal view, male **667** antenna in lateral view, female **668** antenna in lateral view, male.

Lower face medially yellowish-brown, pale area reaching to level of middle of toruli (Figs 663, 664); fore and mid legs yellowish-brown, hind leg with coxa dark brown, femur dark brown with basal ⅓ yellowish-brown, tibia and tarsus yellowish-brown (Fig. 662); petiole 1.0× as long as wide; gaster with anterior ½ yellowish-brown (female, Fig. 665) or white (male, Fig. 666) with black lateral margins, posterior ½ black; male scape slightly expanded, widest in the middle, 3.7× as long as wide (Fig. 668).

##### Description.

*Female*. Length of body 2.1 mm. Antenna with scape and pedicel yellowish-brown, flagellomeres 1–2 pale brown, 3–6 dark brown (Fig. 667). Mandibles yellowish-white, palpi white. Head black and shiny, lower face medially yellowish-brown, pale area reaching to level of middle of toruli (Fig. 663). Frons close to eyes with one row of setae (Fig. 659). Vertex with very weak reticulation, smooth lateral to posterior ocelli (Fig. 660). Occipital margin rounded (Fig. 660).

Mesosoma black and shiny (Fig. 662). Each sidelobe of mesoscutum with seven setae. Scutellum 0.9× as long as wide; with weak reticulation (Fig. 661). Dorsellum along anterior margin with a groove that is divided by longitudinal carinae (Fig. 798), groove medially 0.3× as long as length of dorsellum. Propodeum with very weak reticulation (Fig. 798); anteromedially with a triangular cup; propodeal callus with 11 setae. Legs (Fig. 662): fore and mid legs yellowish-brown, hind leg with coxa dark brown, femur dark brown with basal ⅓ yellowish-brown, tibia and tarsus yellowish-brown. Fore wing: costal cell on ventral surface with two complete rows of setae, and margin with four setae close to marginal vein; with 17 admarginal setae mainly in one row, in apical ⅓ in two rows.

Gaster with anterior ½ yellowish-brown with black lateral margins, posterior ½ black (Fig. 665).

Ratios. HE/MS/WM = 1.7/1.0/1.2; POL/OOL/POO = 8.2/6.2/1.0; OOL/DO = 1.5; WE/WF/WH/HH = 1.0/3.0/5.1/3.8; WH/WT = 1.1; PM/ST = 1.8; TS1/TS2/LT/LT1/LT2/LT3/LT4 = 4.0/2.2/6.7/2.4/1.7/1.0/1.7; LP/WP = 1.0; MM/LG = 1.1.

*Male*. Length of body 1.8 mm. Scape yellowish-white, slightly expanded and widest in the middle (Fig. 668); sensory pores confined to apicoventral ½. Similar to female except shorter gaster that has pale parts white (Fig. 666).

Ratios. LC/WS = 3.7; MM/LG = 1.3.

##### Hosts and biology.

*Hemiceras* and *Rosema* spp. (Notodontidae) feeding on *Inga* spp. (Fabaceae): *Hemiceras clarki* feeding on *Inga vera*, *Hemiceras corema* feeding on *Inga oerstediana*, *Hemiceras nigrescens* feeding on *Inga punctata*, *Hemiceras sabis* feeding on *Inga oerstediana*, *Hemiceras vecina* feeding on *Inga sapindoides*, *Hemiceras zula* feeding on *Inga densiflora* and *Inga oerstediana*, *Hemiceras Janzen13* feeding on *Inga vera*, *Rosema attenuata* feeding on *Inga punctata*, *Rosema thestiaDHJ02* feeding on *Inga oerstediana*.

##### Distribution.

Costa Rica (Guanacaste Province) (Schauff and Janzen 2001).

### Species treatments – described species from the Americas, not found in ACG

#### Euplectrus catocalae

Taxon classificationAnimaliaHymenopteraEulophidae

Howard

Figures 669–675

Euplectrus catocalae Howard, 1885: 27. Lectotype ♂ (USNM), designated here, examined.

##### Material.

Type material: 2♀ 3♂ syntypes of *Euplectrus catocalae* (USNM). Additional material: 6♀ 1♂ (USNM) with same label data as syntypes, but not mentioned in the original description – see remarks below.

##### Diagnosis.

**Figures 669–680.**
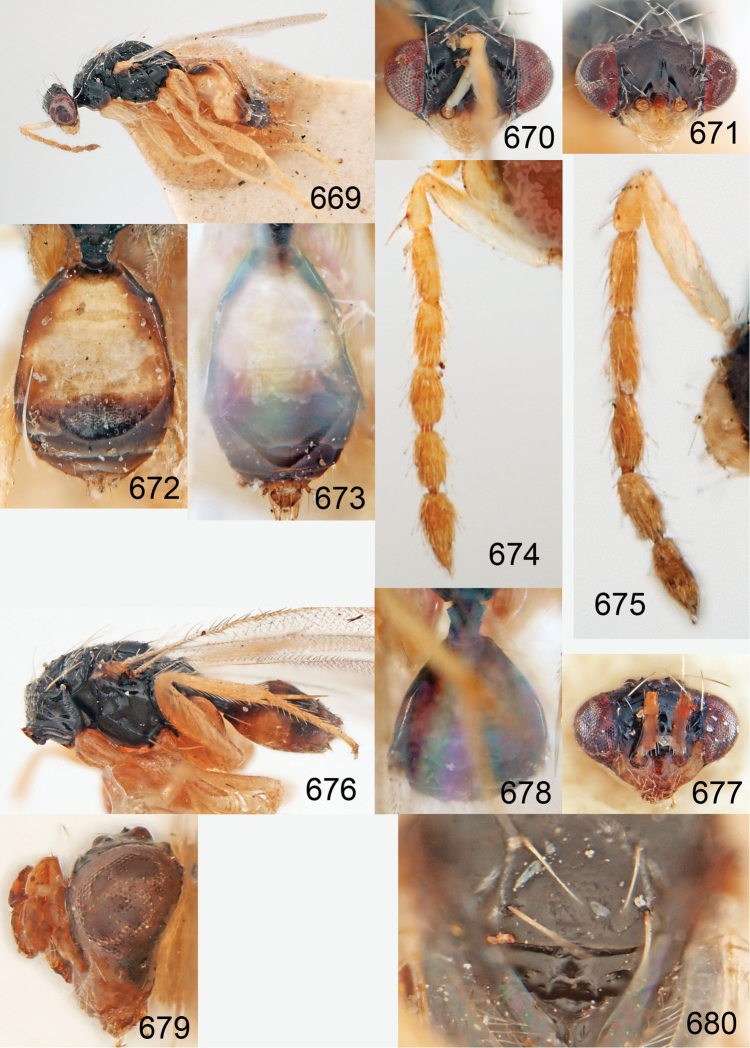
*Euplectrus* spp. **669–675** *Euplectrus catocalae* (paralectotypes (female), lectotype (male)): **669** habitus in lateral view, female **670** head in frontal view, female **671** head in frontal view, male **672** gaster in dorsal view, female **673** gaster in dorsal view, male **674** antenna in lateral view, female **675** antenna in lateral view, male; **676–680** *Euplectrus chapadae* (lectotype), female: **676** habitus in lateral view, head missing **677** head in frontal view **678** gaster in dorsal view **679** head in lateral view **680** dorsellum.

Lower face predominantly pale, medially yellowish-brown and laterally yellowish-white but with a black area the width of the width of scape between pale spot and eye margin (Figs 670, 671); vertex with parts lateral to ocellar triangle dark reddish-brown; gaster with anterior ½ yellowish-white with dark brown lateral margins, posterior ½ dark brown with apex reddish-brown (female, Fig. 672), or dark brown with a large white spot in anterior ½ (male, Fig. 673); legs yellowish-white, hind coxa slightly darker (Fig. 669); petiole 0.8× as long as wide.

##### Description.

*Female*. Length of body 2.3 mm. Antenna with scape yellowish-white, pedicel yellowish-brown, flagellomeres pale brown (Fig. 674). Mandibles and palpi yellowish-white. Head black and shiny, vertex with parts lateral to ocellar triangle dark reddish-brown, lower face predominantly pale, medially yellowish-brown and laterally yellowish-white, with a black area the width of the width of scape between pale spot and eye margin (Fig. 670). Frons close to eyes with two rows of setae. Vertex with very weak reticulation inside ocellar triangle, smooth outside ocellar triangle. Occipital margin with a weak carina behind ocellar triangle.

Mesosoma black and shiny (Fig. 669). Each sidelobe of mesoscutum with 11 setae. Scutellum 1.0× as long as wide; with engraved rather strong reticulation, meshes isodiametric. Dorsellum anteriorly with a narrow groove, reaching ¼ the length of dorsellum. Propodeum with very weak reticulation; anteromedially with a semicircular cup; propodeal callus with 13 setae. Legs yellowish-white, hind coxae slightly darker(Fig. 669). Wing veins and setae transparent; submarginal vein with four setae; costal cell with two irregular and sparse rows of setae on ventral surface, and margin with four setae close to marginal vein; with 12 admarginal setae, in one row.

Gaster with anterior ½ yellowish-white with dark brown lateral margins, posterior ½ dark brown with apex reddish-brown (Fig. 672).

Ratios. HE/MS/WM = 2.0/1.0/1.3; POL/OOL/POO = 6.8/3.8/1.0; OOL/DO = 1.3; WE/WF/WH/HH = 1.0/2.4/4.6/3.0; WH/WT = 1.0; PM/ST = 1.3; TS1/TS2/LT/LT1/LT2/LT3/LT4 = 3.3/1.8/6.7/2.1/1.3/1.0/2.0; LP/WP = 0.8; MM/LG = 0.9.

*Male*. Length of body 2.0 mm. Scape narrow and widest medially (Fig. 675); sensory area with same colour as remaining scape (yellowish-white). Otherwise similar to female.

Ratios. LC/WS = 4.8; MM/LG = 0.9.

##### Hosts and biology.

From a larva of *Catocala* sp. (Erebidae) (Howard 1885).

##### Distribution.

USA (Missouri) (Howard 1885).

##### Remarks.

The description of *Euplectrus catocalae* was based on two females and three males reared from a larva collected July 5 1873, in Saint Louis, Missouri. In the USNM there is a card with five points, each point with a specimen – two females and three males. These specimens are labeled “411 L. Par on Catocala”, “Type No. 2656 U.S.N.M.”, but there is no information on locality or collection date. However, as the number of specimens of each sex, the host, and the specimens agree with the description, these are strong indications that these specimens are the type material. The five specimens are from left to right: a female with head missing, a male with antennae missing, a female with antennae missing, a male with antennae missing, a male with left antenna missing. The male on the far right is designated lectotype and the remaining specimens are paralectotypes. In the USNM there is also another card with seven points, each point with a specimen – six females and one male, and with same label data as as the syntypes. There is no mention of these seven specimens in the original description and even though they are conspecific with the syntypes and probably from the same collecting event they are not type material. Six of these specimens lack the head, and the 7^th^ specimen, a female, lacks the flagellum on the right antenna.

#### Euplectrus chapadae

Taxon classificationAnimaliaHymenopteraEulophidae

Ashmead

Figures 676–680

Euplectrus chapadae Ashmead, 1904: 517. Lectotype ♀ (USNM), designated by LaSalle and Schauff 1992: 20, examined.

##### Material.

Type material: ♀ lectotype of *Euplectrus chapadae* (USNM).

##### Diagnosis.

Lower face completely pale, with median part reddish-brown and parts close to eyes yellowish-brown (Fig. 677); dorsellum with a narrow groove along anterior margin (Fig. 680); legs yellowish-brown (Fig. 676); petiole 1.0× as long as wide; gaster dark brown with a large pale spot in anterior ½ (Fig. 678).

##### Description.

*Female*. Length of body 2.1 mm. Antenna with scape and pedicel yellowish-brown, flagellomere 1 pale brown (Fig. 679), remaining flagellomeres missing. Mandibles and palpi yellowish-white. Head black and shiny, lower face completely pale (Fig. 677), with median part reddish-brown and parts close to eyes yellowish-brown. Frons close to eyes with two rows of setae. Vertex smooth. Occipital margin rounded.

Mesosoma black and shiny (Fig. 676). Each sidelobe of mesoscutum with 11 setae. Scutellum 1.1× as long as wide; with weak engraved reticulation and with lateral and posterior margins smooth. Dorsellum with a narrow groove along anterior margin, medially ¼ as long as length of dorsellum (Fig. 680). Propodeum smooth; anteromedially with a semicircular cup; propodeal callus with ten setae. Legs yellowish-brown (Fig. 676). Fore wing: costal cell with two rows of setae on ventral surface, and margin with four setae close to marginal vein; with 15 admarginal setae, in one row.

Gaster dark brown with a large pale spot in anterior ½ (Fig. 678).

Ratios. HE/MS/WM = 2.8/1.0/1.7; POL/OOL/POO = 5.0/4.0/1.0; OOL/DO = 1.3; WE/WF/WH/HH = 1.0/3.0/5.7/4.0; WH/WT = 1.1; PM/ST = 1.8; TS1/TS2/LT/LT1/LT2/LT3/LT4 = 4.7/2.7/7.3/2.6/1.7/1.0/1.7; LP/WP = 1.0; MM/LG = 1.5.

*Male*. Unknown.

##### Hosts and biology.

Unknown.

##### Distribution.

Brazil (Ashmead 1904).

##### Remarks.

According to LaSalle and Schauff (1992) the lectotype specimen of *Euplectrus chapadae* designated by them was a male. However, the specimen examined, which also has the lectotype label by LaSalle & Schauff, is a female.

The lectotype specimen is with head detached from the body and glued separately to the card. The specimen lacks all flagellomeres except the flagellomere 1 on both antennae, and left fore tarsus.

#### Euplectrus comstockii

Taxon classificationAnimaliaHymenopteraEulophidae

Howard

Figures 681–687

Euplectrus comstockii Howard, 1880: 159. Neotype female in USNM, designated by Schauff (2001), examined.

##### Material.

Type material: ♀ neotype of *Euplectrus comstockii* (USNM). Additional material: 3♀ 4♂ (USNM) from the series of specimens mentioned by Schauff and Janzen (2001), from which the neotype was selected.

##### Diagnosis.

**Figures 681–691.**
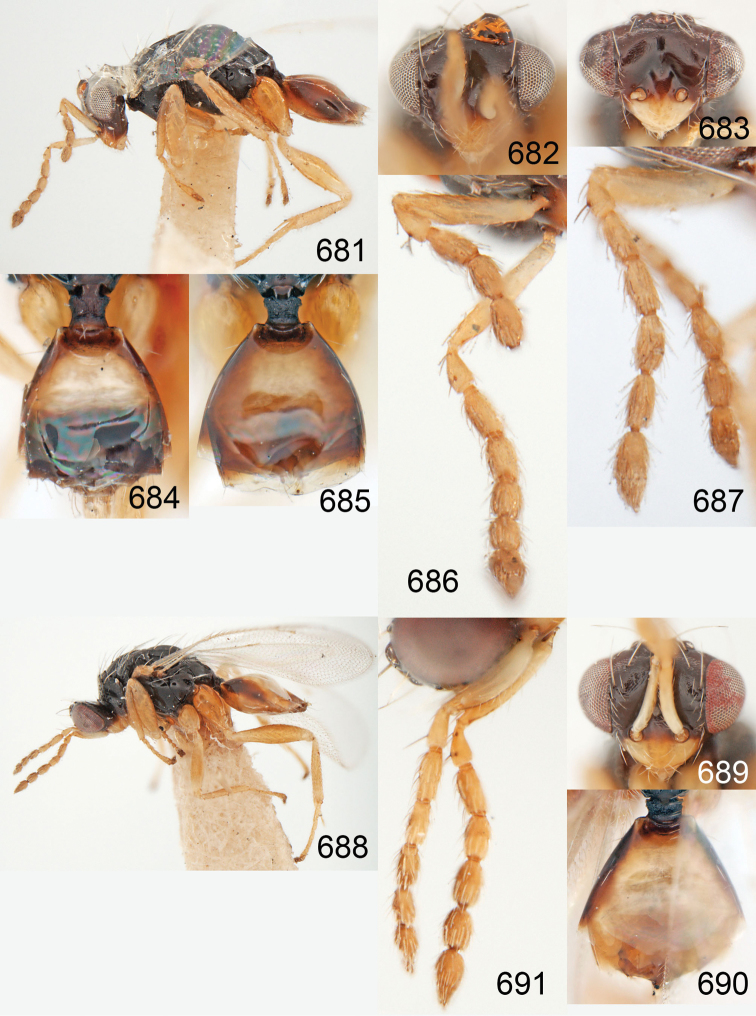
*Euplectrus* spp. **681–687** *Euplectrus comstockii* (neotype (female), non-type (male)): **681** habitus in lateral view, female **682** head in frontal view, female **683** head in frontal view, male **684** gaster in dorsal view, female **685** gaster in dorsal view, male **686** antenna in lateral view, female **687** antenna in lateral view, male **688–691** *Euplectrus junctus* (lectotype), female: **688** habitus in lateral view **689** head in frontal view **690** gaster in dorsal view **691** antenna in lateral view.

Lower face medially yellowish-brown (female, Fig. 682) or white (male, Fig. 683), pale area reaching slightly outside of level of outer lateral margin of toruli, part between pale area and eyes black (female) or dark brown (male); scutellum smooth in anteromedian ⅔, remaining parts with very weak reticulation; legs yellowish-white, except yellowish-brown hind coxa (Fig. 681); petiole 0.7× as long as wide; gaster dark brown, anterior ½ with a round yellowish-brown spot (female, Fig. 684), or anterior ½ white with dark brown lateral margins (male, Fig. 685); male scape slightly expanded and widest apically, 3.3× as long as wide (Fig. 687), sensory pores confined to anteroventral ½.

##### Description.

*Female*. Length of body 1.9 mm. Antenna with scape and pedicel yellowish-brown, base of scape whitish, flagellomeres brown (Fig. 686). Mandibles and palpi yellowish-white. Head black and shiny, vertex dark reddish-brown almost black, lower face yellowish-brown medially, pale area reaching slightly outside of level of outer lateral margin of toruli, part between pale area and eyes black (Fig. 682). Frons close to eyes with two rows of setae. Vertex smooth. Occipital margin with a weak carina behind ocellar triangle.

Mesosoma black and shiny (Fig. 681). Each sidelobe of mesoscutum with 12 setae. Scutellum 1.0× as long as wide; smooth in anteromedian ⅔, remaining parts with very weak reticulation. Dorsellum anteriorly with a groove that is divided by longitudinal carinae, groove medially 0.4× as long as length of dorsellum. Propodeum smooth; anteromedially with a transverse semicircular cup; propodeal callus with nine setae. Legs yellowish-white, hind coxa yellowish-brown (Fig. 681). Both fore wings missing in examined female specimen.

Gaster dark brown, anterior ½ with a round yellowish-brown spot (Fig. 684).

Ratios. HE/MS/WM = 2.2/1.0/1.2; POL/OOL/POO = 5.0/2.7/1.0; OOL/DO = 1.5; WE/WF/WH/HH = 1.0/2.4/4.4/3.2; WH/WT = 1.0; TS1/TS2/LT/LT1/LT2/LT3/LT4 = 4.0/2.8/6.8/2.4/1.4/1.0/1.8; LP/WP = 0.7; MM/LG = 1.5.

*Male*. Length of body 1.6 mm. Scape slightly expanded and widest apically (Fig. 687), sensory pores confined to anteroventral ½. Wings transparent, veins yellowish-white and setae black; submarginal vein with four setae; costal cell with one row of setae on ventral surface, and margin with four setae close to marginal vein; with 13 admarginal setae, in one row. Otherwise similar to female except gaster with anterior ½ white with dark brown lateral margins and posterior ½ dark brown (Fig. 685), and shorter.

Ratios. LC/WS = 3.3; PM/ST = 1.4; MM/LG = 1.8.

##### Hosts and biology.

*Alabama argillacea* (Erebidae) (Howard 1880).

##### Distribution.

USA (Alabama) (Howard 1880).

#### Euplectrus edithae

Taxon classificationAnimaliaHymenopteraEulophidae

Schauff

Figures 692–698 , 703–705 , 799

Euplectrus edithae Schauff in Schauff & Janzen, 2001: 198–200. Holotype ♀ (INBio), not examined.

##### Material.

Type material: 1♀ paratype of *Euplectrus edithae* (BMNH). Additional material: 1♀ from Zurqui de Moravia in Costa Rica, i.e. the type locality (MIUCR); 4♀ 2♂ from COSTA RICA: Puntarenas, Monteverde, no date, ex Lepidoptera (possibly Notodontidae) on Lycopodium (BMNH, CNC, INBio, USNM); 1♀ from Alajuela, N. slope Volcan Cacao (MZLU).

##### Diagnosis.

**Figures 692–702.**
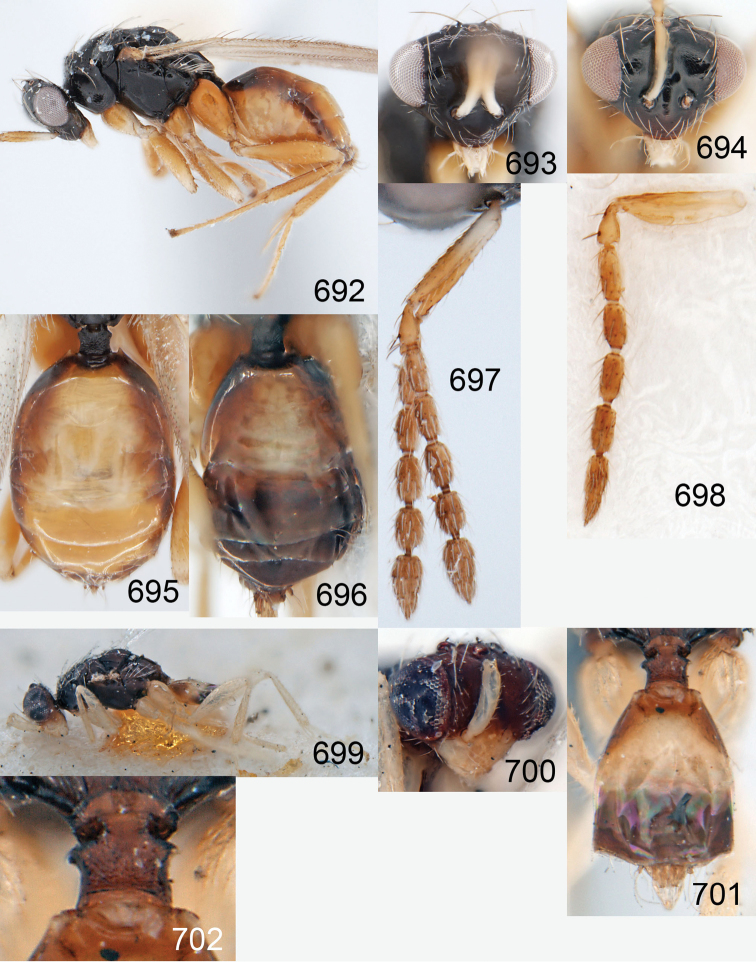
*Euplectrus* spp. **692–698** *Euplectrus edithae* (non-types): **692** habitus in lateral view, female **693** head in frontal view, female **694** head in frontal view, male **695** gaster in dorsal view, female **696** gaster in dorsal view, male **697** antenna in lateral view, female **698** antenna in lateral view, male **699–702** *Euplectrus leucotrophis* (lectotype), male: **699** habitus in lateral view **700** head in frontal view **701** gaster in dorsal view **702** petiole in dorsal view.

Lower face completely black (Figs 693, 694); legs yellowish-brown (Fig. 692); petiole 0.8× as long as wide; gaster in female yellowish-brown with dark brown lateral margins (Fig. 695), in male with anterior ½ yellowish-brown in median ⅓ and dark brown laterally, posterior ½ dark brown (Fig. 696); male scape narrow and widest in apical part, 4.1× as long as wide (Fig. 698), sensory area confined to apicoventral ½ and with same colour as remaining scape (yellowish-white).

##### Description.

**Figures 703–708.**
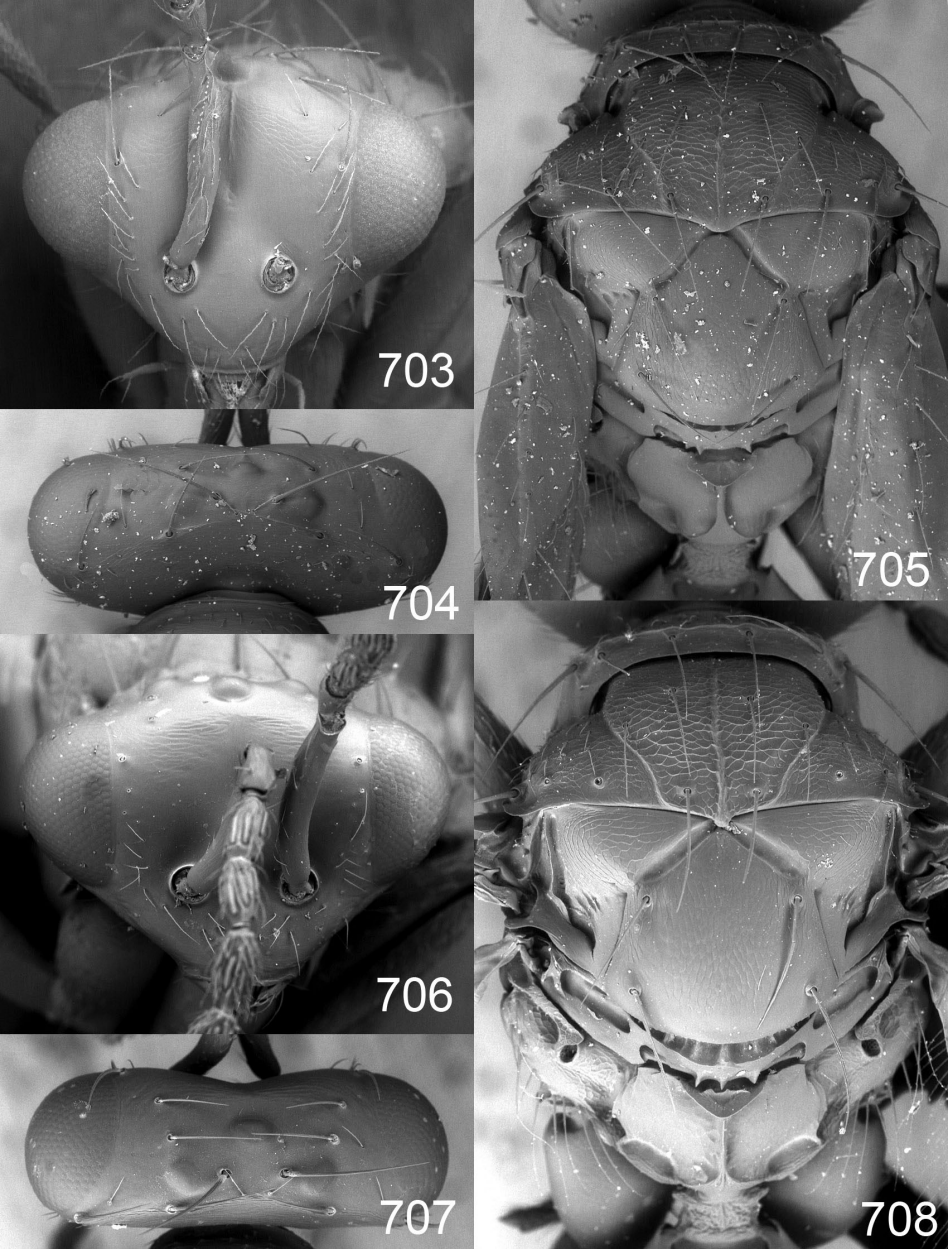
*Euplectrus* spp., females. **703–705** *Euplectrus edithae* (non-types, no codes): **703** head in frontal view **704** vertex **705** mesosoma in dorsal view **706–708** *Euplectrus puttleri* (paratypes, no codes): **706** head in frontal view **707** vertex **708** mesosoma in dorsal view.

*Female*. Length of body 2.4 mm. Antenna with scape yellowish-brown with base yellowish-white, pedicel yellowish-brown, flagellomeres pale brown (Fig. 697). Mandibles and palpi yellowish-white. Head including lower face black and shiny (Fig. 693). Frons close to eyes with three rows of setae (Fig. 703). Vertex with very weak reticulation inside ocellar triangle, smooth outside triangle (Fig. 704). Occipital margin with a weak carina behind ocellar triangle (Fig. 704).

Mesosoma dark brown and shiny (Fig. 692). Each sidelobe of mesoscutum with nine setae. Scutellum 1.0× as long as wide; with weak engraved reticulation, posterior margin smooth (Fig. 705). Dorsellum anteriorly with a narrow groove that is divided by longitudinal carinae (Fig. 799), groove medially 0.3× as long as length of dorsellum. Propodeum smooth (Fig. 799); anteromedially with a transverse semicircular cup; propodeal callus with 12 setae. Legs yellowish-brown (Fig. 692). Fore wing: costal cell with two rows of setae on ventral surface, and margin with four setae close to marginal vein; with 12 admarginal setae, in one row.

Gaster yellowish-brown with dark brown lateral margins (Fig. 695).

Ratios. HE/MS/WM = 1.7/1.3/1.0; POL/OOL/POO = 21.0/16.0/1.0; OOL/DO = 2.3; WE/WF/WH/HH = 1.0/2.8/4.6/3.3; WH/WT = 1.1; PM/ST = 1.3; TS1/TS2/LT/LT1/LT2/LT3/LT4 = 4.2/2.6/7.6/3.2/1.4/1.0/1.6; LP/WP = 0.8; MM/LG = 1.0.

*Male*. Length of body 2.0 mm. Scape narrow and widest in apical part (Fig. 698), sensory area confined to apicoventral ½ and with same colour as remaining scape (yellowish-white). Gaster in anterior ½ yellowish-brown in median ⅓ and dark brown laterally, posterior ½ dark brown (Fig. 696). Otherwise similar to female.

Ratio. LC/WS = 4.1.

##### Hosts and biology.

From a caterpillar feeding on *Lycopodium* (Lycopodiaceae) (**new record**).

##### Distribution.

Costa Rica (Puntarenas and San José Provinces).

#### Euplectrus frontalis

Taxon classificationAnimaliaHymenopteraEulophidae

Howard

Figures 716–719

Euplectrus frontalis Howard, 1885: 27. Holotype ♀ (USNM), examined.

##### Material.

Type material: ♀ holotype of *Euplectrus frontalis* (USNM). Additional material: 1♂ (USNM).

##### Diagnosis.

Lower face in male (female head missing) yellowish-brown with a narrow dark stripe between pale area and eye margin, dark stripe about as wide as ½ the diameter of one torulus, and with part between antennal scrobes reddish-brown (Fig. 717); gaster in female yellowish-brown with dark brown lateral margins and with a round dark brown spot posteromedially (Fig. 718), in male with anterior ½ white with dark brown lateral margins and posterior ½ dark brown; legs yellowish-white (Fig. 716); dorsellum with a narrow groove along anterior margin, medially reaching ⅓ of dorsellum length; petiole 0.5× as long as wide.

##### Description.

*Female*. Length of body 2.5 mm (estimated as the female examined lacks head).

Head missing in holotype female.

Mesosoma black and shiny (Fig. 716). Each sidelobe of mesoscutum with 12 setae. Scutellum 1.0× as long as wide; with engraved rather strong reticulation, meshes slightly elongate, posterior margin smooth. Dorsellum anteriorly with a narrow groove, medially 0.3× as long as length of dorsellum, part behind groove with very weak reticulation. Propodeum with very weak reticulation; anteromedially with a very short and wide cup, 0.3× as long as wide; propodeal callus with 14 setae. Legs yellowish-white (Fig. 716). Wings missing holotype female.

Gaster yellowish-brown with dark brown lateral margins and with a round dark brown spot posteromedially (Fig. 718).

Ratios. TS1/TS2/LT/LT1/LT2/LT3/LT4 = 2.7/1.5/5.7/1.3/1.2/1.0/1.7; LP/WP = 0.5; MM/LG = 1.3.

*Male*. Length of body 2.1 mm. Antenna with scape yellowish-white, expanded apically, sensory area with same colour as remaining scape; pedicel and basal three flagellomeres yellowish-brown, apical three flagellomeres missing in examined male (Fig. 719). Mandibles and palpi yellowish-white. Head dark reddish-brown, lower face yellowish-brown with a narrow dark stripe between pale area and eye margin, dark stripe about as wide as ½ the diameter of one torulus, and with part between antennal scrobes reddish-brown (Fig. 717). Frons close to eyes with two irregular rows of setae. Vertex with very weak reticulation inside ocellar triangle, outside ocellar triangle smooth. Occipital margin rounded.

Fore wing: costal cell with two irregular and sparse rows of setae on ventral surface, and margin with five setae close to marginal vein; with 17 admarginal setae, in one row.

Gaster with anterior ½ white with dark brown lateral margins and posterior ½ dark brown.

Otherwise similar to female.

Ratios. HE/MS/WM = 2.9/1.0/1.8; POL/OOL/POO = 6.3/3.0/1.0; OOL/DO = 0.8; WE/WF/WH/HH = 1.0/2.1/4.3/3.0; LC/WS = 3.6; WH/WT = 1.1; PM/ST = 1.4; MM/LG = 1.3.

##### Hosts and biology.

Noctuidae indet. on black walnut (*Juglans nigra*) (Howard 1885).

##### Distribution.

USA (Virginia) (Howard 1885).

##### Remarks.

The original description was based on a single female from an unknown noctuid larva collected on black walnut in September 13, 1882, Arlington Virginia. In the USNM there are two specimens, a female and a male, under this name. Both are labeled “No. 2841, Sept-13-82”, “Type No. 2657, U.S.N.M.”. Howard does not mention any male in his description, but as the male mentioned above has the same labels as the holotype female these two specimens are very likely from the same series and are conspecific. The female lacks the head and wings, the male lacks left flagellum and apical 3 flagellomeres on the right antenna, left fore leg with only coxa and left mid leg with coxa+femur remaining, left hind leg with apical 3 tarsomeres missing.

As the female lacks the head, features of the male head has been used to include the female in the key. Female and male heads are similar in some features, but the placement of the female in the key must be considered provisional until a female head is found.

#### Euplectrus furnius

Taxon classificationAnimaliaHymenopteraEulophidae

Walker

Euplectrus furnius Walker, 1843: 48. Lectotype ♀ (BMNH), examined.

##### Material.

Type material: ♀ lectotype of *Euplectrus furnius* (BMNH).

##### Diagnosis.

Lower face black; antennal flagellum short, e.g. flagellomeres 3–4 1.1× as long as wide; scutellum with very weak reticulation, almost smooth; legs yellowish-brown; petiole 0.9× as long as wide; gaster with anterior ½ yellowish-brown with dark brown lateral margins, posterior ½ dark brown.

##### Description.

*Female*. Length of body 2.0 mm. Antenna with scape, pedicel and flagellomeres 1–2 yellowish-brown, flagellomeres 3–6 pale brown. Mandibles and palpi yellowish-brown. Head including lower face black and shiny. Vertex smooth and shiny. Occipital margin with a weak carina behind ocellar triangle.

Mesosoma black and shiny. Scutellum convex, 1.0× as long as wide, with very weak engraved reticulation, posterior and lateral margins smooth. Dorsellum anteriorly with a groove that is divided by longitudinal carinae, groove medially 0.3× as long as length of dorsellum. Propodeum smooth; anteromedially with a transverse semicircular cup that has posterior part strongly raised; propodeal callus with eight setae. Legs yellowish-brown. Fore wing: costal cell on margin with two setae close to marginal vein.

Gaster with anterior ½ yellowish-brown with dark brown lateral margins, posterior ½ dark brown.

Ratios. HE/MS/WM = 1.3/1.0/nm; POL/OOL/POO = 4.0/3.0/nm; OOL/DO = nm; WE/WF/WH/HH = 1.0/6.3/7.3/5.7; WH/WT = 1.2; PM/ST = 1.3; TS1/TS2/LT/LT1/LT2/LT3/LT4 = 4.0/2.0/7.6/2.8/1.4/1.0/2.0; LP/WP = 0.9; MM/LG = 1.5.

*Male*. Unknown.

##### Hosts and biology.

Unknown.

##### Distribution.

The West Indies (St Vincent) (Walker 1843).

##### Remarks.

Lectotype specimen with left flagellum missing. See also remarks under *Euplectrus insularis*.

#### Euplectrus insularis

Taxon classificationAnimaliaHymenopteraEulophidae

(Howard)

Pachyscapha insularis Howard, 1897: 159. Lectotype ♂ (BMNH), examined.
Euplectrus insularis (Howard), Peck (1951: 453).

##### Material.

Type material: ♂ lectotype of *Pachyscapha insularis* (BMNH).

##### Diagnosis.

Very similar to the male of *Euplectrus alvarowillei*, i.e. with a black and strongly swollen scape (Fig. 59), pedicel and flagellomeres 1–2 yellowish-white and flagellomere 3 dark brown. Differs in having lower face medially reddish-brown (black in *Euplectrus alvarowillei*); petiole transverse (0.8× as long as wide) (1.0× as long as wide in *Euplectrus alvarowillei*); hind legs with coxae and femora yellowish-brown (darker in *Euplectrus alvarowillei*).

##### Description.

*Male*. Length of body 1.6 mm. Antenna with scape black, strongly swollen, not possible to measure as both scapes have collapsed; pedicel and flagellomeres 1–2 yellowish-white, flagellomere 3 black with peduncle yellowish-white (flagellomeres 4-6 missing). Mandibles and palpi yellowish-brown. Head black and shiny, lower face medially reddish-brown. Vertex smooth and shiny. Occipital margin rounded.

Mesosoma black and shiny. Mesoscutum with sidelobes with several setae. Scutellum convex; 1.1× as long as wide; with very weak reticulation, posterior and lateral margins smooth. Dorsellum along anterior margin with a very narrow groove. Propodeum smooth and shiny; anteromedially with a triangular cup; propodeal callus with seven setae. Legs yellowish-brown.

Gaster with anterior ½ yellowish-white with lateral margins dark brown, posterior ½ dark brown.

Ratios. HE/MS/WM = 1.4/1.3/1.0; POL/OOL/POO = 1.7/1.0/nm; OOL/DO = nm; WE/WF/WH/HH = 1.0/2.8/4.6/3.0; WH/WT = 1.2; PM/ST = nm; TS1/TS2/LT/LT1/LT2/LT3/LT4 = 4.0/2.0/7.2/2.6/1.4/1.0/1.8; LP/WP = 0.8; MM/LG = 1.4.

*Female*. Unknown.

##### Hosts and biology.

Unknown.

##### Distribution.

The West Indies (Grenada) (Howard 1897).

##### Remarks.

Bouček (1977) synonymized *Euplectrus insularis* with *Euplectrus furnius*. *Euplectrus insularis* is known only from males and *Euplectrus furnius* only from females. The biology is not known for either species. Until the host is known for either and the sexes can be associated it is best to keep these two species separate. Lectotype specimen of *Euplectrus insularis* with flagellomeres 4-6 and right fore wing missing.

#### Euplectrus junctus

Taxon classificationAnimaliaHymenopteraEulophidae

Gahan

Figures 688–691

Euplectrus junctus Gahan, 1927: 30–31. Lectotype ♀ (USNM), designated here, examined.

##### Material.

Type material: ♀ lectotype of *Euplectrus junctus* (USNM).

##### Diagnosis.

Lower face medially yellowish-white with median part yellowish-brown, pale area extending slightly outside of outer lateral margins of toruli (Fig. 689); legs yellowish-brown with hind coxa slightly darker (Fig. 688); fore wing submarginal vein with six setae; petiole 0.5× as long as wide; gaster yellowish-brown, in anterior ⅔ with lateral margins dark brown, and with a darker spot in posteromedian part (Fig. 690).

##### Description.

*Female*. Length of body 2.1 mm. Antenna with scape and pedicel yellowish-brown, flagellomeres pale brown with ventral part of flagellomeres 1–3 yellowish-brown (Fig. 691). Mandibles and palpi yellowish-white. Head black with parts lateral to ocellar triangle dark reddish-brown and shiny, lower face medially yellowish-white with median part yellowish-brown, pale area extending slightly outside outer lateral margins of toruli, parts between pale area and eyes black (Fig. 689). Frons close to eyes with two rows of setae. Vertex smooth lateral to ocellar triangle, with very weak reticulation inside ocellar triangle. Occipital margin with a carina.

Mesosoma black and shiny (Fig. 688). Each sidelobe of mesoscutum with 12 setae. Scutellum 1.0× as long as wide; with weak and engraved reticulation, posterior margin smooth. Dorsellum anteriorly with a groove that is divided by longitudinal carinae. Propodeum smooth; anteromedially with a semicircular cup; propodeal callus with five visible setae, but some appears broken off, so probably with more than five setae. Legs yellowish-brown with hind coxa slightly darker (Fig. 688). Fore wing: costal cell on ventral surface with two rows of setae, and margin with two setae close to marginal vein; with 13 admarginal setae, in one row.

Gaster yellowish-brown with margins in anterior ⅔ dark brown and with a darker spot in posteromedian part (Fig. 690).

Ratios. HE/MS/WM = 2.1/1.0/1.4; POL/OOL/POO = 6.0/2.8/1.0; OOL/DO = 1.2; WE/WF/WH/HH = 1.0/2.4/4.3/3.3; WH/WT = 1.1; PM/ST = 1.4; TS1/TS2/LT/LT1/LT2/LT3/LT4 = 3.6/2.2/6.2/1.8/1.0/1.2/2.2; LP/WP = 0.5; MM/LG = 1.4.

*Male*. Unknown.

##### Hosts and biology.

*Isoparce cupressi* (Sphingidae) (Gahan 1927).

##### Distribution.

USA (Florida) (Gahan 1927).

##### Remarks.

This species was described from three females but no primary type was selected. One of the females labeled “ALACHUA CO., FLA, Waldo, x.13-1924, 253, T.H. Hubbell”, “Reared fron Isoparce cupressi”, “Type No. 29451 U.S.N.M.”, “Euplectrus junctus Gahan female Type”, is hereby selected as lectotype.

#### Euplectrus leucotrophis

Taxon classificationAnimaliaHymenopteraEulophidae

Howard

Figures 699–702

Euplectrus leucotrophis Howard, 1885: 26. Lectotype ♂ (USNM), designated here, examined.

##### Material.

Type material: 3♂ syntypes of *Euplectrus leucotrophis*, one of which is selected lectotype here (USNM).

##### Diagnosis.

Entire lower face white with median part yellowish-white (Fig. 700); scape slightly expanded and widest in apical part, 3.6× as long as wide (Fig. 700), sensory pores confined to apico-ventral part, sensory area with same colour as scape; legs white; petiole 0.6× as long as wide, with posterior margin strongly curved forwards (Fig. 702); gaster with anterior ½ yellowish-white with anterolateral margins dark brown, margin broken medially by white stripe, posterior ½ dark brown (Fig. 701).

##### Description.

*Male*. Length of body 1.3 mm. Antenna with scape white, pedicel yellowish-white, flagellomeres missing in type specimens; scape slightly expanded and widest in apical part (Fig. 700), sensory pores confined to apico-ventral part, sensory area with same colour as scape. Mandibles and palpi white. Head dark reddish-brown and shiny, entire lower face white with median part yellowish-white (Fig. 700). Frons close to eyes with a row of setae and with some setae parallel to this row. Vertex smooth. Occipital margin with a weak carina behind ocellar triangle.

Mesosoma dark reddish-brown (Fig. 699). Each sidelobe of mesoscutum with eight setae. Scutellum 1.1× as long as wide; with very weak engraved reticulation, with posterior margin smooth. Dorsellum anteriorly without a groove or foveae. Propodeum smooth; anteromedially with a transverse semicircular cup; propodeal callus with six setae. Legs white (Fig. 699). Fore wing: costal cell with two rows of setae on ventral surface, and margin with four setae close to marginal vein; with 12 admarginal setae, in one row.

Gaster with anterior ½ yellowish-white with anterolateral margins dark brown, margin broken medially by white stripe, posterior ½ dark brown (Fig. 701).

Ratios. HE/MS/WM = 2.5/1.0/1.5; POL/OOL/POO = 5.3/2.0/1.0; OOL/DO = 0.8; WE/WF/WH/HH = 1.0/2.4/4.7/3.6; LC/WS = 3.6; WH/WT = 1.3; PM/ST = 1.2; TS1/TS2/LT/LT1/LT2/LT3/LT4 = 2.7/1.5/5.0/1.4/1.2/1.0/1.3; LP/WP = 0.6; MM/LG = 1.7.

*Female*. Unknown.

##### Hosts and biology.

Recorded as “Arctiidae indet.” (Howard 1885), but highly unlikely to be the host. Arctiidae is nowadays classifed as a subfamily (Arctiinae) in Erebidae.

##### Distribution.

USA (Florida) (Howard 1885).

##### Remarks.

The syntype series consists of three males. One male is on a strongly corroded pin and this specimen lacks the head. Two males are glued to the same card, specimen to the left lacks entire left antenna and pedicel+flagellum on the right antenna, the right hindwing, hind legs, tarsus on right midleg; specimen to the right lacks the flagellum on the left antenna and pedicel+flagellum on the right antenna, tarsus on right fore and mid legs, and right wing-pair. The lectotype is the male to the right of the two males glue to the same card. Labels on the pin: “No.647.a. July 19.80 [1880]”, “Type No. 2654 U.S.N.M.”, “Euplectrus leuctrophis How. ms.”. All specimens have a pale appearance which might be due to bleaching by light.

#### Euplectrus marginatus

Taxon classificationAnimaliaHymenopteraEulophidae

Ashmead

Figures 727–729

Euplectus[sic!] marginatus Ashmead, 1885: 18. Lectotype ♀ (USNM), designated here, examined.

##### Material.

Type material: ♀ lectotype of *Euplectus marginatus* (USNM).

##### Diagnosis.

Head including lower face black (Fig. 728); scutellum with strong engraved reticulation; dorsellum and propodeum with strong reticulation; legs yellowish-brown (Fig. 727); petiole 0.7× as long as wide; gaster dark brown with a yellowish-brown spot anteromedially (Fig. 729).

##### Description.

*Female*. Length of body 1.5 mm. Antenna with scape and pedicel yellowish-brown, (Fig. 728). Mandibles and palpi not visible. Head including lower face black and shiny (Fig. 728). Frons close to eyes with one row of setae. Vertex with weak reticulation. Occipital margin with a carina.

Mesosoma black and shiny (Fig. 727). Mesoscutum with number of setae on sidelobes difficult to see because they are mostly broken. Scutellum 1.0× as long as wide; with strong engraved reticulation. Dorsellum with a narrow groove along anterior margin. Propodeum strongly reticulate; anteromedially with a transverse semicircular cup; propodeal callus with six setae. Legs yellowish-brown (Fig. 727). Fore wing: setae on ventral side of costal cell and admarginal setae difficult to see; margin of costal cell with one seta close to marginal vein.

Gaster dark brown with a small yellowish-brown spot anteromedially (Fig. 729).

Ratios. HE/MS/WM = nm; POL/OOL/POO = 4.1/1.9/1.0; OOL/DO = 1.5; WE/WF/WH/HH = 1.0/3.6/6.0/4.5; WH/WT = 1.0; PM/ST = 1.3; TS1/TS2/LT/LT1/LT2/LT3/LT4 = 4.0/2.8/7.8/2.8/1.3/1.0/2.3; LP/WP = 0.7; MM/LG = 1.5.

*Male*. Not seen.

##### Hosts and biology.

Noctuidae indet. (Lepidoptera) (Ashmead 1885).

##### Distribution.

USA (Florida) (Ashmead 1885).

##### Remarks.

This species was described from 15 specimens, 13 females and 2 males, but no primary type was selected. One of the females is here designated as lectotype, with labels “Jacksonville, Fla”, “Type”, “Type No.27642, U.S.N.M.”, “Euplectrus marginatus Ashm.”, “Euplectrus marginatus Lectotype Gordh 1978”. This specimen was selected as lectotype by G. Gordh but was never validated. The redescription above is based on the lectotype, as are also the illustrations. The lectotype lacks entire right antenna and left antennal flagellum.

#### Euplectrus mellipes

Taxon classificationAnimaliaHymenopteraEulophidae

Provancher

Figures 460–463

Euplectrus mellipes Provancher, 1887: 207. Lectotype ♀ (CNC), designated by Gahan and Rohwer 1917: 399, examined.

##### Material.

Type material: ♀ lectotype of *Euplectrus mellipes* (CNC).

##### Diagnosis.

Lower face medially yellowish-brown, pale area reaching half-way between outer lateral margins of toruli and eyes (Fig. 461); scutellum reticulate with elongate meshes that converge toward the middle, anteromedian ½ of scutellum with weaker reticulation; legs yellowish-brown (Fig. 460); petiole 0.9× as long as wide; gaster with anterior ½ yellowish-brown with dark brown lateral margins, posterior ½ dark brown (Fig. 462).

##### Description.

*Female*. Length of body 2.4 mm. Antenna with scape and pedicel yellowish-brown, flagellomeres brown (Fig. 463). Mandibles and palpi yellowish-brown. Head black and shiny, lower face medially yellowish-brown, pale area reaching half-way between outer lateral margins of toruli and eyes, parts between pale area and eyes black (Fig. 461). Frons close to eyes with one row of setae. Vertex smooth. Occipital margin with a carina behind ocellar triangle.

Mesosoma black and shiny (Fig. 460). Each sidelobe of mesoscutum with 12 setae. Scutellum 1.0× as long as wide; reticulate with elongate meshes that converge toward the middle, anteromedian ½ of scutellum with weaker reticulation. Dorsellum with a very narrow groove along anterior margin, medially 0.1× as long as length of dorsellum. Propodeum smooth; anteromedially with a triangular cup; propodeal callus with 12 setae. Legs yellowish-brown (Fig. 460). Fore wing: costal cell with one row of setae on ventral surface, and margin with four setae close to marginal vein.

Gaster with anterior ½ yellowish-brown with dark brown lateral margins, posterior ½ dark brown (Fig. 462).

Ratios. HE/MS/WM = 2.0/1.0/1.2; POL/OOL/POO = 6.0/3.0/1.0; OOL/DO = 1.2; WE/WF/WH/HH = 1.0/2.4/4.6/3.6; WH/WT = 1.1; PM/ST = 1.4; TS1/TS2/LT/LT1/LT2/LT3/LT4 = 3.3/2.0/6.2/2.0/1.5/1.0/1.7; LP/WP = 0.9; MM/LG = 1.3.

*Male*. Unknown.

##### Hosts and biology.

Unknown.

##### Distribution.

Canada (Provancher 1887).

##### Remarks.

The antennae of the lectotype lack flagellum of right antenna and apical two flagellomeres on left antenna.

#### Euplectrus pachyscaphus

Taxon classificationAnimaliaHymenopteraEulophidae

Girault

Figures 448–452

Euplectrus pachyscapha Girault, 1917a: 2. Lectotype ♀ (USNM), designated here, examined.

##### Material.

Type material: ♀ lectotype of *Euplectrus pachyscapha* (USNM).

##### Diagnosis.

Lower face medially reddish-brown, pale area reaching to outer lateral margins of toruli (Fig. 449); head below level of eyes distinctly pointed (Fig. 449); female scape long with ⅕ reaching above level of vertex (Fig. 452); dorsellum anteriorly with a groove that covers ⅓ the length of dorsellum and is divided by longitudinal carinae; legs yellowish-brown with hind coxa slightly darker (Fig. 448); petiole 0.5× as long as wide; gaster yellowish-brown in median ⅓ with lateral margins dark brown, and with a dark brown round spot posteromedially (Fig. 450).

##### Description.

*Female*. Length of body 2.4 mm. Antenna with scape and pedicel yellowish-brown, flagellomeres pale brown (Fig. 451); scape long with ⅕ reaching above level of vertex (Fig. 452). Mandibles and palpi yellowish-white. Head black and shiny, lower face medially reddish-brown, pale area reaching to outer lateral margins of toruli, parts between pale area and eyes black (Fig. 449). Frons close to eyes with two rows of setae. Vertex inside ocellar triangle with very weak reticulation, outside triangle smooth. Occipital margin with a carina behind ocellar triangle.

Mesosoma black and shiny (Fig. 448). Each sidelobe of mesoscutum with 16 setae. Scutellum 1.0× as long as wide; with weak and engraved reticulation and with elongate meshes in anterior ⅓ and isodiametric in posterior ⅔, posterior margin smooth. Dorsellum with a groove along anterior margin that is divided by longitudinal carinae, medially 0.3× as long as length of dorsellum. Propodeum with weak reticulation; anteromedially with a triangular cup; propodeal callus with ten setae. Legs yellowish-brown with hind coxa slightly darker (Fig. 448). Fore wing: costal cell on ventral surface with two rows of setae, and margin with five setae close to marginal vein; with 17 admarginal setae, in one row.

Gaster yellowish-brown in median ⅓ with lateral margins dark brown, and with a dark brown round spot posteromedially (Fig. 450).

Ratios. HE/MS/WM = 1.7/1.0/1.1; POL/OOL/POO = 5.6/3.4/1.0; OOL/DO = 1.8; WE/WF/WH/HH = 1.0/3.0/5.0/3.9; WH/WT = 1.0; PM/ST = 1.5; TS1/TS2/LT/LT1/LT2/LT3/LT4 = 3.5/2.3/7.1/2.3/1.5/1.0/1.8; LP/WP = 0.5; MM/LG = 1.3.

*Male*. Not found.

##### Hosts and biology.

Unknown.

##### Distribution.

USA (Kansas) (Girault 1917a).

##### Remarks.

*Euplectrus pachyscaphus* was described from an unspecified number of female and male specimens. The only remaining specimen in USNM (M. Gates personal communication) is a female which is used for the redescription above. This specimen is labeled “Type 21159 U.S.N.M.” and a handwritten label “Euplectrus pachyscapha Girault ♀ type”, and is here designated lectotype.

#### Euplectrus puttleri

Taxon classificationAnimaliaHymenopteraEulophidae

Gordh

Figures 706–708 , 709–715 , 800

Euplectrus puttleri Gordh in Puttler et al., 1980: 28–29. Holotype ♀ (USNM), not found.

##### Material.

Type material: 10♀ 3♂ paratypes of *Euplectrus puttleri* (UCR).

##### Diagnosis.

**Figures 709–719.**
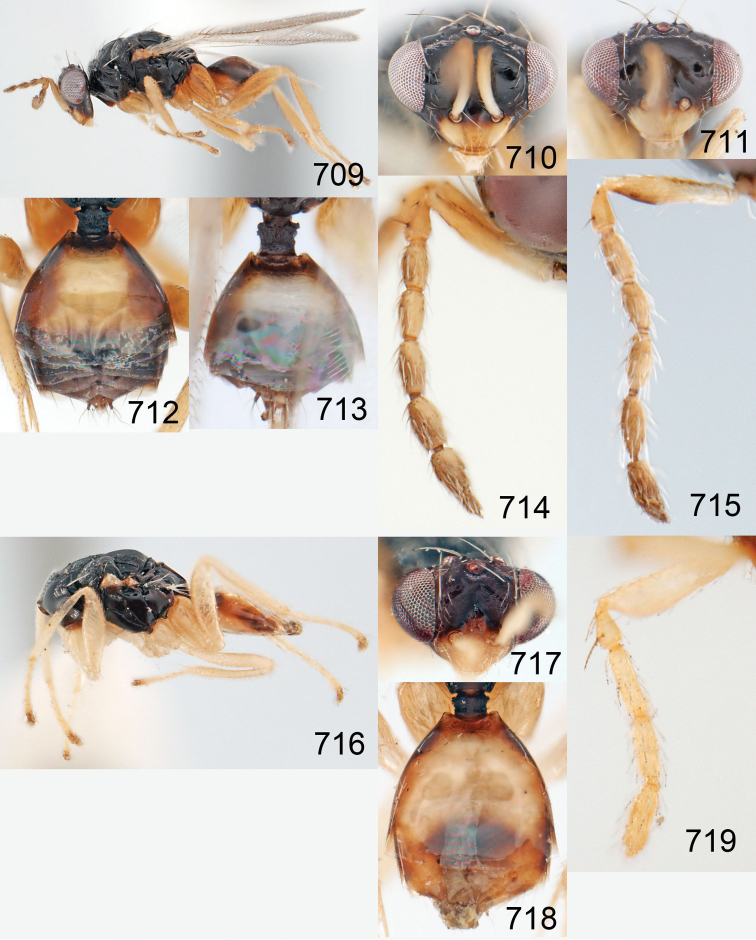
*Euplectrus* spp. **709–715** *Euplectrus puttleri* (paratypes): **709** habitus in lateral view, female **710** head in frontal view, female **711** head in frontal view, male **712** gaster in dorsal view, female **713** gaster in dorsal view, male **714** antenna in lateral view, female **715** antenna in lateral view, male; **716–719** *Euplectrus frontalis*: **716** habitus in lateral view, head missing, female holotype **717** head in frontal view, male non-type **718** gaster in dorsal view, female holotype **719** antenna in lateral view, male non-type.

Lower face medially yellowish-white with median ⅓ yellowish-brown, pale area reaching slightly outside of outer lateral margins of toruli (Figs 710, 711); dorsellum anteriorly with a groove that medially is 0.6× as long as dorsellum (Fig. 800); legs yellowish-brown with hind coxa slightly darker (Fig. 709); petiole 0.7× as long as wide in female, 0.8× in male; gaster with anterior ½ yellowish-brown (female, Fig. 712) or white (male, Fig. 713), with wide dark brown lateral margins, posterior ½ dark brown (Fig. 715).

##### Description.

*Female*. Length of body 2.3 mm. Antenna with scape and pedicel yellowish-brown, flagellomeres pale brown (Fig. 714). Mandibles and palpi yellowish-white. Head black and shiny, lower face medially yellowish-white with median ⅓ yellowish-brown, pale area extending slightly outside of outer lateral margins of toruli, parts between pale area and eyes black (Fig. 710). Frons close to eyes with two rows of setae (Fig. 706). Vertex smooth (Fig. 707). Occipital margin with a carina (Fig. 707).

Mesosoma black and shiny (Fig. 709). Each sidelobe of mesoscutum with 11 setae. Scutellum 1.0× as long as wide; with rather weak and engraved reticulation and with elongate meshes, posterior and lateral margins smooth (Fig. 708). Dorsellum anteriorly with a very wide groove, medially 0.6× as long as length of dorsellum, that is divided by longitudinal carinae (Fig. 800). Propodeum smooth (Fig. 800); anteromedially with a semicircular cup; propodeal callus with 9–12 setae. Legs yellowish-brown with hind coxa slightly darker (Fig. 709). Fore wing: costal cell on ventral surface with one row of setae, and margin with three setae close to marginal vein; with 13 admarginal setae, in one row.

Gaster with anterior ½ yellowish-brown with wide dark brown lateral margins, posterior ½ dark brown (Fig. 712).

Ratios. HE/MS/WM = 1.6/1.0/1.1; POL/OOL/POO = 9.5/5.5/1.0; OOL/DO = 1.7; WE/WF/WH/HH = 1.0/3.8/6.3/4.7; WH/WT = 1.1; PM/ST = 1.0; TS1/TS2/LT/LT1/LT2/LT3/LT4 = 3.8/2.4/6.0/1.8/1.4/1.0/1.6; LP/WP = 0.7; MM/LG = 1.0.

*Male*. Length of body 1.5 mm. Scape slightly expanded and widest above the middle and with base narrow (Fig. 715), sensory area dark brown and confined to apicoventral ⅓. Otherwise similar to female except petiole longer and pale parts of gaster white (Fig. 713).

Ratios. LC/WS = 4.4; LP/WP = 0.8; MM/LG = 1.0.

##### Hosts and biology.

*Anticarsia gemmatalis* (Erebidae) (Puttler et al. 1980).

##### Distribution.

Colombia, and introduced into the USA (Florida, Louisiana, Mississippi) (Puttler et al. 1980).

##### Remarks.

The holotype female of *Euplectrus puttleri* that according to the original description should be deposited in USNM could not be found there (M. Gates, personal communication).

#### Euplectrus rojasi

Taxon classificationAnimaliaHymenopteraEulophidae

Schauff

Euplectrus rojasi Schauff in Schauff & Janzen, 2001: 217–219. Holotype ♀ (USNM), not found.

##### Material.

The species was described from a single female that was deposited in USNM (Schauff and Janzen 2001). However, this holotype could not be located in the collections of USNM (M. Gates, personal communication), and therefore no material of this species has been examined.

##### Diagnosis.

In spite of the lack of material the original description gives valuable clues as to the identity of this species. Lower face is brown medially and dark yellow lateral to median dark brown area; hind coxae are black; posterior part of scutellum is overhanging dorsellum and hides anterior part of dorsellum; propodeum with anteromedian cup flattened, not raised as in the other species of *Euplectrus*; petiole is 1.5× as long as wide, i.e. very long for a *Euplectrus*.

##### Hosts and biology.

Unknown.

##### Distribution.

Costa Rica (Cartago Province) (Schauff and Janzen 2001).

#### Euplectrus semimarginatus

Taxon classificationAnimaliaHymenopteraEulophidae

Girault

Figures 477–478

Euplectrus semimarginatus Girault, 1917b: 1. Lectotype female in USNM, designated here, examined.

##### Material.

Type material: ♀ lectotype of *Euplectrus semimarginatus* (USNM).

##### Diagnosis.

See remarks below.

##### Description.

*Female*. Head missing in lectotype specimen. Length of body 1.9 mm (excluding head).

Mesosoma black and shiny (Fig. 477). Each sidelobe of mesoscutum with nine setae. Scutellum convex; 1.0× as long as wide; with very weak reticulation, hence shiny. Dorsellum hidden behind glue. Propodeum with very weak reticulation and shiny; anteromedian part hidden behind glue; propodeal callus with 15 setae. Legs yellowish-brown (Fig. 477). Wings transparent, veins and setae yellowish-white; setae on submarginal vein all broken off; admarginal setae and setae in costal cell not visible, hidden in glue.

Gaster yellowish-brown with anterolateral margins dark brown (Fig. 478).

Ratios. PM/ST = 1.2; TS1/TS2/LT/LT1/LT2/LT3/LT4 = 4.0/2.6/7.0/2.8/1.4/1.0/1.6; LP/WP = 0.5; MM/LG = 1.3.

*Male*. Unknown.

##### Hosts and biology.

*Aphis rumicis* (Hemiptera: Aphididae) (Girault 1917b). In view of other host records for species of *Euplectrus* this host record is very dubious and needs verification.

##### Distribution.

USA (Texas) (Girault 1917b).

##### Remarks.

The lectotype female lacks the head and is therefore not possible to include in the identification key. However, the gaster is with a very distinct pattern: yellowish-brown with dark brown margins in anterior ⅓ (Fig. 478), much like in *Euplectrus bobwhartoni* and *Euplectrus charlesporteri*. *Euplectrus semimarginatus* also has very weak reticulation on the scutellum, which thus is shiny, as in *Euplectrus bobwhartoni* (Fig. 69) but unlike *Euplectrus charlesporteri* which has stronger reticulation on the scutellum (Fig. 86). *Euplectrus semimarginatus* differs from *Euplectrus bobwhartoni* in having hindleg with tarsomere 1 1.7× as long as tarsomere 4, in *Euplectrus bobwhartoni* tarsomere 1 and 4 are equally long. Also similar to *Euplectrus floryae* and *Euplectrus jesusugaldei* but both these species have the dark margins on female gaster extending along more or less the entire gaster.

In the original description there is no mention on what material the description is based. Therefore a female that agrees with the original description, in USNM, and labeled: “College Stn Sept Banks”, “Texas”, “Type 21431”, “Euplectrus semimarginatus Girault female type” is designated as lectotype here.

#### Euplectrus solitarius

Taxon classificationAnimaliaHymenopteraEulophidae

Ashmead

Figures 486–488

Euplectrus solitarius Ashmead, 1904: 517. Holotype ♀ (USNM), examined.

##### Material.

Type material: ♀ holotype of *Euplectrus solitarius* (USNM).

##### Diagnosis.

Lower face medially reddish-brown, pale area reaching to level of outer lateral margins of toruli (Fig. 487); legs yellowish-brown (Fig. 486); scutellum with very weak engraved reticulation, almost smooth medially and with posterior ⅕ smooth; dorsellum anteriorly with a narrow groove that medially reaches ⅕ of length of dorsellum.

##### Description.

*Female*. Length of body 1.3 mm (excluding gaster which is missing in holotype). Antenna with scape yellowish-white, pedicel yellowish-brown (Fig. 488). Mandibles and palpi yellowish-brown. Head black and shiny, lower face medially reddish-brown, pale area reaching to level of outer lateral margins of toruli, parts between pale area and eyes black (Fig. 487). Frons close to eyes with three rows of setae. Vertex smooth. Occipital margin rounded.

Mesosoma black and shiny (Fig. 486). Each sidelobe of mesoscutum with 14 setae. Scutellum 1.1× as long as wide; with very weak engraved reticulation, almost smooth medially and with posterior ⅕ smooth. Dorsellum anteriorly with a narrow groove, medially about 0.2× as long as length of dorsellum. Propodeum smooth; anteromedially with a triangular cup that has posterior part strongly raised; propodeal callus with seven setae. Legs yellowish-brown (Fig. 486). Fore wing: costal cell with two rows of setae on ventral surface, and margin with five setae close to marginal vein; with 15 admarginal setae, in one row.

Ratios. HE/MS/WM = 1.8/1.1/1.0; POL/OOL/POO = 4.2/2.8/1.0; OOL/DO = 1.6; WE/WF/WH/HH = 1.0/2.4/4.3/3.3; WH/WT = 1.1; PM/ST = 1.6; TS1/TS2/LT/LT1/LT2/LT3/LT4 = 3.4/2.0/5.8/1.8/1.2/1.0/1.4.

*Male*. Unknown.

##### Hosts and biology.

Unknown.

##### Distribution.

Brazil (Ashmead 1904).

##### Remarks.

The holotype specimen lacks flagellum on both antennae, femora, tibiae and tarsi on fore legs, entire hind left leg, and petiole+gaster. The missing body parts make it difficult to fix the identity of this species and it is therefore not possible to include in the key.

#### Euplectrus valverdei

Taxon classificationAnimaliaHymenopteraEulophidae

Schauff

Figures 720–726 , 730–732 , 801

Euplectrus valverdei Schauff in Schauff & Janzen, 2001: 220–224. Holotype ♀ (INBio), not examined.

##### Material.

Type material: 1♀ 1♂ paratypes (BMNH). Additional material: COSTA RICA: 2♀ from Alajuela Province in malaise traps (INBio).

##### Diagnosis.

**Figures 720–729.**
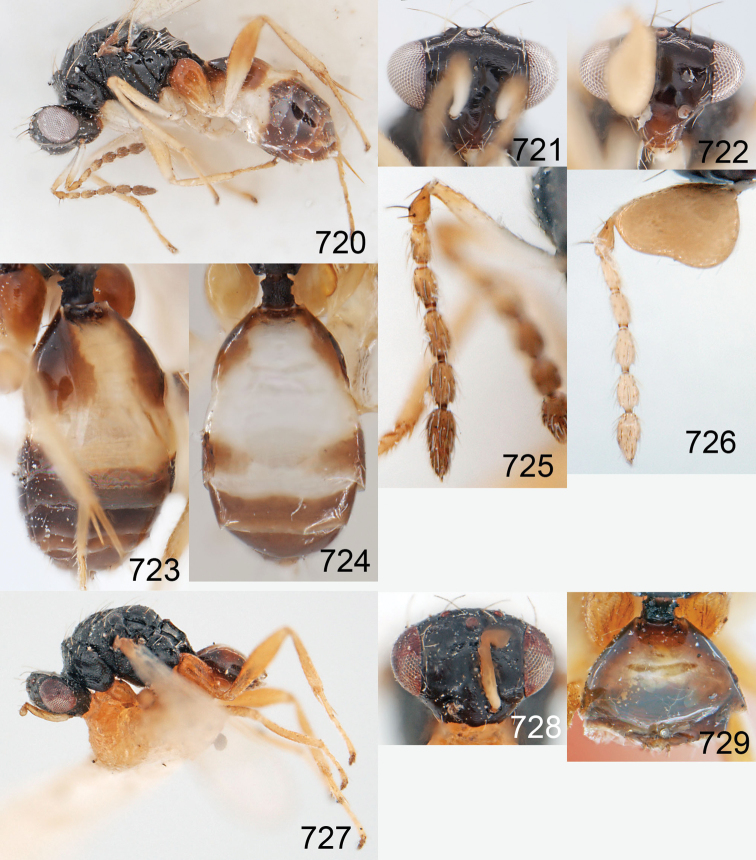
*Euplectrus* spp. **720–726** *Euplectrus valverdei* (paratypes): **720** habitus in lateral view, female **721** head in frontal view, female **722** head in frontal view, male **723** gaster in dorsal view, female **724** gaster in dorsal view, male **725** antenna in lateral view, female **726** antenna in lateral view, male; **727–729** *Euplectrus marginatus* (lectotype), female: **727** habitus in lateral view **728** head in frontal view **729** gaster in dorsal view.

Lower face medially dark reddish-brown to black and not clearly delimited from surrounding black areas (Fig. 721); mandibles dark brown; female with fore and midlegs with coxae yellowish-white, femora, tibiae and tarsi yellowish-brown; hind coxa pale brown, femur with basal ½ yellowish-white and apical ½ pale brown, tibia and tarsus yellowish-brown (Fig. 720), male legs as in female but with hind coxa yellowish-brown; petiole 0.8× as long as wide; female gaster dark brown with a large T-shaped yellowish-white spot in anterior ½ and with apex reddish-brown (Fig. 723), male gaster with anterior ½ white with dark brown lateral margins and posterior ½ dark brown (Fig. 724); male scape strongly expanded and widest close to base (Fig. 726), 1.4× as long as wide, sensory pores cover entire inner lateral surface.

##### Description.

**Figures 730–732.**
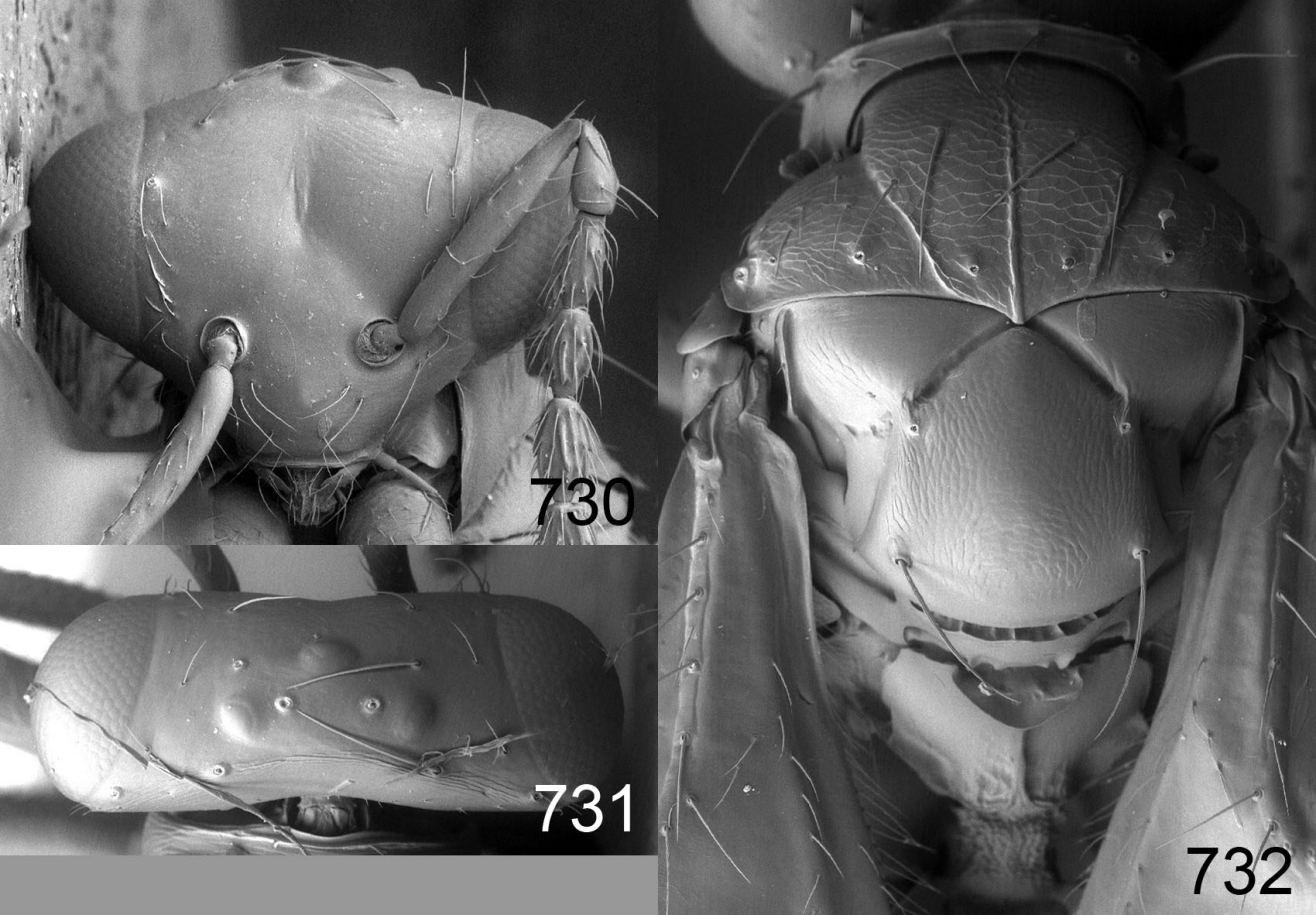
*Euplectrus valverdei*, female (non-types): **730** head in frontal view **731** vertex **732** mesosoma in dorsal view.

**Figures 733–738.**
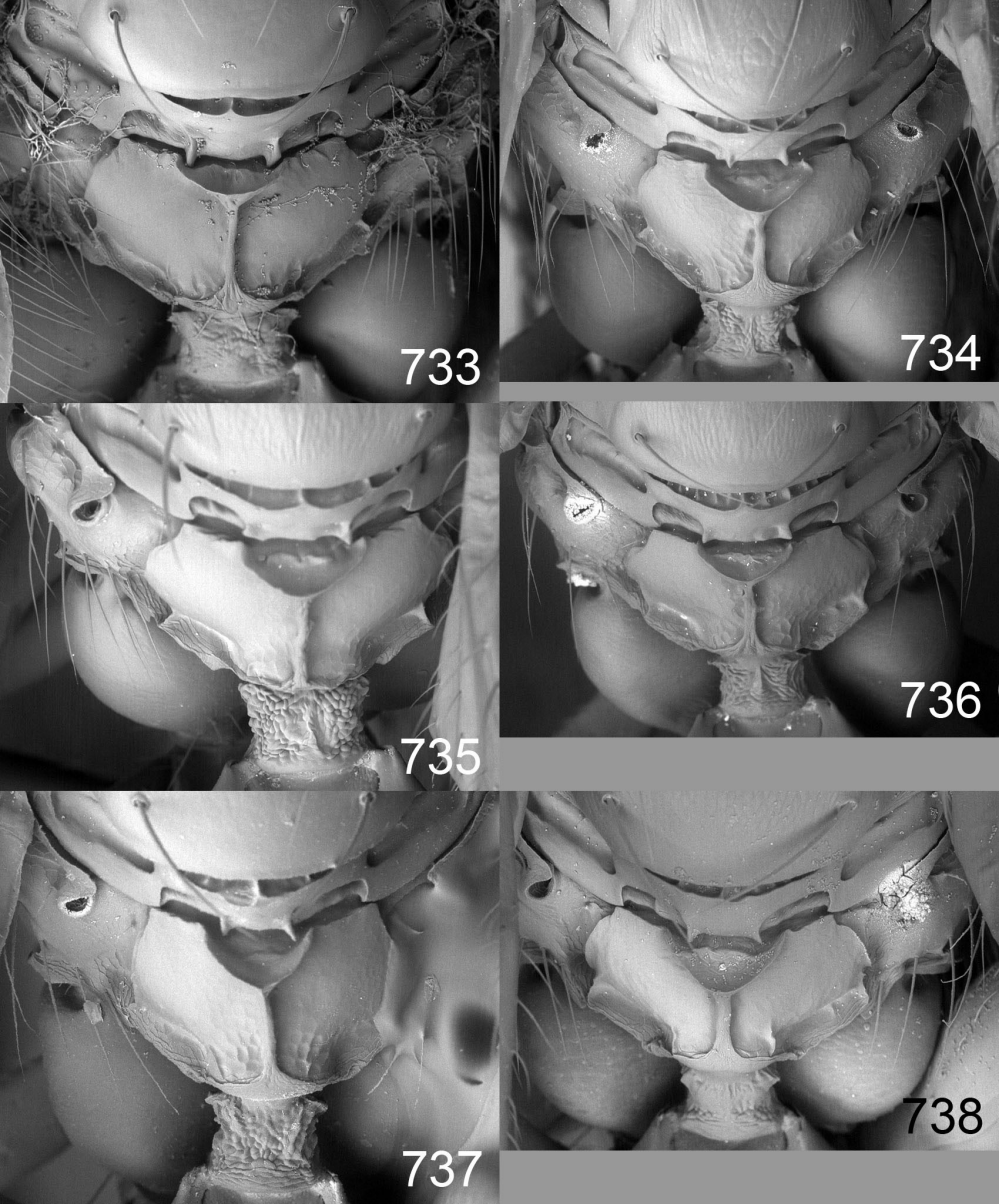
*Euplectrus* spp., propodeum, females: **733** *Euplectrus alejandrovalerioi* (DHJPAR0053946) **734** *Euplectrus alexsmithi* (DHJPAR0028872) **735** *Euplectrus alvarowillei* (DHJPAR0028881) **736** *Euplectrus andybennetti* (94-SRNP-5588, no barcode) **737** *Euplectrus andydeansi* (DHJPAR0046907) **738** *Euplectrus annettewalkerae* (DHJPAR0046917).

**Figures 739–744.**
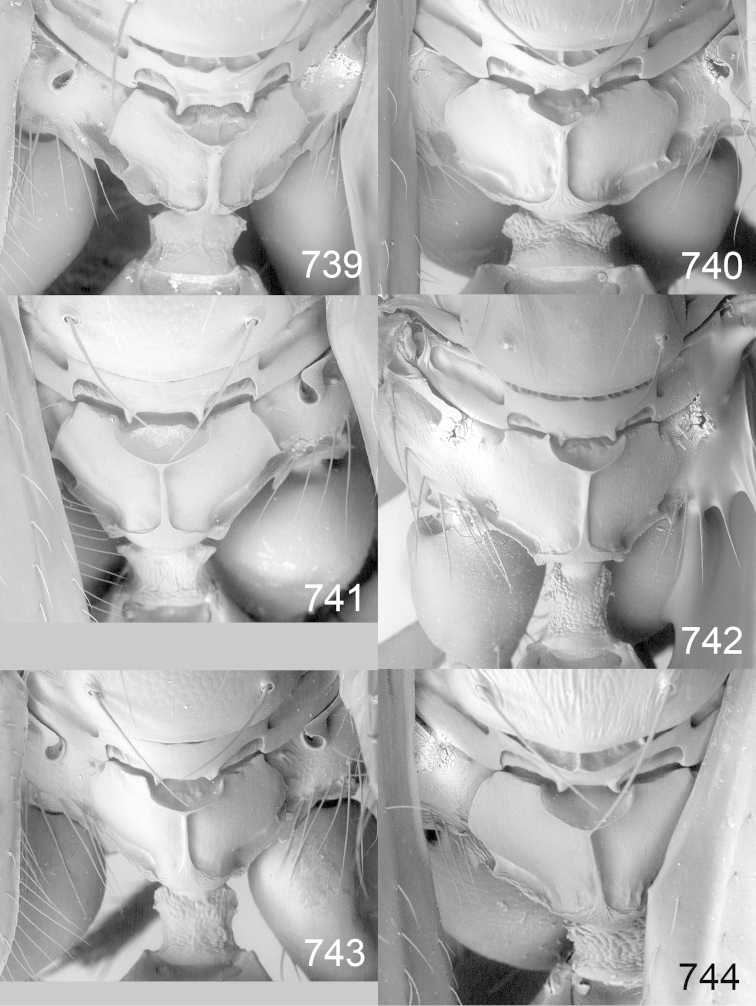
*Euplectrus* spp., propodeum, females: **739** *Euplectrus billbrowni* (DHJPAR0028838) **740** *Euplectrus bobwhartoni* (DHJPAR0028861) **741** *Euplectrus carlosarmientoi* (DHJPAR0023273) **742** *Euplectrus carlrettenmeyeri* (DHJPAR0046912) **743** *Euplectrus charlesmicheneri* (DHJPAR0031152) **744** *Euplectrus charlesporteri* (97-SRNP-9717, no barcode).

**Figures 745–750.**
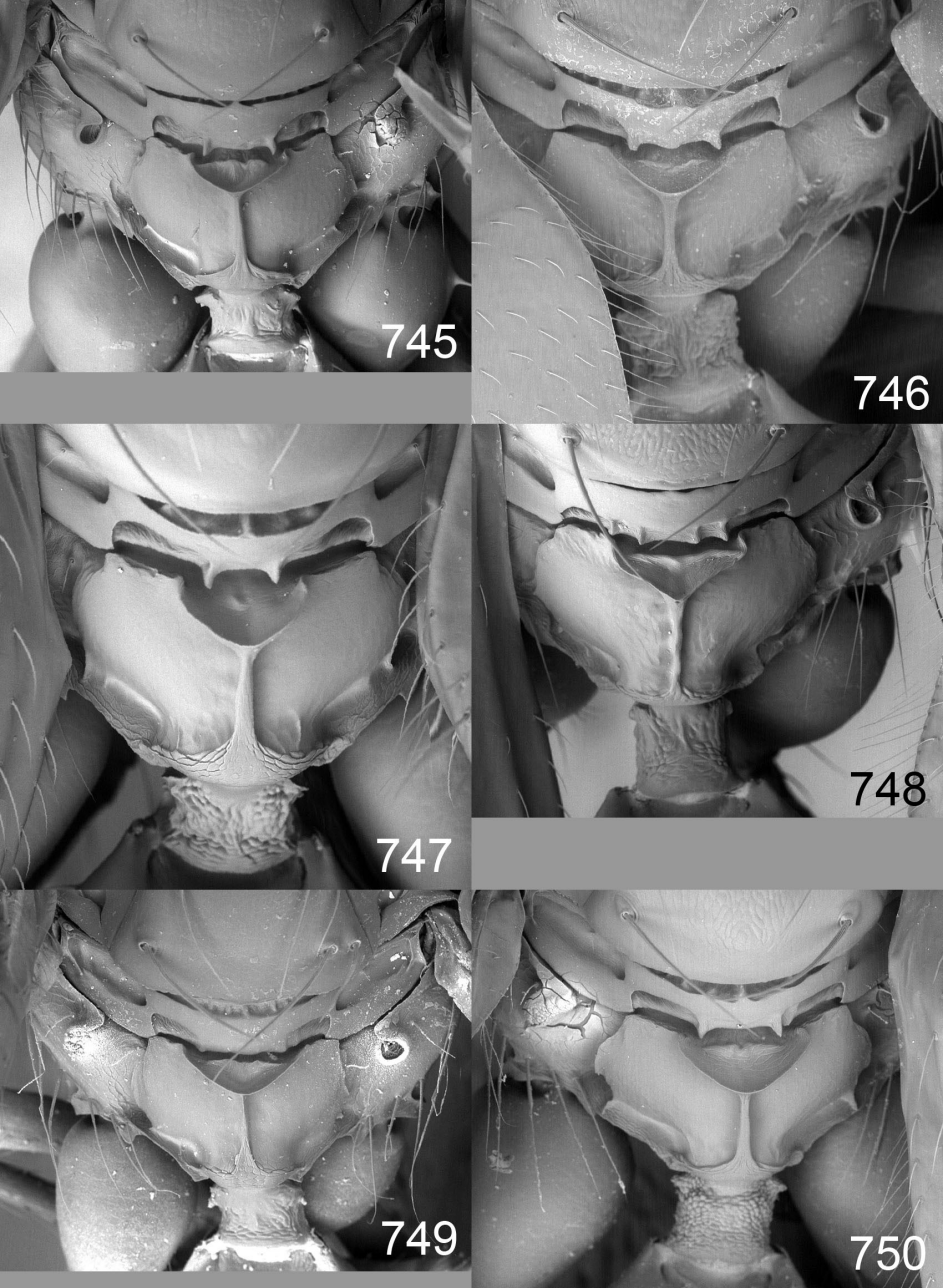
*Euplectrus* spp., propodeum, females: **745** *Euplectrus chrisdarlingi* (DHJPAR0028698) **746** *Euplectrus chrisgrinteri* (95-SRNP-3415, no barcode) **747** *Euplectrus corriemoreauae* (DHJPAR0053131) **748** *Euplectrus daveroubiki* (DHJPAR0023280) **749** *Euplectrus davesmithi* (DHJPAR0028811) **750** *Euplectrus davidwahli* (DHJPAR0042122).

**Figures 751–756.**
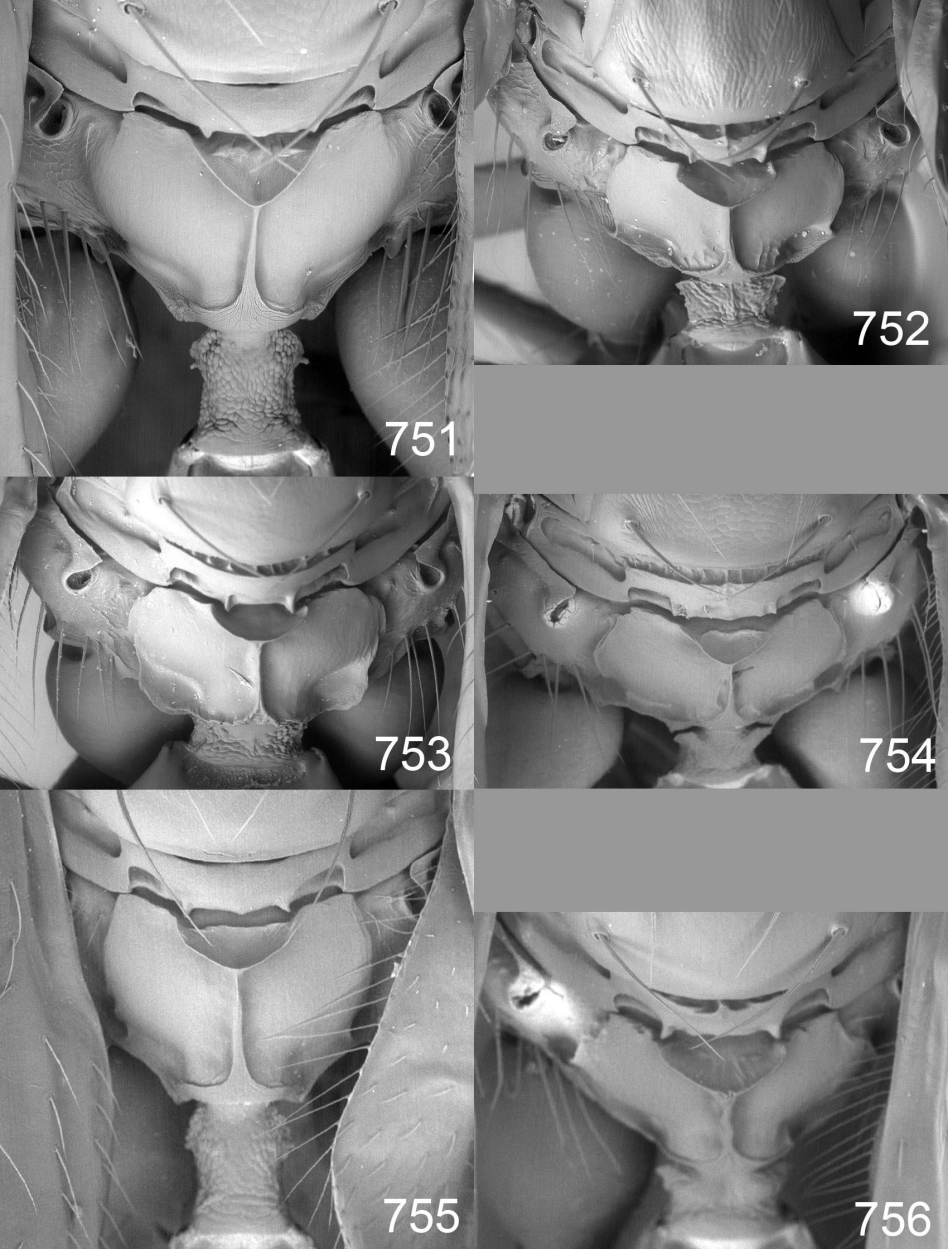
*Euplectrus* spp., propodeum, females: **751** *Euplectrus dianariasae* (DHJPAR0046906) **752** *Euplectrus donquickei* (DHJPAR0028814) **753** *Euplectrus eowilsoni* (DHJPAR0028932) **754** *Euplectrus garygibsoni* (DHJPAR0042124) **755** *Euplectrus gavinbroadi* (DHJPAR0045450) **756** *Euplectrus gerarddelvarei* (DHJPAR0042126).

**Figures 757–762.**
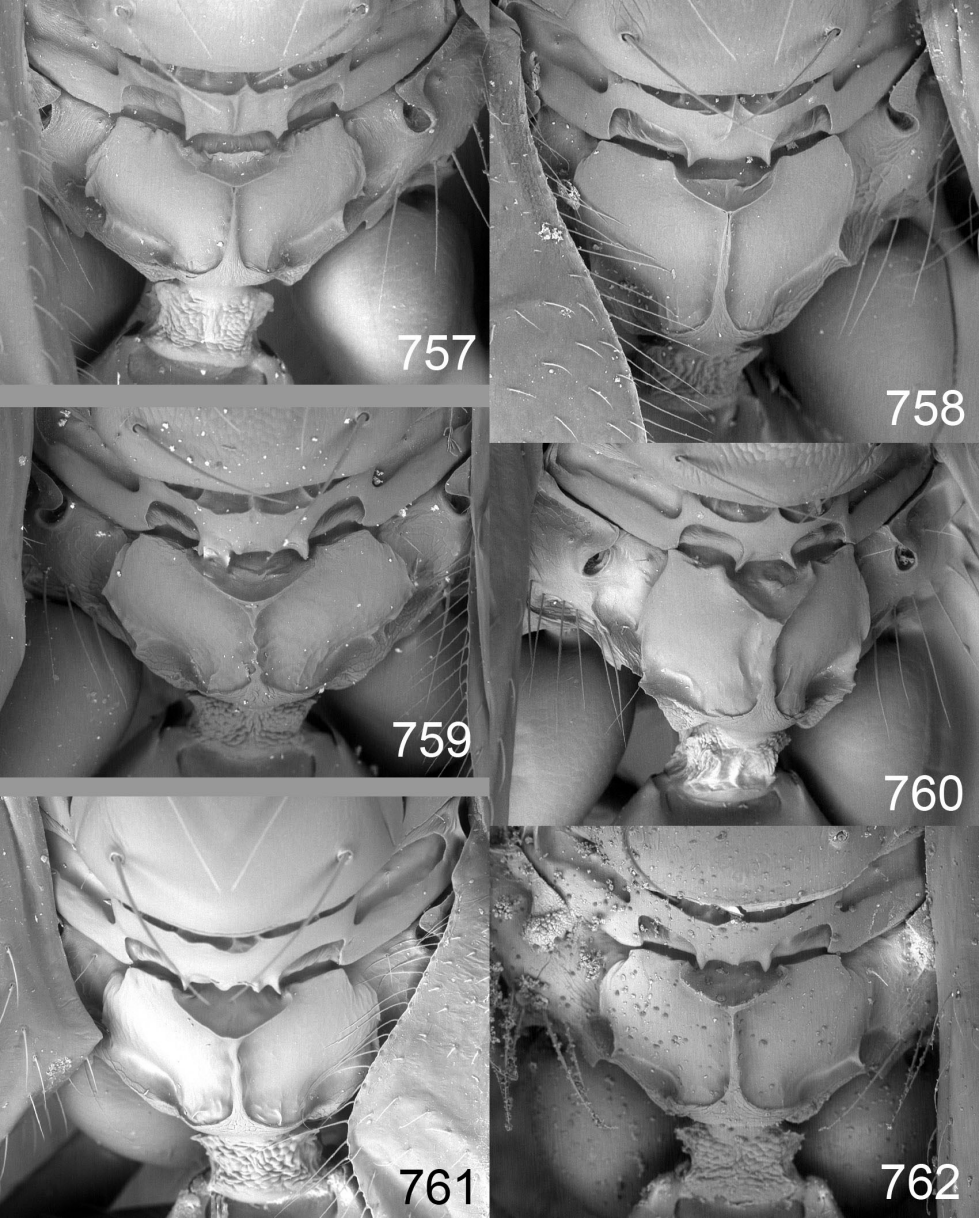
*Euplectrus* spp., propodeum, females (except 758 = male): **757** *Euplectrus henrytownesi* (DHJPAR0028805) **758** *Euplectrus howelldalyi* (DHJPAR0028882) **759** *Euplectrus hugokonsi* (90-SRNP-2035, no barcode) **760** *Euplectrus iangauldi* (DHJPAR0028769) **761** *Euplectrus jacklonginoi* (DHJPAR0038559) **762** *Euplectrus jesusugaldei* (97-SRNP-9370, no barcode).

**Figures 763–768.**
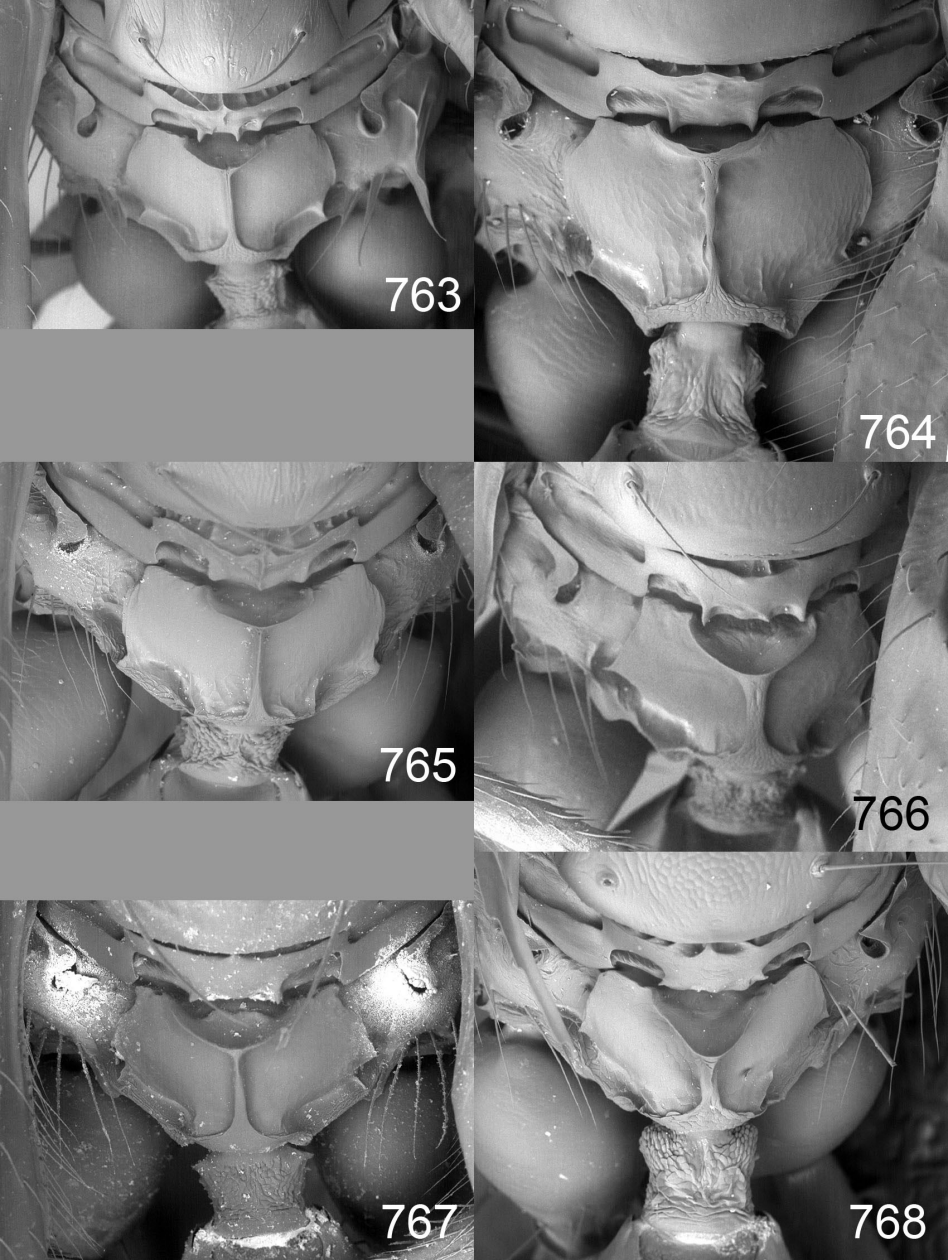
*Euplectrus* spp., propodeum, females: **763** *Euplectrus jimwhitfieldi* (DHJPAR0042135) **764** *Euplectrus jjrodriguezae* (94-SRNP-7129, no barcode) **765** *Euplectrus johnheratyi* (DHJPAR0028817) **766** *Euplectrus johnlasallei* (DHJPAR0028927) **767** *Euplectrus johnnoyesi* (DHJPAR0028699) **768** *Euplectrus josefernandezi* (DHJPAR0028908).

**Figures 769–774.**
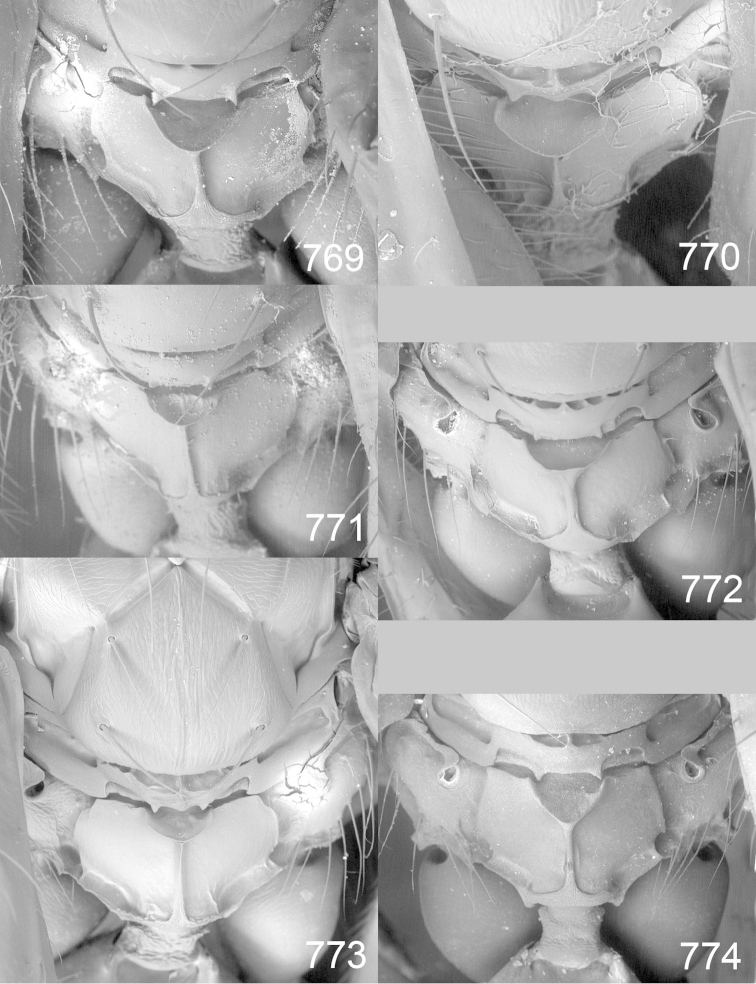
*Euplectrus* spp., propodeum, females: **769** *Euplectrus lubomirmasneri* (DHJPAR0028760) **770** *Euplectrus markshawi* (DHJPAR0028896) **771** *Euplectrus mikegatesi* (DHJPAR0031184) **772** *Euplectrus mikeschauffi* (DHJPAR0045452) **773** *Euplectrus mikesharkeyi* (DHJPAR0030516) **774** *Euplectrus ninazitaniae* (DHJPAR0028735).

**Figures 775–780.**
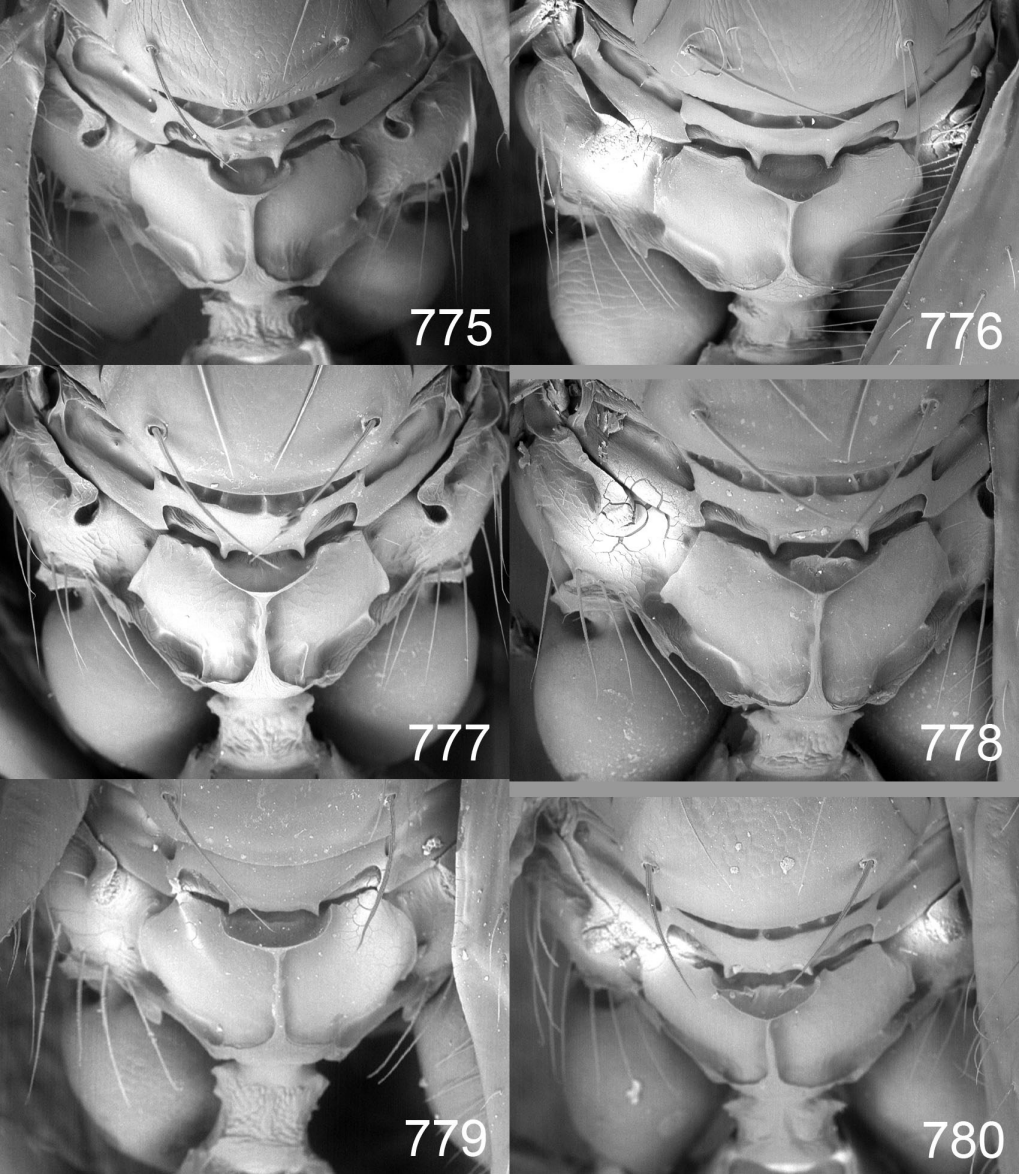
*Euplectrus* spp., propodeum, females: **775** *Euplectrus pammitchellae* (DHJPAR0038580) **776** *Euplectrus paulhansoni* (DHJPAR0028775) **777** *Euplectrus paulheberti* (DHJPAR0053133) **778** *Euplectrus paulhurdi* (DHJPAR0028878) **779** *Euplectrus philwardi* (DHJPAR0028876) **780** *Euplectrus robbinthorpi* (DHJPAR0023276).

**Figures 781–786.**
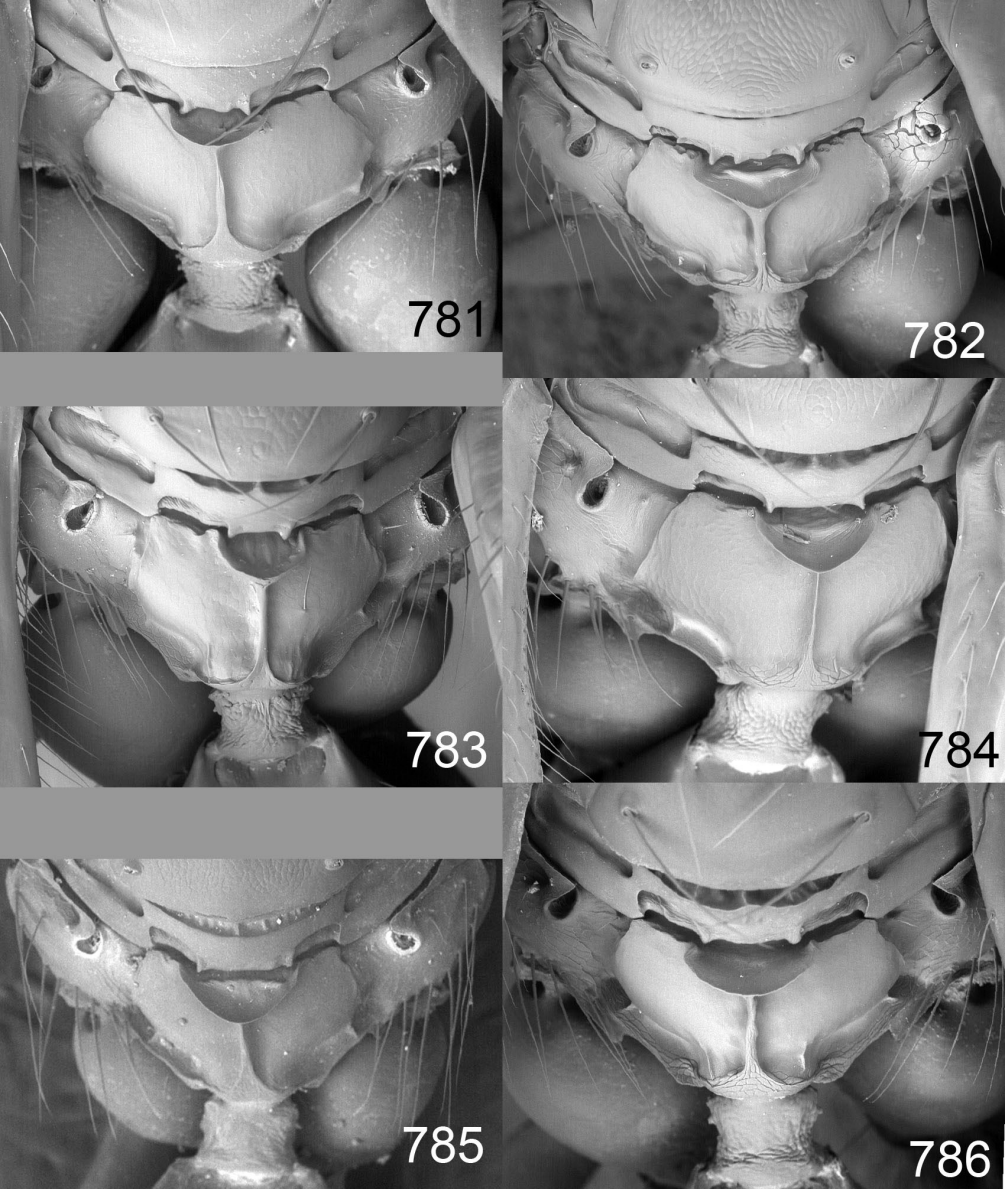
*Euplectrus* spp., propodeum, females: **781** *Euplectrus ronaldzunigai* (DHJPAR0028824) **782** *Euplectrus roysnellingi* (DHJPAR0028972) **783** *Euplectrus scottshawi* (DHJPAR0028877) **784** *Euplectrus sondrawardae* (DHJPAR0028850) **785** *Euplectrus victoriapookae* (DHJPAR0028836) **786** *Euplectrus wonyoungchoi* (DHJPAR0048229).

**Figures 787–792.**
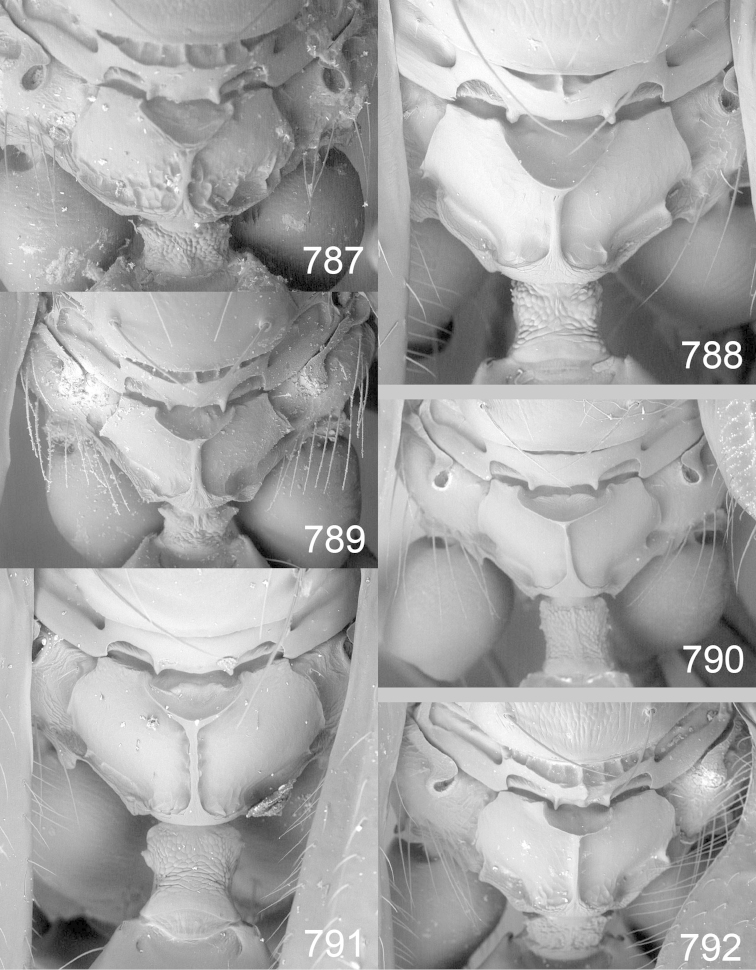
*Euplectrus* spp., propodeum, females: **787** *Euplectrus anae* (92-SRNP-747, no barcode) **788** *Euplectrus carlowae* (paratype) **789** *Euplectrus floryae* (non-type) **790** *Euplectrus hansoni* (non-type, no code) **791** *Euplectrus ireneae* (95-SRNP-6049, no barcode) **792** *Euplectrus ivonae* (no code).

**Figures 793–798.**
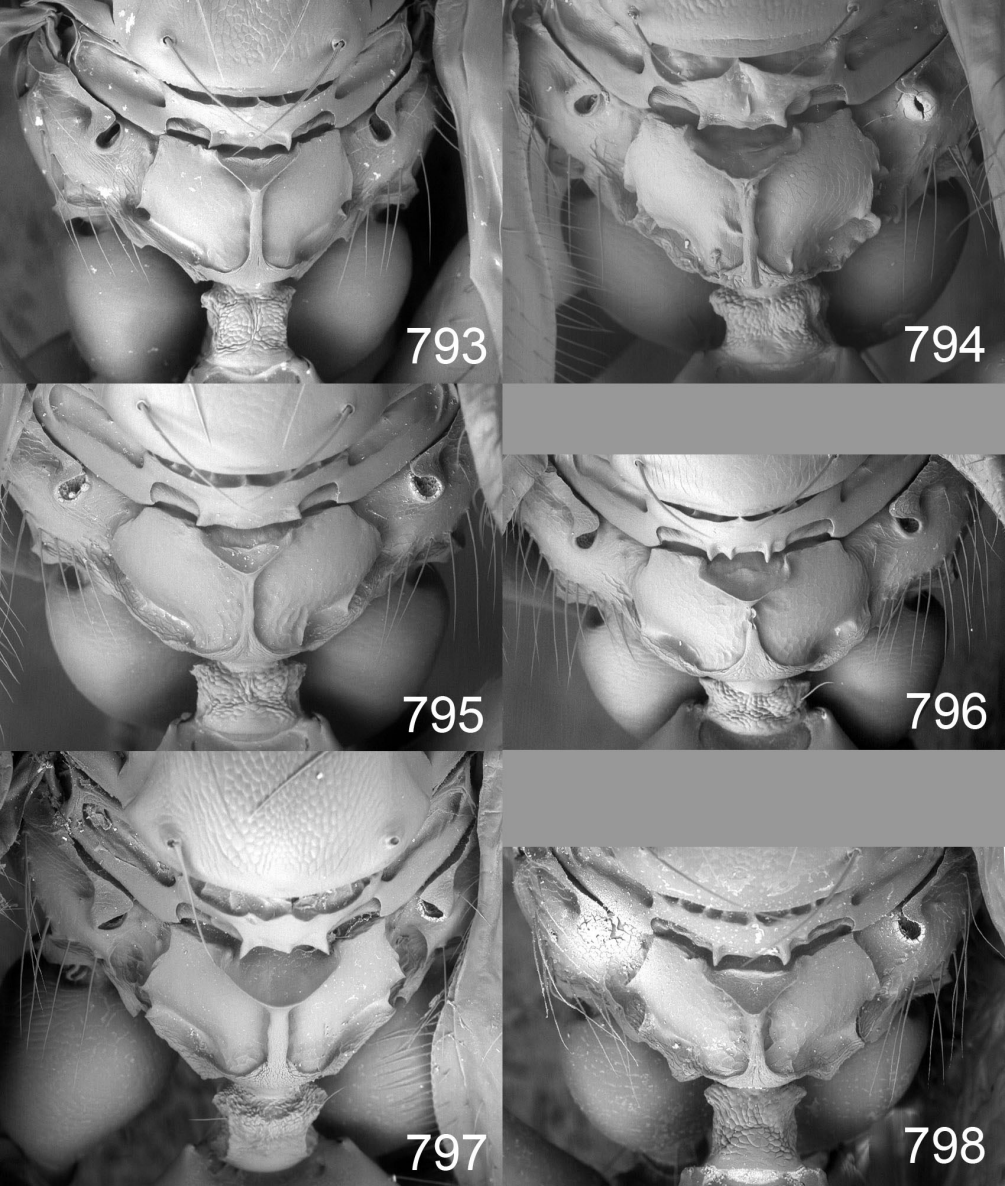
*Euplectrus* spp., propodeum, females: **793** *Euplectrus josei* (non-type) **794** *Euplectrus magdae* (non-type) **795** *Euplectrus mariae* (non-type) **796** *Euplectrus platyhypenae* (non-type) **797** *Euplectrus testaceipes* (non-type) **798** *Euplectrus xiomarae* (non-type).

**Figures 799–802.**
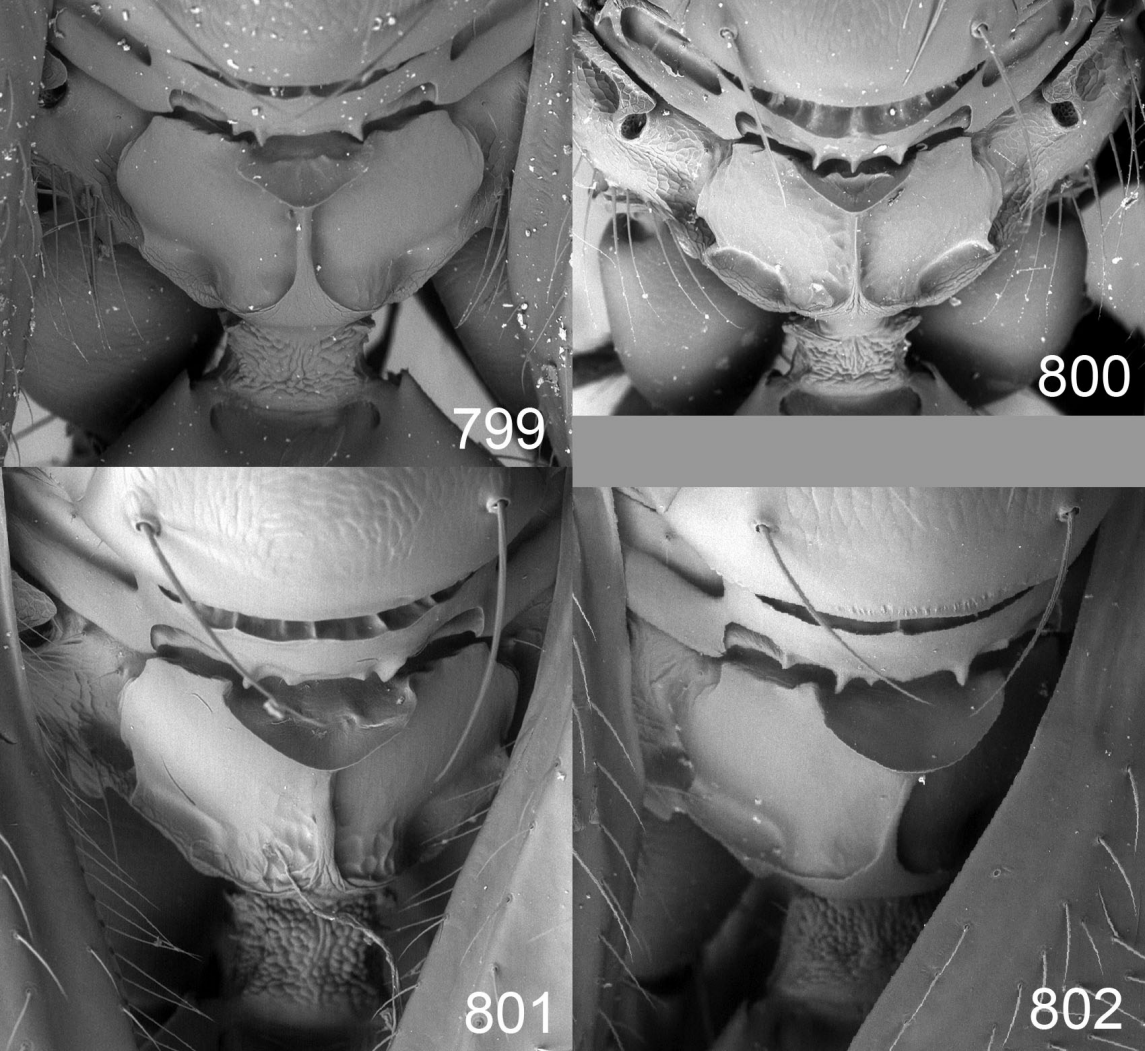
*Euplectrus* spp., propodeum, females: **799** *Euplectrus edithae* (non-type) **800** *Euplectrus puttleri* (paratype) **801** *Euplectrus valverdei* (non-type) **802** *Euplectrus zamorai* (non-type).

*Female*. Length of body 2.6 mm. Antenna with scape white with apex yellowish-white, pedicel, flagellomere 1 and base of 2 yellowish-brown, apical part of flagellomere 2 and entire 3–6 dark brown (Fig. 725). Mandibles dark brown, palpi white. Head black and shiny, lower face below toruli very dark reddish-brown to black and not clearly delimited from surrounding black areas (Fig. 721). Frons close to eyes with two rows of setae (Fig. 730). Vertex smooth (Fig. 731). Occipital margin rounded (Fig. 731).

Mesosoma black and shiny (Fig. 720). Each sidelobe of mesoscutum with 10 setae. Scutellum 1.2× as long as wide; with weak engraved reticulation (Fig. 731). Dorsellum anteriorly with a narrow groove (Fig. 801), medially 0.3× as long as length of dorsellum. Propodeum smooth (Fig. 801); anteromedially with a transverse semicircular cup; propodeal callus with 11 setae. Legs (Fig. 720): fore and mid legs with coxae yellowish-white, femora, tibiae and tarsi yellowish-brown; hind coxa pale brown, femur with basal ½ yellowish-white and apical ½ pale brown, tibia and tarsus yellowish-brown. Fore wing: costal cell on ventral surface with one row of setae, and margin with five setae close to marginal vein; with 12 admarginal setae, in one row.

Gaster dark brown with a large T-shaped yellowish-white spot in anterior ½ and with apex reddish-brown (Fig. 723).

Ratios. HE/MS/WM = 1.7/1.1/1.0; POL/OOL/POO = 6.9/4.6/1.0; OOL/DO = 1.5; WE/WF/WH/HH = 1.0/2.8/4.7/3.2; WH/WT = 1.2; PM/ST = 1.5; TS1/TS2/LT/LT1/LT2/LT3/LT4 = 4.5/2.4/7.5/2.9/1.7/1.0/1.7; LP/WP = 0.8; MM/LG = 1.0.

*Male*. Length of body 1.8 mm. Scape strongly expanded and widest close to base, sensory pores cover entire inner lateral surface (Fig. 726). Otherwise similar to female except scape yellowish-brown, flagellomeres 1–4 yellowish-white, 5–6 pale brown (Fig. 726); hind coxa yellowish-brown; gaster with anterior ½ white with dark brown lateral margins and posterior ½ dark brown (Fig. 724).

Ratios. LC/WS = 1.4; MM/LG = 1.0.

##### Hosts and biology.

Unknown.

##### Distribution.

Costa Rica (Alajuela and San José Provinces).

#### Euplectrus zamorai

Taxon classificationAnimaliaHymenopteraEulophidae

Schauff

Figures 502–505 , 509–511 , 802

Euplectrus zamorai Schauff in Schauff & Janzen, 2001: 227–228. Holotype ♀ (USNM), examined.

##### Material.

Type material: holotype female of *Euplectrus zamorai* (USNM). Additional material: COSTA RICA: 2♀ from Guanacaste and Heredia provinces in malaise traps (BMNH, INBio).

##### Diagnosis.

Lower face black (Fig. 503); antenna with scape, pedicel and flagellomeres 1–3 yellowish-white, flagellomeres 4–6 dark brown (Fig. 505); mandibles and palpi dark brown; fore and midlegs with coxae yellowish-white, femora, tibiae and tarsi yellowish-brown; hind coxa dark brown, femur with basal ½ yellowish-white and apical ½ pale brown, tibia and tarsus yellowish-brown (Fig. 502); petiole 0.9× as long as wide; gaster with anterior ½ yellowish-white with dark brown lateral margins, posterior ½ dark brown (Fig. 504).

##### Description.

*Female*. Length of body 2.2 mm. Antenna with scape, pedicel and flagellomeres 1–3 yellowish-white, 4–6 dark brown (Fig. 505). Mandibles and palpi dark brown. Head including lower face black and shiny (Fig. 503). Frons close to eyes with one row of setae (Fig. 509). Vertex smooth (Fig. 510). Occipital margin with a carina behind ocellar triangle (Fig. 510).

Mesosoma black and shiny (Fig. 502). Each sidelobe of mesoscutum with four setae. Scutellum 0.9× as long as wide; with very weak engraved reticulation, posterior margin smooth (Fig. 511). Dorsellum anteriorly with a narrow groove (Fig. 802), medially 0.2× as long as length of dorsellum. Propodeum smooth (Fig. 802); anteromedially with a semicircular cup that has posterior part strongly raised; propodeal callus with seven setae. Legs (Fig. 502): fore and mid legs with coxae yellowish-white, femora, tibiae and tarsi yellowish-brown; hind coxa dark brown, femur with basal ½ yellowish-white and apical ½ pale brown, tibia and tarsus yellowish-brown. Fore wing: costal cell with two rows of setae on ventral surface, and margin with three setae close to marginal vein; with 12 admarginal setae, in one row.

Gaster with anterior ½ yellowish-white with dark brown lateral margins, posterior ½ dark brown (Fig. 504).

Ratios. HE/MS/WM = 2.2/1.1/1.0; POL/OOL/POO = 4.8/2.0/1.0; OOL/DO = 1.2; WE/WF/WH/HH = 1.0/2.3/4.3/3.2; WH/WT = 1.1; PM/ST = 1.8; TS1/TS2/LT/LT1/LT2/LT3/LT4 = 4.4/2.7/6.7/2.3/1.6/1.0/1.7; LP/WP = 0.9; MM/LG = 0.9.

*Male*. Unknown.

##### Hosts and biology.

Unknown.

##### Distribution.

Costa Rica (Schauff and Janzen 2001) (Guanacaste & Heredia Provinces).

### Species with uncertain status

#### Euplectrus brasiliensis

Taxon classificationAnimaliaHymenopteraEulophidae

Ashmead

Euplectrus brasiliensis Ashmead, 1904: 516–517. Type material lost (LaSalle and Schauff 1992: 20).

##### Hosts and biology.

Unknown.

##### Distribution.

Bolivia (LaSalle and Schauff 1992).

#### Euplectrus hircinus

Taxon classificationAnimaliaHymenopteraEulophidae

(Say)

Eulophus hircinus Say, 1836: 274. Type material not located, probably destroyed (Mawdsley 1993).
Euplectrus hircinus (Say), Peck 1951: 453.

##### Material.

No specimens found.

##### Hosts and biology.

Unknown.

##### Distribution.

USA (Indiana) (Say 1836).

##### Remarks.

The morphological differences between many of the species of *Euplectrus* are small, a fact that previous species descriptors have not realized. Therefore descriptions such as the one by Say, of *Euplectrus hircinus*, fit to any number, or none, of the species. Without type material, *Euplectrus hircinus* is impossible to identify.

#### Euplectrus ronnai

Taxon classificationAnimaliaHymenopteraEulophidae

(Brèthes)

Figures 579 , 580

Heteroscapus ronnai Brèthes, 1918: 10. Lectotype ♂ (MACN), designated here.
Euplectrus ronnai (Brèthes) (De Santis 1980b: 154).

##### Material.

See below under remarks.

##### Hosts and biology.

Lepidoptera indet. (Brèthes 1918).

##### Distribution.

Brazil (Brèthes 1918).

##### Remarks.

The type material of this species is mainly destroyed, the only parts remaining (Juan José Martínez in MACN, personal communication) are one antenna each of a male and a female on slides (Figs 579, 580). As only an antenna of each sex remain it is difficult to fix the identity of *Euplectrus ronnai*. The strongly enlarged yellow scape in the male is the same as in *Euplectrus valverdei*, but some other characters are different between these species. The males of *Euplectrus ronnai* have entire antenna yellow (Fig. 580, also according to the original description), and mid tibia with apical part black (according to original description); males of *Euplectrus valverdei* have flagellomeres 1–4 yellowish-white and 5–6 pale brown, and mid tibia entirely yellowish-brown. Thus these are very probably different species. The only remaining and distinctive part of *Euplectrus ronnai* is the male antenna on slide, which also agrees with the original description, and this is hereby designated as lectotype for the species. The female antenna on slide is designated paralectotype of *Euplectrus ronnai*.

## Supplementary Material

XML Treatment for Euplectrus

XML Treatment for Euplectrus alejandrovalerioi

XML Treatment for Euplectrus alexsmithi

XML Treatment for Euplectrus alvarowillei

XML Treatment for Euplectrus andybennetti

XML Treatment for Euplectrus andydeansi

XML Treatment for Euplectrus annettewalkerae

XML Treatment for Euplectrus billbrowni

XML Treatment for Euplectrus bobwhartoni

XML Treatment for Euplectrus carlosarmientoi

XML Treatment for Euplectrus carlrettenmeyeri

XML Treatment for Euplectrus charlesmicheneri

XML Treatment for Euplectrus charlesporteri

XML Treatment for Euplectrus chrisdarlingi

XML Treatment for Euplectrus chrisgrinteri

XML Treatment for Euplectrus corriemoreauae

XML Treatment for Euplectrus daveroubiki

XML Treatment for Euplectrus davesmithi

XML Treatment for Euplectrus davidwahli

XML Treatment for Euplectrus dianariasae

XML Treatment for Euplectrus donquickei

XML Treatment for Euplectrus eowilsoni

XML Treatment for Euplectrus garygibsoni

XML Treatment for Euplectrus gavinbroadi

XML Treatment for Euplectrus gerarddelvarei

XML Treatment for Euplectrus henrytownesi

XML Treatment for Euplectrus howelldalyi

XML Treatment for Euplectrus hugokonsi

XML Treatment for Euplectrus iangauldi

XML Treatment for Euplectrus jacklonginoi

XML Treatment for Euplectrus jesusugaldei

XML Treatment for Euplectrus jimwhitfieldi

XML Treatment for Euplectrus jjrodriguezae

XML Treatment for Euplectrus johnheratyi

XML Treatment for Euplectrus johnlasallei

XML Treatment for Euplectrus johnnoyesi

XML Treatment for Euplectrus josefernandezi

XML Treatment for Euplectrus lubomirmasneri

XML Treatment for Euplectrus markshawi

XML Treatment for Euplectrus mikegatesi

XML Treatment for Euplectrus mikeschauffi

XML Treatment for Euplectrus mikesharkeyi

XML Treatment for Euplectrus ninazitaniae

XML Treatment for Euplectrus pammitchellae

XML Treatment for Euplectrus paulhansoni

XML Treatment for Euplectrus paulheberti

XML Treatment for Euplectrus paulhurdi

XML Treatment for Euplectrus philwardi

XML Treatment for Euplectrus robbinthorpi

XML Treatment for Euplectrus ronaldzunigai

XML Treatment for Euplectrus roysnellingi

XML Treatment for Euplectrus scottshawi

XML Treatment for Euplectrus sondrawardae

XML Treatment for Euplectrus sydneycameronae

XML Treatment for Euplectrus victoriapookae

XML Treatment for Euplectrus wonyoungchoi

XML Treatment for Euplectrus anae

XML Treatment for Euplectrus carlowae

XML Treatment for Euplectrus floryae

XML Treatment for Euplectrus hansoni

XML Treatment for Euplectrus ireneae

XML Treatment for Euplectrus ivonae

XML Treatment for Euplectrus josei

XML Treatment for Euplectrus magdae

XML Treatment for Euplectrus mariae

XML Treatment for Euplectrus orias

XML Treatment for Euplectrus platyhypenae

XML Treatment for Euplectrus ronniei

XML Treatment for Euplectrus testaceipes

XML Treatment for Euplectrus xiomarae

XML Treatment for Euplectrus catocalae

XML Treatment for Euplectrus chapadae

XML Treatment for Euplectrus comstockii

XML Treatment for Euplectrus edithae

XML Treatment for Euplectrus frontalis

XML Treatment for Euplectrus furnius

XML Treatment for Euplectrus insularis

XML Treatment for Euplectrus junctus

XML Treatment for Euplectrus leucotrophis

XML Treatment for Euplectrus marginatus

XML Treatment for Euplectrus mellipes

XML Treatment for Euplectrus pachyscaphus

XML Treatment for Euplectrus puttleri

XML Treatment for Euplectrus rojasi

XML Treatment for Euplectrus semimarginatus

XML Treatment for Euplectrus solitarius

XML Treatment for Euplectrus valverdei

XML Treatment for Euplectrus zamorai

XML Treatment for Euplectrus brasiliensis

XML Treatment for Euplectrus hircinus

XML Treatment for Euplectrus ronnai

## Figures and Tables

**Figures 27–30.**
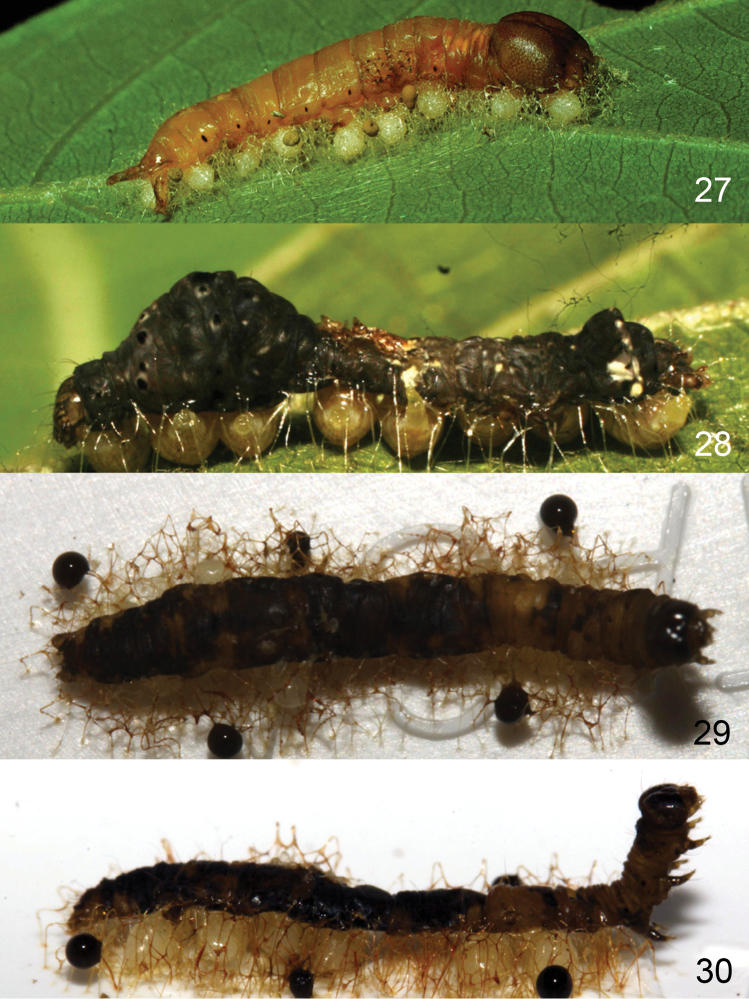
*Euplectrus* spp.: **27** pupae and cocoons of *Euplectrus xiomarae* beneath the cadaver of a penultimate instar *Hemiceras clarki* (Notodontidae) (92-SRNP-4609); the yellow balls between the pupae are meconial pellets, one defecated by each larva immediately before pupation **28** prepupae of *Euplectrus wonyoungchoi* in their partly spun cocoons beneath the cadaver of *Elaphria* Poole08 (Noctuidae) (11-SRNP-32964); they have not yet defecated their meconial pellets **29** pupae of *Euplectrus lubomirmasneri* beneath the cadaver of *Cecharismena zoum* (Erebidae) (14-SRNP-30332); the black balls are recently defecated meconial pellets **30** lateral view of the same *Euplectrus* and caterpillar as in Fig. 29.

**Figures 31–33.**
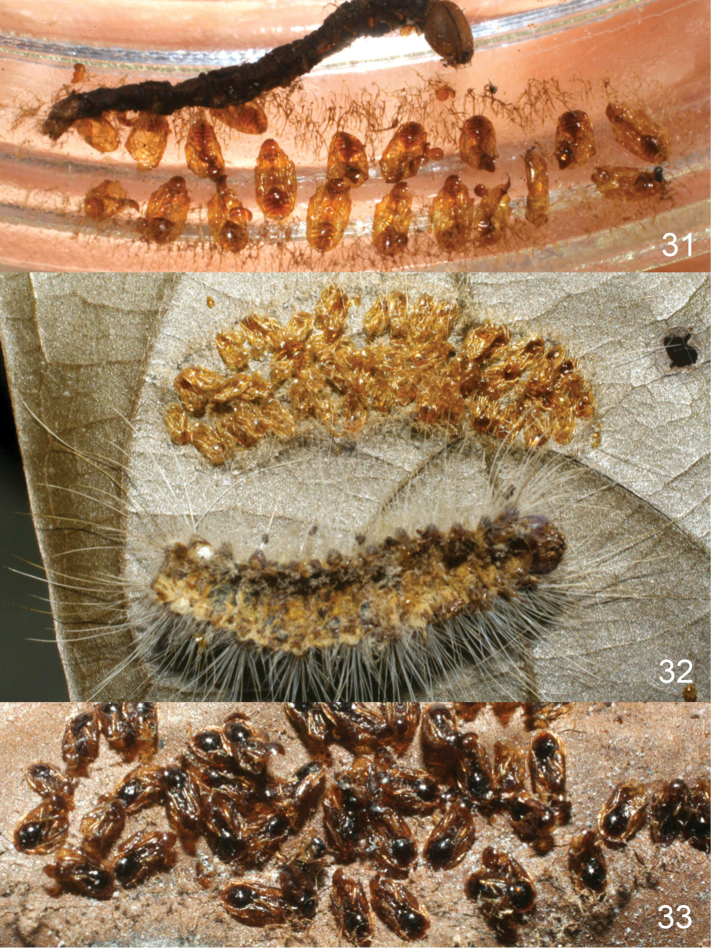
*Euplectrus* spp.: **31** newly eclosed pupal remains of *Euplectrus johnlasallei* that were below the cadaver of the caterpillar of *Sericochroa* Janzen01 (Notodontidae) (07-SRNP-31411), which has been lifted off of them as they were stuck to the surface of the glass rearing bottle to which they were glued by the drying pupal liquids, with the cocoon silk at the sides and the cadaver as the roof of the pupal chamber **32** recently eclosed pupal remains of *Euplectrus fernandezi* glued to the leaf below the cadaver of a last instar *Euglyphis jessiehillae* (Lasiocampidae) (06-SRNP-4798); the hairy caterpillar has been lifted off the pupae and this species of *Euplectrus* appears to not spin a silken cocoon as do all the other reared ACG *Euplectrus* **33** recently eclosed pupal remains of *Euplectrus fernandezi* glued to the leaf, with no visible silk, beneath the hairy cadaver of a last instar *Euglyphis jessiehillae* (Lasiocampidae) (07-SRNP-2713).

**Figures 610–615.**
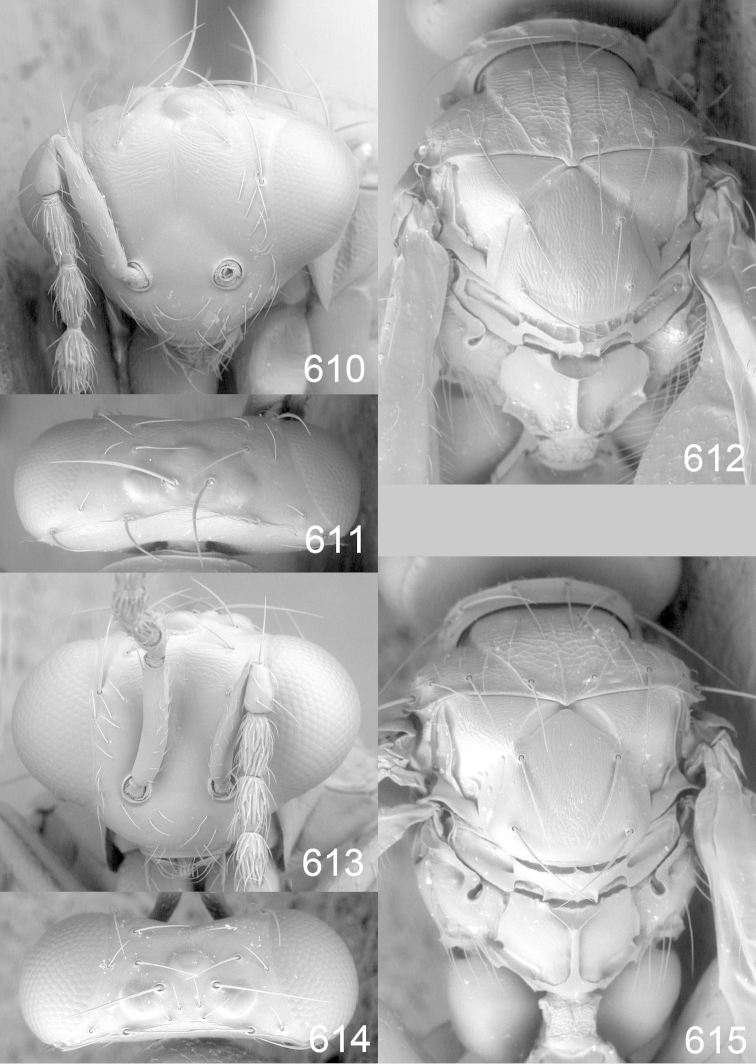
*Euplectrus* spp., females. **610–612** *Euplectrus ivonae* (no codes): **610** head in frontal view **611** vertex **612** mesosoma in dorsal view **613–615** *Euplectrus josei* (non-types): **613** head in frontal view **614** vertex **615** mesosoma in dorsal view.

## Supplementary materials

Supplementary material 1 Neighbour Joining (NJ) tree for all specimens of the DNA barcoded *Euplectrus* Data type: Adobe PDF file Explanation note: This NJ tree includes all *Euplectrus* specimens with a barcode and supplements the NJ tree (Fig. 35) that includes only one representative barcode per species. This dataset is made available under the Open Database License (http://opendatacommons.org/licenses/odbl/1.0/). The Open Database License (ODbL) is a license agreement intended to allow users to freely share, modify, and use this Dataset while maintaining this same freedom for others, provided that the original source and author(s) are credited.Christer Hansson, M. Alex Smith, Daniel H. Janzen, Winnie Hallwachs

## References

[B1] Ashmead WH (1885) Studies on North American Chalcididae, with descriptions of new species from Florida. Transactions of the American Entomological Society 12: x–xix.

[B2] Ashmead WH (1904) Classification of the chalcid flies of the superfamily Chalcidoidea, with descriptions of new species in the Carnegie Museum, collected in South America by Herbert H. Smith. Memoirs of the Carnegie Museum 1(4): i–xi, 225–551, 39 pls.

[B3] Bouček Z (1977) Descriptions of two new species of Neotropical Eulophidae (Hymenoptera) of economic interest, with taxonomic notes on related species and genera. Bulletin of Entomological Research 67(1): 1–15. doi: 10.1017/S0007485300010841

[B4] Brèthes J (1918) Sobre algunos Hymenópteres útiles del sud del Brasil. Anales Sociedad Rural Argentina 52(1): 7–11.

[B5] Burks BD (1979) Family Eulophidae. In: Krombein KV Hurd PD Smith DR Burks BD Catalog of Hymenoptera in America north of Mexico. Smithsonian Institution Press, Washington D.C., 1198 pp.

[B6] Cameron P (1904) New Hymenoptera, mostly from Nicaragua. Invertebrata Pacifica 1: 46–69.

[B7] Cameron P (1913) The Hymenoptera of the Georgetown Museum. Part V Timehri, Guyana (3) 3: 105–137.

[B8] Chattergee PN (1945) On the biology and morphology of *Euplectrus parvulus* Ferr. (Hymenoptera, Eulophidae). Indian Journal of Entomology 6: 95–101.

[B9] Clausen CP (1940) Entomophagous Insects. McGraw-Hill, New York, 688 pp.

[B10] Coudron TA Puttler B (1988) Response of natural and factitious hosts to the ectoparasite *Euplectrus plathypenae* (Hymenoptera: Eulophidae). Annals of the Entomological Society of America 81: 931–937. doi: 10.1093/aesa/81.6.931

[B11] Coudron TA Jones D Jones G (1994) Premature production of late larval storage proteins in larvae of *Trichoplusia ni* by *Euplectrus comstockii*. Archives of Insect Biochemistry and Physiology 26: 97–109. doi: 10.1002/arch.940260204

[B12] Dahlbom AG (1857) Svenska Små-Ichneumonernas familjer och slägten. Öfversigt af Kongl. Vetenskaps-Akademiens Förhandlingar 14: 289–298.

[B13] Dalla Torre KW von (1898) Catalogus Hymenopterorum hucusque descriptorum systematicus et synonymicus. V. Chalcididae et Proctotrupidae. Lepzig, 598 pp.

[B14] De Santis L (1967) Catálogo de los Himenópteros Argentinos de la Serie Parasitica, incluyendo Bethyloidea. Comision de Investigacion Cientifica, La Plata, 337 pp.

[B15] De Santis L (1979) Catálogo de los himénopteros calcidoideos de América al sur de los Estados Unidos. Publicación Especial Comisión de Investigaciones Cientificas Provincia de Buenos Airies, 488 pp.

[B16] De Santis L (1980a) Catálogo de los Himenópteros Brasileños de la Serie Parasitica incluyendo Bethyloidea. Editora da Universidade Federal do Paraná, 395 pp.

[B17] De Santis L (1980b) Nueva sinonimia, nueva combinacion y nuevas citas de Himenopteros Calcidoideos para La Republica Argentina (Insecta). Neotropica 26(76): 153–154, 196.

[B18] De Santis L Fidalgo P (1994) Catálogo de Himenópteros Calcidoideos. Serie de la Academia Nacional de Agronomia y Veterinaria 13: 1–145.

[B19] Fernandez-Triana JL Whitfield JB Rodriguez JJ Smith MA Janzen DH Hallwachs W Hajibabaei M Burns JM Solis MA Brown J Cardinal S Goulet H Hebert PDN (2014) Review of *Apanteles sensu strictu* (Hymenoptera, Braconidae, Microgastrinae) from Area de Conservación Guanacaste, northwestern Costa Rica, with keys to all described species from Mesoamerica. ZooKeys 383: 1–565. doi: 10.3897/zookeys.383.6418 10.3897/zookeys.383.6418 PMC3950464 24624021

[B20] Ferrière C (1941) New species of Euplectrini (Hym. Chalcidoidea) from Europe, Africa and Asia. Bulletin of Entomological Research 32(1): 17–48. doi: 10.1017/S0007485300005198

[B21] Gahan AB Rohwer SA (1917) Lectotypes of the species of Hymenoptera (except Apoidea) described by Abbé Provancher. Canadian Entomologist 49(11): 391–400. doi: 10.4039/Ent49391-11

[B22] Gahan AB (1927) Miscellaneous descriptions of new parasitic Hymenoptera with some synonymical notes. Proceedings of the United States National Museum 71: 1–39, 1 pl. doi: 10.5479/si.00963801.71-2676.1

[B23] Gerling D Limon S (1976) A biological review of the genus *Euplectrus* (Hym.: Eulophidae) with special emphasis on *E. laphygmae* as a parasite on *Spodoptera littoralis* (Lep.: Noctuidae). Entomophaga 21: 179–187. doi: 10.1007/BF02371904

[B24] Gibson GAP (1997) In: Gibson GAP Huber JT Woolley JB Annotated Keys to the Genera of Nearctic Chalcidoidea (Hymenoptera). NRC Research Press, Ottawa, 794 pp.

[B25] Girault AA (1916) Descriptions of and observations on some chalcidoid Hymenoptera - II (continued). Canadian Entomologist 48: 265–268. doi: 10.4039/Ent48265-8

[B26] Girault AA (1917a) New chalcid flies. Private publication, Glenndale, Maryland, 6 pp.

[B27] Girault AA (1917b) Descriptions hymenopterorum chalcidoidicarum variorum cum observationibus. V. Private publication. Glenndale, Maryland, 16 pp.

[B28] Hebert PDN Cywinska A Ball SL deWaard JR (2003) Biological identifications through DNA barcodes. Proceedings of the Royal Society B 270: 313–321. doi: 10.1098/rspb.2002.2218 12614582 10.1098/rspb.2002.2218 PMC1691236

[B29] Howard LO (1880) A new silk-spinning chalcid. Canadian Entomologist 12(8): 158–159. doi: 10.4039/Ent12158-8

[B30] Howard LO (1885) Descriptions of North American Chalcididae from the collections of the U.S. Department of Agriculture and of Dr C.V. Riley, with biological notes. (First paper). Together with a list of the described North American species of the family. Bulletin of the United States Department of Agriculture, Bureau of Entomology, No 5: 1–47.

[B31] Howard LO (1897) On the Chalcididae of the Island of Grenada. Journal of the Linnean Society (Zoology) 26: 129–178.

[B32] Ivanova NV deWaard JR Hebert PDN (2006) An inexpensive, automation-friendly protocol for recovering high-quality DNA. Molecular Ecology Notes 6: 998–1002. doi: 10.1111/j.1471-8286.2006.01428.x

[B33] Janzen DH (1993) Taxonomy: universal and essential infrastructure for development and management of tropical wildland biodiversity. In: Sandlund OT Schei PJ (Eds) Proceedings of the Norway/UNEP Expert Conference on Biodiversity, Trondheim, Norway. NINA, Trondheim, Norway, 100–113.

[B34] Janzen DH Hallwachs W Blandin P Burns JM Cadiou J Chacon I Dapkey T Deans AR Epstein ME Espinoza B Franclemont JG Haber WA Hajibabaei M Hall JPW Hebert PDN Gauld ID Harvey DJ Hausmann A Kitching I Lafontaine D Landry J Lemaire C Miller JY Miller JS Miller L Miller SE Montero J Munroe E Rab Green S Ratnasingham S Rawlins JE Robbins RK Rodriguez JJ Rougerie R Sharkey MJ Smith MA Solis MA Sullivan JB Thiaucourt P Wahl DB Weller SJ Whitfield JB Willmott KR Wood DM Woodley NE Wilson JJ (2009) Integration of DNA barcoding into an ongoing inventory of complex tropical biodiversity. Molecular Ecology Resources 9 (Supplement 1): 1–26. doi: 10.1111/j.1755-0998.2009.02628.x 21564960 10.1111/j.1755-0998.2009.02628.x

[B35] Kerr PH Fisher EM Buffington M (2008) Dome lighting for insect imaging under a microscope. American Entomologist 54: 198–200.

[B36] Kerrich GJ (1974) Systematic studies on Eulophidae of economic significance (Hymenoptera, Chalcidoidea). Bulletin of Entomological Research 63(4): 629–639. doi: 10.1017/S0007485300047866

[B37] Kieleczawa J (2006) Fundamentals of Sequencing of Difficult Templates— An Overview. Journal of Biomolecular Techniques 17: 207–217. 16870712 PMC2291785

[B38] Kimura M (1980) A simple method for estimating evolutionary rate of base substitutions through comparative studies of nucleotide sequences. Journal of Molecular Evolution 16: 111–120. doi: 10.1007/BF01731581 7463489 10.1007/BF01731581

[B39] La Salle J Schauff ME (1992) Preliminary studies on neotropical Eulophidae (Hymenoptera: Chalcidoidea): Ashmead, Cameron, Howard and Walker species. Contributions of the American Entomological Institute 27(1): 1–47.

[B40] Mawdsley JR (1993) The entomological collection of Thomas Say. Psyche 100: 163–171. doi: 10.1155/1993/59616

[B41] Noyes JS (1982) Collecting and preserving chalcid wasps (Hymenoptera: Chalcidoidea). Journal of Natural History 16: 315–334. doi: 10.1080/00222938200770261

[B42] Noyes JS (2014) Universal Chalcidoidea Database. World Wide Web electronic publication. http://www.nhm.ac.uk/chalcidoids [accessed July 2014]

[B43] Peck O (1951) Superfamily Chalcidoidea. In: Muesebeck CFW Krombein KV Townes HK (Eds) Hymenoptera of America north of Mexico - synoptic catalog.). Agriculture Monographs U.S. Department of Agriculture 2: 410–594.

[B44] Peck O (1963) A catalogue of the Nearctic Chalcidoidea (Insecta; Hymenoptera). Canadian Entomologist (Supplement) 30: 1–1092.

[B45] Provancher L (1887) Faune entomologique de Canada, 2. Additions et corrections à la faune Hymenoptèrologique de la province de Québec, 165–272.

[B46] Puttler B Gordh G Long SH (1980) Bionomics of *Euplectrus puttleri*, new species, an introduced parasite of the velvetbean caterpillar, *Anticarsia gemmatalis*, from South America. Annals of the Entomological Society of America 73(1): 2–35. doi: 10.1093/aesa/73.1.28

[B47] Quicke DLJ Smith MA Janzen DH Hallwachs W Fernandez-Triana J Laurenne NM Zaldívar-Riverón A Shaw M Broad GR Klopfstein S Shaw SR Hrcek J Hebert PN Miller SE Rodriguez JJ Whitfield JB Sharkey MJ Sharanowski BJ Jussila R Gauld ID Chesters D Vogler AP (2012) Utility of the DNA barcoding gene fragment for parasitic wasp phylogeny (Hymenoptera: Ichneumonoidea): data release and new measure of taxonomic congruence. Molecular Ecology Resources 12: 676–685. doi: 10.1111/j.1755-0998.2012.03143.x 22487608 10.1111/j.1755-0998.2012.03143.x

[B48] Ratnasingham S Hebert PDN (2007) BOLD: The Barcode of Life Data System (www.barcodinglife.org). Molecular Ecology Notes 7: 355–364. doi: 10.1111/j.1471-8286.2007.01678.x 18784790 10.1111/j.1471-8286.2007.01678.x PMC1890991

[B49] Saitou N Nei M (1987) The neighbor-joining method: A new method for reconstructing phylogenetic trees. Molecular Biology and Evolution 4: 406–425. 3447015 10.1093/oxfordjournals.molbev.a040454

[B50] Say T (1836) Descriptions of new North American Hymenoptera, and observations on some already described. Boston Journal of Natural History 1: 151–203, 210–305.

[B51] Schauff ME Janzen DH (2001) Taxonomy and ecology of Costa Rican *Euplectrus* (Hymenoptera: Eulophidae), parasitoids of caterpillars. Journal of Hymenoptera Research 10: 181–230.

[B52] Smith MA Woodley NE Janzen DH Hallwachs W Hebert PDN (2006) DNA barcodes reveal cryptic host-specificity within the presumed polyphagous members of a genus of parasitoid flies (Diptera: Tachinidae). Proceedings of the National Academy of Sciences 103: 3657–3662. doi: 10.1073/pnas.0511318103 10.1073/pnas.0511318103 PMC1383497 16505365

[B53] Smith MA Wood DM Janzen DH Hallwachs W Hebert PDN (2007) DNA barcodes affirm that 16 species of apparently generalist tropical parasitoid flies (Diptera, Tachinidae) are not all generalists. Proceedings of the National Academy of Sciences 104: 4967–4972. doi: 10.1073/pnas.0700050104 10.1073/pnas.0700050104 PMC1821123 17360352

[B54] Smith MA Rodriguez JJ Whitfield JB Deans AR Janzen DH Hallwachs W Hebert PDN (2008) Extreme diversity of tropical parasitoid wasps exposed by iterative integration of natural history, DNA barcoding, morphology, and collections. Proceedings of the National Academy of Sciences 105: 12359–12364. doi: 10.1073/pnas.0805319105 10.1073/pnas.0805319105 PMC2518452 18716001

[B55] Smith DR Janzen DH Hallwachs W Smith MA (2012) Hyperparasitoid wasps (Hymenoptera, Trigonalidae) reared from dry forest and rain forest caterpillars of Area de Conservación Guanacaste, Costa Rica. Journal of Hymenoptera Research 29: 119–144. doi: 10.3897/JHR.29.3233

[B56] Swederus NS (1795) Beskrifning på et nytt genus Pteromalus ibland Insecterna, hörande til Hymenoptera. Kungliga Svenska Vetenskapsakademiens Handlingar 16: 201–205, 216–222.

[B57] Tamura K Stecher G Peterson D Filipski A Kumar S (2013) MEGA6: Molecular Evolutionary Genetics Analysis version 6.0. Molecular Biology and Evolution 30: 2725–2729. doi: 10.1093/molbev/mst197 24132122 10.1093/molbev/mst197 PMC3840312

[B58] Thomsen M (1927) Some observations on the biology and anatomy of a cocoonmaking chalcid larva, *Euplectrus bicolor* Swed. Videnskabelige meddelelser fra Dansk naturhistorisk forening i Kjøbenhavn 84: 73–90.

[B59] Walker F (1843) Descriptions of Chalcidites discovered in St. Vincent’s Isle by the Rev. Lansdown Guilding. Annals and Magazine of Natural History 12: 46–49. doi: 10.1080/03745484309442485

[B60] Westwood JO (1832) Descriptions of several new British forms amongst the parasitic hymenopterous insects. Philosophical Magazine (3) 1: 127–129.

[B61] Wijesekara GAW Schauff ME (1995) Two new genera and three new species of Euplectrini (Hymenoptera: Eulophidae) from the New World. Proceedings of the Entomological Society in Washington 99: 101–109.

