# Supplementary material for: Integrative taxonomy of New World Euplectrus Westwood (Hymenoptera, Eulophidae), with focus on 55 new species from Area de Conservación Guanacaste, northwestern Costa Rica
Source: Zookeys. 2015 Mar 10;(485):1–236. doi: 10.3897/zookeys.485.9124 (PMC4361813; doi:10.3897/zookeys.485.9124)
Supplement: Supplementary material 1 — Neighbour Joining (NJ) tree for all specimens of the DNA barcoded Euplectrus [file zookeys-485-001-s001.pdf]

BOLD TaxonID Tree

Title	:	Biological and taxonomic review of Euplectrus in ACG [DS-ASE...
Date	:	11-December-2014
Data Type	:	Nucleotide
Distance Model	:	Kimura 2 Parameter
Marker	:	COI-5P
Codon Positions	:	1st, 2nd, 3rd
Labels	:	Extra Info, SampleID, Sequence Length
Filters	:	Length > 200
Colorization	:	[blue]=Stop Codons [red]=Contamination or misidentification

Sequence Count	:	176
Species count	:	50
Genus count	:	1
Family count	:	1
Unidentified	:	0

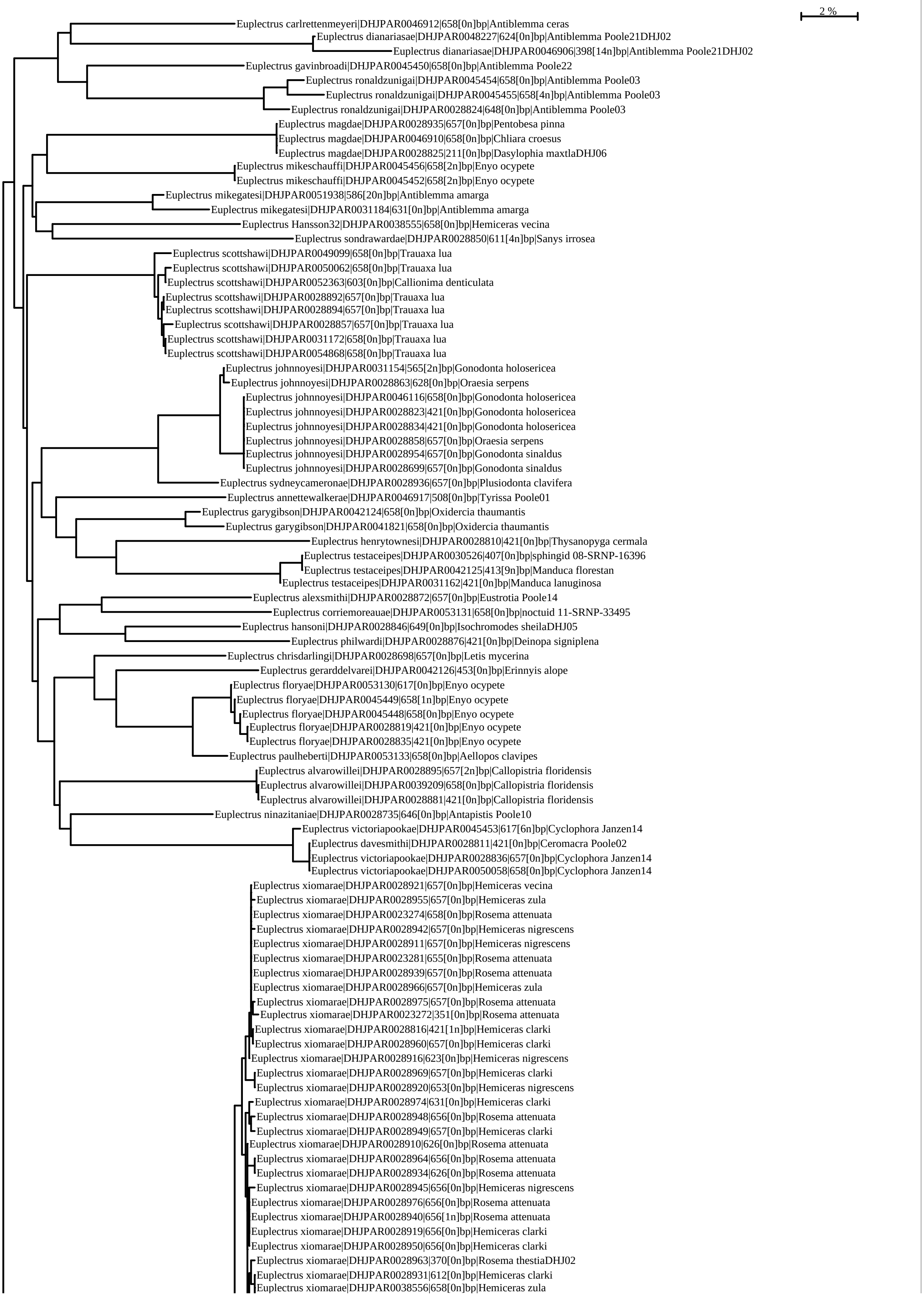
2 %
Euplectrus carlrettenmeyeri|DHJPAR0046912|658[0n]bp|Antiblemma ceras
Euplectrus dianariasae|DHJPAR0048227|624[0n]bp|Antiblemma Poole21DHJ02
Euplectrus dianariasae|DHJPAR0046906|398[14n]bp|Antiblemma Poole21DHJ02
Euplectrus gavinbroadi|DHJPAR0045450|658[0n]bp|Antiblemma Poole22
Euplectrus ronaldzunigai|DHJPAR0045454|658[0n]bp|Antiblemma Poole03
Euplectrus ronaldzunigai|DHJPAR0045455|658[4n]bp|Antiblemma Poole03
Euplectrus ronaldzunigai|DHJPAR0028824|648[0n]bp|Antiblemma Poole03
Euplectrus magdae|DHJPAR0028935|657[0n]bp|Pentobesa pinna
Euplectrus magdae|DHJPAR0046910|658[0n]bp|Chliara croesus
Euplectrus magdae|DHJPAR0028825|211[0n]bp|Dasylophia maxtlaDHJ06
Euplectrus mikeschauffi|DHJPAR0045456|658[2n]bp|Enyo ocypete
Euplectrus mikeschauffi|DHJPAR0045452|658[2n]bp|Enyo ocypete
Euplectrus mikegatesi|DHJPAR0051938|586[20n]bp|Antiblemma amarga
Euplectrus mikegatesi|DHJPAR0031184|631[0n]bp|Antiblemma amarga
Euplectrus Hansson32|DHJPAR0038555|658[0n]bp|Hemiceras vecina
Euplectrus sondrawardae|DHJPAR0028850|611[4n]bp|Sanys irrosea
Euplectrus scottshawi|DHJPAR0049099|658[0n]bp|Trauaxa lua
Euplectrus scottshawi|DHJPAR0050062|658[0n]bp|Trauaxa lua
Euplectrus scottshawi|DHJPAR0052363|603[0n]bp|Callionima denticulata
Euplectrus scottshawi|DHJPAR0028892|657[0n]bp|Trauaxa lua
Euplectrus scottshawi|DHJPAR0028894|657[0n]bp|Trauaxa lua
Euplectrus scottshawi|DHJPAR0028857|657[0n]bp|Trauaxa lua
Euplectrus scottshawi|DHJPAR0031172|658[0n]bp|Trauaxa lua
Euplectrus scottshawi|DHJPAR0054868|658[0n]bp|Trauaxa lua
Euplectrus johnnoyesi|DHJPAR0031154|565[2n]bp|Gonodonta holosericea
Euplectrus johnnoyesi|DHJPAR0028863|628[0n]bp|Oraesia serpens
Euplectrus johnnoyesi|DHJPAR0046116|658[0n]bp|Gonodonta holosericea
Euplectrus johnnoyesi|DHJPAR0028823|421[0n]bp|Gonodonta holosericea
Euplectrus johnnoyesi|DHJPAR0028834|421[0n]bp|Gonodonta holosericea
Euplectrus johnnoyesi|DHJPAR0028858|657[0n]bp|Oraesia serpens
Euplectrus johnnoyesi|DHJPAR0028954|657[0n]bp|Gonodonta sinaldus
Euplectrus johnnoyesi|DHJPAR0028699|657[0n]bp|Gonodonta sinaldus
Euplectrus sydneycameronae|DHJPAR0028936|657[0n]bp|Plusiodonta clavifera
Euplectrus annettewalkerae|DHJPAR0046917|508[0n]bp|Tyrissa Poole01
Euplectrus garygibson|DHJPAR0042124|658[0n]bp|Oxidercia thaumantis
Euplectrus garygibson|DHJPAR0041821|658[0n]bp|Oxidercia thaumantis
Euplectrus henrytownesi|DHJPAR0028810|421[0n]bp|Thysanopyga cermala
Euplectrus testaceipes|DHJPAR0030526|407[0n]bp|sphingid 08-SRNP-16396
Euplectrus testaceipes|DHJPAR0042125|413[9n]bp|Manduca florestan
Euplectrus testaceipes|DHJPAR0031162|421[0n]bp|Manduca lanuginosa
Euplectrus alexsmithi|DHJPAR0028872|657[0n]bp|Eustrotia Poole14
Euplectrus corriemoreauae|DHJPAR0053131|658[0n]bp|noctuid 11-SRNP-33495
Euplectrus hansoni|DHJPAR0028846|649[0n]bp|Isochromodes sheilaDHJ05
Euplectrus philwardi|DHJPAR0028876|421[0n]bp|Deinopa signiplena
Euplectrus chrisdarlingi|DHJPAR0028698|657[0n]bp|Letis mycerina
Euplectrus gerarddelvarei|DHJPAR0042126|453[0n]bp|Erinnyis alope
Euplectrus floryae|DHJPAR0053130|617[0n]bp|Enyo ocypete
Euplectrus floryae|DHJPAR0045449|658[1n]bp|Enyo ocypete
Euplectrus floryae|DHJPAR0045448|658[0n]bp|Enyo ocypete
Euplectrus floryae|DHJPAR0028819|421[0n]bp|Enyo ocypete
Euplectrus floryae|DHJPAR0028835|421[0n]bp|Enyo ocypete
Euplectrus paulheberti|DHJPAR0053133|658[0n]bp|Aellopos clavipes
Euplectrus alvarowillei|DHJPAR0028895|657[2n]bp|Callopistria floridensis
Euplectrus alvarowillei|DHJPAR0039209|658[0n]bp|Callopistria floridensis
Euplectrus alvarowillei|DHJPAR0028881|421[0n]bp|Callopistria floridensis
Euplectrus ninazitaniae|DHJPAR0028735|646[0n]bp|Antapistis Poole10
Euplectrus victoriapookae|DHJPAR0045453|617[6n]bp|Cyclophora Janzen14
Euplectrus davesmithi|DHJPAR0028811|421[0n]bp|Ceromacra Poole02
Euplectrus victoriapookae|DHJPAR0028836|657[0n]bp|Cyclophora Janzen14
Euplectrus victoriapookae|DHJPAR0050058|658[0n]bp|Cyclophora Janzen14
Euplectrus xiomarae|DHJPAR0028921|657[0n]bp|Hemiceras vecina
Euplectrus xiomarae|DHJPAR0028955|657[0n]bp|Hemiceras zula
Euplectrus xiomarae|DHJPAR0023274|658[0n]bp|Rosema attenuata
Euplectrus xiomarae|DHJPAR0028942|657[0n]bp|Hemiceras nigrescens
Euplectrus xiomarae|DHJPAR0028911|657[0n]bp|Hemiceras nigrescens
Euplectrus xiomarae|DHJPAR0023281|655[0n]bp|Rosema attenuata
Euplectrus xiomarae|DHJPAR0028939|657[0n]bp|Rosema attenuata
Euplectrus xiomarae|DHJPAR0028966|657[0n]bp|Hemiceras zula
Euplectrus xiomarae|DHJPAR0028975|657[0n]bp|Rosema attenuata
Euplectrus xiomarae|DHJPAR0023272|351[0n]bp|Rosema attenuata
Euplectrus xiomarae|DHJPAR0028816|421[1n]bp|Hemiceras clarki
Euplectrus xiomarae|DHJPAR0028960|657[0n]bp|Hemiceras clarki
Euplectrus xiomarae|DHJPAR0028916|623[0n]bp|Hemiceras nigrescens
Euplectrus xiomarae|DHJPAR0028969|657[0n]bp|Hemiceras clarki
Euplectrus xiomarae|DHJPAR0028920|653[0n]bp|Hemiceras nigrescens
Euplectrus xiomarae|DHJPAR0028974|631[0n]bp|Hemiceras clarki
Euplectrus xiomarae|DHJPAR0028948|656[0n]bp|Rosema attenuata
Euplectrus xiomarae|DHJPAR0028949|657[0n]bp|Hemiceras clarki
Euplectrus xiomarae|DHJPAR0028910|626[0n]bp|Rosema attenuata
Euplectrus xiomarae|DHJPAR0028964|656[0n]bp|Rosema attenuata
Euplectrus xiomarae|DHJPAR0028934|626[0n]bp|Rosema attenuata
Euplectrus xiomarae|DHJPAR0028945|656[0n]bp|Hemiceras nigrescens
Euplectrus xiomarae|DHJPAR0028976|656[0n]bp|Rosema attenuata
Euplectrus xiomarae|DHJPAR0028940|656[1n]bp|Rosema attenuata
Euplectrus xiomarae|DHJPAR0028919|656[0n]bp|Hemiceras clarki
Euplectrus xiomarae|DHJPAR0028950|656[0n]bp|Hemiceras clarki
Euplectrus xiomarae|DHJPAR0028963|370[0n]bp|Rosema thestiaDHJ02
Euplectrus xiomarae|DHJPAR0028931|612[0n]bp|Hemiceras clarki
Euplectrus xiomarae|DHJPAR0038556|658[0n]bp|Hemiceras zula

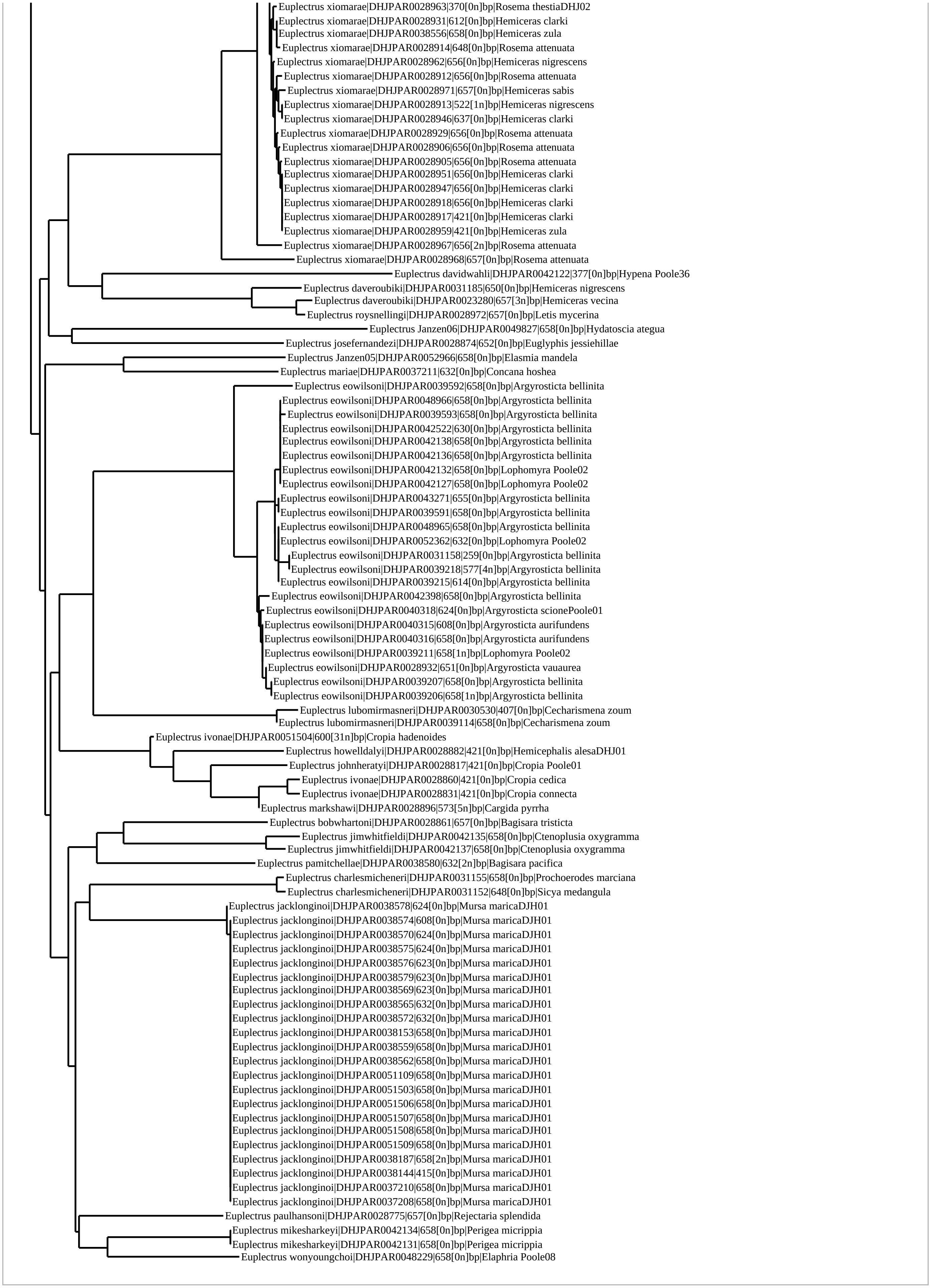
Euplectrus xiomarae|DHJPAR0028963|370[0n]bp|Rosema thestiaDHJ02
Euplectrus xiomarae|DHJPAR0028931|612[0n]bp|Hemiceras clarki
Euplectrus xiomarae|DHJPAR0038556|658[0n]bp|Hemiceras zula
Euplectrus xiomarae|DHJPAR0028914|648[0n]bp|Rosema attenuata
Euplectrus xiomarae|DHJPAR0028962|656[0n]bp|Hemiceras nigrescens
Euplectrus xiomarae|DHJPAR0028912|656[0n]bp|Rosema attenuata
Euplectrus xiomarae|DHJPAR0028971|657[0n]bp|Hemiceras sabis
Euplectrus xiomarae|DHJPAR0028913|522[1n]bp|Hemiceras nigrescens
Euplectrus xiomarae|DHJPAR0028946|637[0n]bp|Hemiceras clarki
Euplectrus xiomarae|DHJPAR0028929|656[0n]bp|Rosema attenuata
Euplectrus xiomarae|DHJPAR0028906|656[0n]bp|Rosema attenuata
Euplectrus xiomarae|DHJPAR0028905|656[0n]bp|Rosema attenuata
Euplectrus xiomarae|DHJPAR0028951|656[0n]bp|Hemiceras clarki
Euplectrus xiomarae|DHJPAR0028947|656[0n]bp|Hemiceras clarki
Euplectrus xiomarae|DHJPAR0028918|656[0n]bp|Hemiceras clarki
Euplectrus xiomarae|DHJPAR0028917|421[0n]bp|Hemiceras clarki
Euplectrus xiomarae|DHJPAR0028959|421[0n]bp|Hemiceras zula
Euplectrus xiomarae|DHJPAR0028967|656[2n]bp|Rosema attenuata
Euplectrus xiomarae|DHJPAR0028968|657[0n]bp|Rosema attenuata
Euplectrus davidwahli|DHJPAR0042122|377[0n]bp|Hypena Poole36
Euplectrus daveroubiki|DHJPAR0031185|650[0n]bp|Hemiceras nigrescens
Euplectrus daveroubiki|DHJPAR0023280|657[3n]bp|Hemiceras vecina
Euplectrus roysnellingi|DHJPAR0028972|657[0n]bp|Letis mycerina
Euplectrus Janzen06|DHJPAR0049827|658[0n]bp|Hydatoscia ategua
Euplectrus josefernandezi|DHJPAR0028874|652[0n]bp|Euglyphis jessiehillae
Euplectrus Janzen05|DHJPAR0052966|658[0n]bp|Elasmia mandela
Euplectrus mariae|DHJPAR0037211|632[0n]bp|Concana hoshea
Euplectrus eowilsoni|DHJPAR0039592|658[0n]bp|Argyrosticta bellinita
Euplectrus eowilsoni|DHJPAR0048966|658[0n]bp|Argyrosticta bellinita
Euplectrus eowilsoni|DHJPAR0039593|658[0n]bp|Argyrosticta bellinita
Euplectrus eowilsoni|DHJPAR0042522|630[0n]bp|Argyrosticta bellinita
Euplectrus eowilsoni|DHJPAR0042138|658[0n]bp|Argyrosticta bellinita
Euplectrus eowilsoni|DHJPAR0042136|658[0n]bp|Argyrosticta bellinita
Euplectrus eowilsoni|DHJPAR0042132|658[0n]bp|Lophomyra Poole02
Euplectrus eowilsoni|DHJPAR0042127|658[0n]bp|Lophomyra Poole02
Euplectrus eowilsoni|DHJPAR0043271|655[0n]bp|Argyrosticta bellinita
Euplectrus eowilsoni|DHJPAR0039591|658[0n]bp|Argyrosticta bellinita
Euplectrus eowilsoni|DHJPAR0048965|658[0n]bp|Argyrosticta bellinita
Euplectrus eowilsoni|DHJPAR0052362|632[0n]bp|Lophomyra Poole02
Euplectrus eowilsoni|DHJPAR0031158|259[0n]bp|Argyrosticta bellinita
Euplectrus eowilsoni|DHJPAR0039218|577[4n]bp|Argyrosticta bellinita
Euplectrus eowilsoni|DHJPAR0039215|614[0n]bp|Argyrosticta bellinita
Euplectrus eowilsoni|DHJPAR0042398|658[0n]bp|Argyrosticta bellinita
Euplectrus eowilsoni|DHJPAR0040318|624[0n]bp|Argyrosticta scionePoole01
Euplectrus eowilsoni|DHJPAR0040315|608[0n]bp|Argyrosticta aurifundens
Euplectrus eowilsoni|DHJPAR0040316|658[0n]bp|Argyrosticta aurifundens
Euplectrus eowilsoni|DHJPAR0039211|658[1n]bp|Lophomyra Poole02
Euplectrus eowilsoni|DHJPAR0028932|651[0n]bp|Argyrosticta vauaurea
Euplectrus eowilsoni|DHJPAR0039207|658[0n]bp|Argyrosticta bellinita
Euplectrus eowilsoni|DHJPAR0039206|658[1n]bp|Argyrosticta bellinita
Euplectrus lubomirmasneri|DHJPAR0030530|407[0n]bp|Cecharismena zoum
Euplectrus lubomirmasneri|DHJPAR0039114|658[0n]bp|Cecharismena zoum
Euplectrus ivonae|DHJPAR0051504|600[31n]bp|Cropia hadenoides
Euplectrus howelldalyi|DHJPAR0028882|421[0n]bp|Hemicephalis alesaDHJ01
Euplectrus johnheratyi|DHJPAR0028817|421[0n]bp|Cropia Poole01
Euplectrus ivonae|DHJPAR0028860|421[0n]bp|Cropia cedica
Euplectrus ivonae|DHJPAR0028831|421[0n]bp|Cropia connecta
Euplectrus markshawi|DHJPAR0028896|573[5n]bp|Cargida pyrrha
Euplectrus bobwhartoni|DHJPAR0028861|657[0n]bp|Bagisara tristicta
Euplectrus jimwhitfieldi|DHJPAR0042135|658[0n]bp|Ctenoplusia oxygramma
Euplectrus jimwhitfieldi|DHJPAR0042137|658[0n]bp|Ctenoplusia oxygramma
Euplectrus pamitchellae|DHJPAR0038580|632[2n]bp|Bagisara pacifica
Euplectrus charlesmicheneri|DHJPAR0031155|658[0n]bp|Prochoerodes marciana
Euplectrus charlesmicheneri|DHJPAR0031152|648[0n]bp|Sicya medangula
Euplectrus jacklonginoi|DHJPAR0038578|624[0n]bp|Mursa maricaDJH01
Euplectrus jacklonginoi|DHJPAR0038574|608[0n]bp|Mursa maricaDJH01
Euplectrus jacklonginoi|DHJPAR0038570|624[0n]bp|Mursa maricaDJH01
Euplectrus jacklonginoi|DHJPAR0038575|624[0n]bp|Mursa maricaDJH01
Euplectrus jacklonginoi|DHJPAR0038576|623[0n]bp|Mursa maricaDJH01
Euplectrus jacklonginoi|DHJPAR0038579|623[0n]bp|Mursa maricaDJH01
Euplectrus jacklonginoi|DHJPAR0038569|623[0n]bp|Mursa maricaDJH01
Euplectrus jacklonginoi|DHJPAR0038565|632[0n]bp|Mursa maricaDJH01
Euplectrus jacklonginoi|DHJPAR0038572|632[0n]bp|Mursa maricaDJH01
Euplectrus jacklonginoi|DHJPAR0038153|658[0n]bp|Mursa maricaDJH01
Euplectrus jacklonginoi|DHJPAR0038559|658[0n]bp|Mursa maricaDJH01
Euplectrus jacklonginoi|DHJPAR0038562|658[0n]bp|Mursa maricaDJH01
Euplectrus jacklonginoi|DHJPAR0051109|658[0n]bp|Mursa maricaDJH01
Euplectrus jacklonginoi|DHJPAR0051503|658[0n]bp|Mursa maricaDJH01
Euplectrus jacklonginoi|DHJPAR0051506|658[0n]bp|Mursa maricaDJH01
Euplectrus jacklonginoi|DHJPAR0051507|658[0n]bp|Mursa maricaDJH01
Euplectrus jacklonginoi|DHJPAR0051508|658[0n]bp|Mursa maricaDJH01
Euplectrus jacklonginoi|DHJPAR0051509|658[0n]bp|Mursa maricaDJH01
Euplectrus jacklonginoi|DHJPAR0038187|658[2n]bp|Mursa maricaDJH01
Euplectrus jacklonginoi|DHJPAR0038144|415[0n]bp|Mursa maricaDJH01
Euplectrus jacklonginoi|DHJPAR0037210|658[0n]bp|Mursa maricaDJH01
Euplectrus jacklonginoi|DHJPAR0037208|658[0n]bp|Mursa maricaDJH01
Euplectrus paulhansoni|DHJPAR0028775|657[0n]bp|Rejectaria splendida
Euplectrus mikesharkeyi|DHJPAR0042134|658[0n]bp|Perigea micrippia
Euplectrus mikesharkeyi|DHJPAR0042131|658[0n]bp|Perigea micrippia
Euplectrus wonyoungchoi|DHJPAR0048229|658[0n]bp|Elaphria Poole08
